# Replacement of the L-iduronic acid unit of the anticoagulant pentasaccharide idraparinux by a 6-deoxy-L-talopyranose – Synthesis and conformational analysis

**DOI:** 10.1038/s41598-018-31854-z

**Published:** 2018-09-13

**Authors:** Fruzsina Demeter, Tamás Gyöngyösi, Zsuzsanna Bereczky, Katalin E. Kövér, Mihály Herczeg, Anikó Borbás

**Affiliations:** 1 Department of Pharmaceutical Chemistry, University of Debrecen, Egyetem tér 1, Debrecen, 4032 Hungary; 2 Department of Inorganic and Analytical Chemistry, University of Debrecen, P.O. Box 400, Debrecen, 4002 Hungary; 3 Division of Clinical Laboratory Sciences, Department of Laboratory Medicine, Faculty of Medicine, University of Debrecen, 98 Nagyerdei krt., Debrecen, 4032 Hungary

## Abstract

One critical part of the synthesis of heparinoid anticoagulants is the creation of the L-iduronic acid building block featured with unique conformational plasticity which is crucial for the anticoagulant activity. Herein, we studied whether a much more easily synthesizable sugar, the 6-deoxy-L-talose, built in a heparinoid oligosaccharide, could show a similar conformational plasticity, thereby can be a potential substituent of the L-idose. Three pentasaccharides related to the synthetic anticoagulant pentasaccharide idraparinux were prepared, in which the L-iduronate was replaced by a 6-deoxy-L-talopyranoside unit. The *talo*-configured building block was formed by C4 epimerisation of the commercially available L-rhamnose with high efficacy at both the monosaccharide and the disaccharide level. The detailed conformational analysis of these new derivatives, differing only in their methylation pattern, was performed and the conformationally relevant NMR parameters, such as proton-proton coupling constants and interproton distances were compared to the corresponding ones measured in idraparinux. The lack of anticoagulant activity of these novel heparin analogues could be explained by the biologically not favorable ^1^C_4_ chair conformation of their 6-deoxy-L-talopyranoside residues.

## Introduction

Venous thromboembolism is a major cause of mortality and morbidity in the western countries, and epidemiological studies indicate that the aging of the population will increase the incidence of this illness worldwide^1,2^. Medical therapy for venous thromboembolism has been limited to the use of the thrombin inhibitor heparin and the vitamin K antagonists 4-hydroxycoumarins (e.g. warfarin) over 70 years since the 1930’s^3^. Then, the 21st century opened a new era for the anticoagulant treatment. The first breakthrough was the approval of fondaparinux, the synthetic analogue of the antithrombin-binding pentasaccharide domain of heparin as a new antithrombotic drug. Fondaparinux is an indirect, selective factor Xa inhibitor possessing a higher safety profile and a longer elimination half-life compared to the animal-originated heparin products^4^. Just a few years later new oral anticoagulant drugs, the direct thrombin inhibitor dabigatran etexilate and the direct factor Xa inhibitors rivaroxaban, apixaban and edoxaban have been approved for clinical use, revolutionising the anticoagulant therapy^5^. Although these new oral anticoagulants have major pharmacologic advantages over vitamin K antagonists, they also have drawbacks and are not approved for some clinical situations^6–8^. These data predict that the classic anticoagulant drugs, particularly the heparin derivatives will continue to be important medicines for antithrombotic therapy^9^.

In the field of heparinoid anticoagulants, current research focuses on unmet issues such as low oral bioavailability of heparin^10^, lack of specific antidote for low molecular weight heparins^11^, and chemoenzymatic production of heparin oligosaccharides^12–15^. Furthermore, many research efforts have been devoted to the development of highly efficient synthetic routes to the outstanding anticoagulant pentasaccharides fondaparinux^16–18^ and idraparinux^19,20^ as well as the preparation of various analogues of the antithrombin-binding pentasaccharide unit of heparin as novel anticoagulant candidates^21–25^.

For a successful synthesis of heparin oligosaccharides a number of factors must be considered such as access to L-idose or L-iduronic acid (IdoA) unit, the choice of uronic acid or the corresponding non-oxidised precursor as building blocks, stereochemical control in glycosylation, suitable protecting-group strategy and efficient assembly of the backbone sequence^26,27^. A range of synthetic approaches have been described to generate heparinoids including solid-supported synthesis^28^, modular approach^29,30^ and nonglycosylating strategy^19^. To avoid the inherent low reactivity and base-sensitivity of uronic acid donors, typically the corresponding glycopyranosides are used as glycosyl donors and the formation of the uronic acid can be performed at the disaccharide level^31^ or by TEMPO-mediated late-stage oxidation at the higher oligosaccharide level^20,32^.

**Figure 1:**
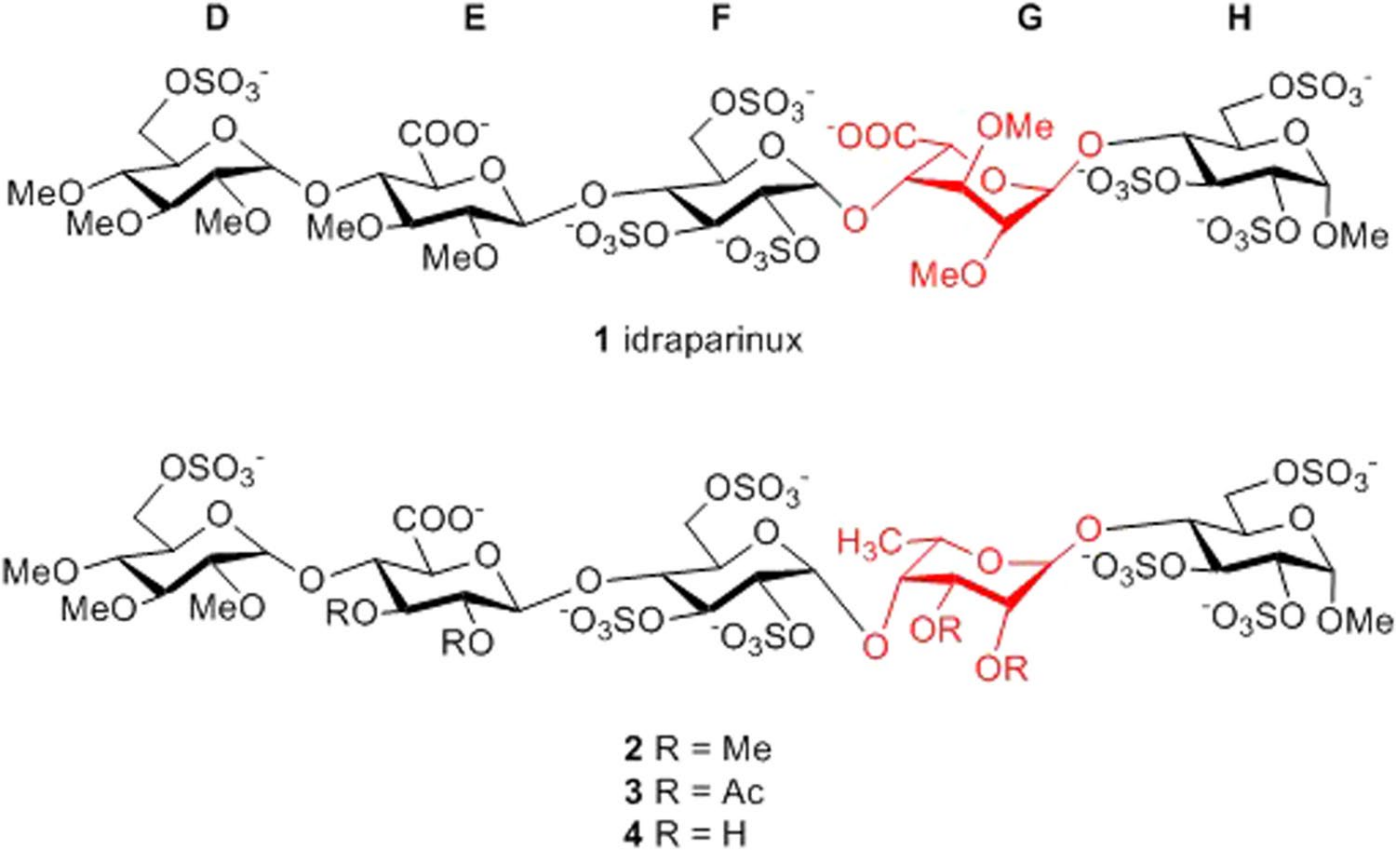
The synthetic anticoagulant pentasaccharide idraparinux (**1**) and the planned analogues **2**–**4** containing a 6-deoxy-L-talopyranoside instead of the L-iduronic acid (unit **G**).

However, each strategy toward chemical synthesis of heparin oligosaccharides faces the same difficulty: the lengthy, laborious and low-yielding synthesis of the L-iduronic acid building block, which is a critically important structural component for the anticoagulant activity. Despite recent progress^33–35^, the short and efficient synthesis of an orthogonally protected L-idose or iduronic acid glycosyl donor useful for heparinoid synthesis remained unmet. We envisioned that replacing the IdoA with a more easily available sugar unit would solve the problem and L-talopyranuronic acid could be a good candidate as a potential structural substituent. It is known, that a unique conformational plasticity of the L-iduronic acid, shift the ^1^*C*_4_ - ^2^*S*_*O*_ equilibrium to the bioactive ^2^*S*_*O*_ skew-boat conformation, is required for the antithrombotic activity^36^ and it was also shown, that its conformation is regulated by the sulphation pattern of nearby saccharides^14,37^. We assume that L-talose, which only differs from L-idose in the C3 configuration, can also adopt the required skew-boat conformation. Moreover, an attractive, short synthesis of L-talopyranosyl thioglycoside, ready for glycosylation has been developed recently^38^. This method, based on iridium-catalyzed CH-activation of the corresponding 6-deoxy derivative^39^ can potentially utilize in the synthesis of the L-talopyranuronic acid-containing heparinoid oligosaccharides. As a first step towards this goal we decided to prepare idraparinux-analogue pentasaccharides in which the iduronic acid unit is substituted by a 6-deoxy-L-talopyranoside, a very easily accessible 6-deoxy-L-hexose (Fig. 1). Idraparinux (**1**) is a synthetic anticoagulant pentasaccharide based on the heparin binding domain possessing a higher anti-Xa activity and a longer half-life than the synthetic anticoagulant drug fondaparinux^4^. Being a fully *O*-sulfated, *O*-methylated non-glycosaminoglycan analogue, its synthesis is easier than that of fondaparinux, which makes it an ideal model compound. Although the 6-deoxy-L-talopyranoside lacks the biologically important carboxylate moiety, it is suitable for studying the conformational behavior of a talopyranose built in the highly sulphated pentasaccharide. Herein, we report the synthesis and NMR-based conformational analysis of three idraparinux analogue pentasaccharides (**2**–**4**) in which the iduronic acid unit (unit **G**) is substituted by a 6-deoxy-L-talopyranoside moiety (Fig. 1).

## Results

**Figure 2:**
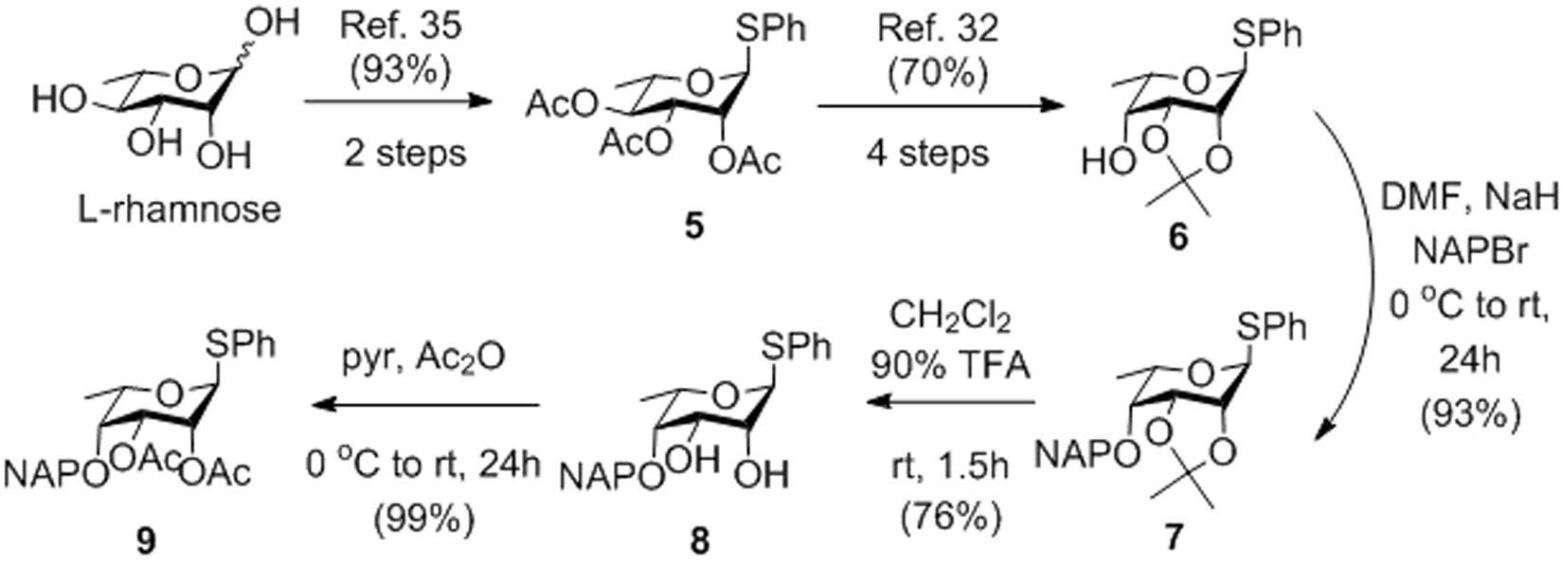
Synthesis of the 6-deoxy-L-talopyranoside donor (NAP: (2-naphthyl)methyl).

Over the last years, we have developed several new methods for the synthesis of idraparinux^20,40,41^. The most efficient strategy involves a 3 + 2 coupling of a **FGH** trisaccharide acceptor and a **DE** disaccharide donor, and application of acetyl groups to mask the hydroxyls to be methylated and benzyl ethers to protect the hydroxyls to be sulfated in the final product^20^. We applied the same strategy for the synthesis of compounds **2**–**4**. The preparation of the orthogonally protected 6-deoxy-L-talopyranosyl glycosyl donor **9** was accomplished from the commercially available and cheap 6-deoxy L-hexose, L-rhamnose (Fig. 2). Peracetylation and thioglycosylation of the starting L-rhamnose gave **5**^42^, which was converted to the L-talo-configured **6**^39^ by the well-established C4 epimerization method involving oxidation followed by stereoselective reduction of the corresponding 2,3-*O*-acetalated rhamnose derivative^43^. Protection of the 4-OH by (2-naphthyl)methylation gave **7** in 93% yield. Finally, the isopropylidene protecting group was replaced by ester groups by deacetalation followed by acetylation to result in the 6-deoxy-L-talopyranoside donor **9** having a C2 participating group capable of ensuring the desired 1,2-trans-selectivity upon glycosylation.

**Figure 3:**
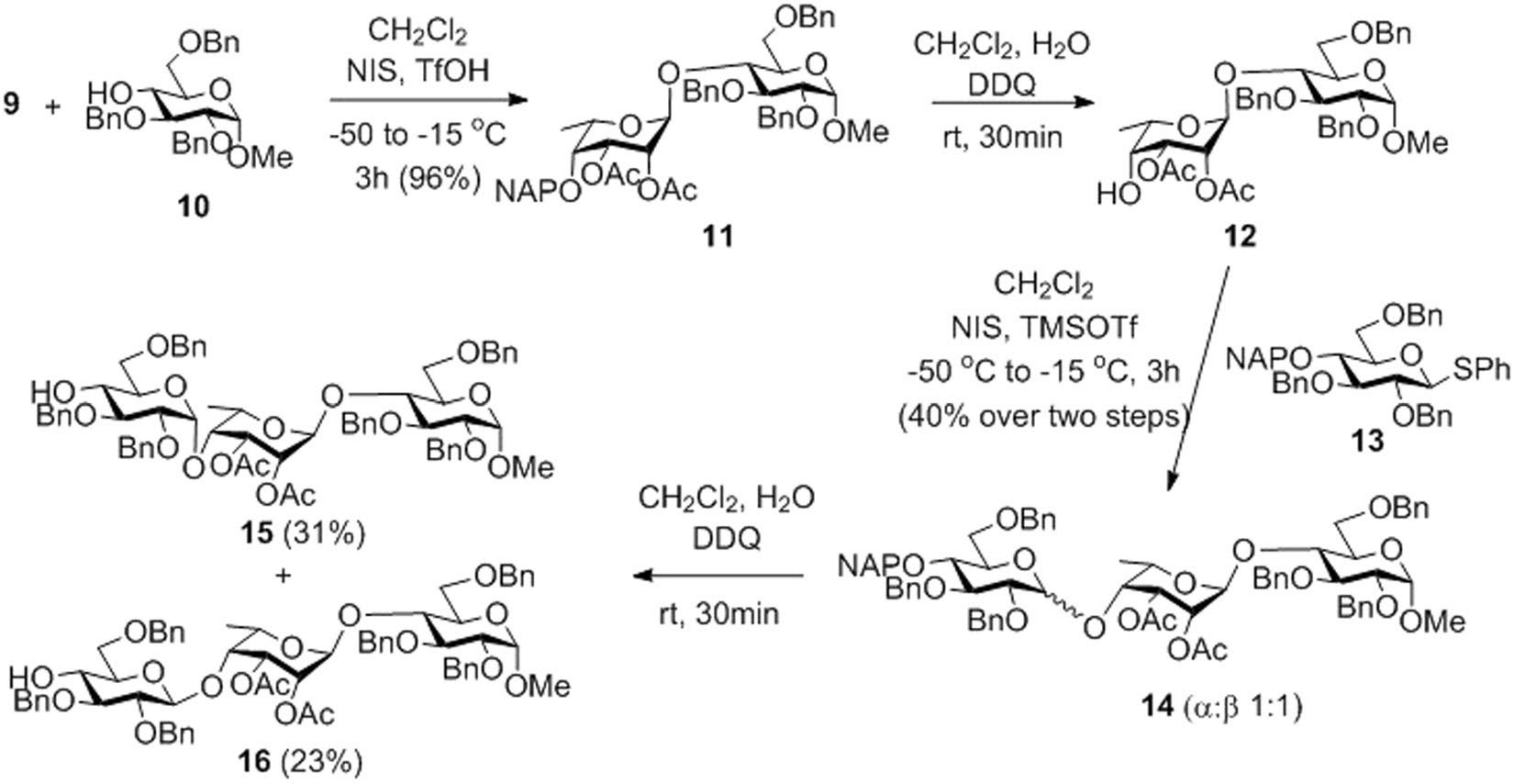
Synthesis of the **FGH** trisaccharide acceptor **15** by applying the 2,3-di-*O*-acetylated 6d-talose donor **9**.

Glycosylation of acceptor **10**^44^ with donor **9** in the presence of *N*-iodosuccinimide (NIS) and trifluoromethanesulfonic acid (TfOH) led to the formation of the expected 1,2-trans-α-linked disaccharide **11** in 96% yield (Fig. 3). Oxidative cleavage of the 4′-*O*-(2-naphthyl)methyl (NAP) group using 2,3-dichloro-5,6-dicyanobenzoquinone (DDQ)^45^ gave the disaccharide acceptor **12**. This disaccharide was isolated together with a small amount of the 2′,4′-di-*O*-acetyl byproduct due to the undesired acetyl-migration occurred during the column chromatographic purification. Condensation of acceptor **12** with donor **13**^20^ in the presence of NIS and trimethylsilyl trifluoromethanesulfonate (TMSOTf) resulted in an inseparable 1:1 mixture of the α- and *β*-linked trisaccharides **14** in a moderate yield (Fig. 3). The complete lack of stereoselectivity of the glycosylation was surprising because analogous reactions of **13** with either an L-idose^20^ or an L-iduronate^25^ acceptor proceeded with exclusive *α*-selectivity. After an oxidative cleavage of the temporary NAP-protecting group of **14** by DDQ, the desired trisaccharide acceptor **15** was isolated successfully, albeit with low yield.

**Figure 4:**
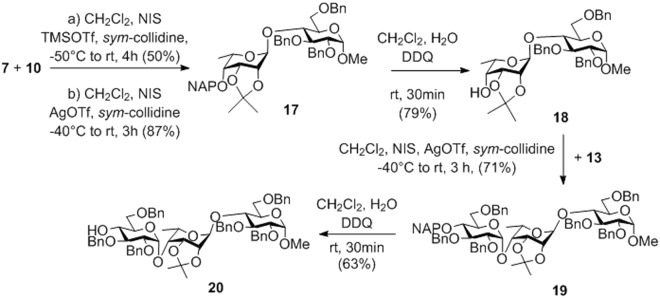
Synthesis of the **FGH** trisaccharide acceptor **20** by using the 2,3-*O*-isopropylidenated **7** as an *α*-selective donor.

In order to avoid side reactions and increase the yield, the assembly of the **FGH** trisaccharide was attempted by using the isopropylidenated 6-deoxy-L-talopyranoside derivative **7** as the donor in the first glycosylation step (Fig. 4). To our delight, the glycosylation with donor **7**, equipped with a non-participating group at C2, proceeded with exclusive 1,2-trans-selectivity providing the desired *α*-linked disaccharide **17** as the only product. Although the yield of **17** was moderate upon NIS-TMSOTf promotion, it was significantly increased by changing the Lewis acid in the promoter system to silver triflate (AgOTf). The role of the hindered base *sym*-collidine in the coupling reactions was to protect the acid-labile isopropylidene group against the acidic conditions of glycosylation. The 4′-OH of **17** was freed by oxidative cleavage of the NAP group using DDQ and the obtained disaccharide acceptor **18** was glycosylated with donor **13** in the presence of a NIS-AgOTf promoter system. Pleasingly, the condensation reaction occurred with complete *α*-selectivity affording the desired **FGH** trisaccharide **19** in 60% yield. Finally, removal of the NAP-ether from the terminal glucose unit gave acceptor **20** in 63% yield.

**Figure 5:**
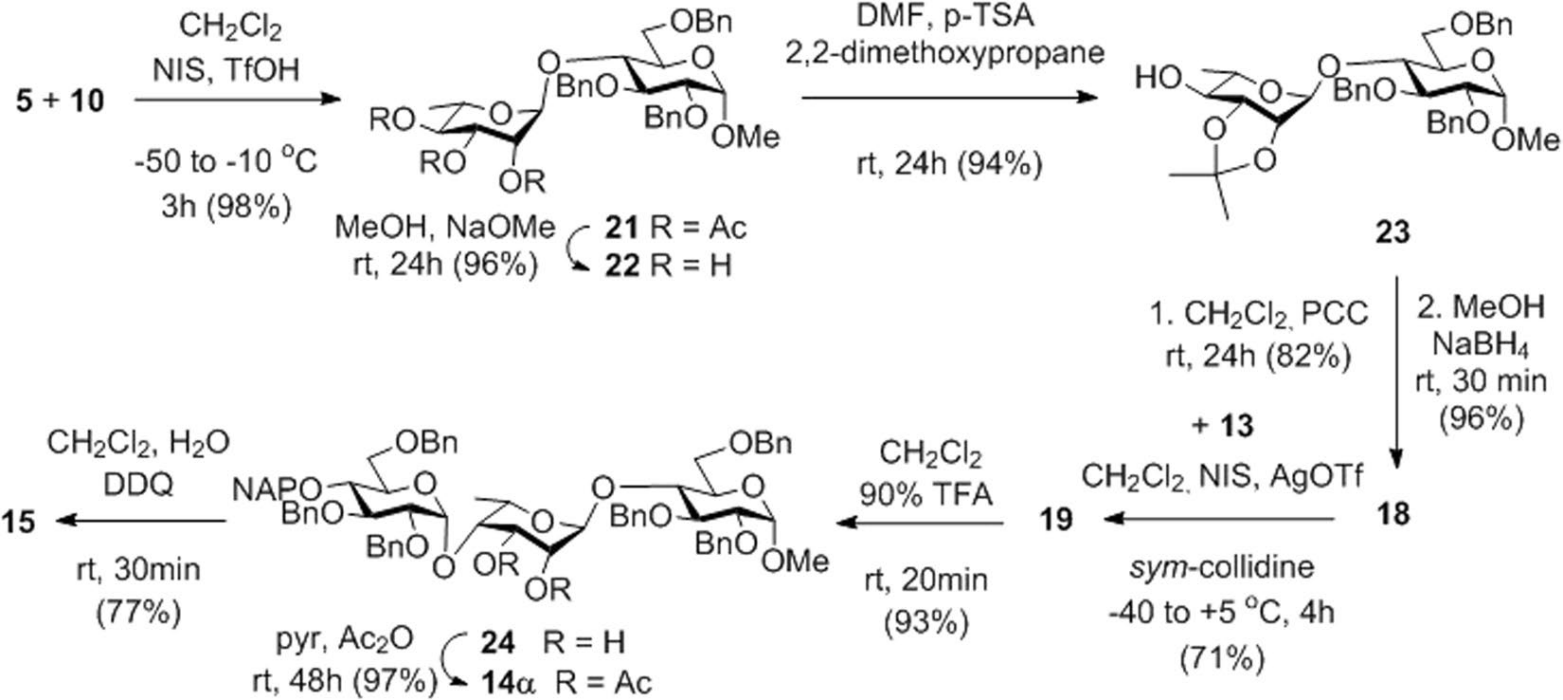
Improved synthesis of **FGH** acceptor **15**.

Although most difficulties of the first synthetic route to the acceptor **FGH** was overcame by changing the ester protected 6-deoxy-L-talopyranosyl building block **9** for the 2,3-acetal-protected donor **7** in the second route, we were not satisfied with the overall yield of this latter procedure. Therefore, we tested a third route for the preparation of trisaccharide **FGH** in which the phenylthio-*α*-L-rhamnopyranoside derivative **5** was used as the talose-precursor building block and the C4 epimerization of this unit was carried out at the disaccharide level (Fig. 5). Condensation of the L-rhamnose donor **5** and acceptor **10** led to the exclusive formation of the *α*-linked disaccharide **21** in 98% yield. After Zemplén deacetylation and a subsequent isopropylidenation, the two-step C4 epimerization, involving oxidation with pyridinium chlorochromate (PCC) followed by stereoselective reduction using NaBH_4_, proceeded with high efficacy affording the 6-deoxy-L-talose-containing disaccharide **18** in an excellent 71% overall yield from **21** via **22** and **23**. Disaccharide **18** was glycosylated with **13**, as described in the previous route. The 2′,3′-*O*-isopropylidene acetal moiety of **19** was changed to ester groups by acidic hydrolysis of the acetal group followed by acetylation of the obtained **24** to give **14** in 90% yield over the two steps. Oxidative removal of the 4″-*O*-NAP ether provided the desired **FGH** acceptor **15** in 77% yield.

**Figure 6:**
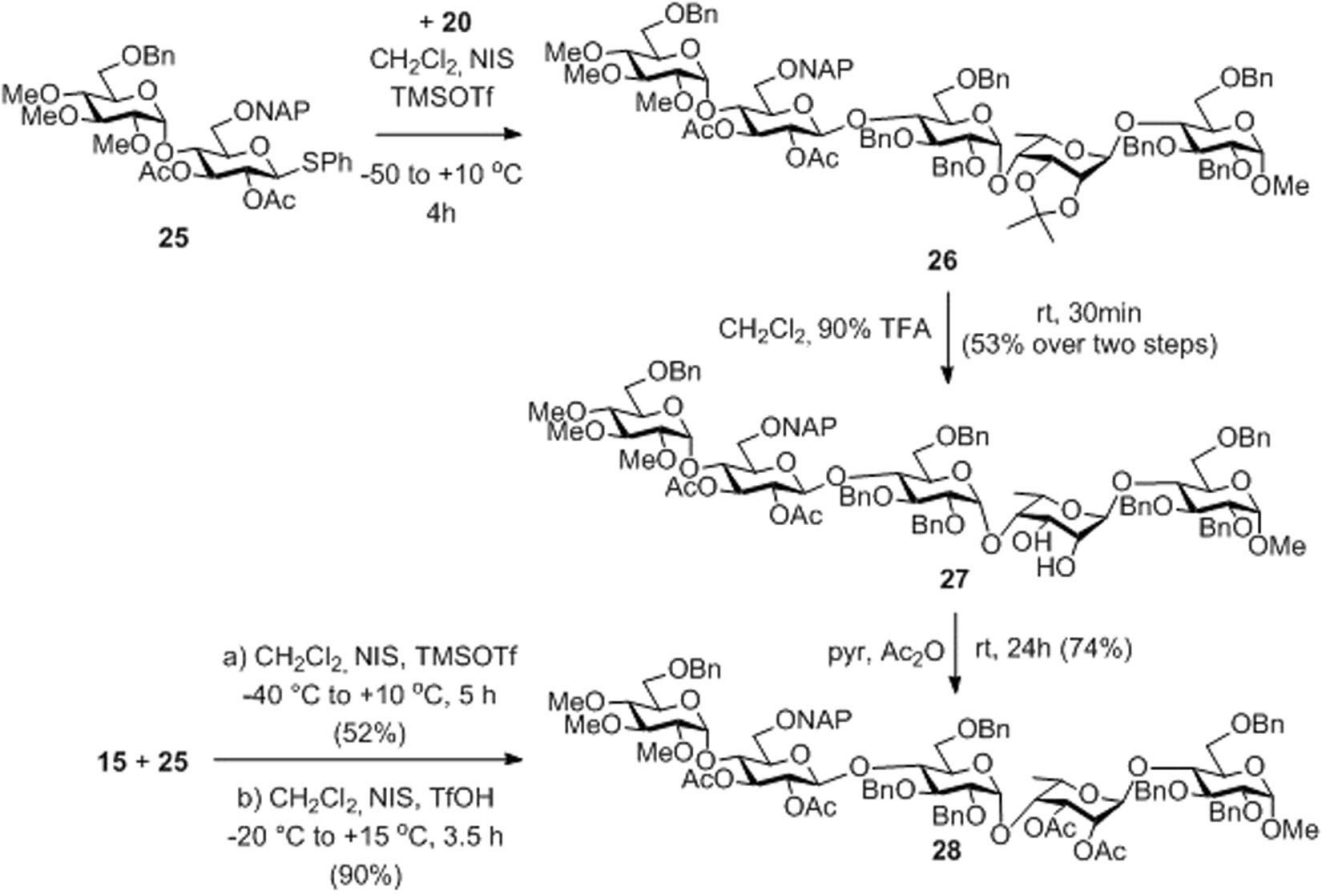
The [2 + 3] block syntheses by using trisaccharides **20** and **15** as the acceptors.

The assembly of the targeted pentasaccharides was carried out by coupling of the trisaccharide acceptors **15** and **20** with the non-glucuronide type **DE** disaccharide donor **25**^46^ (Fig. 6). According to our previously established strategy, we planned the oxidation of the glucose precursor **E** into D-glucuronic acid at the pentasaccharide level^20,24,41^. Condensation reaction of the isopropylidene-containing trisaccharide acceptor **20** and donor **25** upon NIS-TMSOTf activation provided the needed pentasaccharide **26** together with its diol derivative **27** formed by partial loss of the acetal group under the acidic conditions of the coupling. The products were unified by cleavage of the isopropylidene group of **26** to give diol **27** which was then acetylated to obtain the fully protected pentasaccharide **28** in 74% yield. The advantage of this protecting group pattern was that all hydroxyls to be methylated or freed in the final products were masked with the same acetate ester groups while the hydroxyls to be sulphated were protected in form of benzyl esters. Condensation of the 2′,3′-di-*O*-acetylated trisaccharide acceptor **15** and donor **25** provided a direct access to pentasaccharide **28**. While the coupling showed only moderate efficacy upon NIS-TMSOTf promotion, changing the promoter system to NIS-TfOH compound **28** was formed in an excellent 90% isolated yield.

**Figure 7:**
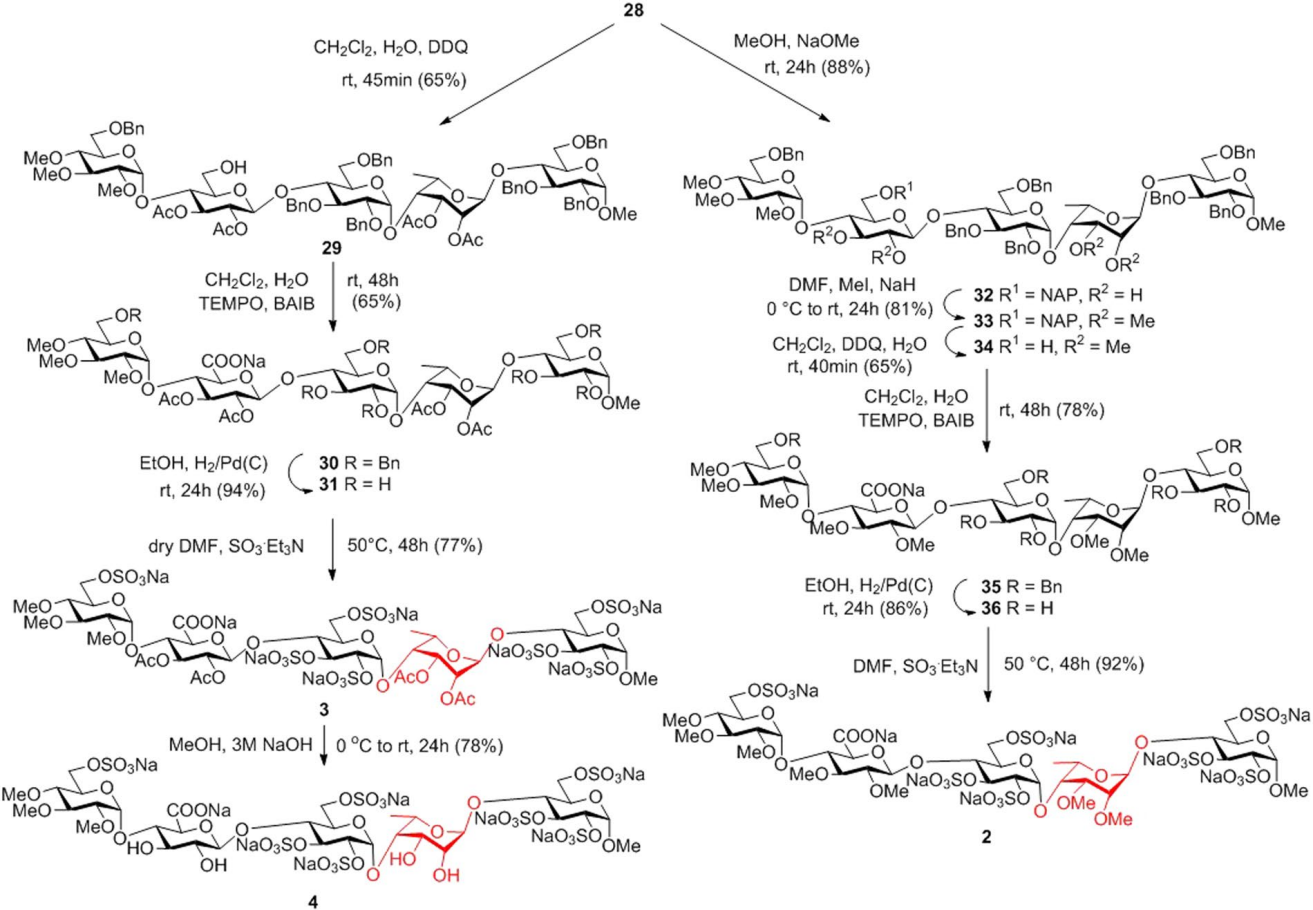
Synthesis of the idraparinux-analogue pentasaccharides **2**–**4** with various substitution pattern at units **E** and **G**.

Towards synthesis of the final products **3** and **4**, the first transformation at the pentasaccharide level was the removal of the temporary (2-naphthyl)methyl group from unit **E** followed by (2,2,6,6-tetramethylpiperidin-1-yl)oxyl (TEMPO) and [bis(acetoxy)iodo]benzene (BAIB) mediated oxidation^47,48^ of the freed primary hydroxyl group of **29** to produce the glucuronate-containing pentasaccharide **30** in form of a sodium salt (Fig. 7). Then, catalytic hydrogenolysis gave the debenzylated **31** in 94% yield, sulphation of which using excess SO_3_/Et_3_N complex in DMF afforded, after treatment with Dowex Na^+^ ion-exchange resin, the partially acetylated idraparinux-analogue final product **3** as an octasodium salt in 77% yield. The cleavage of the acetyls with the use of 3 M aqueous NaOH solution resulted in **4**, another idraparinux-analogue derivative containing free hydroxyl groups at units **E** and **G**.

Transformation of compound **28** into the fully *O*-methylated and *O*-sulphated final product required a different pathway. It is known that uronic acid residues are prone to suffer *β*-elimination in the basic conditions of the etherification^46,49^. Therefore, prior to the oxidative formation of the glucuronide residue, compound **28** was deacetylated under Zemplén conditions and the obtained **32** was methylated in the presence of methyl iodide and sodium hydride. After the efficient methyl etherification, compound **33** was converted into the glucoronate derivative **35** in high yield by NAP-deprotection followed by TEMPO-BAIB oxidation of the freed hydroxyl at unit **E**. Finally, the hydroxyl groups to be sulphated were debenzylated by catalytic hydrogenolysis to give **36** in 86% yield. Subsequently, simultaneous *O*-sulphation of the seven freed hydroxyls was achieved with high efficacy using excess SO_3_/Et_3_N to give, after treatment with Dowex Na^+^ ion exchange resin, the target compound **2** in 91% yield.

The structure of all synthesized pentasaccharide derivatives was corroborated by ^1^H and ^13^C NMR spectroscopy. The unambiguous assignment of NMR resonances was achieved by combined use of 1D and 2D homo- and heteronuclear NMR spectroscopic methods, including COSY, TOCSY, ROESY, HSQC and HMBC experiments.

**Figure 8:**
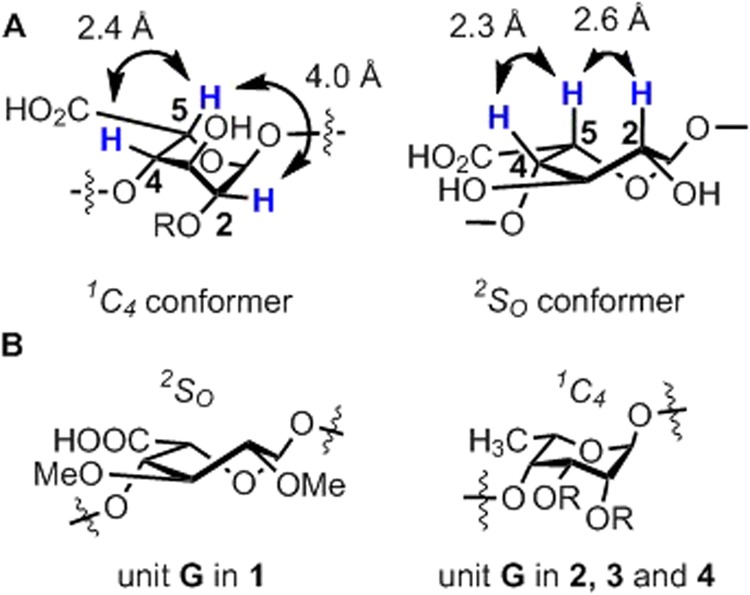
(**A**) Dominant conformational forms of the IdoA residue present in heparin-related oligosaccharides and calculated distances between H2 and H5, as well as H4 and H5^14^; R = H or SO_3_H. (**B**) Predominant conformation of unit **G** in pentasaccharide **1** and pentasaccharides **2**–**4**.

In heparin-related oligosaccharides the L-iduronic acid and L-iduronic acid 2-sulphate residues exist in a dynamic equilibrium of the ^1^*C*_4_, ^2^*S*_*O*_ and ^4^*C*_1_ conformers where the chair ^1^*C*_4_ and the skew-boat ^2^*S*_*O*_ are the predominant conformational forms (Fig. 8A)^14,15,36^. Each conformer has a unique set of three bond proton-proton coupling constants ^3^*J*(H,H) which have recently been calculated by Liu and co-workers^14^ using Amber 14 with GLYCAM06 parameter^50^ (Table 1, calculated values). To investigate the conformational behavior of the functionally most critical unit **G** and to assess the relative population of the skew-boat (^2^*S*_*O*_) conformer, known to be essential for binding to antithrombin, the structurally relevant ^3^*J*(H,H) couplings were measured for the novel *talo*-derivatives (**2**–**4**) and compared to the calculated values as well as to the corresponding data of idraparinux **1**^40^, which was used as reference compound in the present study (Table 1).

**Table 1:** ^3^*J*(H,H) couplings [Hz] and ratio of pertinent NOE intensities calculated for the L-iduronic-acid residue in its locked conformations together with the relevant ^3^*J*(H,H) [Hz] and NOE values measured for residue **G** in **1**–**4**.

*Calculated values* Locked conformation^[*a*,*b*]^				*Measured values* for unite G			
	^4^C_1_	^1^ *C*_4_	^2^S_*O*_	1	2	3	4
^3^*J*(1,2)	7.85	1.35	5.55	2.6	<1.0	1.6	1.6
^3^*J*(2,3)	8.03	2.04	8.48	5.5	3.0	3.6	3.2
^3^*J*(3,4)	8.50	2.19	4.82	2.2	3.0	3.7	not determined due to signal overlap
Ratio of H2-H5 and H4-H5 NOE signals	0.03	0.05	0.53	0.5	0.1	0.1	<0.2

^[*a*]^Calculated ^3^*J*(H,H) values (Hz) for locked ^2^*S*_*O*_, ^1^*C*_4_ and ^4^*C*_1_ conformers^14^.

^[*b*]^Calculated ratio of NOE H2-H5 and H4-H5 signal intensities for pure ^2^*S*_*O*_, ^1^*C*_4_ and ^4^*C*_1_ conformers^14^.

The distributions of conformers were also studied by ^1^H-^1^H NOE analysis. The NOE cross peak intensities are uniquely sensitive to detect the presence of the skew-boat conformer due to the significant difference in the atomic distance between H2 and H5 in the ^2^*S*_*O*_ conformational form (2.6 Å), compared with the ^1^*C*_4_ (4.0 Å) (Fig. 8A). The shorter H2-H5 interproton distance in the ^2^*S*_*O*_ conformer offers a stronger NOE intensity. Liu and co-workers demonstrated^14^ that together with the analysis of ^3^*J*(H,H) couplings, the ratio of H2-H5 and H4-H5 NOE intensities can be used to estimate the conformer population of the L-iduronic acid residue.

As shown in Table 1, the ^3^*J*(1,2) and ^3^*J*(2,3) values for each *talo*-analogue are considerably smaller than the ones measured in idraparinux, thus suggesting that the relative population of the biologically favorable skew boat (^2^*S*_*O*_) conformer is reduced in the *talo*-derivatives and the conformational equilibrium is shifted towards the functionally less preferred ^1^*C*_4_ chair conformer (Fig. 8B). The analysis of H2-H5 and H4-H5 NOE intensity ratios (Table 1) also confirms this shift of the conformational equilibrium. The small values of NOE ratios, ranging between 0.1 and 0.2, suggest that a major ^1^*C*_4_ chair conformer with longer atomic distance between H2 and H5, correspondingly with weaker H2-H5 NOE crosspeak, indeed exists in D_2_O solution. (The interproton distances within unit **G** estimated from the volume integrals of ROESY cross peaks are summarized in Table S2).

Finally, the inhibitory activities of pentasaccharides **2**–**4** towards blood-coagulation proteinase Xa were determined by using Berichrom heparin assay (Table S1). Unfortunately, almost complete loss of the factor Xa-inhibitory activity was observed.

## Discussion

Heparin and heparinoid anticoagulants exert their anticoagulant activity by binding and activation of antithrombin (AT) which is an endogenous inhibitor of serine proteases in the coagulation cascade^51^. It has been known that plasticity of the L-iduronic acid unit of heparin, an easy shift from the equilibrium state of the preferred ^1^C_4_ and ^2^S_*O*_ conformations to the ^2^S_*O*_ skew-boat form, is crucial for the stabilization of the activated conformation of AT^36,52^. A detailed knowledge has been accumulated on how the sulphation pattern of neighbouring sugar residues and the sulphation of iduronic acid itself influence the conformational preference of this pyranosyl unit^14,15,37,53,54^. However, the precise structural requirements of the conformational plasticity is not known and it has not been studied whether other L-sugars, incorporated in a heparin structure, can adopt the bioactive ^2^S_*O*_ conformation. Considering the complicated synthesis of the iduronate building block, the possible substitution of this unit by a more easily available monosaccharide, possessing the required conformational plasticity, would be of great importance.

In this work, we replaced the iduronate residue of the synthetic anticoagulant idraparinux by a 6-deoxy-L-talopyranose, which is the most easily available L-hexose epimer of L-idose, and studied the conformational behavior and biological activity of the obtained pentasaccharides. Beside the closest, fully *O*-sulphated and fully *O*-methylated analogue of idraparinux, a partially methylated and a methylated/acetylated derivative was also synthesized. The assembly of pentasaccharide skeleton was accomplished by coupling a 6-deoxy-L-talopyranoside-containing trisaccharide acceptor to a non-glucuronide disaccharide donor and the glucose precursor was oxidized to the required glucuronide at the pentasaccharide level. The key building block, the 6-deoxy-L-talose-containing trisaccharide **FGH** was prepared through three different reaction paths, the shortest and most efficient route was when a phenylthio-*α*-L-rhamnopyranoside was used as the precursor building block and its conversion to the *talo*-configured unit **G** was performed at the disaccharide level.

While we successfully simplified and shortened the preparation of a heparinoid anticoagulant by replacing the synthetically most demanding unit, the biological activity was almost completely lost. The conformational analysis of pentasaccharides **2**–**4**, based on ^1^H NMR ROESY measurements, revealed that the critically important unit **G** predominantly populated the functionally less preferred ^1^*C*_4_ chair conformation. It was also shown that differences in the methylation pattern of the pentasaccharides had no effects on the conformer distribution of the *talo*-residue. The observed loss of the biological activities could be attributed to the lack of the essential carboxylic moiety of unit **G** as well as to the less abundance of the bioactive ^2^S_*O*_ conformer in the conformational equilibrium. We assume, that conversion of the 6-deoxy-L-talose residue to L-taluronic acid using established methods^38,47^ and introduction of a 2-*O*-sulphate moiety, which is present in the natural AT-binding sequence, might push the conformational equilibrium of unit **G** toward the crucial ^2^*S*_*O*_ form.

It is very important to note that although literature data show correlation between the affinity of heparin oligosaccharides and the relative population of the ^2^*S*_*O*_ form of iduronic acid in solution^55^, recent results of Liu, Guerrini and co-workers^14,15^ revealed that predominant population of the ^2^*S*_*O*_ skew boat conformer of iduronic acid in free form is not a prerequisite for the activation of AT. They demonstrated that a synthetic heparin hexasaccharide, iduronate residue of which is displayed 73% of ^1^*C*_4_ conformer in solution, can efficiently activate antithrombin and the iduronic acid adopts a ^2^*S*_*O*_ conformation when bound to AT. These results indicate that although conformational analysis is very important, the biological test can not be avoided before making a final judgment on the anticoagulant activity of a compound. Further studies to find the *aurea mediocritas*, to simplify the structure and synthesis of heparin oligosaccharides to an extent that the compounds could keep the biological activity, are under way.

## Methods

Optical rotations were measured at room temperature on a Perkin-Elmer 241 automatic polarimeter. TLC analysis was performed on Kieselgel 60 F_254_ (Merck) silica-gel plates with visualization by immersing in a sulfuric-acid solution (5% in EtOH) followed by heating. Column chromatography was performed on silica gel 60 (Merck 0.063–0.200 mm) and Sephadex LH-20 (Sigma-Aldrich, bead size: 25–100 mm). Organic solutions were dried over MgSO_4_ and concentrated under vacuum. One- (1D) and two-dimensional (2D) ^1^H, ^13^C, COSY, ROESY and HSQC spectra were recorded on Bruker Avance II 400 (^1^H: 400 MHz; ^13^C: 100.28 MHz), Avance II 500 (^1^H: 500.13 MHz; ^13^C: 125.76 MHz) and Avance Neo 700 (^1^H: 700.25 MHz; ^13^C: 176.08 MHz) spectrometers at 25 °C. Chemical shifts are referenced to SiMe_4_ or sodium 3-(trimethylsilyl)-1-propanesulfonate (DSS, = 0.00 ppm for ^1^H nucleus) and to the solvent signals (CDCl_3_: *δ* = 77.00 ppm, (CD_3_OD: *δ* = 49.15 ppm for ^13^C nucleus). MALDI-TOF MS analyses of the compounds were carried out in the positive reflektron mode using a BIFLEX III mass spectrometer (Bruker, Germany) equipped with delayed-ion extraction. 2,5-Dihydroxybenzoic acid (DHB) was used as matrix and F_3_CCOONa as cationising agent in DMF. ESI-TOF MS spectra were recorded by a microTOF-Q type QqTOFMS mass spectrometer (Bruker) in the positive ion mode using MeOH as the solvent. Elemental analysis was performed on an Elementar Vario MicroCube (CHNS) instrument. The anti-factor Xa activity of **2**–**4** was determined *in vitro* by Berichrom® Heparin chromogenic assay on a Siemens BCS-XP automated coagulometer (Siemens, Marburg, Germany), using pooled normal human plasma (see Supporting Infromation). Pentasaccharides **2**–**4** were tested in at least 3 different concentrations (final concentration range of 250–1000 *μ*g/mL) using a pentasaccharide (fondaparinux/Arixtra) calibrator from Diagnostica Stago (Asnieres, France).

**Octa-sodium [methyl (2,3,4-tri-*O*-methyl-6-*O*-sulfonato-*α*-D-glucopyranosyl)-(1→4)-(2,3-di-*O*-methyl-*β*-D-glucopyranosyl-uronate)-(1→4)-(2,3,6-tri-*O*-sulfonato-*α*-D-glucopyranosyl)-(1→4)-(2,3-di-*O*-methyl-6-deoxy-*α*-L-talopyranosyl)-(1→4)-(2,3,6-tri-*O*-sulfonato-*α*-D-glucopyranoside)]** (**2**)

A solution of compound **36** (120 mg, 0.125 mmol) in dry DMF (7.0 mL) was treated with SO_3_/Et_3_N (792 mg, 4.371 mmol). After stirring for 48 h at 50 °C, the reaction mixture was neutralized with a saturated aqueous solution of NaHCO_3_ (1.836 g, 21.85 mmol) and the resulting mixture was concentrated. The crude product was treated with Dowex ion-exchange resin (Na^+^) and purified by column chromatography on Sephadex G-25 (H_2_O) to give compound **2** (192 mg, 92%) as a white foam. [*α*]_*D*_ + 55.0 (*c* 0.14, H_2_O); *R*_*f*_ 0.36 (7:6:1 CH_2_Cl_2_/MeOH/H_2_O); ^1^H NMR (400 MHz, D_2_O) *δ* = 5.71 (d, *J* = 3.2 Hz, 1H, H-1-F), 5.45 (d, *J* = 3.6 Hz, 1H, H-1-D), 5.15 (d, *J* = 2.7 Hz, 2H, H-1-H, H-1-G), 4.70–4.61 (m, 3H, H-1-E, H-3-F, H-5-G), 4.57 (t, *J* = 9.2 Hz, 1H, H-3-H), 4.41–4.29 (m, 5H, H-2-H, H-2-F, H-6a,b-F, H-6a-H), 4.27–4.22 (m, 2H, H-6b-H, H-6a-D), 4.18 (s, 1H, H-4-G), 4.11 (d, *J* = 10.9 Hz, 1H, H-6b-D), 4.04–4.00 (m, 3H, H-4-F, H-5-F, H-5-H), 3.92 (t, *J* = 9.4 Hz, 1H, H-4-H), 3.89–3.85 (m, 2H, H-4-E, H-5-D), 3.79 (s, 1H, H-3-G), 3.72 (d, *J* = 9.7 Hz, 1H, H-5-E), 3.62–3.45 (m, 3H, H-2-G, H-3-E, H-3-D), 3.34–3.29 (m, 2H, H-2-D, H-4-D), 3.25 (t, *J* = 8.0 Hz, 1H, H-2-E), 3.61, 3.60, 3.58, 3.56, 3.54, 3.48, 3.47, 3.46 (8 x s, 24H, 8 x OC*H*_3_), 1.31 (d, *J* = 6.5 Hz, 3H, C*H*_3_ talose) ppm; ^13^C NMR (100 MHz, D_2_O) *δ* = 175.9 (1C, COONa), 102.1 (1C, C-1-E), 98.0 (2C, C-1-H, C-1-G), 96.9 (1C, C-1-D), 96.2 (1C, C-1-F), 86.3 (1C, C-3-E), 83.8 (1C, C-2-E), 82.6 (1C, C-2-D), 81.2 (1C, C-3-D), 78.8 (1C, C-4-D), 78.2 (1C, C-3-G), 77.9 (1C, C-2-G), 77.6 (1C, C-5-E), 76.7 (1C, C-3-H), 76.2 (1C, C-2-F), 76.0 (1C, C-2-H), 75.8 (1C, C-3-F), 75.2 (1C, C-4-E), 73.9 (1C, C-4-F), 73.4 (1C, C-4-H), 71.2 (1C, C-4-G), 70.9 (1C, C-5-F), 69.9 (1C, C-5-H), 69.5 (1C, C-5-D), 68.6 (1C, C-5-G), 67.3, 66.9 (2C, C-6-F, C-6-H), 66.7 (1C, C-6-D), 61.2, 61.0, 60.8, 60.3, 59.8, 59.7, 57.6 (7C, 7 x O*C*H_3_), 56.3 (1C, C-1-H-O*C*H_3_), 17.1 (1C, *C*H_3_ talose) ppm; MS (ESI-TOF): m/z calcd for C_38_H_58_Na_5_O_47_S_7_, [M-3Na]^3−^ 534.990; found: 534.989 [M-3Na]^3−^; elemental analysis calcd (%) for C_38_H_58_Na_8_O_47_S_7_ (1673.924); C, 27.25; H, 3.49; S, 13.40; found: C, 27.37; H, 3.55; S, 13.48.

**Octa-sodium [methyl (2,3,4-tri-*O*-methyl-6-*O*-sulfonato-*α*-D-glucopyranosyl)-(1→4)-(2,3-di-*O*-acetyl-*β*-D-glucopyranosyl-uronate)-(1→4)-(2,3,6-tri-*O*-sulfonato-*α*-D-glucopyranosyl)-(1→4)-(2,3-di-*O*-acetyl-6-deoxy-*α*-L-talopyranosyl)-(1→4)-(2,3,6-tri-*O*-sulfonato-*α*-D-glucopyranoside)]** (**3**)

A solution of compound **31** (78 mg, 0.073 mmol) in dry DMF (4.0 mL) was treated with SO_3_/Et_3_N (461 mg, 2.545 mmol). After stirring for 48 h at 50 °C, the reaction mixture was neutralized with a saturated aqueous solution of NaHCO_3_ (1.069 g, 12.72 mmol). The resulting mixture was concentrated. The crude product was treated with Dowex ion-exchange resin (Na^+^) and purified by column chromatography on Sephadex G-25 (H_2_O) to give compound **3** (100 mg, 77%) as a white foam. [*α*]_*D*_ + 14.0 (*c* 0.10, H_2_O); *R*_*f*_ 0.40 (7:6:1 CH_2_Cl_2_/MeOH/H_2_O); ^1^H NMR (400 MHz, D_2_O) *δ* = 5.35 (d, *J* = 2.9 Hz, 1H, H-1-F), 5.30 (t, *J* = 9.2 Hz, 1H, H-3-E), 5.27 (d, *J* = 1.8 Hz, 1H, H-3-G), 5.21 (d, *J* = 3.8 Hz, 1H, H-1-D), 5.16 (d, *J* = 3.5 Hz, 2H, H-1-H, H-2-G), 5.04 (s, 1H, H-1-G), 4.97 (d, *J* = 8.0 Hz, 1H, H-1-E), 4.91 (d, *J* = 8.9 Hz, 1H, H-2-E), 4.82–4.72 (m, 2H, H-3-F, H-5-G), 4.59 (t, *J* = 9.5 Hz, 1H, H-3-H), 4.45 (dd, *J* = 2.2 Hz, *J* = 11.3 Hz, 1H, H-6a-F), 4.39–4.34 (m, 2H, H-2-H, H-6b-F), 4.31–4.27 (m, 3H, H-2-F, H-6a-D, H-6a-H), 4.24–4.22 (m, 1H, H-5-F), 4.18–4.13 (m, 4H, H-4-E, H-4-G, H-6b-H, H-6b-D), 4.05–4.01 (m, 2H, H-4-F, H-5-H), 3.96–3.88 (m, 3H, H-4-H, H-5-E, H-5-D), 3.63 (s, 3H, C-3-D-OC*H*_3_), 3.58 (s, 3H, C-4-D-OC*H*_3_), 3.54 (t, *J* = 9.6 Hz, 1H, H-3-D), 3.48 (s, 3H, C-2-D-OCH3), 3.46 (s, 3H, C-1-H-OC*H*_3_), 3.32 (t, *J* = 9.8 Hz, 1H, H-4-D), 3.29 (dd, *J* = 3.9 Hz, *J* = 9.9 Hz, 1H, H-2-D), 2.17, 2.14 (2 x s, 12H, 4 x C*H*_3_ OAc), 1.38 (d, *J* = 6.6 Hz, 3H, C*H*_3_ talose) ppm; ^13^C NMR (100 MHz, D_2_O) *δ* = 175.0, 174.6, 174.5, 173.9, 173.8 (5C, COONa, 4 x Cq OAc), 99.7 (1C, C-1-E), 98.5 (1C, C-1-G), 98.4 (1C, C-1-F), 98.3 (1C, C-1-D), 98.2 (1C, C-1-H), 83.4 (1C, C-3-D), 81.0 (1C, C-2-D), 79.0 (1C, C-4-D), 77.8 (1C, C-5-E), 77.2 (1C, C-4-E), 76.9 (1C, C-2-F), 76.7 (1C, C-3-F), 76.5 (1C, C-3-H), 76.3 (1C, C-3-F), 76.2 (1C, C-2-H), 76.0 (1C, C-4-G), 74.6 (1C, C-4-F), 73.6 (1C, C-2-E), 73.2 (1C, C-4-H), 71.1 (1C, C-5-F), 69.9 (2C, C-5-H, C-5D), 68.4 (1C, C-2-G), 68.3 (1C, C-3-G), 68.2 (1C, C-5-G), 66.8 (2C, C-6-D, C-6-H), 66.7 (1C, C-6-F), 61.1 (1C, C-3-D-O*C*H_3_), 60.9 (1C, C-4-D-O*C*H_3_), 60.4 (1C, C-2-D-O*C*H_3_), 56.3 (1C, C-1-H-O*C*H_3_), 21.7, 21.6, 21.4, 21.2 (4C, 4 × *C*H_3_ OAc), 16.8 (1C, *C*H_3_ talose) ppm; MS (ESI-TOF): m/z calcd for C_42_H_58_Na_4_O_51_S_7_, [M-4Na]^4−^ 423.490; found: 423.491 [M-4Na]^4−^; elemental analysis calcd (%) for C_42_H_58_Na_8_O_51_S_7_ (1785.92); C, 28.23; H, 3.27; S, 12.56; found: C, 28.37; H, 3.34; S, 12.63.

**Octa-sodium [methyl (2,3,4-tri-*O*-methyl-6-*O*-sulfonato-*α*-D-glucopyranosyl)-(1→4)-(*β*-D-glucopyranosyl-uronate)-(1→4)-(2,3,6-tri-*O*-sulfonato-*α*-D-glucopyranosyl)-(1→4)-(6-deoxy-*α*-L-talopyranosyl)-(1→4)-(2,3,6-tri-*O*-sulfonato-*α*-D-glucopyranoside)]** (**4**)

To a solution of compound **3** (50 mg, 0.028 mmol) in MeOH (1.2 mL) a solution of NaOH (3 M, 600 *μ*L) was added at 0 °C. When complete conversion of the starting material into a main spot had been observed by TLC analysis (24 h at room temperature), the mixture was neutralized with AcOH and all volatiles were evaporated. The crude product was purified by column chromatography on Sephadex G-25 (H_2_O) to give compound **4** (35 mg, 78%) as a white foam. [*α*]_*D*_ + 62.5 (*c* 0.10, H_2_O); *R*_*f*_ 0.69 (7:3 MeCN/H_2_O); ^1^H NMR (400 MHz, D_2_O) *δ* = 5.69 (d, *J* = 3.9 Hz, 1H, H-1-F), 5.64 (d, *J* = 3.7 Hz, 1H, H-1-D), 5.15 (d, *J* = 3.5 Hz, 1H, H-1-H), 5.10 (d, *J* = 0.5 Hz, 1H, H-1-G), 4.71–4.66 (m, 2H, H-3-F, H-5-G), 4.64 (d, *J* = 7.9 Hz, 1H, H-1-E), 4.58 (t, *J* = 9.3 Hz, 1H, H-3-H), 4.47 (dd, *J* = 3.9 Hz, *J* = 9.4 Hz, 1H, H-2-F), 4.42–4.26 (m, 5H, H-2-H, H-6a-F, H-6a-D, H-6a,b-H), 4.23–4–21 (m, 1H, H-6b-F), 4.13 (d, *J* = 9.8 Hz, 1H, H-6b-D), 4.06–4.00 (m, 5H, H-3-G, H-4-G, H-4-F, H-5-H, H-5-F), 3.95–3.85 (m, 4H, H-2-G, H-4-E, H-4-H, H-5-D), 3.79 (d, *J* = 10.0 Hz, 1H, H-5-E), 3.73 (t, *J* = 8.8 Hz, 1H, H-3-E), 3.60 (s, 3H, C-3-D-O*C*H_3_), 3.57 (s, 3H, C-4-D-O*C*H_3_), 3.54–3.51 (m, 1H, H-3-D), 3.52 (s, 3H, C-2-D-O*C*H_3_), 3.46 (s, 3H, C-1-H-O*C*H_3_), 3.42 (dd, *J* = 8.2 Hz, *J* = 9.1 Hz, 1H, H-2-E), 3.37–3.31 (m, 2H, H-2-D, H-4-D), 1.32 (d, *J* = 6.5 Hz, 3H, C*H*_3_ talose) ppm; ^13^C NMR (100 MHz, D_2_O) *δ* = 102.1 (1C, C-1-E), 101.2 (1C, C-1-G), 98.1 (1C, C-1-H), 97.9 (1C, C-1-F), 96.7 (1C, C-1-D), 82.3 (1C, C-3-D), 81.2 (1C, C-2-D), 78.9 (2C, C-4-D, C-4-F), 78.0 (1C, C-5-E), 77.5 (1C, C-3-E), 77.1 (1C, C-4-E), 76.5 (2C, C-3-H, C-3-F), 76.1 (1C, C-2-H), 75.6 (1C, C-2-F), 74.4 (1C, C-2-E), 73.8 (1C, C-4-H), 73.6 (1C, C-3-G), 71.0 (1C, C-4-G), 70.5 (1C, C-2-G), 70.0 (1C, C-5-H), 69.6 (1C, C-5D), 68.3 (1C, C-5-G), 67.7 (1C, C-5-F), 67.2 (1C, C-6-H), 66.9 (2C, C-6-D, C-6-F), 61.0 (1C, C-3-D-O*C*H_3_), 60.8 (1C, C-4-D-O*C*H_3_), 59.0 (1C, C-2-D-O*C*H_3_), 56.3 (1C, C-1-H-O*C*H_3_), 17.7 (1C, *C*H_3_ talose) ppm; MS (ESI-TOF): m/z calcd for C_34_H_50_Na_3_O_47_S_7_, [M-4Na]^4−^ 300.586; found: 300.585 [M-4Na]^4−^; elemental analysis calcd (%) for C_34_H_50_Na_8_O_47_S_7_ (1617.87); C, 25.22; H, 3.11; S, 13.86; found: C, 25.31; H, 3.18; S, 13.94.

## Electronic supplementary material

Supplementary Information
