# Supplementary material for: Replacement of the L-iduronic acid unit of the anticoagulant pentasaccharide idraparinux by a 6-deoxy-L-talopyranose – Synthesis and conformational analysis
Source: Sci Rep. 2018 Sep 13;8:13736. doi: 10.1038/s41598-018-31854-z (PMC6137110; doi:10.1038/s41598-018-31854-z)
Supplement: Supplementary file 1 — Supplementary Information [file 41598_2018_31854_MOESM1_ESM.pdf]

# Supporting Information

# Replacement of the L-iduronic acid unit of the anticoagulant pentasaccharide idraparinux by a 6-deoxy-L-talopyranose – Synthesis and conformational analysis

**Fruzsina Demeter[a], Tamás Gyöngyösi[b], Zsuzsanna Bereczky[c], Katalin E. Kövér*[b], Mihály Herczeg*[a] and Anikó Borbás* [a]**

[a]Department of Pharmaceutical Chemistry, University of Debrecen, Egyetem tér 1, H-4032, Debrecen, Hungary

[b]Department of Inorganic and Analytical Chemistry, University of Debrecen, P. O. Box 400, H-4002, Debrecen, Hungary,

[c]Division of Clinical Laboratory Science, Department of Laboratory Medicine, Faculty of Medicine, University of Debrecen, Nagyerdei krt. 98, H-4032 Debrecen, Hungary

*e-mail: herczeg.mihaly@science.unideb.hu*

*kover@science.unideb.hu*

*borbas.aniko@pharm.unideb.hu*

## Table of Contents

General Information ........ S2

Methods and characterization of compounds ........ S2

Measurements of factor Xa inhibitory activity (Table S1) ........ S22

References ........ S24

$^{1}$H and $^{13}$C NMR spectra of the new compounds ........ S25

$^{1}$H-$^{1}$H ROESY spectra of the target compounds ........ S102

$^{1}$H-$^{1}$H distances based on ROESY experiments (Table S2) ........ S108

## General Information

Optical rotations were measured at room temperature on a Perkin–Elmer 241 automatic polarimeter. TLC analysis was performed on Kieselgel 60 $F_{254}$ (Merck) silica–gel plates with visualization by immersing in a sulfuric-acid solution (5% in EtOH) followed by heating. Column chromatography was performed on silica gel 60 (Merck 0.063–0.200 mm) and Sephadex LH-20 (Sigma–Aldrich, bead size: 25–100 mm). Organic solutions were dried over $MgSO_4$ and concentrated under vacuum. One- (1D) and two-dimensional (2D) $^{1}H$, $^{13}C$, COSY, ROESY and HSQC spectra were recorded on Bruker Avance II 400 ($^{1}H$: 400 MHz; $^{13}C$: 100.28 MHz), Avance II 500 ($^{1}H$: 500.13 MHz; $^{13}C$:125.76 MHz) and Avance Neo 700 ($^{1}H$: 700.25 MHz; $^{13}C$:176.08 MHz) spectrometers at 25 °C. Chemical shifts are referenced to $SiMe_4$ or sodium 3-(trimethylsilyl)-1-propanesulfonate (DSS, $\delta$ = 0.00 ppm for $^{1}H$ nucleus) and to the solvent signals ($CDCl_3$: $\delta$ = 77.00 ppm, $CD_3OD$: $\delta$ = 49.15 ppm for $^{13}C$ nucleus). MALDI-TOF MS analyses of the compounds were carried out in the positive reflektron mode using a BIFLEX III mass spectrometer (Bruker, Germany) equipped with delayed-ion extraction. 2,5-Dihydroxybenzoic acid (DHB) was used as matrix and $F_3CCOONa$ as cationising agent in DMF. ESI-TOF MS spectra were recorded by a microTOF-Q type QqTOFMS mass spectrometer (Bruker) in the positive ion mode using MeOH as the solvent. Elemental analysis was performed on an Elementar Vario MicroCube (CHNS) instrument.

## Methods and characterization of compounds

### Phenyl 2,3-*O*-isopropylidene-4-*O*-(2-naphtyl)methyl-6-deoxy-1-thio-α-L-talopyranoside (7)

To a solution of compound **6**[1] (4.58 g, 15.47 mmol) in dry DMF (100 mL) was slowly added NaH (775 mg, 19.33 mmol, 1.25 equiv.) at 0 °C. After stirring for 30 min at 0 °C, NAPBr (4.25 g, 19.33 mmol, 1.25 equiv.) was added. When complete conversion of the starting material into a main spot had been observed by TLC analysis (24 h at room temperature), $CH_3OH$ (20 mL) was added. The reaction mixture was stirred for 5 min and the solvents were evaporated. The residue was dissolved in $CH_2Cl_2$ and washed with $H_2O$ (2 x 75 mL), the organic layer was filtered, dried and evaporated. The crude product was purified by column chromatography (8:2 *n*-hexane/EtOAc) to give compound **7** (6.27 g, 93%) as white crystals. $[\alpha]_D$ −108.4 (*c* 0.16, $CHCl_3$); M.p.: 95-98 °C; $R_f$ 0.46 (65:35 *n*-hexane/EtOAc); $^{1}H$ NMR (400 MHz, $CDCl_3$) $\delta$ = 7.82-7.22 (m, 12H, arom), 5.44 (d, *J* = 5.1 Hz, 1H, H-1), 4.87 (dd, *J* = 92.9 Hz, *J* = 12.2 Hz, 2H, NAPC$H_2$), 4.42 (dd, *J* = 6.0 Hz, *J* = 4.6 Hz, 1H), 4.16-4.10 (m, 2H),

3.71 (t, *J* = 4.1 Hz, 1H), 1.57 (s, 3H, C*H*$_3$ isopropylidene), 1.39 (s, 3H, C*H*$_3$ isopropylidene), 1.25 (d, *J* = 6.7 Hz, 3H, C*H*$_3$ talose) ppm; $^{13}$C NMR (100 MHz, CDCl$_3$) $\delta$ = 135.4-133.2 (4C, C$_q$ arom), 131.5-126.1 (12C, arom), 110.4 (1C, C$_q$ isopropylidene), 82.3 (1C, C-1), 74.8, 74.4, 73.4, 68.6 (4C, C-2, C-3, C-4, C-5), 74.1 (1C, NAP*C*H$_2$), 27.0, 25.7 (2C, 2 x *C*H$_3$ isopropylidene), 15.9 (1C, *C*H$_3$ talose) ppm; elemental analysis calcd (%) for $C_{26}H_{28}O_4S$ (436.17): C, 71.53; H, 6.47; S, 7.34; found: C, 71.58; H, 6.51; S, 7.39.

**Phenyl 4-*O*-(2-naphtyl)methyl-6-deoxy-1-thio-α-L-talopyranoside (8)**

To a solution of compound **7** (250 mg, 0.573 mmol) in $CH_2Cl_2$ (5.0 mL) 90% TFA (TFA: $H_2O$ 9:1, 2 mL) was added and the mixture was stirred for 1.5 h at rt. After the reaction had been completed, the mixture was concentrated. The residue was purified by column chromatography on silica gel (7:3 *n*-hexane/EtOAc) to give compound **8** (172 mg, 76%) as a colourless syrup. $[\alpha]_D$ −165.6 (*c* 0.11, CHCl$_3$); $R_f$ 0.31 (7:3 *n*-hexane/EtOAc); $^1$H NMR (400 MHz, CDCl$_3$) $\delta$ = 7.81-7.20 (m, 12H, arom), 5.56 (s, 1H, H-1), 5.31 (s, 2H, 2 x O*H*), 4.84 (dd, *J* = 45.0 Hz, *J* = 11.1 Hz, 2H, 2 x NAPC*H*$_2$), 4.32 (q, *J* = 6.4 Hz, 1H), 4.02 (s, 1H), 3.88 (s, 1H), 3.68 (s, 1H), 1.22 (d, *J* = 6.5 Hz, 3H, C*H*$_3$ talose) ppm; $^{13}$C NMR (100 MHz, CDCl$_3$) $\delta$ = 134.6-133.3 (4C, C$_q$ arom), 131.5-126.1 (12C, arom), 89.1 (1C, C-1), 81.0, 72.6, 67.6 (4C, C-2, C-3, C-4, C-5), 76.9 (1C, NAP*C*H$_2$), 16.9 (1C, *C*H$_3$ talose) ppm; MS (MALDI-TOF): *m*/*z* calcd for $C_{23}H_{24}NaO_4S$, $[M+Na]^+$ 419.13; found: 419.13; elemental analysis calcd (%) for $C_{23}H_{24}O_4S$ (396.14); C, 69.67; H, 6.10; S, 8.09; found: C, 69.71; H, 6.13; S, 8.14.

**Phenyl 2,3-di-*O*-acetyl-4-*O*-(2-naphtyl)methyl-6-deoxy-1-thio-α-L-talopyranoside (9)**

To a solution of compound **8** (4.704 g, 11.86 mmol) in dry pyridine (30 mL) $Ac_2O$ (10 mL) was added and the mixture was stirred for 24 h at room temperature. After the reaction had been completed, the mixture was concentrated. The residue was purified by column chromatography on silica gel (7:3 *n*-hexane/EtOAc) to give compound **9** (5.766 g, 99%) as a colourless syrup. $[\alpha]_D$ −106.2 (*c* 0.25, CHCl$_3$); $R_f$ 0.37 (7:3 *n*-hexane/EtOAc); $^1$H NMR (400 MHz, CDCl$_3$) $\delta$ = 7.83-7.22 (m, 12H, arom), 5.54 (d, *J* = 2.0 Hz, 1H, H-1), 5.35 (m, 1H, H-2), 5.29 (t, *J* = 3.4 Hz, 1H, H-4), 4.83 (dd, *J* = 32.5 Hz, *J* = 11.8 Hz, 2H, NAPC*H*$_2$), 4.47 (dq, *J* = 6.4 Hz, *J* = 1.5 Hz, 1H, H-5), 3.72 (s, 1H, H-3), 2.09 (s, 3H, OAc), 2.01 (s, 3H, OAc), 1.34 (d, *J* = 6.6 Hz, 3H, C*H*$_3$ talose) ppm; $^{13}$C NMR (100 MHz, CDCl$_3$) $\delta$ = 170.5, 170.0 (2C, 2 x C$_q$ OAc), 135.9, 133.6, 133.3, 133.1 (4C, C$_q$ arom), 131.5-126.0 (12C, arom), 85.3 (1C, C-1), 76.1 (1C, C-3), 74.9 (1C, NAP*C*H$_2$), 69.6, 69.1 (2C, C-2, C-4), 68.7 (1C, C-5), 21.2, 20.9

(2C, 2 x $CH_3$ OAc), 16.43 (1C, $CH_3$ talose) ppm; elemental analysis calcd (%) for $C_{27}H_{28}O_6S$ (480.16); C, 67.48; H, 5.87; S, 6.67; found: C, 67.34; H, 5.93; S, 6.71.

**Methyl [2,3-di-*O*-acetyl-4-*O*-(2-naphtyl)methyl-6-deoxy-1-thio-α-L-talopyranosyl]-(1→4)-2,3,6-tri-*O*-benzyl-α-D-glucopyranoside (11)**

To a solution of compound **10**[2] (2.00 g, 4.16 mmol) and compound **9** (2.90 g, 6.24 mmol, 1.4 equiv.) in dry $CH_2Cl_2$ (85 mL) 4 Å molecular sieves (2.5 g) were added. After stirring for 30 min at room 1temperature, the mixture was cooled to −50 °C and a solution of NIS (1.40 g, 6.24 mmol, 1.5 equiv. for the donor) and TfOH (109 µL, 1.25 mmol, 0.3 equiv. for the donor) in dry THF (2.5 mL) was added. Allowed to warm up to −15 °C, the mixture was stirred for 3 h at that temperature. When the TLC analysis (65:35 *n*-hexane/EtOAc) showed complete consumption of the donor, the reaction mixture was neutralized with $Et_3N$ (1.5 mL), diluted with $CH_2Cl_2$ (200 mL), and filtered. The filtrate was washed with an aqueous solution of $Na_2S_2O_3$ (10%, 2 x 75 mL), a saturated aqueous solution of $NaHCO_3$ (2 x 75 mL), and water (2 x 75 mL), dried, and concentrated. The crude product was purified by column chromatography on silica gel (95:5 $CH_2Cl_2$/EtOAc) to give compound **11** (3.32 g, 96%) as a colourless syrup. $[\alpha]_D$ −33.9 (*c* 0.26, $CHCl_3$); $R_f$ 0.39 (65:35 *n*-hexane/EtOAc); $^1H$ NMR (400 MHz, $CDCl_3$) $\delta$ = 7.81-7.13 (m, 22H arom), 5.17 (t, *J* = 3.4 Hz, 1H, H-3’), 5.09-5.05 (m, 3H, H-1’, H-2’, Bn$CH_2$a), 4.81-4.44 (m, 8H, H-1, 3 x Bn$CH_2$, Bn$CH_2$b), 4.13 (q, *J* = 6.7 Hz, 1H, H-5’), 3.94-3.84 (m, 2H, H-3, H-4), 3.77-3.71 (m, 2H, H-5, H-6a), 3.64 (d, *J* = 9.5 Hz, 1H, H-6b), 3.58 (dd, *J* = 9.0 Hz, *J* = 3.6 Hz, 1H, H-2), 3.34 (s, 4H, H-4’, C-1-O$CH_3$), 2.06 (s, 3H, $CH_3$ OAc), 1.97 (s, 3H, $CH_3$ OAc), 0.84 (d, *J* = 6.5 Hz, 3H, $CH_3$ talose) ppm; $^{13}C$ NMR (100 MHz, $CDCl_3$) $\delta$ = 170.9, 169.9 (2C, 2 x $C_q$ OAc), 138.8, 137.9, 137.9, 135.9, 133.2, 132.9 (6C, 6 x $C_q$ arom), 128.5-125.9 (22C, arom), 98.1 (1C, C-1’), 97.9 (1C, C-1), 80.4 (1C, C-2), 80.2 (1C, C-3), 75.9 (1C, C-4’), 74.2 (1C, C-4), 75.7, 74.9, 73.3, 73.2 (4C, NAP$CH_2$, 3 x Bn$CH_2$) 70.0 (1C, C-5), 69.1 (1C, C-3’), 68.4 (2C, C-2,’C-6), 67.1 (1C, C-5’), 55.2 (1C, C-1-O$CH_3$), 21.2, 20.9 (2C, 2 x $CH_3$ OAc), 16.2 (1C, $CH_3$ talose) ppm; MS (MALDI-TOF): *m*/*z* calcd for $C_{49}H_{54}NaO_{12}$, $[M+Na]^+$ 857.35; found: 857.27; elemental analysis calcd (%) for $C_{49}H_{54}O_{12}$ (834.36); C, 70.49; H, 6.52; found: C, 70.65; H, 6.58.

**Methyl (2,3-di-*O*-acetyl-6-deoxy-1-thio-α-L-talopyranosyl)-(1→4)-2,3,6-tri-*O*-benzyl-α-D-glucopyranoside (12)**

To a vigorously stirred solution of compound **11** (3.227 g, 3.868 mmol) in $CH_2Cl_2$ (61 mL) and $H_2O$ (7.0 mL), DDQ (1.32 g, 5.802 mmol, 1.5 equiv.) was added. After 30 min the

mixture was diluted with $CH_2Cl_2$ (250 mL) and extracted with saturated aqueous solution of $NaHCO_3$ (2 x 75 mL) and $H_2O$ (2 x 75 mL), dried and concentrated. The crude product was purified by silica gel chromatography (9:1 $CH_2Cl_2$/EtOAc) to give compound **12** (1.834 g, 68%) as a colourless syrup. $[\alpha]_D$ −17.1 (*c* 0.36, $CHCl_3$); $R_f$ 0.40 (9:1 $CH_2Cl_2$/EtOAc); $^1H$ NMR (400 MHz, $CDCl_3$) $\delta$ = 7.37-7.24 (m, 15H, arom), 5.12-5.00 (m, 4H, H-1', H-2', H-3', BnC$H_{2a}$), 4.76-4.59 (m, 4H, H-1, BnC$H_2$, BnC$H2_b$), 4.51 (d, $J$ = 2.2 Hz, 2H, BnC$H_2$), 4.11 (q, $J$ = 6.6 Hz, 1H, H-5'), 3.93-3.84 (m, 2H, H-3, H-4), 3.75-3.64 (m, 3H, H-5, H-6a,b), 3.59 (dd, $J$ = 8.9 Hz, $J$ = 3.5 Hz, 1H, H-2), 3.41 (d, $J$ = 10.7 Hz, 1H, H-4'), 3.36 (s, 3H, C-1-OC$H_3$), 2.27 (d, $J$ = 11.0 Hz, 1H, C-4'-O*H*), 2.10 (s, 3H, C$H_3$ OAc), 2.05 (s, 3H, C$H_3$ OAc), 0.83 (d, $J$ = 6.4 Hz, 3H, C$H_3$ talose) ppm; $^{13}C$ NMR (100 MHz, $CDCl_3$) $\delta$ = 170.0, 169.6 (2C, $C_q$ OAc), 138.8, 138.0 (3C, 3 x $C_q$ arom), 128.5-127.5 (15C, arom), 98.0 (1C, C-1), 97.5 (1C, C-1'), 80.5 (1C, C-2), 80.1 (1C, C-3), 75.8, 73.3, 73.2 (3C, 3 x Bn*C*$H_2$), 74.3 (1C, C-4), 70.0, 69.8 (2C, C-4', C-5), 68.5 (1C, C-6), 67.6 (2C, C-2', C-3'), 67.2 (1C, C-5'), 55.3 (1C, C-1-O*C*$H_3$), 21.0 (2C, 2 x *C*$H_3$ OAc), 15.8 (1C, *C*$H_3$ talose) ppm; MS (MALDI-TOF): *m/z* calcd for $C_{38}H_{46}NaO_{12}$, $[M+Na]^+$ 717.29; found: 717.30; elemental analysis calcd (%) for $C_{38}H_{46}O_{12}$ (694.30); C, 65.69; H, 6.67; found: C, 65.94; H, 6.70.

**Methyl [2,3,6-tri-*O*-benzyl-4-*O*-(2-naphtyl)methyl-α-D-glucopyranosyl]-(1→4)-(2,3-di-*O*-acetyl-6-deoxy-α-L-talopyranosyl)-(1→4)-2,3,6-tri-*O*-benzyl-α-D-glucopyranoside (14*α*) and methyl [2,3,6-tri-*O*-benzyl-4-*O*-(2-naphtyl)methyl-α/β-D-glucopyranosyl]-(1→4)-(2,3-di-*O*-acetyl-6-deoxy-α-L-talopyranosyl)-(1→4)-2,3,6-tri-*O*-benzyl-α-D-glucopyranoside (14β)**

**Method I.** To a solution of compound **12** (1.80 g, 2.592 mmol) and compound **13**[3] (2.65 g, 3.888 mmol, 1.5 equiv.) in dry $CH_2Cl_2$ (150 mL), 4 Å molecular sieves (2.5 g) were added. After stirring for 30 min at room temperature, the mixture was cooled to −50 °C and a solution of NIS (1.31 g, 5.832 mmol, 1.5 equiv. for the donor) and TMSOTf (316 µL, 1.750 mmol, 0.3 equiv.) in dry THF (2.0 mL) was added. Allowed to warm up the solution to −15 °C and stirred for 3 h at that temperature. When the TLC analysis (95:5 $CH_2Cl_2$/EtOAc) showed complete consumption of the donor, the reaction mixture was neutralized with $Et_3N$ (1.5 mL), diluted with $CH_2Cl_2$ (250 mL), and filtered. The filtrate was washed with an aqueous solution of $Na_2S_2O_3$ (10%, 2 x 75 mL), a saturated aqueous solution of $NaHCO_3$ (2 x 75 mL), and water (2 x 75 mL), dried, and concentrated. The crude product was purified by column chromatography on silica gel (95:5 $CH_2Cl_2$/EtOAc) to give compound **14*α*,β** as an inseparable 1:1 mixture (1.89 g, 58%) as a colourless syrup. $R_f$ 0.40 (95:5 $CH_2Cl_2$/EtOAc);

MS (MALDI-TOF): *m/z* calcd for $C_{76}H_{82}NaO_{17}$, $[M+Na]^+$ 1289.54; found: 1289.40.

**Method II.** To a stirred solution of the **24** (2.00 g, 1.690 mmol) in dry pyridine (4.2 mL), $Ac_2O$ (1.4 mL) was added. After 48 h at room temperature, all volatiles were evaporated, and the residue was co-evaporated twice with toluene. The crude product was purified by silica gel chromatography (6:4 *n*-hexane/EtOAc) to give compound **14α** (2.081 g, 97%) as a colourless syrup. $[\alpha]_D$ −8.5 (*c* 0.13, $CHCl_3$); $R_f$ 0.50 (6:4 *n*-hexane/EtOAc); $^1$H NMR (400 MHz, $CDCl_3$) $\delta$ = 7.80-7.19 (m, 37H, arom.), 5.27-4.40 (m, 19H, H-1, H-1', H-1", H-2', H-3', NAPC*H*$_2$, 6 x BnC*H*$_2$), 4.13-3.54 (m, 14H), 3.35 (s, 3H, C-1-OC*H*$_3$), 1.99, 1.88 (2 x s, 6H, 2 x C*H*$_3$ OAc), 0.89 (d, *J* = 6.4 Hz, 3H, C*H*$_3$ talose) ppm; $^{13}$C NMR (100 MHz, $CDCl_3$) $\delta$ = 171.1, 170.1 (2C, 2 x *C*O Ac), 139.0, 138.8, 138.4, 138.1, 138.0, 137.9, 136.0, 133.3, 132.9 (9C, $C_q$ arom), 128.5-125.8 (37C, arom), 98.0, 97.8, 97.2 (3C, C-1, C-1', C-1"), 81.6, 80.5, 80.4, 80.2, 77.8, 74.3, 71.1, 70.1, 68.8, 68.5, 67.2 (12C, skeleton carbons), 75.8, 75.5, 74.9, 73.6, 73.4, 73.1 (7C, NAP*C*H$_2$, 6 x Bn*C*H$_2$), 68.4, 68.3 (2C, C-6, C-6"), 55.3 (1C, C-1-O*C*H$_3$), 21.1 (2C, 2 x *C*H$_3$ OAc), 16.6 (1C, *C*H$_3$ talose) ppm; MS (MALDI-TOF): *m/z* calcd for $C_{76}H_{82}NaO_{17}$, $[M+Na]^+$ 1290.46; found: 1290.02; elemental analysis calcd (%) for $C_{76}H_{82}O_{17}$ (1266.56); C, 72.02; H, 6.54; found: C, 72.09; H, 6.62.

**Methyl (2,3,6-tri-*O*-benzyl-α-D-glucopyranosyl)-(1→4)-(2,3-di-*O*-acetyl-6-deoxy-α-L-talopyranosyl)-(1→4)-2,3,6-tri-*O*-benzyl-α-D-glucopyranoside (15) and methyl (2,3,6-tri-*O*-benzyl-β-D-glucopyranosyl)-(1→4)-(2,3-di-*O*-acetyl-6-deoxy-α-L-talopyranosyl)-(1→4)-2,3,6-tri-*O*-benzyl-α-D-glucopyranoside (16)**

**Method I.** To a vigorously stirred solution of **14α,β** (400 mg, 0.316 mmol) in $CH_2Cl_2$ (5 mL) and $H_2O$ (570 µL), DDQ (107 mg, 0.470 mmol, 1.5 equiv.) was added. After 30 min the mixture was diluted with $CH_2Cl_2$ (75 mL) and extracted with saturated aqueous solution of $NaHCO_3$ (2 x 20 mL) and $H_2O$ (2 x 20 mL), dried and concentrated. The crude product was purified by silica gel chromatography (55:45 *n*-hexane/EtOAc) to give compound **15** (109 mg, 31%) as a colourless syrup and compound **16** (81 mg, 23%) as a colourless syrup.

**Method II.** To a vigorously stirred solution of compound **14α** (2.00 g, 1.578 mmol) in $CH_2Cl_2$ (24 mL) and $H_2O$ (3.0 mL), DDQ (537 mg, 2.367 mmol, 1.5 equiv.) was added. After 30 min the mixture was diluted with $CH_2Cl_2$ (250 mL) and washed with saturated aqueous solution of $NaHCO_3$ (2 x 35 mL) and $H_2O$ (2 x 35 mL), dried and concentrated. The crude product was purified by silica gel chromatography (6:4 *n*-hexane/EtOAc) to give compound **15** (1.780 g, 77%) as a colourless syrup.

**Data of 15**: $[\alpha]_D$ +1.9 (*c* 0.11, $CHCl_3$); $R_f$ 0.53 (55:45 *n*-hexane/EtOAc); $^1$H NMR (400 MHz, $CDCl_3$) $\delta$ = 7.39-7.18 (m, 30H, arom), 5.28 (s, 1H, H-3'), 5.08-4.90 (m, 5H, H-1', H-1", H-2', BnC$H_2$), 4.64-4.45 (m, 11H, H-1, 5 x BnC$H_2$) 4.13 (dd, *J* = 13.5 Hz, *J* = 6.8 Hz, 1H, H-5'), 3.91-3.83 (m, 5H, H-3, H-3", H-5, H-5", H-6a), 3.77-3.55 (m, 7H, H-2, H-4, H-4', H-4", H-6b, H-6a", H-6b"), 3.50 (dd, *J* = 9.9 Hz, *J* = 3.1 Hz, 1H, H-2"), 3.35 (s, 3H, C-1-OC$H_3$), 2.46 (s, 1H, H-4"-O*H*), 2.00 (s, 3H, C$H_3$ OAc), 1.87 (s, 3H, C$H_3$ OAc), 0.89 (d, *J* = 6.3 Hz, 3H, C$H_3$ talose) ppm; $^{13}$C NMR (100 MHz, $CDCl_3$) $\delta$ = 171.2, 170.0 (2C, 2 x $C_q$ OAc) 138.9, 138.8, 138.2, 138.0, 138.0, 137.9 (6C, $C_q$ arom), 128.5-127.4 (30C, arom), 98.0 (1C, C-1), 97.5 (1C, C-1'), 97.2 (1C, C-1"), 80.7 (1C, C-5), 80.4 (1C, C-2), 80.1 (1C, C-5"), 80.0 (1C, C-2"), 75.8, 75.1, 73.6, 73.3, 73.1, 72.8 (6C, 6 x BnC$H_2$), 74.3 (1C, C-3), 73.7 (1C, C-4'), 71.0 (1C, C-4"), 70.8 (1C, C-3"), 70.1 (1C, C-4), 69.4 (1C, C-6"), 68.8 (1C, C-2'), 68.6 (1C, C-3'), 68.4 (1C, C-6), 67.1 (1C, C-5'), 55.3 (1C, C-1-O*C*$H_3$), 21.1, 21.0 (2C, 2 x *C*$H_3$ OAc), 16.5 (1C, *C*$H_3$ talose) ppm; MS (ESI-TOF): *m*/*z* calcd for $C_{65}H_{74}NaO_{17}$, $[M+Na]^+$ 1149.482; found: 1149.481.

**Data of 16**: $[\alpha]_D$ −21.8 (*c* 0.37, $CHCl_3$); $R_f$ 0.45 (55:45 *n*-hexane/EtOAc); $^1$H NMR (400 MHz, $CDCl_3$) $\delta$ = 7.39-7.12 (m, 30H, arom), 5.25-4.88 (m, 6H), 4.75-4.48 (m, 8H), 4.19-4.12 (m, 3H) 3.94-3.58 (m, 11H), 3.45-3.39 (m, 2H), 3.36 (s, 3H, C-1-OC$H_3$), 3.30-3.27 (m, 1H), 2.58 (s, 1H, C-4"-O*H*), 2.02 (s, 3H, C$H_3$ OAc), 1.96 (s, 3H, C$H_3$ OAc), 0.85 (d, *J* = 6.5 Hz, 3H, C$H_3$ talose) ppm; $^{13}$C NMR (100 MHz, $CDCl_3$) $\delta$ = 170.8, 170.5 (2C, 2 x $C_q$ OAc) 138.9, 138.3, 138.0, 137.9, 137.8 (6C, $C_q$ arom), 128.9-127.2 (30C, arom), 103.8 (1C, C-1"), 98.0, 97.9 (2C, C-1, C-1'), 84.0, 82.6, 80.5, 80.1, 74.3, 73.8, 73.5, 71.7, 70.1, 68.4, 66.8, 66.5 (12C, skeleton carbons) 75.8, 75.4, 75.2, 73.7, 73.3, 73.2 (6C, 6 x BnC$H_2$), 70.3, 68.7 (2C, C-6, C-6"), 55.3 (1C, C-1-O*C*$H_3$), 21.2, 21.1 (2C, 2 x *C*$H_3$ OAc), 16.2 (1C, *C*$H_3$ talose) ppm; MS (ESI-TOF): *m*/*z* calcd for $C_{65}H_{74}NaO_{17}$, $[M+Na]^+$ 1149.482; found: 1149.476.

**Methyl [4-*O*-(2-naphtyl)methyl-2,3-*O*-isopropylidene-6-deoxy-α-L-talopyranosyl]-(1→4)-2,3,6-tri-*O*-benzyl-α-D-glucopyranoside (17)**

**Method I.** To a solution of compound **10**[2] (142 mg, 0.305 mmol) and compound **7** (200 mg, 0.458 mmol, 1.5 equiv.) in dry $CH_2Cl_2$ (8.0 mL), 4 Å molecular sieves (0.5 g) and *sym*-collidine (10 μL, 0.073 mmol, 0.16 equiv. for donor) were added. After stirring for 30 min at room temperature, the mixture was cooled to −50 °C and a solution of NIS (154 mg, 0.687 mmol, 1.5 equiv. for the donor) and TMSOTf (37 μL, 0.206 mmol, 0.3 equiv.) in dry THF (750 μL) was added. The mixture was allowed to warm up to room temperature and stirred for 3 h at that temperature. When the TLC analysis (95:5 $CH_2Cl_2$/EtOAc) showed complete

consumption of the donor, the reaction mixture was neutralized with $Et_3N$ (0.5 mL), diluted with $CH_2Cl_2$ (100 mL), and filtered. The filtrate was washed with an aqueous solution of $Na_2S_2O_3$ (10%, 2 x 25 mL), a saturated aqueous solution of $NaHCO_3$ (2 x 25 mL), and water (2 x 25 mL), dried, and concentrated. The crude product was purified by column chromatography on silica gel (98:2 $CH_2Cl_2$/acetone) to give compound **17** (120 g, 50%) as a colourless syrup.

**Method II.** To a solution of compound **10**[2] (556 mg, 1.196 mmol) and compound **7** (783 mg, 1.794 mmol) in dry $CH_2Cl_2$ (21 mL) 4 Å molecular sieves (1.0 g) and *sym*-collidine (48 µL, 0.359 mmol, 0.2 equiv. for donor) were added. After stirring for 30 min at room temperature, the mixture was cooled to −40 °C and solutions of NIS (605 mg, 2.691 mmol, 1.5 equiv. for donor) in dry THF (829 µL) and AgOTf (110 mg, 0.28 mmol, 0.24 equiv. for donor) in dry toluene (828 µL) were added. After stirring for 3 h, TLC analysis (98:2 $CH_2Cl_2$/acetone) showed complete consumption of the donor. The reaction mixture was neutralized with $Et_3N$ (200 µL), diluted with $CH_2Cl_2$ (150 mL), and filtered. The filtrate was washed with an aqueous solution of $Na_2S_2O_3$ (10%, 2 x 50 mL), a saturated aqueous solution of $NaHCO_3$ (2 x 50 mL), and water (2 x 50 mL), dried, and concentrated. The crude product was purified by column chromatography on silica gel (65:35 *n*-hexane/EtOAc) to give compound **17** (824 mg, 87%) as a colourless syrup. $[\alpha]_D$ −2.0 (*c* 0.15, $CHCl_3$); $R_f$ 0.49 (98:2 $CH_2Cl_2$/acetone); $^1H$ NMR (400 MHz, $CDCl_3$) $\delta$ = 7.80-7.10 (m, 22H, arom), 5.09 (d, *J* = 3.6 Hz, 1H, H-1’), 4.88-4.45 (m, 9H, H-1, 3 x BnC*H*$_2$, NAPC*H*$_2$), 4.31 (dd, *J* = 4.4 Hz, *J* = 6.7 Hz, 1H, H-3’), 3.98 (dd, *J* = 3.6 Hz, *J* = 6.8 Hz, 1H, H-2’), 3.94-3.89 (m, 2H, H-4, H-5’) 3.84 (t, *J* = 9.2 Hz, 1H, H-3), 3.74-3.64 (m, 3H, H-5, H-6a,b), 3.53 (dd, *J* = 3.6 Hz, *J* = 9.4 Hz, 1H, H-2), 3.50 (t, *J* = 4.0 Hz, 1H, H-4’), 3.34 (s, 3H, C-1-OC*H*$_3$), 1.56, 1.35 (2 x s, 6H, 2 x C*H*$_3$ *i*p), 0.99 (d, *J* = 6.6 Hz, 3H, C*H*$_3$ talose) ppm; $^{13}C$ NMR (100 MHz, $CDCl_3$) $\delta$ = 139.0, 138.2, 137.9, 135.3, 133.1, 133.0 (6C, $C_q$ arom), 128.4-126.0 (22C, arom), 110.0 (1C, $C_q$ *i*p), 98.0 (1C, C-1), 97.5 (1C, C-1’), 80.3 (1C, C-3), 79.9 (1C, C-2), 75.5 (1C, C-2’), 74.9 (1C, C-4), 74.2 (1C, C-3’), 72.8 (1C, C-4’), 70.2 (1C, C-5), 68.5 (1C, C-6), 67.2 (1C, C-5’), 75.5, 73.9, 73.5, 73.3 (4C, 3 x BnC*H*$_2$, NAP*C*H$_2$), 55.1 (1C, C-1-O*C*H$_3$), 26.5, 25.4 (2C, 2 x *C*H$_3$ *i*p), 16.5 (1C, *C*H$_3$ talose) ppm; MS (MALDI-TOF): *m*/*z* calcd for $C_{48}H_{54}NaO_{10}$, $[M+Na]^+$ 813.36; found: 813.85; elemental analysis calcd (%) for $C_{48}H_{54}O_{10}$ (790.95); C, 72.89; H, 6.88; found: C, 72.94; H, 6.93.

**Methyl (2,3-*O*-isopropylidene-6-deoxy-α-L-talopyranosyl)-(1→4)-2,3,6-tri-*O*-benzyl-α-D-glucopyranoside (18)**

**Method I.** To a vigorously stirred solution of compound **17** (824 mg, 1.042 mmol) in $CH_2Cl_2$ (16 mL) and $H_2O$ (2.0 mL), DDQ (355 mg, 1.563 mmol, 1.5 equiv.) was added. After 30 min the mixture was diluted with $CH_2Cl_2$ (125 mL) and extracted with a saturated aqueous solution of $NaHCO_3$ (2 x 35 mL) and $H_2O$ (2 x 35 mL), dried and concentrated. The crude product was purified by silica gel chromatography (95:5 $CH_2Cl_2$/acetone) to give compound **18** (536 mg, 79%) as a colourless syrup.

**Method II.** To a stirred solution of the **23** (3.30 g, 5.071 mmol) in dry $CH_2Cl_2$ (53 mL), 4 Å MS (1.74 g) and PCC (8.68 g) were added. After 24 h at room temperature, the mixture was filtered through out a pod of Celite, washed with 4:1 mixture of *n*-hexane/EtOAc and concentrated. The crude product was purified by silica gel chromatography (7:3 *n*-hexane/acetone) to give compound **4'-ulose** derivative (2.70 g, 82%) as a colourless syrup. $[\alpha]_D$ −6.3 (*c* 0.16, $CHCl_3$); $R_f$ 0.44 (7:3 *n*-hexane/acetone); $^1H$ NMR (400 MHz, $CDCl_3$) $\delta$ = 7.35-7.24 (m, 15H, arom), 5.14 (s, 1H, H-1'), 5.04-4.56 (m, 8H, H-1, 3 x $BnCH_2$), 4.31 (dt, *J* = 3.8 Hz, *J* = 13.9 Hz, 2H), 3.94 (t, *J* = 9.3 Hz, 1H), 3.87 (t, *J* = 9.1 Hz, 1H), 3.72 (dd, *J* = 2.4 Hz, *J* = 9.6 Hz, 1H), 3.65-3.64 (m, 2H), 3.59 (dd, *J* = 3.6 Hz, *J* = 9.2 Hz, 1H), 3.36 (s, 3H, C-1-$OCH_3$), 1.42, 1.31 (2 x s, 6H, 2 x $CH_3$ ip), 1.02 (d, *J* = 6.7 Hz, 3H, $CH_3$ rhamnose) ppm; $^{13}C$ NMR (100 MHz, $CDCl_3$) $\delta$ = 205.6 (1C, C-4'), 138.5, 137.9, 137.4 (3C, $C_q$ arom), 128.5-127.8 (15C, arom), 111.1 (1C, $C_q$ *i*p), 97.9, 97.0 (2C, C-1, C-1'), 80.5, 79.9, 79.0, 76.0, 74.9, 70.6, 69.9 (7C, skeleton carbons), 75.5, 73.7, 73.3 (3C, 3 x $BnCH_2$), 68.6 (1C, C-6), 55.3 (1C, C-1-$OCH_3$), 26.8, 25.6 (2C, 2 x $CH_3$ *i*p), 15.0 (1C, $CH_3$ rhamnose) ppm; MS (MALDI-TOF): *m*/*z* calcd for $C_{37}H_{44}NaO_{10}$, $[M+Na]^+$ 671.28; found: 671.35; elemental analysis calcd (%) for $C_{37}H_{44}O_{10}$ (648.29); C, 68.50; H, 6.84 found: C, 68.56; H, 6.90. To a stirred solution of the **4'-ulose** derivative (140 mg, 0.216 mmol) in dry MeOH (5.0 mL), $NaBH_4$ (12 mg, 0.324 mmol, 1.5 equiv.) was added. After 1 h at room temperature the mixture was neutralized with 60% AcOH (1.0 mL) and concentrated. The crude product was purified by silica gel chromatography (7:3 *n*-hexane/acetone) to give compound **18** (134 mg, 96%) as a colourless syrup. $[\alpha]_D$ +15.6 (*c* 0.37, $CHCl_3$); $R_f$ 0.58 (95:5 $CH_2Cl_2$/acetone); $^1H$ NMR (400 MHz, $CDCl_3$) $\delta$ = 7.36-7.25 (m, 15H, arom), 5.16 (d, *J* = 0.9 Hz, 1H, H-1'), 5.00-4.56 (m, 7H, H-1, 3 x $BnCH_2$), 4.07 (dd, *J* = 5.0 Hz, *J* = 6.3 Hz, 1H, H-3'), 3.98-3.93 (m, 2H, H-2', H-4), 3.88-3.83 (m, 2H, H-3, H-5') 3.76-3.72 (m, 1H, H-5), 3.67 (d, *J* = 2.7 Hz, 2H, H-6a,b), 3.57 (dd, *J* = 3.6 Hz, *J* = 9.4 Hz, 1H, H-2), 3.36 (s, 3H, C-1-$OCH_3$), 3.33 (t, *J* = 5.5 Hz, 1H, H-4'), 2.05 (d, *J* = 6.6 Hz, 1H, H-4'-O*H*), 1.54, 1.33 (2 x s, 6H, 2 x $CH_3$ *i*p), 0.97 (d, *J* = 6.5 Hz, 3H, $CH_3$ talose) ppm; $^{13}C$ NMR (100 MHz, $CDCl_3$) $\delta$ = 139.0, 138.2, 137.8 (3C, $C_q$ arom), 128.5-127.5 (15C, arom), 109.3 (1C, $C_q$ *i*p), 98.1 (1C, C-1), 97.0 (1C, C-1'), 80.3 (2C, C-3, C-2), 73.9

(1C, C-2’), 73.7 (1C, C-4), 73.3 (1C, C-3’), 70.2 (1C, C-5), 69.9 (1C, C-6), 67.1 (1C- C-4’), 65.6, (1C, C-5’) 75.8, 73.6, 73.5 (3C, 3 x Bn$CH_2$), 55.3 (1C, C-1-O$CH_3$), 26.0, 25.3 (2C, 2 x $CH_3$ *i*p), 16.5 (1C, $CH_3$ talose) ppm; MS (MALDI-TOF): *m*/*z* calcd for $C_{37}H_{46}NaO_{10}$, $[M+Na]^+$ 673.30; found: 673.20; elemental analysis calcd (%) for $C_{37}H_{46}O_{10}$ (650.31); C, 68.29; H, 7.13; found: C, 68.52; H, 7.17.

**Methyl [2,3,6-tri-*O*-benzyl-4-*O*-(2-naphtyl)methyl-α-D-glucopyranosyl]-(1→4)-(2,3-*O*-isopropylidene-6-deoxy-α-L-talopyranosyl)-(1→4)-2,3,6-tri-*O*-benzyl-α-D-glucopyranoside (19)**

To a solution of compound **18** (530 mg, 0.814 mmol) and compound **13**[3] (834 mg, 1.221 mmol) in dry $CH_2Cl_2$ (14 mL) 4 Å molecular sieves (1.5 g) and *sym*-collidine (33 μL, 0.244 mmol, 0.2 equiv. for donor) were added. After stirring for 30 min at room temperature, the mixture was cooled to −40 °C and solutions of NIS (412 mg, 1.832 mmol, 1.5 equiv. for donor) in dry THF (564 μL) and AgOTf (75 mg, 0.293 mmol, 0.24 equiv. for donor) in dry toluene (564 μL) were added. After stirring for 3 h, TLC analysis (97:3 $CH_2Cl_2$/acetone) showed complete consumption of the donor. The reaction mixture was neutralized with $Et_3N$ (200 μL), diluted with $CH_2Cl_2$ (150 mL), and filtered. The filtrate was washed with 10% aqueous solution of $Na_2S_2O_3$ (2 x 50 mL), saturated aqueous solution of $NaHCO_3$ (2 x 50 mL) and water (2 x 50 mL), dried, and concentrated. The crude product was purified by column chromatography on silica gel (97:3 $CH_2Cl_2$/acetone) to give compound **19** (706 mg, 71%) as a colourless syrup. $[\alpha]_D$ +17.0 (*c* 0.12, $CHCl_3$); $R_f$ 0.45 (65:35 *n*-hexane/EtOAc); $^1H$ NMR (400 MHz, $CDCl_3$) $\delta$ = 7.79-7.18 (m, 37H, arom), 5.22 (s, 1H, H-1”), 5.00-4.53 (m, 17H, H-1, H-1’, H-3’, NAPC$H_2$, 6 x BnC$H_2$), 4.19-4.13 (m, 2H, H-4’, H-5’), 4.03-3.99 (m, 1H, H-3”), 3.95-3.52 (m, 12H, H-2, H-2’, H-2”, H-3, H-4, H-4”, H-5, H-5”, H-6a,b, H-6”a,b), 3.36 (s, 3H, C-1-OC$H_3$), 1.51, 1.33 (2 x s, 9H, 2 x C$H_3$ isopropylidene, C$H_3$ talose) ppm; $^{13}C$ NMR (100 MHz, $CDCl_3$) $\delta$ = 139.0, 138.9, 138.5, 138.3, 138.2, 137.7, 135.6, 133.3, 132.9 (9C, $C_q$ arom), 128.4-125.9 (37C, arom), 110.6 (1C, $C_q$ isopropylidene), 98.1 (1C, C-1), 97.5 (1C, C-1’), 94.3 (1C, C-1”), 81.7 (1C, C-3”), 80.4 (1C, C-3), 79.7 (1C, C-2), 79.4 (1C, C-2”), 77.3 (1C, C-5), 77.0 (1C, C-2’), 76.2 (1C, C-5”), 75.5, 75.5, 75.2, 73.5 (8C, NAP$CH_2$, 6 x Bn$CH_2$, C-3’), 70.8, 70.3 (2C, C-4, C-4”), 69.4 (2C, C-4’, C-5’), 68.5, 68.2 (2C, C-6, C-6”), 55.1 (1C, C-1-O$CH_3$), 27.5, 25.5 (2C, 2 x $CH_3$ isopropylidene), 14.8 (1C, $CH_3$ talose) ppm; MS (MALDI-TOF): *m*/*z* calcd for $C_{75}H_{82}NaO_{15}$, $[M+Na]^+$ 1245.55; found: 1245.55; elemental analysis calcd (%) for $C_{75}H_{82}O_{15}$ (1222.57); C, 73.63; H, 6.76; found: C, 73.41; H, 6.84.

**Methyl (2,3,6-tri-*O*-benzyl-α-D-glucopyranosyl)-(1→4)-(2,3-*O*-isopropylidene-6-deoxy-α-L-talopyranosyl)-(1→4)-2,3,6-tri-*O*-benzyl-α-D-glucopyranoside (20)**

To a vigorously stirred solution of compound **19** (159 mg, 0.130 mmol) in $CH_2Cl_2$ (2 mL) and $H_2O$ (234 μL), DDQ (44 mg, 0.195 mmol, 1.5 equiv.) was added. After 30 min the mixture was diluted with $CH_2Cl_2$ (50 mL) and extracted with saturated aqueous solution of $NaHCO_3$ (2 x 15 mL) and $H_2O$ (2 x 15 mL), dried and concentrated. The crude product was purified by silica gel chromatography (6:4 *n*-hexane/EtOAc) to give compound **20** (89 mg, 63%) as a colourless syrup. $[\alpha]_D$ +26.5 (*c* 0.16, $CHCl_3$); $R_f$ 0.45 (6:4 *n*-hexane/EtOAc); $^1H$ NMR (400 MHz, $CDCl_3$) $\delta$ = 7.40-7.19 (m, 30H, arom), 5.21 (d, *J* = 3.6 Hz, 1H, H-1”), 4.98-4.49 (m, 14H, H-1’, H-1”, 6 x BnC$H_2$), 4.19-3.50 (m, 16H), 3.36 (s, 3H, C-1-OC$H_3$), 2.42 (s, 1H, C-4”-O*H*), 1.50, 1.31 (2 x s, 9H, 2 x C$H_3$ isopropylidene, C$H_3$ talose) ppm; $^{13}C$ NMR (100 MHz, $CDCl_3$) $\delta$ = 139.1, 138.9, 138.4, 138.4, 138.2, 137.9 (6C, $C_q$ arom), 128.5-127.4 (30C, arom), 110.6 (1C, $C_q$ isopropylidene), 98.2, 97.6, 94.3 (3C, C-1, C-1’, C-1”), 81.0, 80.4, 79.8, 79.1, 77.0, 76.3, 72.0, 70.7, 70.4, 70.4, 69.4, 69.4 (12C, skeleton carbons), 75.6, 75.2, 73.7, 73.6, 73.6, 71.7 (6C, 6 x Bn*C*$H_2$), 69.2, 68.6 (2C, 2 x C-6), 55.2 (1C, C-1-O*C*$H_3$), 27.5, 25.6 (2C, 2 x *C*$H_3$ isopropylidene), 14.9 (1C, *C*$H_3$ talose) ppm; MS (ESI-TOF): *m*/*z* calcd for $C_{64}H_{74}NaO_{15}$, $[M+Na]^+$ 1105.492; found: 1105.481.

**Methyl (2,3,4-tri-*O*-acetyl-α-L-rhamnopyranosyl)-(1→4)-2,3,6-tri-*O*-benzyl-α-D-glucopyranoside (21)**

To a solution of compound **10**[2] (3.5 g, 7.540 mmol) and compound **5**[4] (4.2 g, 11.31 mmol, 1.5 equiv.) in dry $CH_2Cl_2$ (125 mL), 4 Å molecular sieves (3.5 g) were added. After stirring for 30 min at room temperature, the mixture was cooled to −50 °C and a solution of NIS (3.8 g, 16.90 mmol, 1.5 equiv. for the donor) and TfOH (296 μL, 3.393 mmol, 0.3 equiv.) in dry THF (4.0 mL) was added. The mixture was allowed to warm up to −10 °C and stirred for 3 h at that temperature. When the TLC analysis (7:3 *n*-hexane/acetone) showed complete consumption of the donor, the reaction mixture was neutralized with $Et_3N$ (0.5 mL), diluted with $CH_2Cl_2$ (350 mL), and filtered. The filtrate was washed with an aqueous solution of $Na_2S_2O_3$ (10%, 2 x 75 mL), a saturated aqueous solution of $NaHCO_3$ (2 x 75 mL), and water (2 x 75 mL), dried, and concentrated. The crude product was purified by column chromatography on silica gel (7:3 *n*-hexane/acetone) to give compound **21** (5.439 g, 98%) as a colourless syrup. $[\alpha]_D$ −14.7 (*c* 0.30, $CHCl_3$); $R_f$ 0.45 (7:3 *n*-hexane/acetone); $^1H$ NMR (400 MHz, $CDCl_3$) $\delta$ = 7.38-7.23 (m, 15H, arom), 5.27-4.51 (m, 11H, H-1, H-1’, H-2’, H-3’, H-4’, 3 x BnC$H_2$), 4.05 (dq, *J* = 6.1 Hz, *J* = 12.3 Hz, 1H, H-5’), 3.92-3.85 (m, 2H), 3.75-3.73 (m,

2H) 3.68-3.64 (m, 1H), 3.59 (dd, *J* = 3.4 Hz, *J* = 9.1 Hz, 1H, H-2), 3.36 (s, 3H, C-1-OC*H*3), 2.05, 1.99, 1.98 (3 x s, 9H, 3 x C*H*3 OAc), 0.78 (d, *J* = 6.2 Hz, 3H, C*H*3 rhamnose) ppm; $^{13}$C NMR (100 MHz, $CDCl_3$) $\delta$ = 170.3, 170.1, 169.9 (3C, 3 x $C_q$ OAc), 138.8, 138.0, 137.9 (3C, $C_q$ arom), 128.5-127.3 (15C, arom), 98.0, 97.2 (2C, C-1, C-1'), 80.4, 79.8, 75.0, 71.0, 70.3, 69.9, 69.2, 66.8 (8C, skeleton carbons), 75.5, 73.4, 73.2 (3C, 3 x BnC$H_2$), 68.5 (1C, C-6), 55.3 (1C, C-1-O$CH_3$), 20.9, 20.8 (2C, 2 x $CH_3$ OAc), 17.0 (1C, $CH_3$ rhamnose) ppm; MS (MALDI-TOF): *m/z* calcd for $C_{40}H_{48}NaO_{13}$, $[M+Na]^+$ 759.30; found: 760.11; elemental analysis calcd (%) for $C_{40}H_{48}O_{13}$ (736.31); C, 65.21; H, 6.57; found: C, 65.09; H, 6.62.

**Methyl α-L-rhamnopyranosyl-(1→4)-2,3,6-tri-*O*-benzyl-α-D-glucopyranoside (22)**

To a stirred solution of compound **21** (5.439 g, 7.386 mmol) in MeOH (150 mL), NaOMe (50 mg, 0.926 mmol) was added. After 24 h the mixture was neutralized with Amberlite IR-120 $H^+$ ion exchanged resin, filtered, washed with MeOH and concentrated. The crude product was purified by silica gel chromatography (9:1 $CH_2Cl_2$/MeOH) to give **22** (4.350 g, 96%) as a colourless syrup. $[\alpha]_D$ −23.3 (*c* 0.09, $CHCl_3$); $R_f$ 0.48 (9:1 $CH_2Cl_2$/MeOH); $^1$H NMR (400 MHz, $CDCl_3$) $\delta$ = 7.37-7.21 (m, 15H, arom), 5.03-4.45 (m, 8H, H-1, H-1', 3 x BnC$H_2$), 3.84-3.75 (m, 3H), 3.68-3.65 (m, 2H), 3.58-3.52 (m, 4H), 3.35 (s, 3H, C-1-OC$H_3$), 3.32-3.27 (m, 1H), 2.89-2.73, (m, 3H, 3 x O*H*), 0.93 (d, *J* = 6.1 Hz, 3H, C$H_3$ rhamnose) ppm; $^{13}$C NMR (100 MHz, $CDCl_3$) $\delta$ = 138.9, 138.0, 137.7 (3C, $C_q$ arom), 128.6-127.5 (15C, arom), 100.0, 98.0 (2C, C-1, C-1'), 80.5, 80.1, 75.3, 73.2, 71.7, 71.1, 70.1, 68.6 (8C, skeleton carbons), 75.4, 73.7, 73.4 (3C, 3 x BnC$H_2$), 68.7 (1C, C-6), 55.4 (1C, C-1-O$CH_3$), 17.4 (1C, $CH_3$ rhamnose) ppm; MS (MALDI-TOF): *m/z* calcd for $C_{34}H_{42}NaO_{10}$, $[M+Na]^+$ 633.27; found: 633.22; elemental analysis calcd (%) for $C_{34}H_{42}O_{10}$ (610.28); C, 66.87; H, 6.93; found: C, 67.04; H, 6.99.

**Methyl (2,3-*O*-isopropylidene-α-L-rhamnopyranosyl)-(1→4)-2,3,6-tri-*O*-benzyl-α-D-glucopyranoside (23)**

To a stirred solution of **22** compound (4.30 g, 7.046 mmol) in dry DMF (10 mL), 2,2-dimethoxypropane (2.6 mL, 21.138 mmol) and *p*-TSA (38 mg) were added. After 24 h at room temperature, the mixture was neutralized with TEA (200 μL) and concentrated. The crude product was purified by silica gel chromatography (6:4 *n*-hexane/acetone) to give the **23** (4.320 g, 94%) as a colourless syrup. $[\alpha]_D$ +13.3 (*c* 0.15, $CHCl_3$); $R_f$ 0.54 (6:4 *n*-hexane/acetone); $^1$H NMR (400 MHz, $CDCl_3$) $\delta$ = 7.39-7.24 (m, 15H, arom), 5.15 (s, 1H, H-1'), 5.03-4.56 (m, 7H, H-1, 3 x BnC$H_2$), 4.06 (d, *J* = 5.7 Hz, 1H), 3.97-3.71 (m, 5H), 3.65-

3.64 (m, 2H, H-6a,b), 3.57 (dd, *J* = 3.6 Hz, *J* = 9.0 Hz, 1H, H-2), 3.36 (s, 3H, C-1-OC$H_3$), 3.28-3.26 (m, 1H, H-4'), 2.21-2.13, (m, 1H, H-4'-O*H*), 1.48, 1.28 (2 x s, 6H, 2 x C$H_3$ ip), 0.95 (d, *J* = 6.1 Hz, 3H, C$H_3$ rhamnose) ppm; $^{13}$C NMR (100 MHz, $CDCl_3$) $\delta$ = 138.9, 138.1, 137.7 (3C, $C_q$ arom), 128.5-127.5 (15C, arom), 109.4 (1C, $C_q$ *i*p), 98.0, 97.0 (2C, C-1, C-1'), 80.4, 80.1, 78.4, 76.1, 74.3, 70.1, 66.5 (8C, skeleton carbons), 75.6, 73.7, 73.4 (3C, 3 x BnC$H_2$), 68.9 (1C, C-6), 55.3 (1C, C-1-O$CH_3$), 28.0, 26.2 (2C, 2 x $CH_3$ *i*p), 17.2 (1C, $CH_3$ rhamnose) ppm; MS (MALDI-TOF): *m*/*z* calcd for $C_{37}H_{46}NaO_{10}$, $[M+Na]^+$ 673.30; found: 673.23; elemental analysis calcd (%) for $C_{37}H_{46}O_{10}$ (650.31); C, 68.29; H, 7.13; found: C, 68.53; H, 7.21.

**Methyl (2,3,6-tri-*O*-benzyl-4-*O*-(2-naphtyl)methyl -α-D-glucopyranosyl)-(1→4)-(2,3-di-*O*-acetyl-6-deoxy-α-L-talopyranosyl)-(1→4)-2,3,6-tri-*O*-benzyl-α-D-glucopyranoside (24)**
To a stirred solution of compound **19** (1.864 g, 1.524 mmol) in $CH_2Cl_2$ (19 mL), TFA (90%, 5.5 mL) was added. After 20 min at room temperature, the mixture was neutralized with $NaHCO_3$, diluted with $CH_2Cl_2$ (250 mL) and washed with saturated aqueous solution of $NaHCO_3$ (2 x 35 mL) and $H_2O$ (2 x 35 mL), dried and concentrated. The crude product was purified by silica gel chromatography (97:3 $CH_2Cl_2$/acetone) to give compound **24** (2.114 g, 93%) as a colourless syrup. $[\alpha]_D$ −7.7 (*c* 0.22, $CHCl_3$); $R_f$ 0.42 (97:3 $CH_2Cl_2$/EtOAc); $^1$H NMR (400 MHz, $CDCl_3$) $\delta$ = 7.80-7.18 (m, 37H, arom.), 5.07-4.33 (m, 17H, H-1, H-1', H-1", NAPC$H_2$, 6 x BnC$H_2$), 4.04-3.52 (m, 16H), 3.40 (s, 1H, O*H*), 3.35 (s, 3H, C-1-OC$H_3$), 3.14 (d, *J* = 11.6 Hz, 1H, O*H*), 0.82 (d, *J* = 6.5 Hz, 3H, C$H_3$ talose) ppm; $^{13}$C NMR (100 MHz, $CDCl_3$) $\delta$ = 138.9, 138.4, 138.1, 137.8, 137.1, 135.5, 133.3, 133.0 (9C, $C_q$ arom), 128.7-125.8 (37C, arom), 101.7, 100.9, 98.0 (3C, C-1, C-1', C-1"), 85.2, 82.6, 80.5, 80.3, 79.9, 77.6, 74.0, 71.4, 71.2, 70.3, 67.0, 66.0 (12C, skeleton carbons), 75.8, 75.7, 75.2, 74.7, 73.6, 73.3 (7C, NAP$CH_2$, 6 x Bn$CH_2$), 68.8, 68.0 (2C, C-6, C-6"), 55.4 (1C, C-1-O$CH_3$), 16.9 (1C, $CH_3$ talose) ppm; MS (MALDI-TOF): *m*/*z* calcd for $C_{72}H_{78}NaO_{15}$, $[M+Na]^+$ 1205.52; found: 1205.61; elemental analysis calcd (%) for $C_{72}H_{78}O_{15}$ (1182.53); C, 73.08; H, 6.64; found: C, 73.16; H, 6.69.

**Methyl (6-*O*-benzyl-2,3,4-tri-*O*-methyl-α-D-glucopyranosyl)-(1→4)-[2,3-di-*O*-acetyl-6-*O*-(2-naphtyl)methyl-β-D-glucopyranosyl]-(1→4)-(2,3,6-tri-*O*-benzyl-α-D-glucopyranosyl)-(1→4)-(2,3-*O*-isopropylidene-6-deoxy-α-L-talopyranosyl)-(1→4)-2,3,6-tri-*O*-benzyl-α-D-glucopyranoside (26) and Methyl-(6-*O*-benzyl-2,3,4-tri-*O*-methyl-α-D-glucopyranosyl)-(1→4)-[2,3-di-*O*-acetyl-6-*O*-(2-naphtyl)methyl-β-D-glucopyranosyl]-(1→4)-(2,3,6-tri-*O*-**

**benzyl-α-D-glucopyranosyl)-(1→4)-(6-deoxy-α-L-talopyranosyl)-(1→4)-2,3,6-tri-*O*-benzyl-α-D-glucopyranoside (27)**

To a solution of compound **20** (83 mg, 0.077 mmol) and compound **25**[5] (91 mg, 0.115 mmol, 1.5 equiv.) in dry $CH_2Cl_2$ (4.5 mL), 4 Å molecular sieves (0.5 g) were added. After stirring for 30 min at room temperature, the mixture was cooled to −50 °C and the solution of NIS (39 mg, 0.172 mmol, 1.5 equiv. for the donor) and TMSOTf (9.0 µL, 0.052 mmol, 0.3 equiv.) in dry THF (500 µL) was added. Allowed to warm up to +10 °C the mixture was stirred for 4 h at that temperature. When the TLC analysis (6:4 *n*-hexane/EtOAc) showed complete consumption of the donor, the reaction mixture was neutralized with $Et_3N$ (50 µL), diluted with $CH_2Cl_2$ (75 mL), and filtered. The filtrate was washed with an aqueous solution of $Na_2S_2O_3$ (10%, 2 x 15 mL), a saturated aqueous solution of $NaHCO_3$ (2 x 15 mL), and water (2 x 15 mL), dried, and concentrated. The crude product was purified by column chromatography on silica gel (6:4 *n*-hexane/EtOAc) to give compound **26** (29 mg, 21%) as a colourless syrup and compound **27** (49 mg, 36%) as a colourless syrup.

After characterization, compound **26** was converted to **27** as follows: To a solution of compound **26** (29 mg, 0.016 mmol) in $CH_2Cl_2$ (200 µL) TFA (90%, 60 µL) was added and the mixture was stirred for 30 min at room temperature. After the reaction had been completed, the mixture was neutralized with $NaHCO_3$ and concentrated. The residue was purified by column chromatography on silica gel (65:35 *n*-hexane/acetone) to give compound **27** (23 mg, 81%) as a colourless syrup.

**Data of 26**: $[\alpha]_D$ +44.1 (*c* 0.12, $CHCl_3$); $R_f$ 0.13 (6:4 *n*-hexane/EtOAc); $^1$H NMR (400 MHz, $CDCl_3$) $\delta$ = 7.81-7.11 (m, 42H, arom), 5.12-5.07 (m, 3H, H-1-F, H-3-E, H-1-D), 4.98 (d, $J$ = 11.6 Hz, 1H, BnC$H_{2a}$), 4.90 (d, $J$ = 5.1 Hz, 1H, H-1-G), 4.88-4.82 (m, 3H, H-2-E, BnC$H_2$), 4.87-4.68 (m, 3H, BnC$H_2$), 4.46-4.52 (m, 8H, H-1-H, H-3-G, H-1-E, BnC$H_2$), 4.46-4.26 (m, 6H, BnC$H_2$, NAPC$H_2$), 4.14 (t, $J$ = 6.7 Hz, 1H, H-5-G), 4.06 (dd, $J$ = 6.5 Hz, $J$ = 3.6 Hz, 1H, H-4-G), 3.94-3.82 (m, 7H, H-2-G, H-3-F, H-3-H, H-4-F, H-4-E, H-5-H, H-6a-H), 3.76-3.72 (m, 2H, H-4-H, H-6a-F), 3.69 (dd, $J$ = 11.2 Hz, $J$ = 1.3 Hz, 1H, H-6b-H), 3.68-3.55 (m, 4H, H-5-D, H-5-F, H6a,b-E), 3.58 (s, 3H, C-3-D-OC$H_3$), 3.53-3.48 (m, 3H, H-2-F, H-2-H, H-6b-F), 3.46-3.35 (m, 3H, H-3-D, H-6a,b-D), 3.41 (s, 3H, C-4-D-OC$H_3$), 3.40 (s, 3H, C-2-D-OC$H_3$), 3.37 (s, 3H, C-1-H-OC$H_3$), 2.04, 1.89 (2 x s, 6H, 2 x C$H_3$ OAc), 1.50, 1.31 (2 x s, 6H, 2 x C$H_3$ isopropylidene), 1.30 (d, $J$ = 7.1 Hz, 3H, C$H_3$ talose) ppm; $^{13}$C NMR (100 MHz, $CDCl_3$) $\delta$ = 170.1, 169.8 (2C, 2 x $C_q$ OAc), 139.7, 139.1, 138.7, 138.4, 138.3, 138.2, 137.7, 136.2, 133.4, 133.0 (10C, $C_q$ arom), 128.8-125.8 (42C, arom), 110.8 (1C, $C_q$ isopropylidene), 100.0 (1C, C-1-E), 98.2 (1C, C-1-H), 97.9 (1C, C-1-D), 97.6 (1C, C-1-G), 95.5 (1C, C-1-F),

83.3 (1C, C-3-D), 81.9 (1C, C-2-D), 80.5 (1C, C-3-H), 80.0 (1C, C-3-F), 79.9 (1C, C-2-H), 79.4 (1C, C-4-D), 78.9 (1C, C-2-F), 76.7 (1C, C-4-F), 76.3 (1C, C-5-H), 75.3 (1C, C-3-E), 75.2 (1C, C-5-E), 75.0 (1C, C-4-E), 73.0 (1C, C-2-E), 72.3 (1C, C-3-G), 71.3 (1C, C-5-D), 70.7 (1C, C-5-F), 70.4 (1C, C-4-H), 70.2 (1C, C-4-G), 69.6 (1C, C-5-G), 75.6, 73.8, 73.7, 73.6, 73.4 (8C, NAP*C*$H_2$, 7 x Bn*C*$H_2$), 68.8 (1C, C-6-E), 68.6 (1C, C-6-H), 68.5 (1C, C-6-D), 67.7 (1C, C-6-F), 60.8 (1C, C-3-D-O*C*$H_3$), 60.5 (1C, C-4-D-O*C*$H_3$), 59.4 (1C, C-2-D-O*C*$H_3$), 55.3 (1C, C-1-H-O*C*$H_3$), 27.6, 25.7 (2C, 2 x *C*$H_3$ isopropylidene), 21.1, 20.8 (2C, 2 x *C*$H_3$ OAc), 14.9 (1C, *C*$H_3$ talose) ppm; MS (MALDI-TOF): *m*/*z* calcd for $C_{101}H_{118}NaO_{27}$, $[M+Na]^+$ 1787.02; found: 1787.10; elemental analysis calcd (%) for $C_{101}H_{118}O_{27}$ (1762.79); C, 68.77; H, 6.74 found: C, 68.94; H, 6.81.

**Data of 27**: $[\alpha]_D$ +4.4 (*c* 0.36, $CHCl_3$); $R_f$ 0.35 (65:35 *n*-hexane/acetone); $^1H$ NMR (400 MHz, $CDCl_3$) $\delta$ = 7.81-7.11 (m, 42H, arom), 5.23-4.24 (m, 23H, 5 x H-1, H-2-E, H-3-E, NAPC$H_2$, 7 x BnC$H_2$), 4.01-3.03 (m, 28H), 3.58, 3.47, 3.41, 3.40 (4 x s, 12H, 4 x OC$H_3$), 2.01, 1.92 (2 x s, 6H, 2 x C$H_3$ OAc), 0.75 (d, *J* = 6.5 Hz, 3H, C$H_3$ talose) ppm; $^{13}C$ NMR (100 MHz, $CDCl_3$) $\delta$ = 170.1, 169.6 (2C, 2 x $C_q$ OAc), 139.0, 138.9, 138.1, 138.0, 137.7, 137.5, 137.1, 136.2, 133.3, 132.9 (10C, $C_q$ arom), 128.8-125.8 (42C, arom), 101.6, 100.9, 99.7, 99.5, 97.9 (5C, 5 x C-1), 85.0, 83.3, 81.8, 80.5, 80.3, 79.3, 77.9, 76.2, 75.3, 75.0, 74.2, 73.9, 72.8, 71.3, 71.0, 70.2, 69.5, 65.9 (20C, skeleton carbons), 60.8, 60.5, 59.4 (3C, 3 x O*C*$H_3$), 75.7, 74.8, 73.6, 73.3, 73.2 (8C, NAP*C*$H_2$, 7 x Bn*C*$H_2$), 68.9, 68.6, 68.4 (4C, 4 x C-6), 55.4 (1C, C-1-O*C*$H_3$), 21.1 (2C, 2 x *C*$H_3$ OAc), 16.8 (1C, *C*$H_3$ talose) ppm; MS (MALDI-TOF): *m*/*z* calcd for $C_{98}H_{114}NaO_{27}$, $[M+Na]^+$ 1746.95; found: 1747.10; elemental analysis calcd (%) for $C_{98}H_{114}O_{27}$ (1722.75); C, 68.28; H, 6.67 found: C, 68.38; H, 6.73.

**Methyl (6-*O*-benzyl-2,3,4-tri-*O*-methyl-α-D-glucopyranosyl)-(1→4)-[2,3-di-*O*-acetyl-6-*O*-(2-naphtyl)methyl-β-D-glucopyranosyl]-(1→4)-(2,3,6-tri-*O*-benzyl-α-D-glucopyranosyl)-(1→4)-(2,3-di-*O*-acetyl-6-deoxy-α-L-talopyranosyl)-(1→4)-2,3,6-tri-*O*-benzyl-α-D-glucopyranoside (28)**

**Method I.**: To a solution of compound **15** (100 mg, 0.089 mmol) and compound **25**[5] (105 mg, 0.133 mmol, 1.5 equiv.) in dry $CH_2Cl_2$ (5.0 mL), 4 Å molecular sieves (0.5 g) were added. After stirring for 30 min at room temperature, the mixture was cooled to −40 °C and a solution of NIS (45 mg, 0.199 mmol, 1.5 equiv. for the donor) and TMSOTf (11 μL, 0.060 mmol, 0.3 equiv.) in dry THF (580 μL) was added. Allowed to warm up to +10 °C the mixture was stirred for 5 h at that temperature. When the TLC analysis (1:1 *n*-hexane/EtOAc) showed complete consumption of the donor, the reaction mixture was neutralized with $Et_3N$

(50 μL), diluted with $CH_2Cl_2$ (75 mL), and filtered. The filtrate was washed with an aqueous solution of $Na_2S_2O_3$ (10%, 2 x 15 mL), a saturated aqueous solution of $NaHCO_3$ (2 x 15 mL), and water (2 x 15 mL), dried, and concentrated. The crude product was purified by column chromatography on silica gel (55:45 *n*-hexane/EtOAc) to give compound **28** (84 mg, 52%) as a colourless syrup.

**Method II.**: To a solution of compound **15** (1.00 g, 0.887 mmol) and compound **25**[5] (1.053 g, 1.331 mmol, 1.5 equiv.) in dry $CH_2Cl_2$ (34 mL), 4 Å molecular sieves (1.5 g) was added. After stirring for 30 min at room temperature, the mixture was cooled to −20 °C and a solution of NIS (449 mg, 1.997 mmol, 1.5 equiv. for the donor) and TfOH (53 μL, 0.599 mmol, 0.3 equiv.) in dry THF (810 μL) was added. Allowed to warm up to +15 °C the mixture was stirred for 3.5 h at that temperature. When the TLC analysis (1:1 *n*-hexane/EtOAc) showed complete consumption of the donor, the reaction mixture was neutralized with $Et_3N$ (100 μL), diluted with $CH_2Cl_2$ (250 mL), and filtered. The filtrate was washed with an aqueous solution of $Na_2S_2O_3$ (10%, 2 x 35 mL), a saturated aqueous solution of $NaHCO_3$ (2 x 35 mL) and water (2 x 35 mL), dried, and concentrated. The crude product was purified by column chromatography on silica gel (55:45 *n*-hexane/EtOAc) to give compound **28** (1.440 g, 90%) as a colourless syrup.

**Method III.** To a solution of compound **27** (50 mg, 0.029 mmol) in dry pyridine (1.0 mL) $Ac_2O$ (0.5 mL) was added and the mixture was stirred for 24 h at room temperature. After the reaction had been completed, the mixture was concentrated. The residue was purified by column chromatography on silica gel (1:1 *n*-hexane/EtOAc) to give compound **28** (38 mg, 74%) as a colourless syrup. $[\alpha]_D$ +16.3 (*c* 0.16, $CHCl_3$); $R_f$ 0.49 (1:1 *n*-hexane/EtOAc); $^1H$ NMR (400 MHz, $CDCl_3$) $\delta$ = 7.80-7.10 (m, 42H, arom), 5.24-4.26 (m, 25H, 5 x H-1, H-2-E, H-3-E, H-2-G, H-3-G, NAPC$H_2$, 7 x BnC$H_2$), 5.18-3.03 (m, 24H), 3.58, 3.41, 3.40, 3.35 (4 x s, 12H, 4 x OC$H_3$), 2.00, 1.90, 1.83, 1.81 (4 x s, 12H, 4 x C$H_3$ OAc), 0.81 (d, $J$ = 6.1 Hz, 3H, C$H_3$ talose) ppm; $^{13}C$ NMR (100 MHz, $CDCl_3$) $\delta$ = 171.1, 170.3, 170.0, 169.4 (4C, 4 x *C*O OAc), 139.5, 139.0, 138.4, 138.1, 138.0, 137.6, 136.1, 133.3, 132.9 (10C, $C_q$ arom), 128.8-125.7 (42C, arom), 99.9, 98.1, 98.0, 97.8 (5C, 5 x C-1), 83.2, 81.8, 80.5, 80.1, 79.7, 79.3, 77.0, 75.2, 75.1, 74.9, 74.1, 72.8, 71.2, 70.9, 70.1, 68.6, 68.1, 67.6, 66.9 (20C, skeleton carbons), 75.8, 73.7, 73.4, 73.3, 73.1 (8C, NAP*C*$H_2$, 7 x BnC$H_2$), 68.8, 68.4, 68.3, 67.6 (4C, 4 x C-6), 60.7, 60.4, 59.2 (3C, 3 x O*C*$H_3$), 55.3 (1C, C-1-H-O*C*$H_3$), 21.1, 21.0, 20.6 (4C, 4 x *C*$H_3$ OAc), 16.3 (1C, *C*$H_3$ talose) ppm; MS (MALDI-TOF): *m/z* calcd for $C_{102}H_{118}NaO_{29}$, $[M+Na]^+$ 1831.03; found: 1831.33; elemental analysis calcd (%) for $C_{102}H_{118}O_{29}$ (1806.78); C, 67.76; H, 6.58 found: C, 67.82; H, 6.64.

**Methyl (6-*O*-benzyl-2,3,4-tri-*O*-methyl-α-D-glucopyranosyl)-(1→4)-(2,3-di-*O*-acetyl-β-D-glucopyranosyl)-(1→4)-(2,3,6-tri-*O*-benzyl-α-D-glucopyranosyl)-(1→4)-(2,3-di-*O*-acetyl-6-deoxy-α-L-talopyranosyl)-(1→4)-2,3,6-tri-*O*-benzyl-α-D-glucopyranoside (29)**

To a vigorously stirred solution of compound **28** (460 mg, 0.254 mmol) in $CH_2Cl_2$ (3.9 mL) and $H_2O$ (440 μL), DDQ (87 mg, 0.381 mmol, 1.5 equiv.) was added. After 45 min the mixture was diluted with $CH_2Cl_2$ (150 mL) and washed with a saturated aqueous solution of $NaHCO_3$ (2 x 35 mL) and $H_2O$ (2 x 35 mL), dried and concentrated. The crude product was purified by silica gel chromatography (1:1 *n*-hexane/EtOAc) to give compound **29** (275 mg, 65%) as a colourless syrup. $[\alpha]_D$ +14.4 (*c* 0.09, $CHCl_3$); $R_f$ 0.28 (1:1 *n*-hexane/EtOAc); $^1$H NMR (400 MHz, $CDCl_3$) $\delta$ = 7.42-7.21 (m, 35H, arom), 5.19 (s, 1H, H-3-G), 5.10-4.35 (m, 23H, 5 x H-1, H-2-E, H-3-E, H-2-G, H-3-G, 7 x Bn$CH_2$), 4.07 (dd, *J* = 5.5 Hz, *J* = 12.1 Hz, 1H, H-5-G), 3.90-3.82 (m, 5H, H-3-H, H-4-H, H-3-F, H-4-F, H-5-F), 3.73-3.67 (m, 5H, H-4-E, H-4-G, H-5-H, H-6a-F, H-6a-H), 3.63-3.57 (m, 6H, H-2-H, H-5-D, H-6a,b-D, H-6a-E, H-6b-F), 3.589 (s, 3H, C-3-D-O$CH_3$), 3.50 (d, *J* = 10.6 Hz, 1H, H-6b-H), 3.45 (s, 3H, C-4-D-O$CH_3$), 3.44 (s, 3H, C-2-D-O$CH_3$), 3.41-3.36 (m, 3H, H-2-F, H-3-D, H-6b-E), 3.35 (s, 3H, C-1-H-O$CH_3$), 3.15 (t, *J* = 9.2 Hz, 1H, H-4-D), 3.10-3.04 (m, 2H, H-2-D, H-5-E), 2.00, 1.95, 1.86, 1.79 (4 x s, 12H, 4 x $CH_3$ OAc), 0.80 (d, *J* = 6.2 Hz, 3H, $CH_3$ talose) ppm; $^{13}$C NMR (100 MHz, $CDCl_3$) $\delta$ = 171.1, 170.3, 169.9, 169.4 (4C, 4 x *C*O OAc), 139.2, 139.1, 138.4, 138.1, 138.0, 137.5 (7C, $C_q$ arom), 128.8-127.2 (35C, arom), 99.8 (1C, C-1-E), 98.2 (2C, C-1-D, C-1-F), 98.0 (1C, C-1-H), 97.3 (1C, C-1-G), 83.5 (1C, C-3-D), 81.6 (1C, C-2-D), 80.5 (1C, C-2-H), 80.2 (1C, C-3-H), 79.8 (1C, C-3-F), 79.5 (1C, C-2-F), 79.4 (1C, C-4-D), 77.0 (1C, C-4-F), 75.3 (2C, C-3-E, C-4-E), 75.0 (1C, C-4-G), 74.8 (1C, C-5-E), 74.2 (1C, C-4-H), 72.7 (1C, C-2-E), 71.4 (1C, C-5-D), 70.9 (1C, C-5-E), 70.1 (1C, C-5-H), 68.6 (1C, C-2-G), 68.1 (1C, C-3-G), 66.8 (1C, C-5-G), 75.8, 75.2, 73.8, 73.6, 73.5, 73.4, 73.2 (7C, 7 x Bn$CH_2$), 68.6, 68.4 (2C, C-6-D, C-6-F), 67.3 (1C, C-6-H), 61.4 (1C, C-6-E), 60.8 (1C, C-3-D-O$CH_3$), 60.5 (1C, C-4-D-O$CH_3$), 59.6 (1C, C-2-D-O$CH_3$), 55.3 (1C, C-1-H-O$CH_3$), 21.2, 21.1, 21.0, 20.6 (4C, 4 x $CH_3$ OAc), 16.3 (1C, $CH_3$ talose) ppm; MS (MALDI-TOF): *m*/*z* calcd for $C_{91}H_{110}NaO_{29}$, $[M+Na]^+$ 1689.70; found: 1690.24; elemental analysis calcd (%) for $C_{91}H_{110}O_{29}$ (1666.71); C, 65.53; H, 6.65 found: C, 65.67; H, 6.71.

**Methyl (6-*O*-benzyl-2,3,4-tri-*O*-methyl-α-D-glucopyranosyl)-(1→4)-[sodium-(2,3-di-*O*-acetyl-β-D-glucopyranosyl)-uronate]-(1→4)-(2,3,6-tri-*O*-benzyl-α-D-glucopyranosyl)-**

**(1→4)-(2,3-di-*O*-acetyl-6-deoxy-α-L-talopyranosyl)-(1→4)-2,3,6-tri-*O*-benzyl-α-D-glucopyranoside (30)**

To a vigorously stirring solution of compound **29** (255 mg, 0.153 mmol) in $CH_2Cl_2$ (4.0 mL) and water (2.0 mL), TEMPO (4.0 mg, 0.027 mmol) and BAIB (148 mg, 0.459 mmol) were added. After stirring for 48 h at room temperature, the reaction mixture was quenched by addition of an aqueous solution of $Na_2S_2O_3$ (10%, 14 mL). The phases were separated, and the aqueous layer was extracted with $CH_2Cl_2$ (3 x 20 mL). The combined organic layers were dried and concentrated. The crude product was purified by column chromatography on silica gel (55:45 *n*-hexane/acetone) to give compound **30** (170 mg, 65%) as a colourless syrup. $[\alpha]_D$ +10.0 (*c* 0.13, $CHCl_3$); $R_f$ 0.31 (6:4 *n*-hexane/acetone); $^1H$ NMR (400 MHz, $CDCl_3$+$CD_3OD$) $\delta$ = 7.46-7.22 (m, 35H, arom), 5.16-4.42 (m, 23H, 5 x H-1, H-2-E, H-3-E, H-2-G, H-3-G, 7 x BnC$H_2$), 4.11-4.04 (m, 2H), 3.90-3.26 (m, 19H), 3.59, 3.45, 3.44, 3.35 (4 x s, 12H, 4 x OC$H_3$), 3.06 (dd, *J* = 3.4 Hz, *J* = 9.7 Hz, 1H, H-2-D), 2.02, 1.91, 1.81, 1.80 (4 x s, 12H, 4 x C$H_3$ OAc), 0.79 (d, *J* = 6.4 Hz, 3H, C$H_3$ talose) ppm; $^{13}C$ NMR (100 MHz, $CDCl_3$+$CD_3OD$) $\delta$ = 172.3, 171.6, 171.2, 170.4 (4C, 4 x *C*O OAc), 139.8, 139.7, 139.3, 139.1, 138.9, 138.8, 138.5 (7C, $C_q$ arom), 129.6-128.1 (35C, arom), 101.0, 98.9, 98.8, 98.7, 98.1 (5C, 5 x C-1), 84.1, 82.5, 81.6, 80.9, 80.8, 80.2, 79.8, 78.2, 77.3, 76.1, 75.3, 74.6, 73.1, 72.0, 71.9, 71.1, 69.5, 69.0, 67.7, 66.8 (20C, skeleton carbons), 76.6, 76.3, 74.4, 74.3, 73.9 (7C, 7 x BnC$H_2$), 69.2, 68.9, 68.4 (3C, 3 x C-6), 61.0, 60.7, 59.7 (3C, 3 x O*C*$H_3$), 55.6 (1C, C-1-H-O*C*$H_3$), 21.4, 21.3, 21.1, 20.8 (4C, 4 x *C*$H_3$ OAc), 16.7 (1C, *C*$H_3$ talose) ppm; MS (MALDI-TOF): *m*/*z* calcd for $C_{91}H_{107}Na_2O_{30}$, $[M+Na]^+$ 1726.81; found: 1727.13; elemental analysis calcd (%) for $C_{91}H_{107}O_{30}$ (1702.67); C, 64.15; H, 6.33 found: C, 64.43; H, 6.41.

**Methyl (2,3,4-tri-*O*-methyl-α-D-glucopyranosyl)-(1→4)-[sodium-(2,3-di-*O*-acetyl-β-D-glucopyranosyl)-uronate]-(1→4)-(α-D-glucopyranosyl)-(1→4)-(2,3-di-*O*-acetyl-6-deoxy-α-L-talopyranosyl)-(1→4)-α-D-glucopyranoside (31)**

Compound **30** (153 mg, 0.090 mmol) was dissolved in 96% EtOH / AcOH (19:1, 7.0 mL), and Pd/C (10%, 95 mg) was added and stirred in an autoclave under a $H_2$ atmosphere (at 10 bar) for 24 h. The catalyst was filtered off through a pad of Celite and the filtrate was concentrated under reduced pressure. The crude product was purified by column chromatography on silica gel (7:5:0.5 $CH_2Cl_2$/MeOH/$H_2O$) to give compound **31** (90 mg, 94%) as a colourless syrup. $[\alpha]_D$ +55.3 (*c* 0.15, MeOH); $R_f$ 0.59 (7:5:0.5 $CH_2Cl_2$/MeOH/$H_2O$); $^1H$ NMR (400 MHz, $D_2O$) $\delta$ = 5.34 (d, *J* = 3.9 Hz, 1H), 5.33-5.31 (m, 1H), 5.29 (t, *J* = 3.1 Hz, 1H), 5.21 (d, *J* = 3.8 Hz, 1H), 5.09 (s, 1H), 4.99 (s, 1H), 4.89 (d, *J* = 5.1 Hz, 1H), 4.80 (d, *J* =

3.8 Hz, 1H), 4.54 (q, *J* = 6.4 Hz, 1H), 4.11-3.50 (m, 20H), 3.60, 3.53, 3.46, 3.40 (4 x s, 12H, 4 x OC*H*3), 3.29-3.23 (m, 2H), 2.17, 2.14, 2.11, 2.10 (4 x s, 12H, 4 x C*H*3 OAc), 1.26 (d, *J* = 6.5 Hz, 3H, C*H*3 talose) ppm; $^{13}$C NMR (100 MHz, $D_2O$) $\delta$ = 173.8, 173.3, 172.8, 172.6, 172.4 (5C, *C*OONa, 4 x *C*O OAc), 99.4, 99.1, 98.4, 97.8, 97.2 (5C, 5 x C-1), 82.4, 80.1, 78.3, 77.1, 76.3, 75.2, 74.6, 72.8, 72.5, 71.8, 71.7, 71.5, 71.3, 70.7, 70.6, 69.0, 68.5, 67.4 (20C, skeleton carbons), 60.1, 59.5, 59.4 (3C, 3 x O*C*H3), 59.9, 59.7, 59.5 (3C, 3 x C-6), 55.0 (1C, C-1-H-O*C*H3), 20.5, 20.4, 20.3, 20.1 (4C, 4 x *C*H3 OAc), 16.1 (1C, *C*H3 talose) ppm; MS (MALDI-TOF): *m*/*z* calcd for $C_{42}H_{65}Na_2O_{30}$, [M+Na]$^+$ 1095.34; found: 1095.45; elemental analysis calcd (%) for $C_{42}H_{65}NaO_{30}$ (1072.35); C, 47.02; H, 6.11 found: C, 47.21; H, 6.19.

**Methyl (6-*O*-benzyl-2,3,4-tri-*O*-methyl-α-D-glucopyranosyl)-(1→4)-[6-*O*-(2-naphtyl)methyl-β-D-glucopyranosyl]-(1→4)-(2,3,6-tri-*O*-benzyl-α-D-glucopyranosyl)-(1→4)-(6-deoxy-α-L-talopyranosyl)-(1→4)-2,3,6-tri-*O*-benzyl-α-D-glucopyranoside (32)**

To a stirred solution of compound **28** (900 mg, 0.497 mmol) in MeOH (5.0 mL) NaOMe (50 mg, 0.926 mmol) was added. After 24 h the mixture was neutralized with Amberlite IR-120 H$^+$ ion exchanged resin, filtered, washed with MeOH and concentrated. The crude product was purified by silica gel chromatography (6:4 *n*-hexane/EtOAc) to give compound **32** (717 mg, 88%) as a colourless syrup. $[\alpha]_D$ +36.7 (*c* 0.18, $CHCl_3$); $R_f$ 0.36 (6:4 *n*-hexane/acetone); $^1$H NMR (400 MHz, $CDCl_3$) $\delta$ = 7.80-7.16 (m, 42H, arom), 5.13-4.29 (m, 21H, 5 x H-1, NAPC*H*2, 7 x BnC*H*2), 4.02-3.05 (m, 28H), 3.62, 3.55, 3.43, 3.35 (4 x s, 12H, 4 x OC*H*3), 0.76 (d, *J* = 6.4 Hz, 3H, C*H*3 talose) ppm; $^{13}$C NMR (100 MHz, $CDCl_3$) $\delta$ = 139.0, 138.8, 138.0, 137.9, 137.7, 137.6, 137.0, 136.3, 133.3, 132.9 (10C, $C_q$ arom), 128.5-125.7 (42C, arom), 102.6, 101.5, 100.9, 100.5, 97.9 (5C, 5 x C-1), 85.1, 84.0, 82.8, 82.1, 81.3, 80.4, 80.2, 79.4, 78.3, 76.4, 76.0, 74.8, 73.8, 71.3, 71.0, 70.8, 70.2, 66.9, 65.8 (20C, skeleton carbons), 75.6, 75.3, 74.7, 73.6, 73.5, 73.3 (8C, NAP*C*H2, 7 x BnC*H*2), 69.1, 68.7, 68.3, 67.9 (4C, 4 x C-6), 60.8, 60.5, 60.3 (3C, 3 x O*C*H3), 55.3 (1C, C-1-H-O*C*H3), 16.8 (1C, *C*H3 talose) ppm; MS (MALDI-TOF): *m*/*z* calcd for $C_{94}H_{110}NaO_{25}$, [M+Na]$^+$ 1662.88; found: 1663.18; elemental analysis calcd (%) for $C_{94}H_{110}O_{25}$ (1639.89); C, 68.85; H, 6.76 found: C, 68.97; H, 6.73.

**Methyl (6-*O*-benzyl-2,3,4-tri-*O*-methyl-α-D-glucopyranosyl)-(1→4)-[2,3-di-*O*-methyl-6-*O*-(2-naphtyl)methyl-β-D-glucopyranosyl]-(1→4)-(2,3,6-tri-*O*-benzyl-α-D-glucopyranosyl)-(1→4)-(2,3-di-*O*-methyl-6-deoxy-α-L-talopyranosyl)-(1→4)-2,3,6-tri-*O*-benzyl-α-D-glucopyranoside (33)**

To a solution of compound **32** (717 mg, 0.437 mmol) in dry DMF (2.0 mL) was slowly added NaH (60%, 84 mg, 2.099 mmol, 1.2 equiv./OH) at 0 °C. After stirring for 30 min at 0 °C, MeI (136 µL, 2.186 mmol, 1.25 equiv./OH) was added. When complete conversion of the starting material into a main spot had been observed by TLC analysis (24 h at room temperature), $CH_3OH$ (5.0 mL) was added. The reaction mixture was stirred for 5 min and the solvents were evaporated. The residue was dissolved in $CH_2Cl_2$ (125 mL) and washed with $H_2O$ (2 x 35 mL), the organic layer was filtered, dried and evaporated. The crude product was purified by column chromatography (1:1 *n*-hexane/EtOAc) to give compound **33** (600 mg, 81%) as a colourless syrup. $[\alpha]_D$ +47.3 (*c* 0.15, $CHCl_3$); $R_f$ 0.40 (1:1 *n*-hexane/EtOAc); $^1H$ NMR (400 MHz, $CDCl_3$) $\delta$ = 7.79-7.09 (m, 42H, arom), 5.60 (d, *J* = 3.7 Hz, 1H), 5.41 (s, 1H), 5.06-4.32 (m, 19H, 3 x H-1, NAPC$H_2$, 7 x BnC$H_2$), 4.14-3.14 (m, 27H), 3.60, 3.57, 3.54, 3.46, 3.43, 3.38, 3.37, 3.25 (8 x s, 24H, 8 x OC$H_3$), 3.02 (t, *J* = 8.5 Hz, 1H), 0.98 (d, *J* = 6.1 Hz, 3H, C$H_3$ talose) ppm; $^{13}C$ NMR (100 MHz, $CDCl_3$) $\delta$ = 139.9, 139.1, 138.2, 138.1, 137.9, 136.5, 133.3, 132.9 (10C, $C_q$ arom), 128.6-125.6 (42C, arom), 102.7, 98.1, 96.8, 96.2 (5C, 5 x C-1), 86.6, 84.7, 83.5, 81.9, 80.3, 80.2, 79.4, 79.3, 78.7, 78.6, 77.1, 75.0, 74.7, 72.2, 70.8, 70.6, 70.3 (20C, skeleton carbons), 75.7, 74.8, 73.5, 73.4, 73.2 (8C, NAP*C*$H_2$, 7 x BnC$H_2$), 69.3, 68.8, 68.1, 68.0 (4C, 4 x C-6), 60.8, 60.4, 60.3, 60.2, 59.6, 59.5, 58.8 (7C, 7 x O*C*$H_3$), 55.3 (1C, C-1-H-O*C*$H_3$), 14.3 (1C, *C*$H_3$ talose) ppm; MS (MALDI-TOF): *m*/*z* calcd for $C_{98}H_{118}NaO_{25}$, $[M+Na]^+$ 1718.99; found: 1719.30; elemental analysis calcd (%) for $C_{98}H_{118}O_{25}$ (1694.80); C, 69.40; H, 7.01 found: C, 69.67; H, 7.09.

**Methyl (6-*O*-benzyl-2,3,4-tri-*O*-methyl-α-D-glucopyranosyl)-(1→4)-(2,3-di-*O*-methyl-β-D-glucopyranosyl)-(1→4)-(2,3,6-tri-*O*-benzyl-α-D-glucopyranosyl)-(1→4)-(2,3-di-*O*-methyl-6-deoxy-α-L-talopyranosyl)-(1→4)-2,3,6-tri-*O*-benzyl-α-D-glucopyranoside (34)**

To a vigorously stirred solution of compound **33** (576 mg, 0.339 mmol) in $CH_2Cl_2$ (5.2 mL) and $H_2O$ (650 µL), DDQ (116 mg, 0.509 mmol, 1.5 equiv.) was added. After 40 min the mixture was diluted with $CH_2Cl_2$ (150 mL) and washed with a saturated aqueous solution of $NaHCO_3$ (2 x 35 mL) and $H_2O$ (2 x 75 mL), dried and concentrated. The crude product was purified by silica gel chromatography (35:65 *n*-hexane/EtOAc) to give compound **34** (350 mg, 65%) as a colourless syrup. $[\alpha]_D$ +52.9 (*c* 0.15, $CHCl_3$); $R_f$ 0.41 (6:4 *n*-hexane/acetone); $^1H$ NMR (400 MHz, $CDCl_3$) $\delta$ = 7.36-7.14 (m, 35H, arom), 5.52 (d, *J* = 3.7 Hz, 1H), 5.45 (s, 1H), 5.07 (d, *J* = 3.1 Hz, 1H), 5.01-4.51(m, 16H, 2 x H-1, 7 x BnC$H_2$), 4.26 (d, *J* = 7.9 Hz, 1H), 4.01 (dd, *J* = 2.8 Hz, *J* = 6.6 Hz, 1H), 3.90 (t, *J* = 9.2 Hz, 1H), 3.85-3.10 (m, 24H), 3.62, 3.58, 3.54, 3.46, 3.45, 3.44, 3.37, 3.25 (8 x s, 24H, 8 x OC$H_3$), 2.94 (dd, *J* = 8.3 Hz, *J* = 8.8

Hz, 1H), 2.03 (s, 1H, C-6-E-O*H*), 0.96 (d, *J* = 6.3 Hz, 3H, C*H*$_3$ talose) ppm; $^{13}$C NMR (100 MHz, $CDCl_3$) $\delta$ = 139.5, 139.0, 138.2, 138.1, 138.0, 137.9 (7C, $C_q$ arom), 128.5-127.3 (35C, arom), 102.6, 98.0, 96.8, 96.7, 96.0 (5C, 5 x C-1), 86.4, 84.8, 83.5, 81.8, 80.3, 80.2, 79.5, 79.2, 78.7, 78.6, 77.4, 74.8, 74.2, 72.9, 70.9, 70.5, 70.3, 68.1 (20C, skeleton carbons), 75.7, 75.2, 73.5, 73.4, 71.1 (7C, 7 x BnC*H*$_2$), 68.7, 68.4, 67.8 (3C, 3 x C-6), 61.9 (1C, C-6-E), 60.8, 60.4, 60.2, 59.9, 59.6, 58.8, 57.8 (7C, 7 x O*C*H$_3$), 55.2 (1C, C-1-H-O*C*H$_3$), 16.2 (1C, *C*H$_3$ talose) ppm; MS (MALDI-TOF): *m*/*z* calcd for $C_{87}H_{110}NaO_{25}$, $[M+Na]^+$ 1578.80; found: 1578.21; elemental analysis calcd (%) for $C_{87}H_{110}O_{25}$ (1554.73); C, 67.16; H, 7.13 found: C, 67.04; H, 7.21.

**Methyl (6-*O*-benzyl-2,3,4-tri-*O*-methyl-α-D-glucopyranosyl)-(1→4)-[sodium-(2,3-di-*O*-methyl-β-D-glucopyranosyl)-uronate]-(1→4)-(2,3,6-tri-*O*-benzyl-α-D-glucopyranosyl)-(1→4)-(2,3-di-*O*-methyl-6-deoxy-α-L-talopyranosyl)-(1→4)-2,3,6-tri-*O*-benzyl-α-D-glucopyranoside (35)**

To a vigorously stirring solution of compound **34** (340 mg, 0.218 mmol) in $CH_2Cl_2$ (5.5 mL) and water (2.8 mL), TEMPO (6.0 mg, 0.039 mmol) and BAIB (211 mg, 0.655 mmol) were added. After stirring for 48 h at room temperature, the reaction mixture was quenched by addition of an aqueous solution of $Na_2S_2O_3$ (10%, 20 mL). The phases were separated, and the aqueous layer was extracted with $CH_2Cl_2$ (3 x 25 mL). The combined organic layers were dried and concentrated. The crude product was purified by column chromatography on silica gel (95:5 $CH_2Cl_2$/MeOH) to give compound **35** (273 mg, 78%) as a colourless syrup. $[\alpha]_D$ +10.9 (*c* 0.42, $CHCl_3$); $R_f$ 0.29 (95:5 $CH_2Cl_2$/MeOH); $^1$H NMR (400 MHz, $CD_3OD$) $\delta$ = 7.30-7.04 (m, 35H, arom), 5.41 (d, *J* = 2.4 Hz, 1H), 5.36 (d, *J* = 0.7 Hz, 1H), 5.33 (d, *J* = 3.2 Hz, 1H), 4.94 (s, 1H), 4.89-4.30 (m, 14H, 7 x BnC*H*$_2$), 4.31 (d, *J* = 7.8 Hz, 1H), 3.94 (d, *J* = 6.0 Hz, 1H), 3.83-2.99 (m, 24H), 3.48, 3.42, 3.34, 3.32, 3.25, 3.04 (6 x s, 24H, 8 x OC*H*$_3$), 2.87-2.83 (m, 1H), 0.73 (d, *J* = 6.1 Hz, 3H, C*H*$_3$ talose) ppm; $^{13}$C NMR (100 MHz, $CD_3OD$) $\delta$ = 172.6 (1C, *C*OONa), 140.4, 140.2, 139.6, 139.5, 139.4, 139.3 (7C, $C_q$ arom), 129.8-128.2 (35C, arom), 104.0, 98.9, 98.2, 97.8, 96.7 (5C, 5 x C-1), 86.8, 85.4, 84.4, 83.1, 82.0, 81.3, 80.4, 80.3, 80.2, 80.0, 78.6, 76.5, 75.2, 71.8, 71.7, 68.9 (20C, skeleton carbons), 76.6, 76.3, 74.5, 74.4, 74.0, 72.6 (7C, 7 x BnC*H*$_2$), 70.0, 69.4, 68.9 (3C, 3 x C-6), 61.0, 60.8, 60.7, 59.7, 59.3, 57.9 (7C, 7 x O*C*H$_3$), 55.6 (1C, C-1-H-O*C*H$_3$), 17.1 (1C, *C*H$_3$ talose) ppm; MS (MALDI-TOF): *m*/*z* calcd for $C_{87}H_{107}Na_2O_{26}$, $[M+Na]^+$ 1613.68; found: 1614.29; elemental analysis calcd (%) for $C_{87}H_{107}NaO_{26}$ (1590.69); C, 65.65; H, 6.78 found: C, 65.79; H, 6.83.

**Methyl (2,3,4-tri-*O*-methyl-α-D-glucopyranosyl)-(1→4)-[sodium-(2,3-di-*O*-methyl-β-D-glucopyranosyl)-uronate]-(1→4)-(α-D-glucopyranosyl)-(1→4)-(2,3-di-*O*-methyl-6-deoxy-α-L-talopyranosyl)-(1→4)-α-D-glucopyranoside (36)**

Compound **35** (250 mg, 0.157 mmol) was dissolved in 96% EtOH / AcOH (19:1, 12.6 mL), and Pd/C (10%, 166 mg) was added and stirred in an autoclave under a $H_2$ atmosphere (at 10 bar) for 24 h. The catalyst was filtered off through a pad of Celite and the filtrate was concentrated under reduced pressure. The crude product was purified by column chromatography on silica gel (6:7:1 EtOAc/MeOH/$H_2O$) to give compound **36** (130 mg, 86%) as a colourless syrup. $[\alpha]_D$ +92.7 (*c* 0.11, $CHCl_3$); $R_f$ 0.43 (6:7:1 EtOAc/MeOH/$H_2O$); $^1H$ NMR (400 MHz, $D_2O$) $\delta$ = 5.38 (d, *J* = 3.6 Hz, 1H), 5.22 (d, *J* = 4.0 Hz, 1H), 5.05 (s, 1H), 4.74 (d, *J* = 3.6 Hz, 1H), 4.49 (d, *J* = 7.7 Hz, 1H), 4.30 (q, *J* = 6.4 Hz, 1H), 4.00 (s, 1H), 3.91 (dd, *J* = 4.8 Hz, *J* = 9.9 Hz, 1H), 3.85-3.40 (m, 20H), 3.56, 3.55, 3.53, 3.48, 3.46, 3.38, 3.37, 3.33 (8 x s, 24H, 8 x $OCH_3$), 3.25-3.16 (m, 3H), 1.17 (d, *J* = 6.4 Hz, 3H, $CH_3$ talose) ppm; $^{13}C$ NMR (100 MHz, $D_2O$) $\delta$ = 174.6 (1C, *C*OONa), 101.7, 99.1, 98.2, 97.6, 95.9 (5C, 5 x C-1), 85.6, 83.1, 81.6, 80.6, 78.6, 78.2, 77.3, 76.8, 76.3, 76.2, 74.5, 74.1, 71.9, 71.8, 71.7, 71.5, 70.6, 70.5, 67.4 (20C, skeleton carbons), 60.2, 59.9, 59.6 (3C, 3 x C-6), 60.3, 60.1, 59.8, 59.3, 58.9, 58.3, 56.2 (7C, 7 x $OCH_3$), 55.0 (1C, C-1-H-$OCH_3$), 16.5 (1C, $CH_3$ talose) ppm; MS (MALDI-TOF): *m*/*z* calcd for $C_{38}H_{65}Na_2O_{26}$, $[M+Na]^+$ 983.36; found: 983.17; elemental analysis calcd (%) for $C_{38}H_{65}NaO_{26}$ (960.37); C, 47.50; H, 6.82 found: C, 47.37; H, 6.93.

## Measurements of factor Xa inhibitory activity (Table S1)

| | Anti-Xa activity (U/mg) |
|---|---|
| Heparin | 1 |
| Idraparinux (**1**) | 1911 |
| Methylated pentasaccharide (**2**) | 0.3 |
| Partially acetylated pentasaccharide (**3**) | 0.3 |
| Partially methylated pentasaccharide (**4**) | 0.5 |

**Table S1.** Inhibitory activity of Heparin, reference compound idraparinux (**1**) and the recently prepared pentasaccharide **2**, **3** and **4** towards factor Xa

For biological investigation compound **1** was prepared in our laboratory. The anti-factor Xa activity of **2-4** was determined in vitro by Berichrom® Heparin chromogenic assay on a Siemens BCS-XP automated coagulometer (Siemens, Marburg, Germany), using pooled normal human plasma. In this spectrophotometric kinetic assay the FXa inhibitory activity of heparin/LMWH or pentasaccharides is determined by detecting the residual FXa activity in the presence of antithrombin and chromogenic substrate specific for FXa. The change in absorbance at 405 nm is proportional to the residual FXa activity. The lower residual FXa activity corresponds to higher heparin/LMWH or pentasaccharide concentration in the plasma. Pentasaccharides **2-4** were tested in at least 3 different pentasaccharide concentrations (final concentration range of 0.00-1000 μg/mL) using a pentasaccharide (fondaparinux/Arixtra) calibrator from Diagnostica Stago (Asnieres, France).

| **Concentration** (μg/mL) | **2** Activity (IU/mL) | **3** Activity (IU/mL) | **4** Activity (IU/mL) |
|---|---|---|---|
| 0 | 0 | 0 | 0 |
| 250 | 0.16 | 0.14 | 0.23 |
| 500 | 0.26 | 0.26 | 0.36 |
| 1000 | 0.35 | 0.32 | 0.50 |

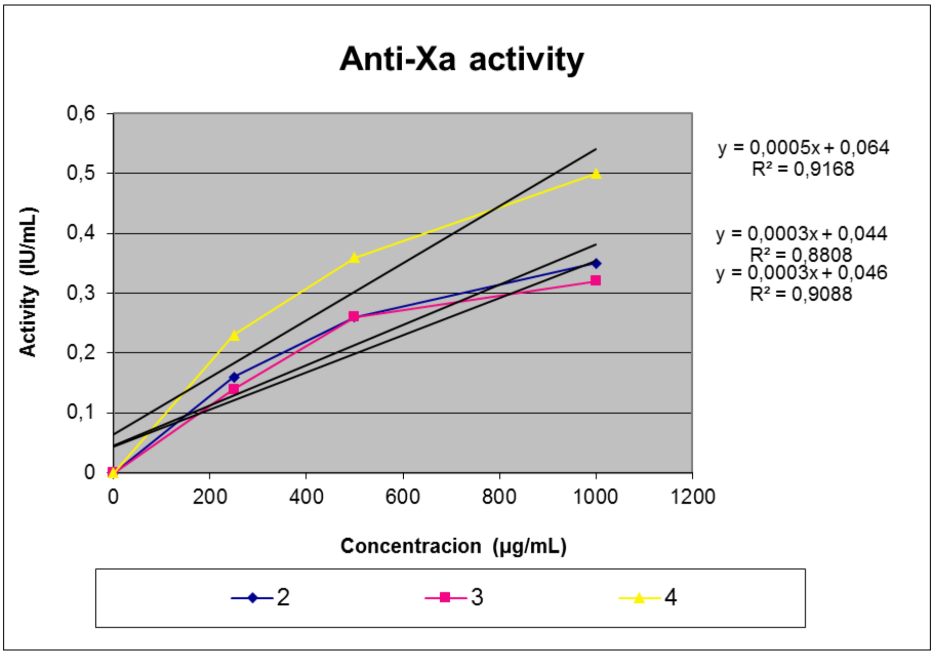

# References


1. Frihed, T. G.; Pedersen C. M.; Bols, M., Synthesis of All Eight Stereoisomeric 6-Deoxy-L-hexopyranosyl Donors – Trends in Using Stereoselective Reductions or Mitsunobu Epimerizations, *Eur. J. Org. Chem.* **2014**, 7924–7939.
2. Ek, M.; Garegg, P. J.; Hultberg, H.; Oscarson, S., Reductive Ring Openings of Carbohydrate Benzylidene Acetals Using Borane-Trimethylamine and Aluminium Chloride. Regioselectivity and Solvent Dependance, *J. Carbohydr. Chem.* **1983**, *2*, 305–311.; b) Debenham, S. D.; Toone, E. J., Regioselective reduction of 4,6-*O*-benzylidenes using triethylsilane and $BF_3{\cdot}Et_2O$, *Tetrahedron: Asymmetry*, **2000**, *11*, 385–387.
3. Herczeg, M.; Mező, E.; Eszenyi, D.; Antus, S.; Borbás, A., New synthesis of idraparinux, the non-glycosaminoglycan analogue of the antithrombin-binding domain of heparin, *Tetrahedron*, **2014**, *70*, 2919-2927.
4. Groneberg, R. D.; Miyazaki, T.; Stylianides, N. A.; Schulze, T. J.; Stahl, W.; Schreiner, E. P.; Suzuki, T.; Iwabuchi, Y.; Smith, A. L.; Nicolaou, K. C., Total synthesis of calicheamicin .gamma.1I. 1. Synthesis of the oligosaccharide fragment, *J. Am. Chem. Soc.*, **1993**, 115, 7593–7611.
5. Lázár, L.; Mező, E.; Herczeg, M.; Lipták, A.; Antus, S.; Borbás, A., Synthesis of the non-reducing end trisaccharide of the antithrombin-binding domain of heparin and its bioisosteric sulfonic acid analogues *Tetrahedron*, **2012**, *68*, 7386–7399.
6. Thiele, C. M.; Petzold, K.; Schleucher J., EASY ROESY: Reliable Cross-Peak Integration in Adiabatic Symmetrized ROESY, *Chem. Eur. J.* **2009**, *15*, 585–588.

## $^{1}H$ and $^{13}C$ NMR spectra of the new compounds

**$^{1}H$ and $^{13}C$ NMR spectra of compound 2:**

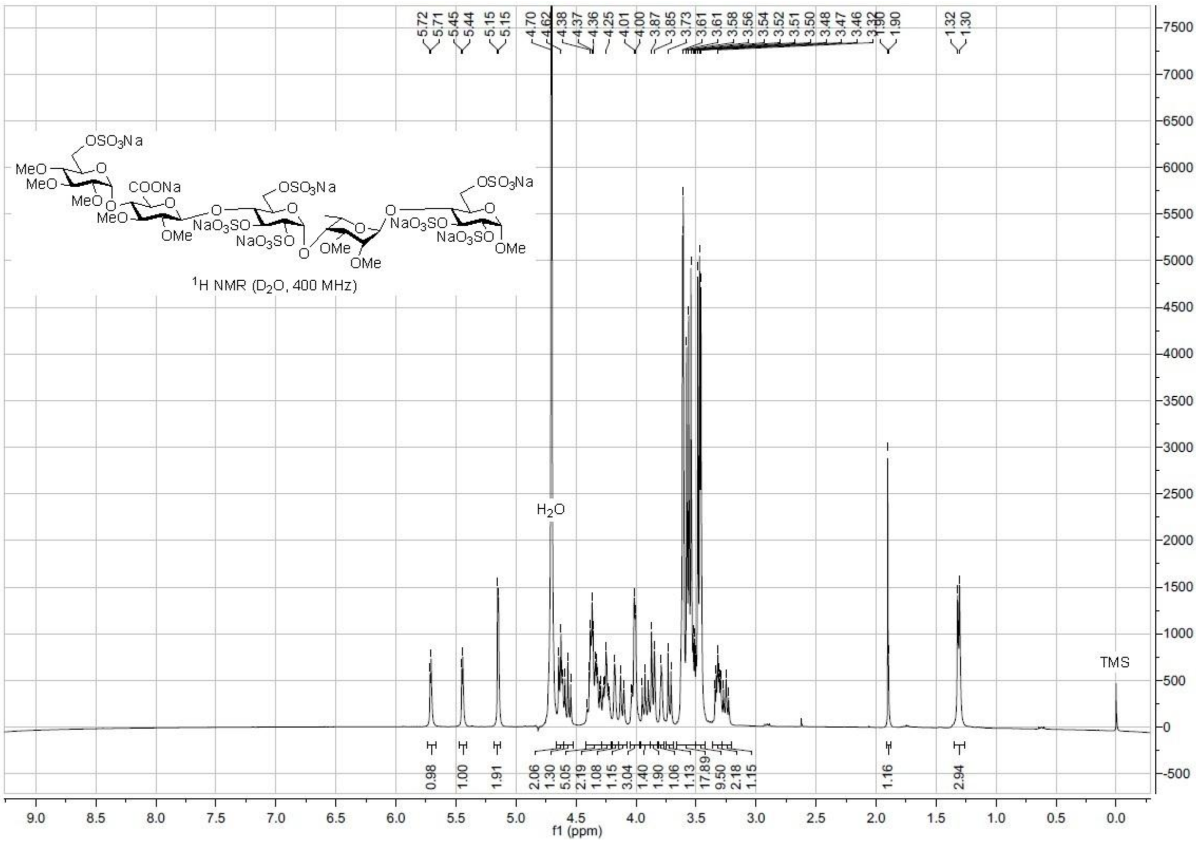


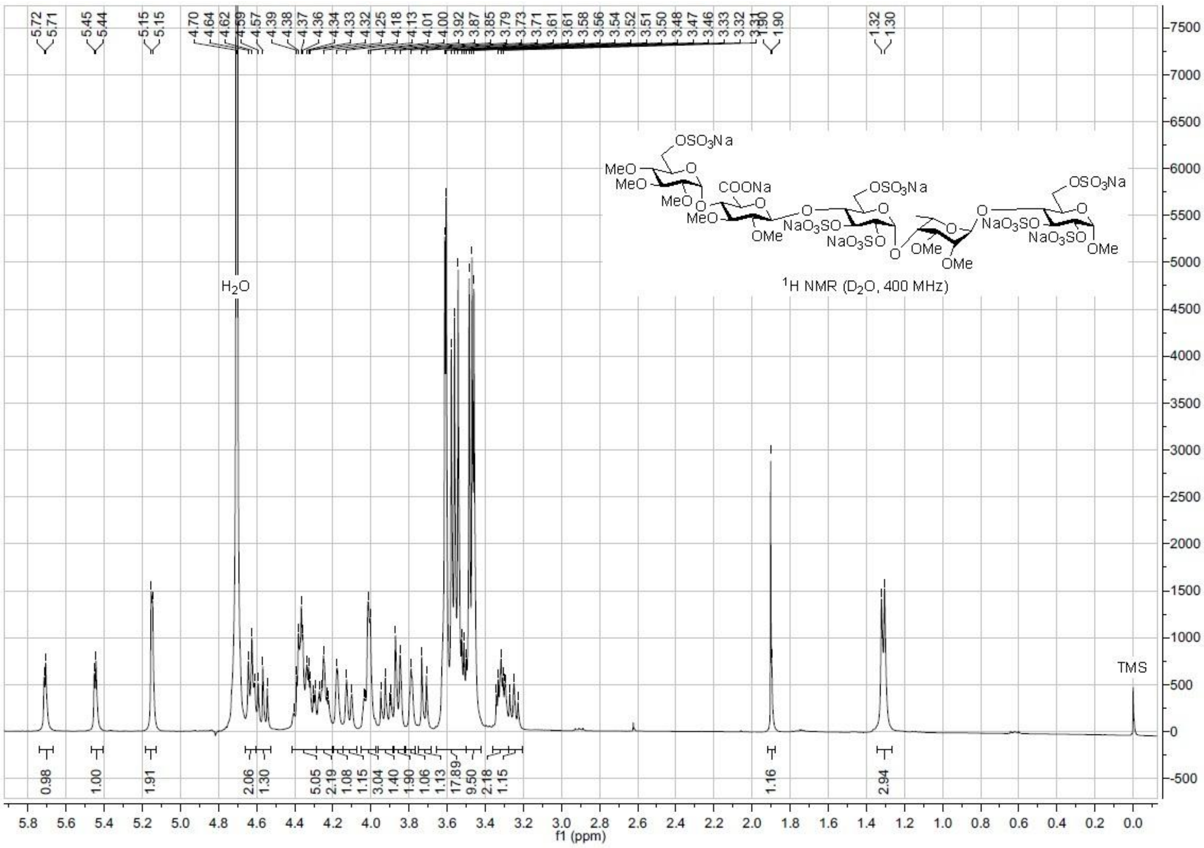

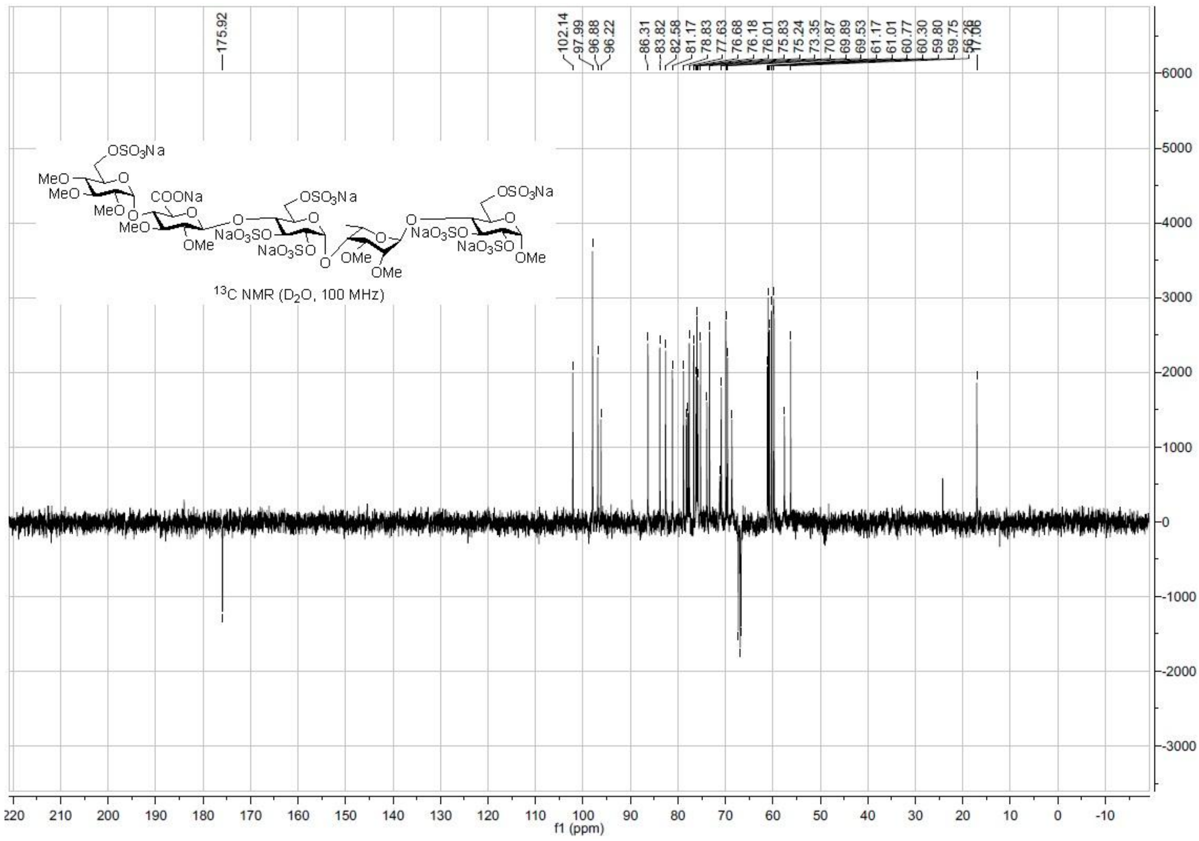
175.92
102.14
97.99
96.88
96.22
86.31
83.82
82.58
81.17
78.83
77.63
76.68
76.18
76.01
75.83
75.24
73.35
70.87
69.89
69.53
61.17
61.01
60.77
60.30
59.80
59.75
OSO3Na
MeO
MeO
MeO
MeO
COONa
OMe
NaO3SO
NaO3SO
OMe
OMe
OMe
13C NMR (D2O, 100 MHz)
f1 (ppm)

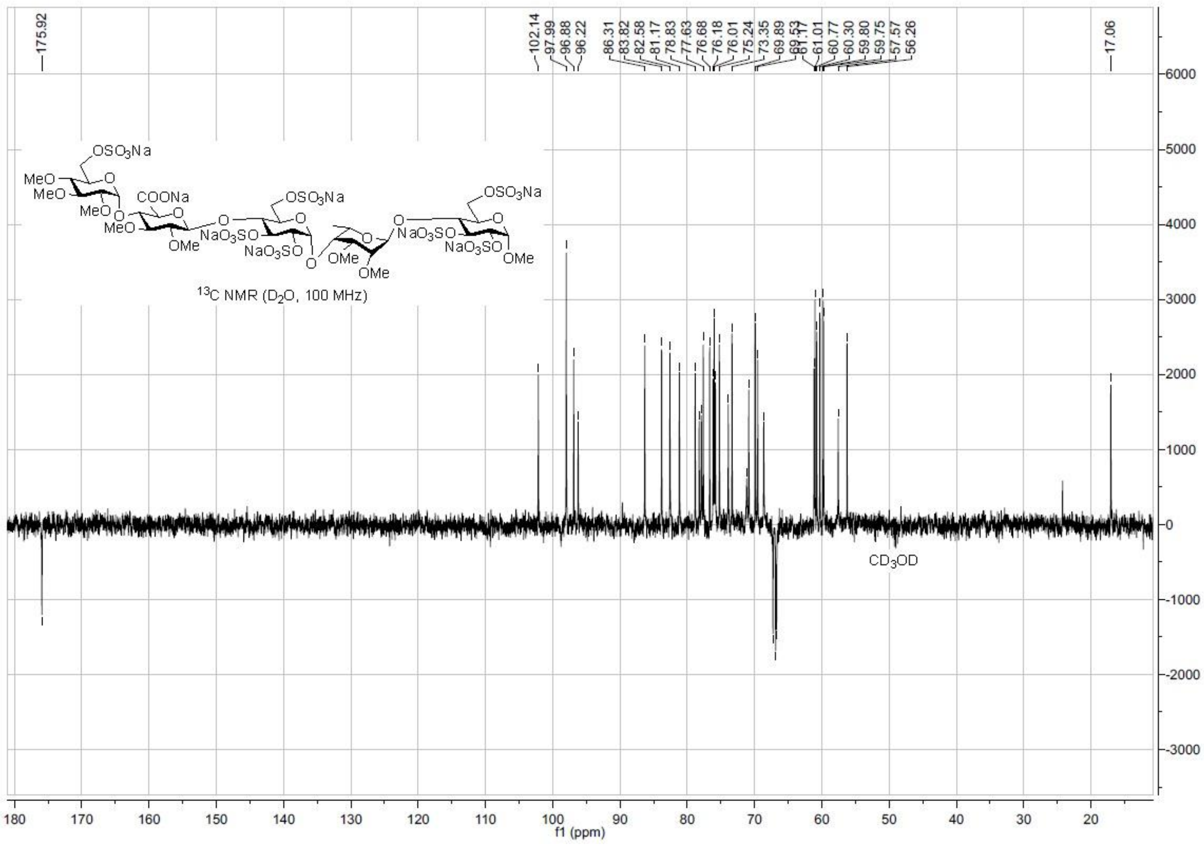
175.92
102.14
97.99
96.88
96.22
86.31
83.82
82.58
81.17
78.83
77.63
76.68
76.18
76.01
75.24
73.35
69.89
61.01
60.77
60.30
59.80
59.75
57.57
56.26
17.06
OSO3Na
MeO
MeO
MeO
MeO
COONa
OMe
NaO3SO
NaO3SO
OMe
OMe
OMe
13C NMR (D2O, 100 MHz)
CD3OD
f1 (ppm)

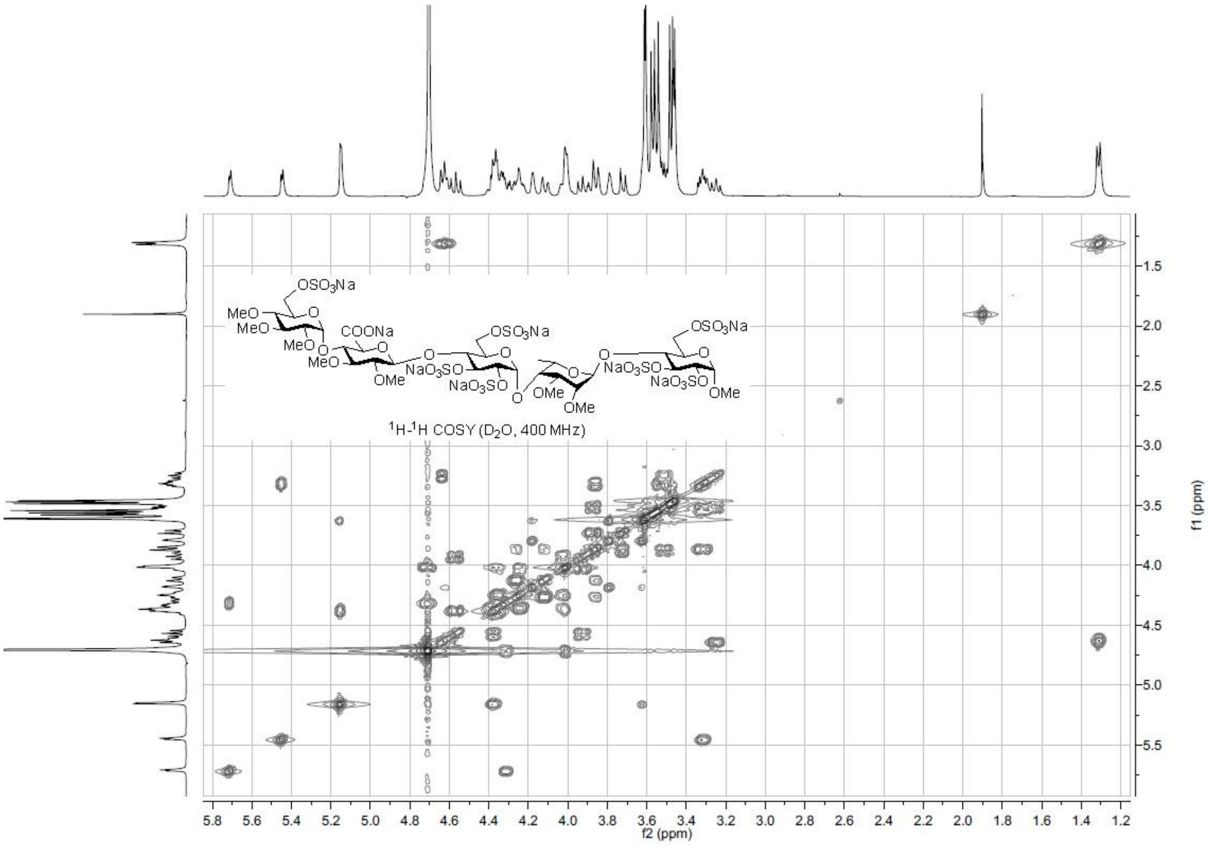
1H-1H COSY (D2O, 400 MHz)
f2 (ppm)
f1 (ppm)

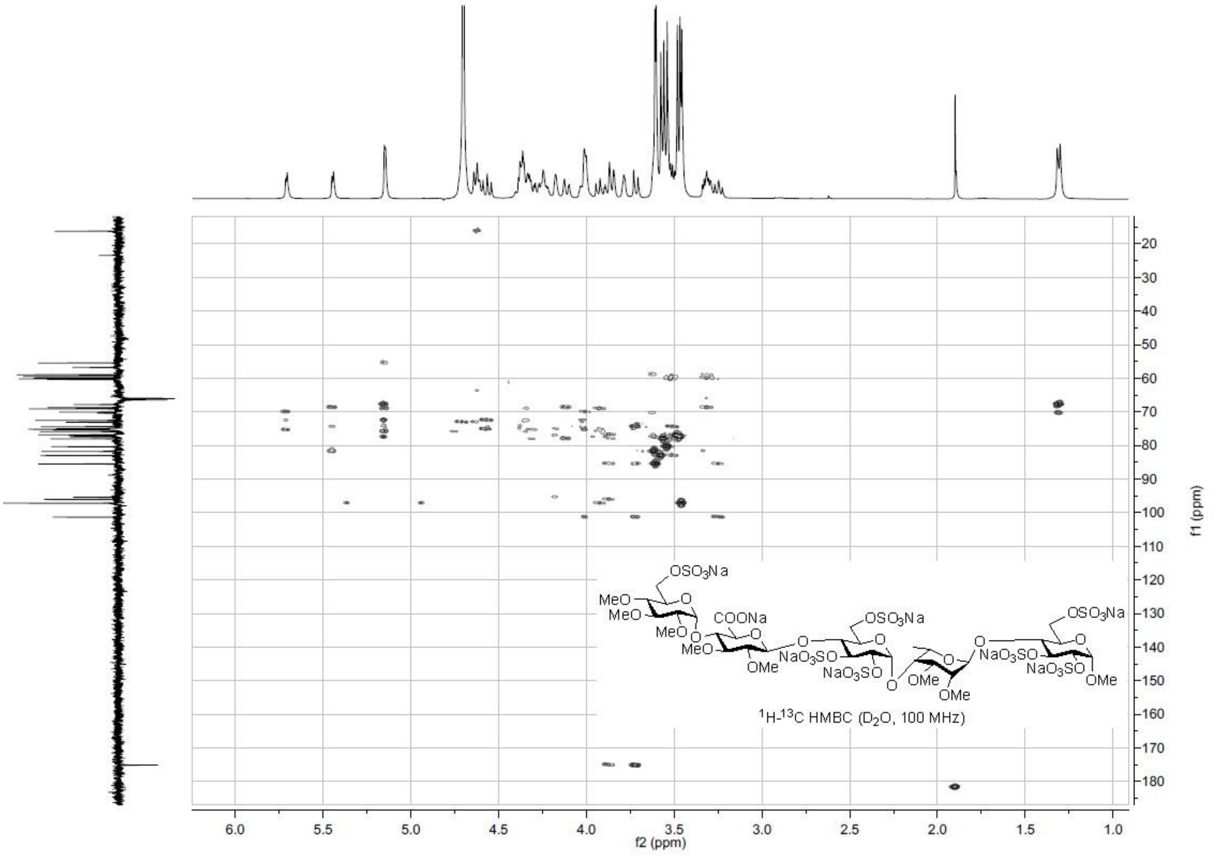
1H-13C HMBC (D2O, 100 MHz)
f2 (ppm)
f1 (ppm)

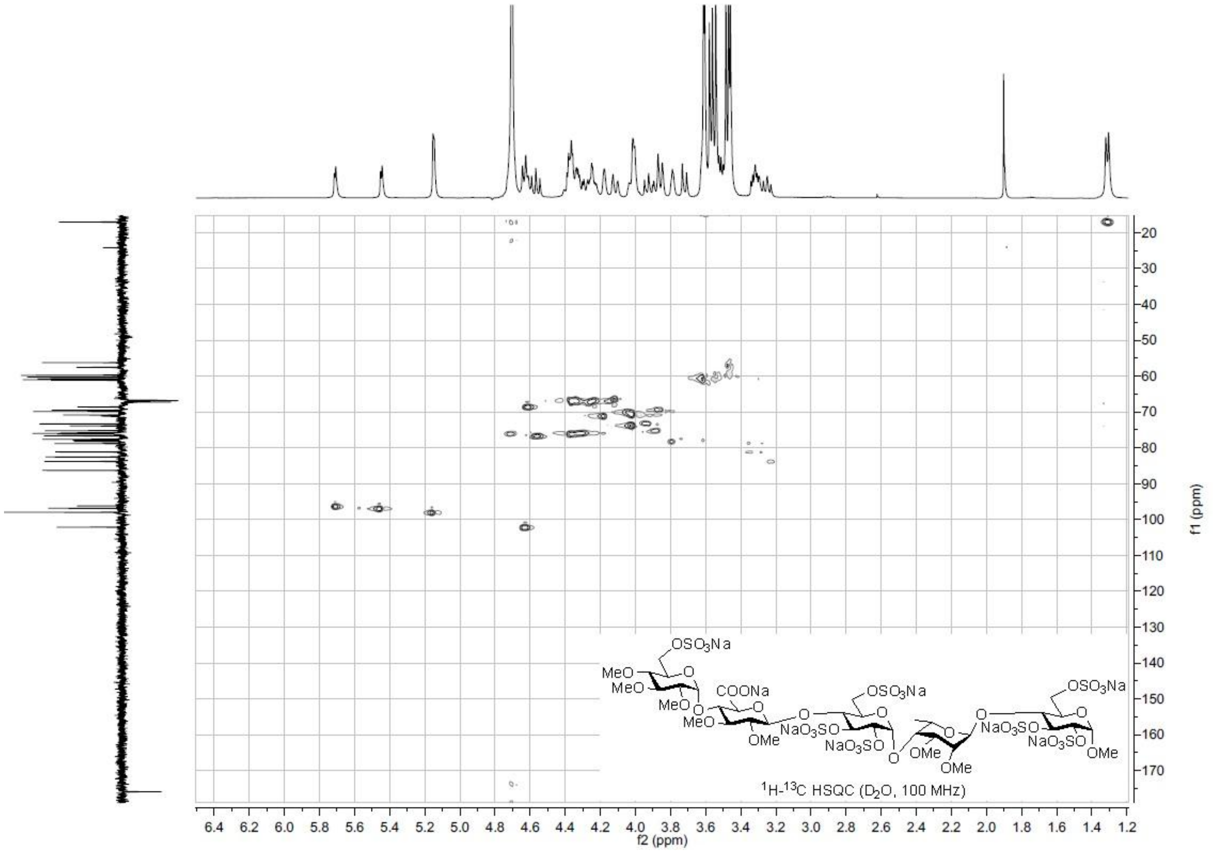

## $^{1}H$ and $^{13}C$ NMR spectra of compound 3:

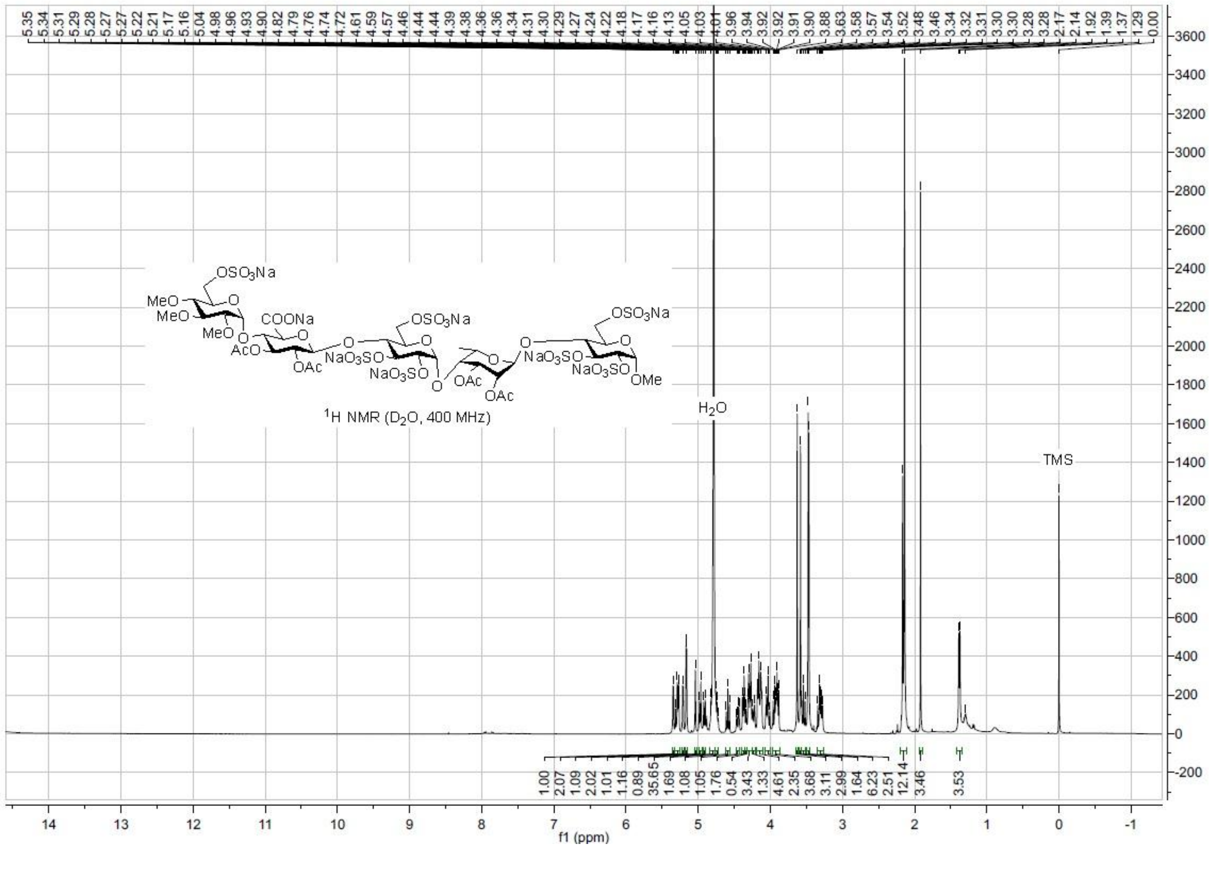

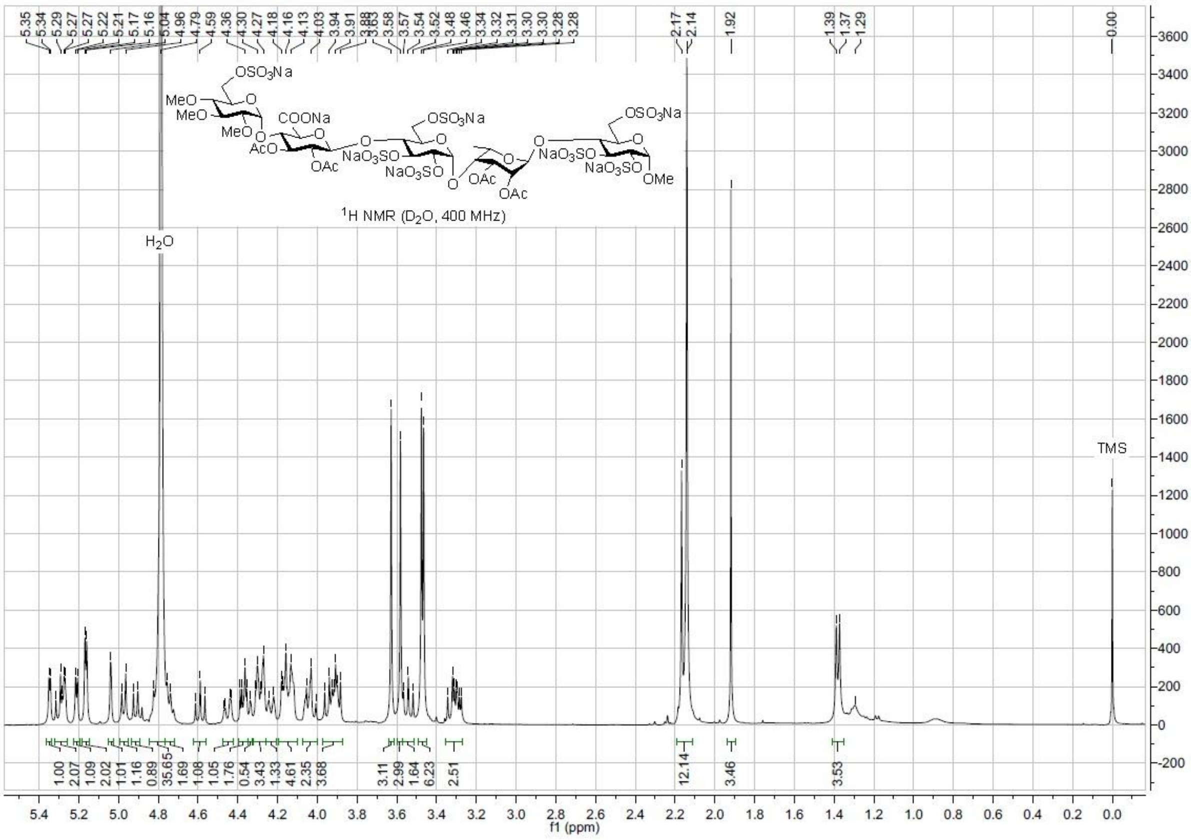

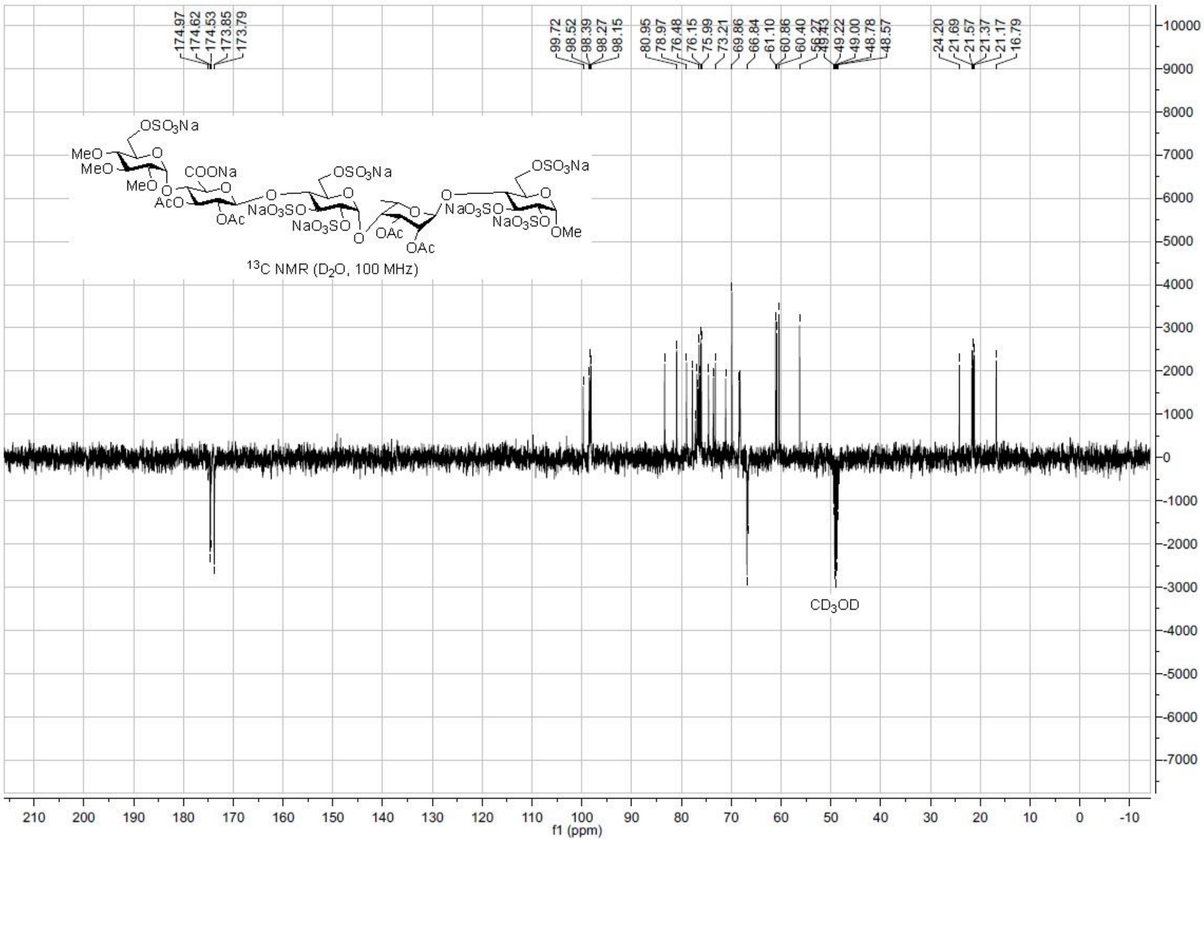

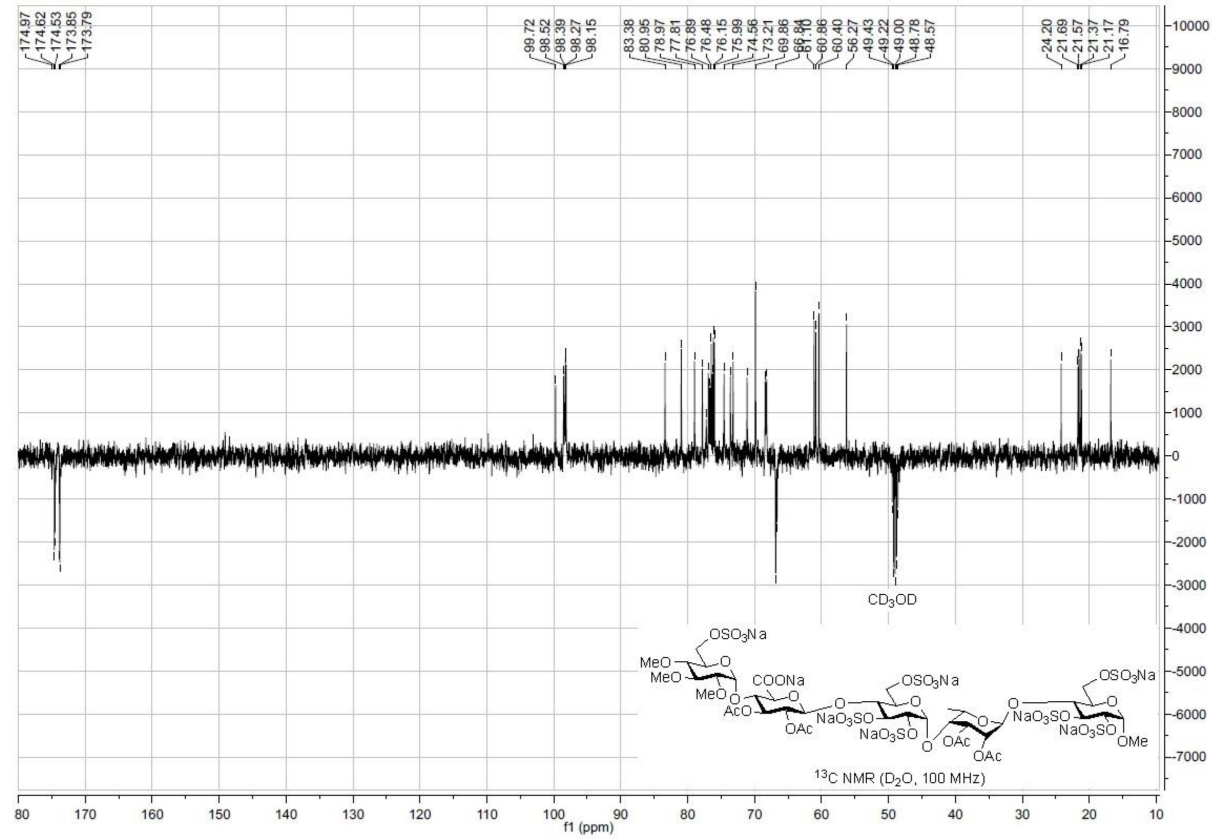
174.97
174.62
174.53
173.85
173.79
99.72
98.52
98.39
98.27
98.15
83.38
80.95
78.97
77.81
76.89
76.48
76.15
75.99
74.56
73.21
69.86
60.86
60.40
56.27
49.43
49.22
49.00
48.78
48.57
24.20
21.69
21.57
21.37
21.17
16.79
CD3OD
OSO3Na
MeO
MeO
MeO
COONa
AcO
OAc
NaO3SO
OAc
NaO3SO
OMe
13C NMR (D2O, 100 MHz)
f1 (ppm)

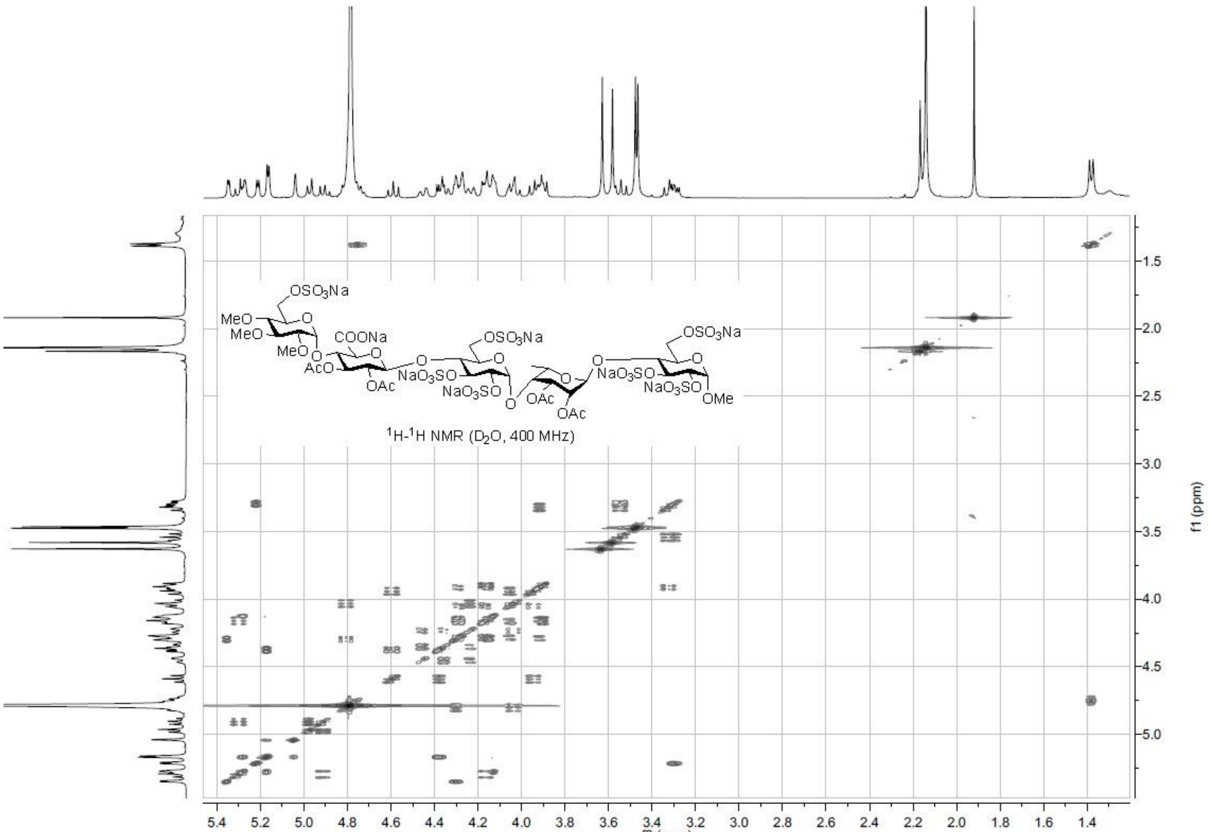
OSO3Na
MeO
MeO
MeO
COONa
AcO
OAc
NaO3SO
OAc
OMe
1H-1H NMR (D2O, 400 MHz)
f2 (ppm)
f1 (ppm)

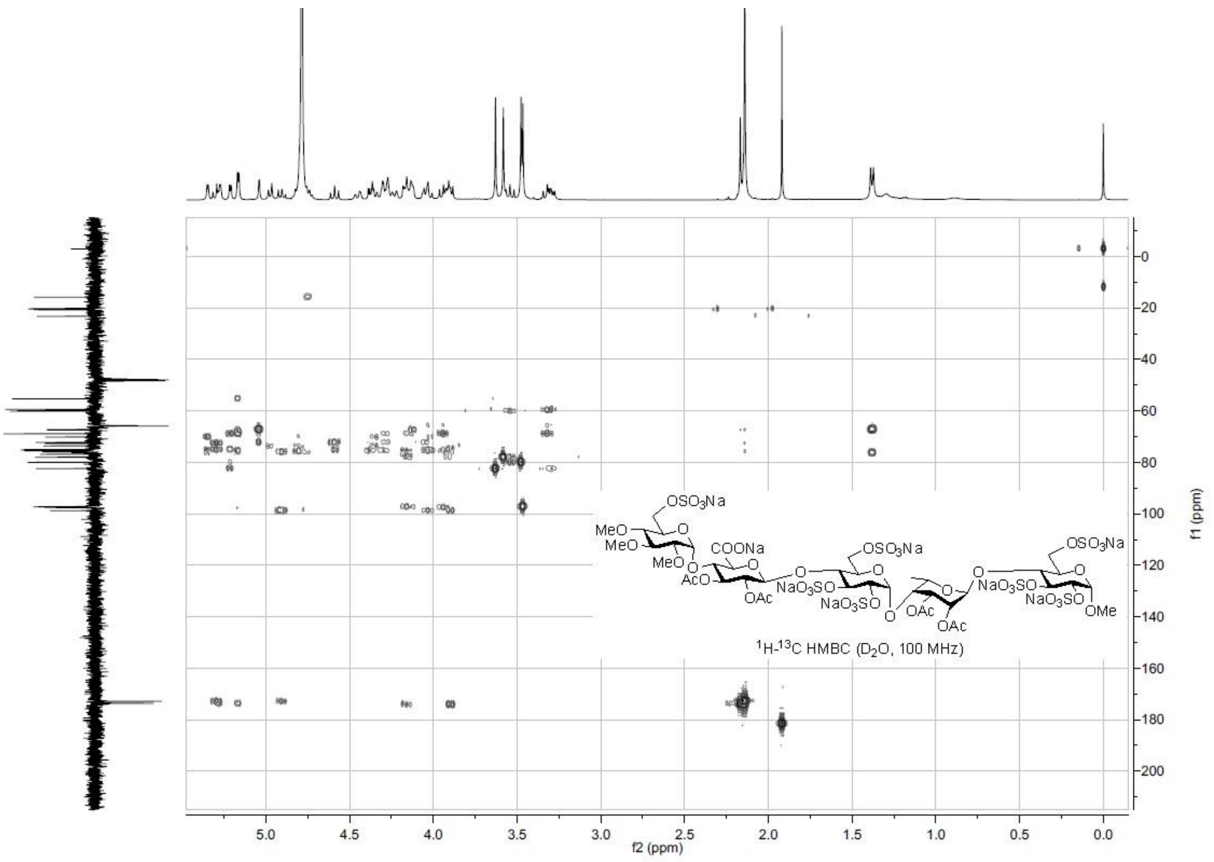

OSO3Na
MeO
MeO
MeO
COONa
AcO
OAc
NaO3SO
NaO3SO
OSO3Na
OAc
OAc
OSO3Na
NaO3SO
NaO3SO
OMe
1H-13C HMBC (D2O, 100 MHz)
f2 (ppm)
f1 (ppm)


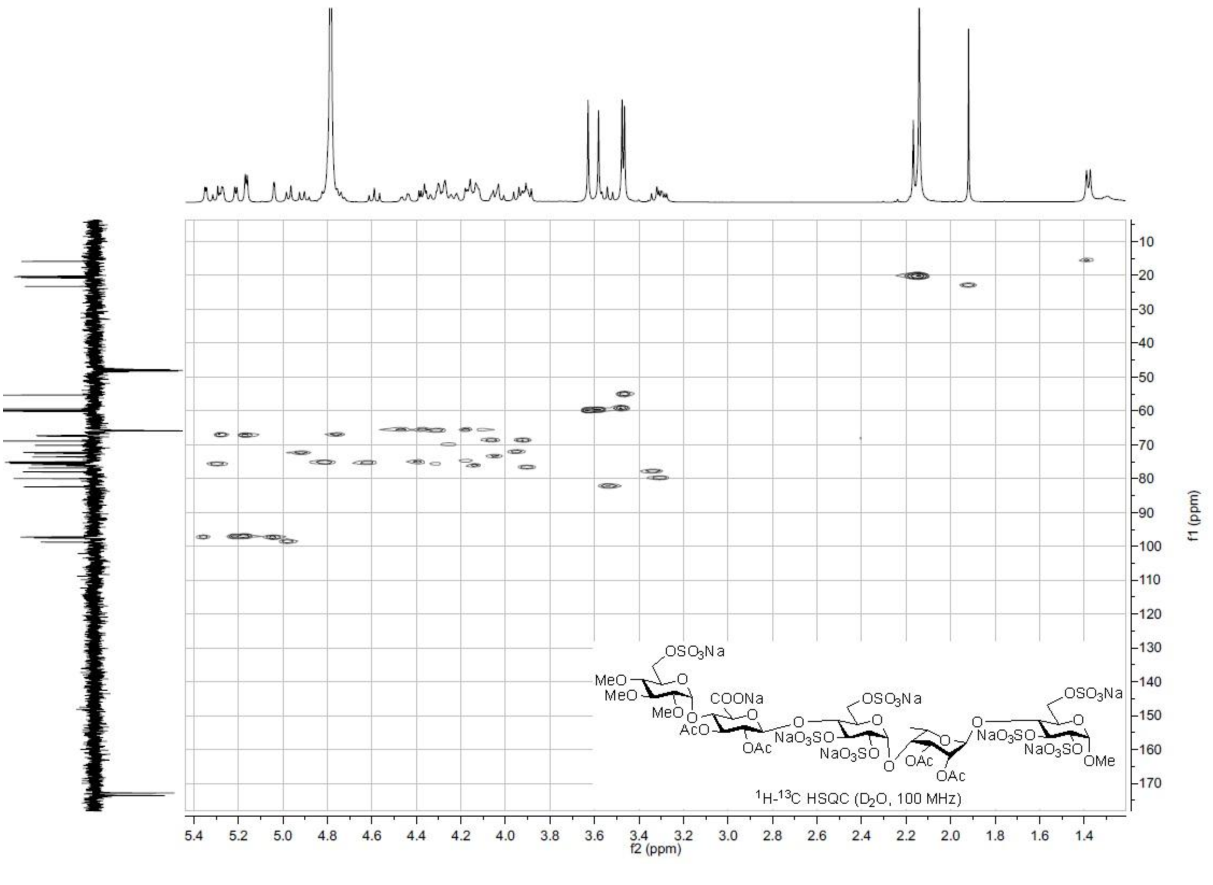

OSO3Na
MeO
MeO
MeO
COONa
AcO
OAc
NaO3SO
NaO3SO
OSO3Na
OAc
OAc
OSO3Na
NaO3SO
NaO3SO
OMe
1H-13C HSQC (D2O, 100 MHz)
f2 (ppm)
f1 (ppm)

**$^{1}$H and $^{13}$C NMR spectra of compound 4:**

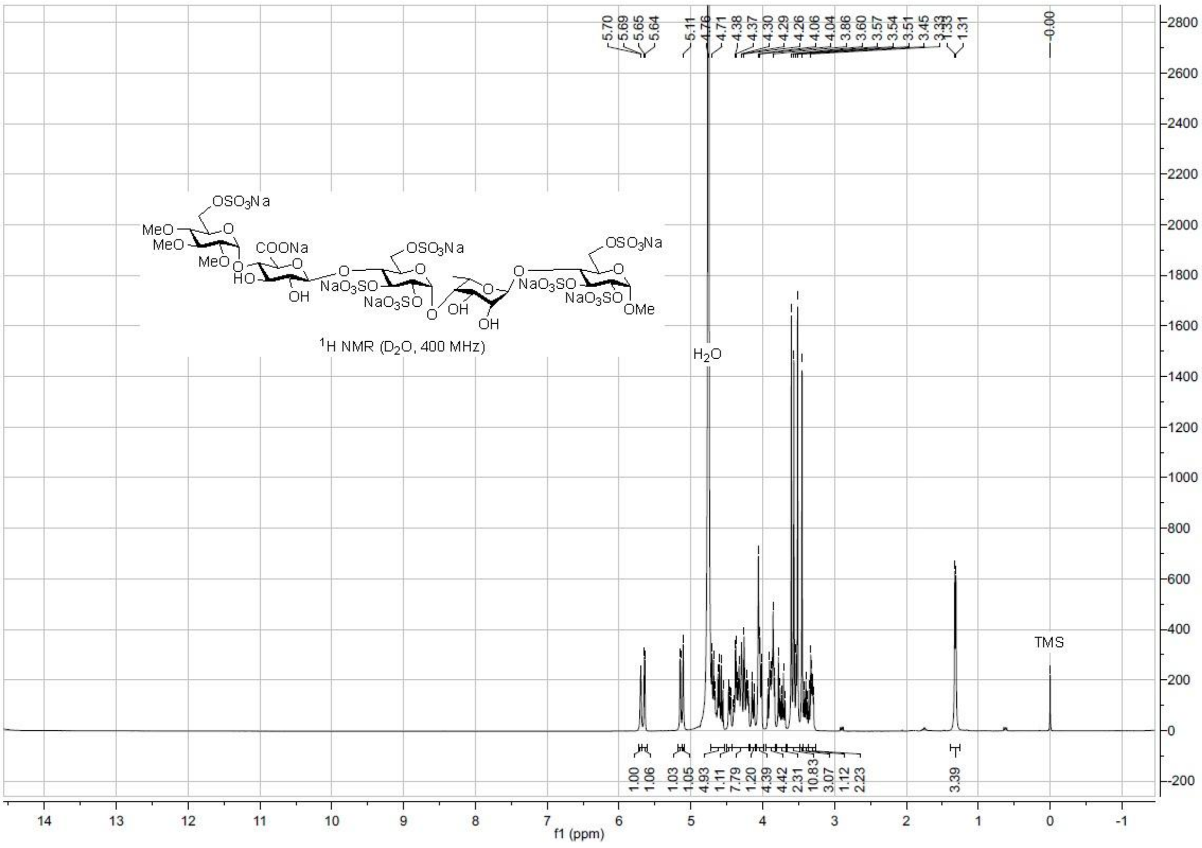


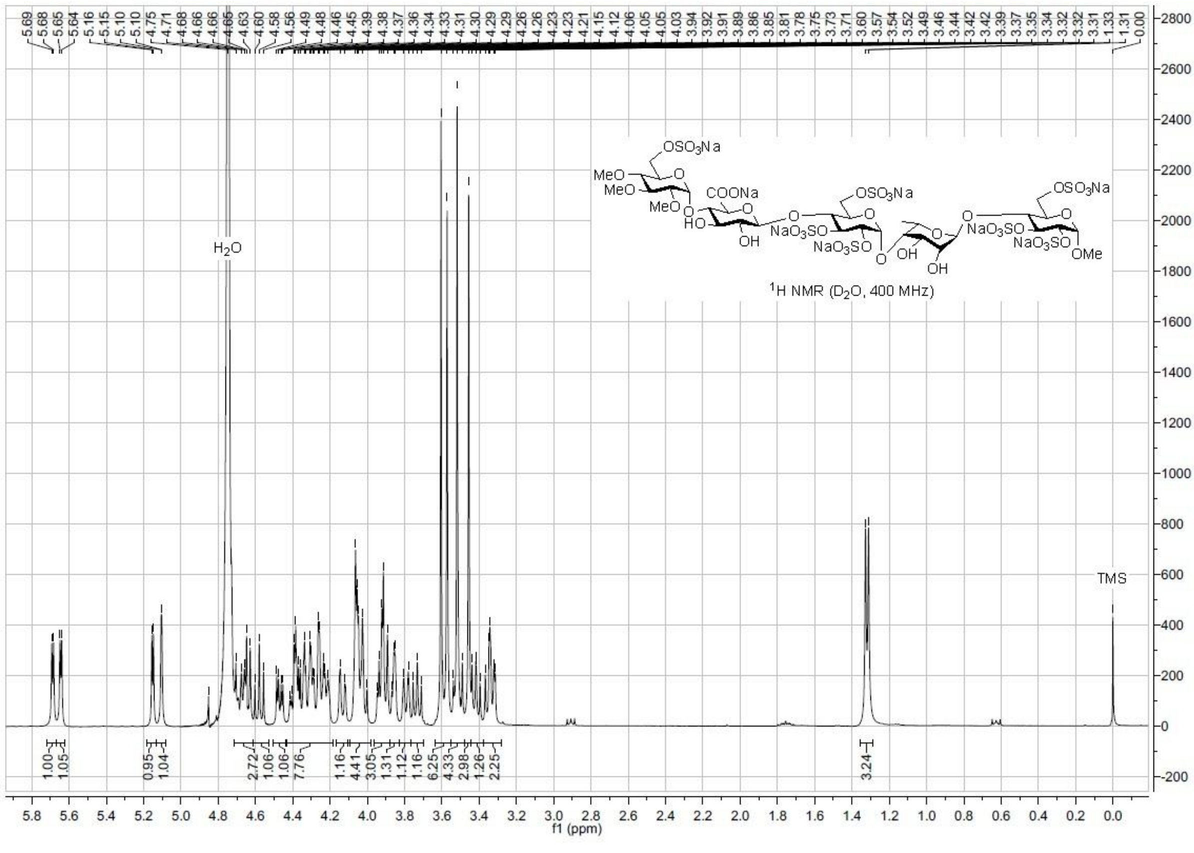

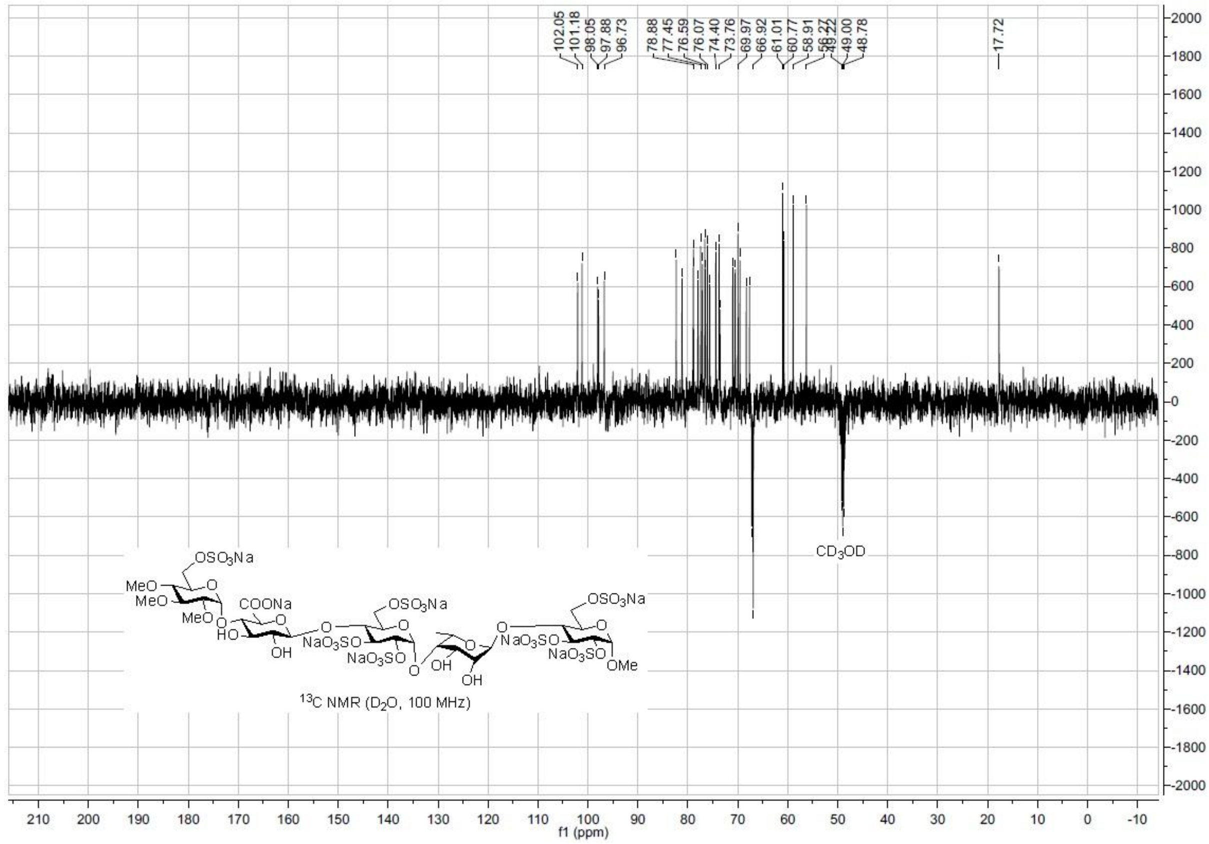

102.05
101.18
98.05
97.88
96.73
78.88
77.45
76.59
76.07
74.40
73.76
69.97
66.92
61.01
60.77
58.91
56.27
49.22
49.00
48.78
17.72
CD3OD
13C NMR (D2O, 100 MHz)
f1 (ppm)


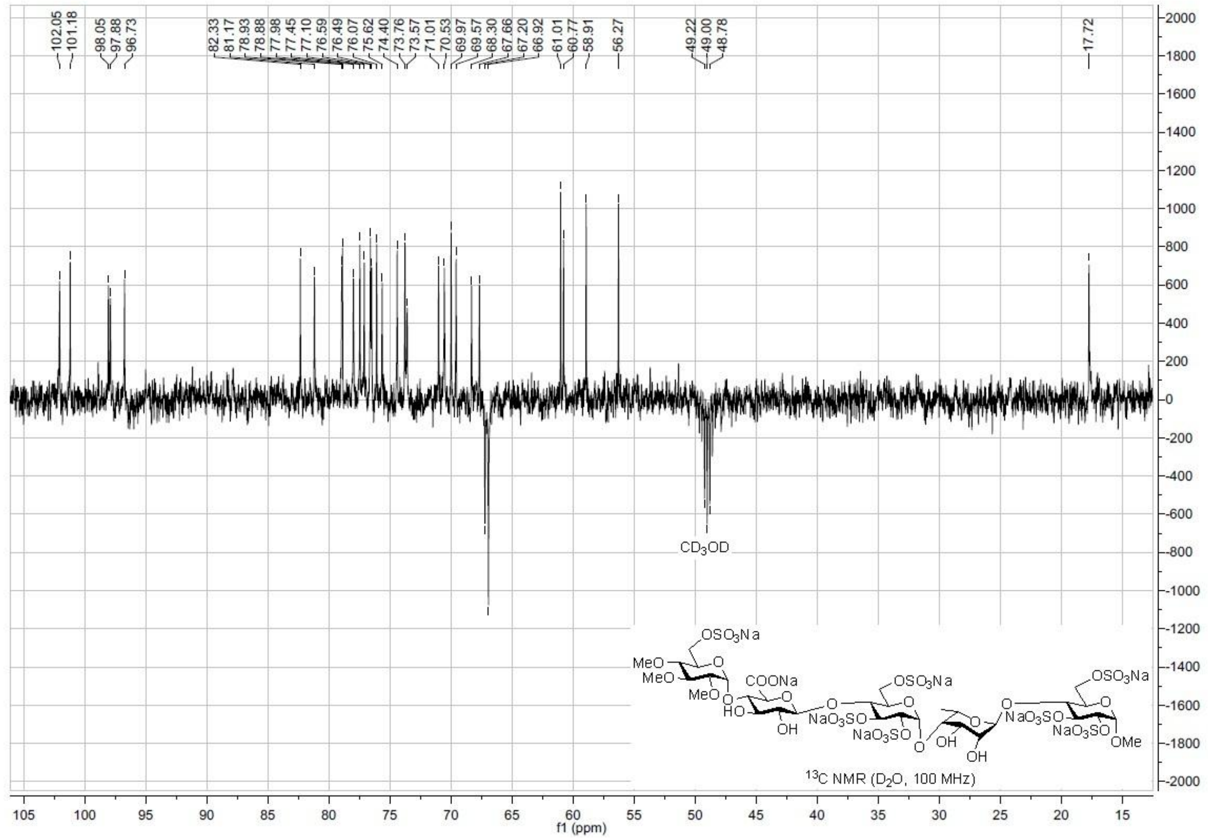

102.05
101.18
98.05
97.88
96.73
82.33
81.17
78.93
78.88
77.98
77.45
77.10
76.59
76.49
76.07
75.62
74.40
73.76
73.57
71.01
70.53
69.97
69.57
68.30
67.66
67.20
66.92
61.01
60.77
58.91
56.27
49.22
49.00
48.78
17.72
CD3OD
13C NMR (D2O, 100 MHz)
f1 (ppm)

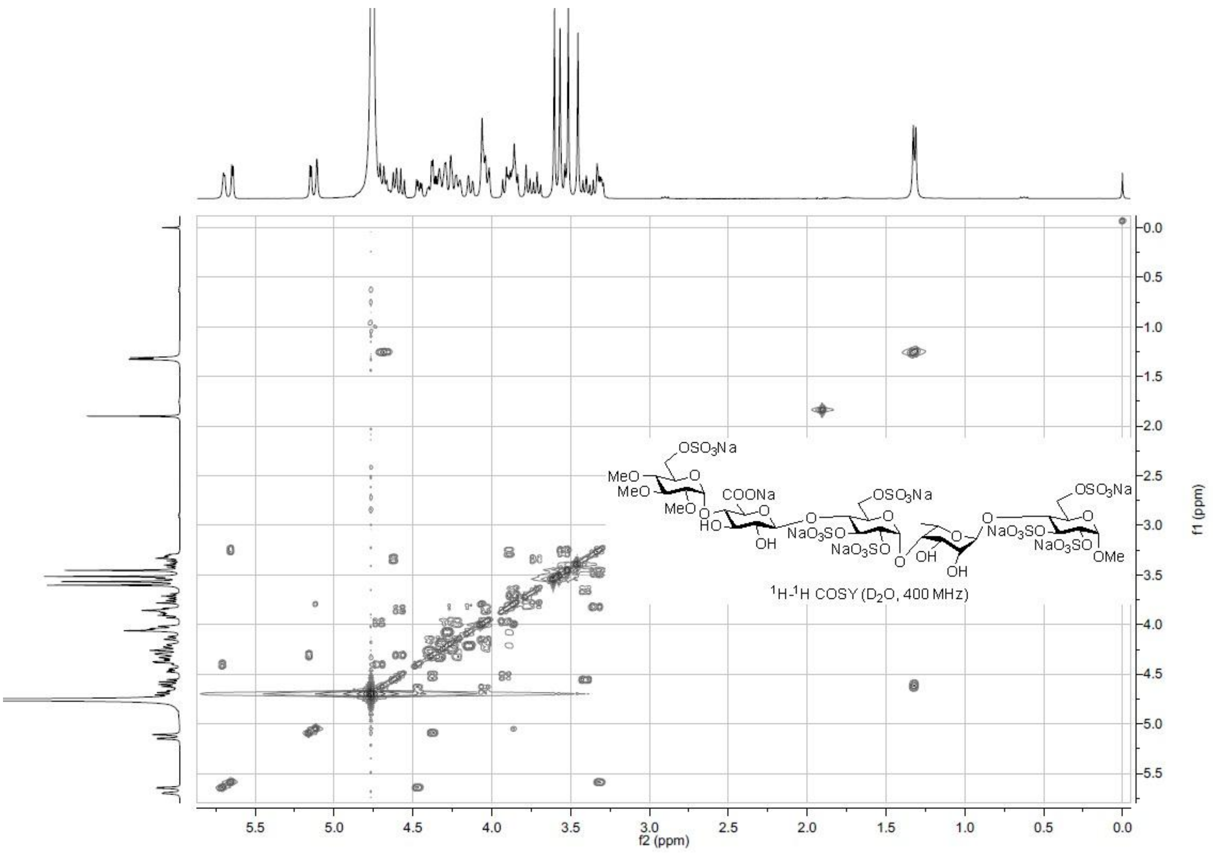
OSO3Na
MeO
MeO
MeO
COONa
HO
OH
NaO3SO
OSO3Na
NaO3SO
OH
OH
NaO3SO
OSO3Na
NaO3SO
OMe
¹H-¹H COSY (D2O, 400 MHz)
f1 (ppm)
f2 (ppm)

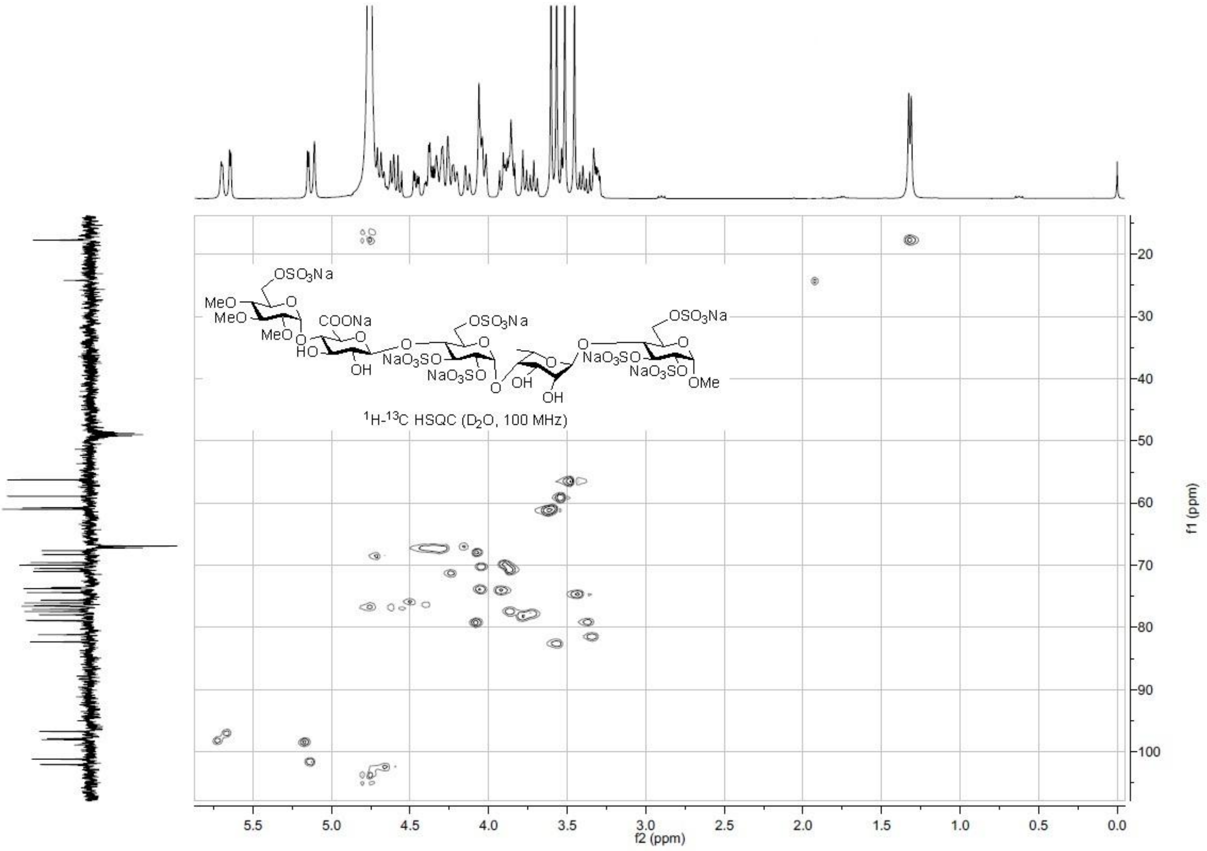
OSO3Na
MeO
MeO
MeO
COONa
HO
OH
NaO3SO
OSO3Na
NaO3SO
OH
OH
NaO3SO
OSO3Na
NaO3SO
OMe
¹H-¹³C HSQC (D2O, 100 MHz)
f1 (ppm)
f2 (ppm)

**$^{1}H$ and $^{13}C$ NMR spectra of compound 7:**

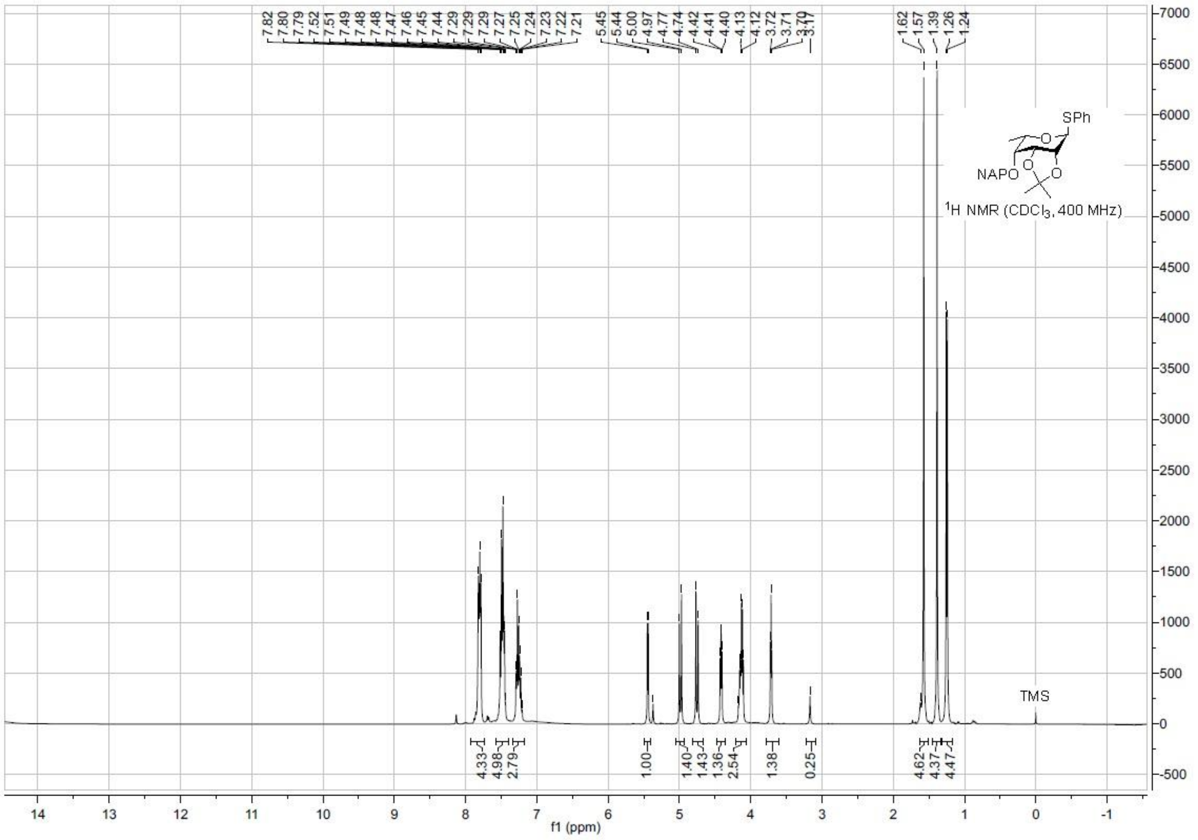


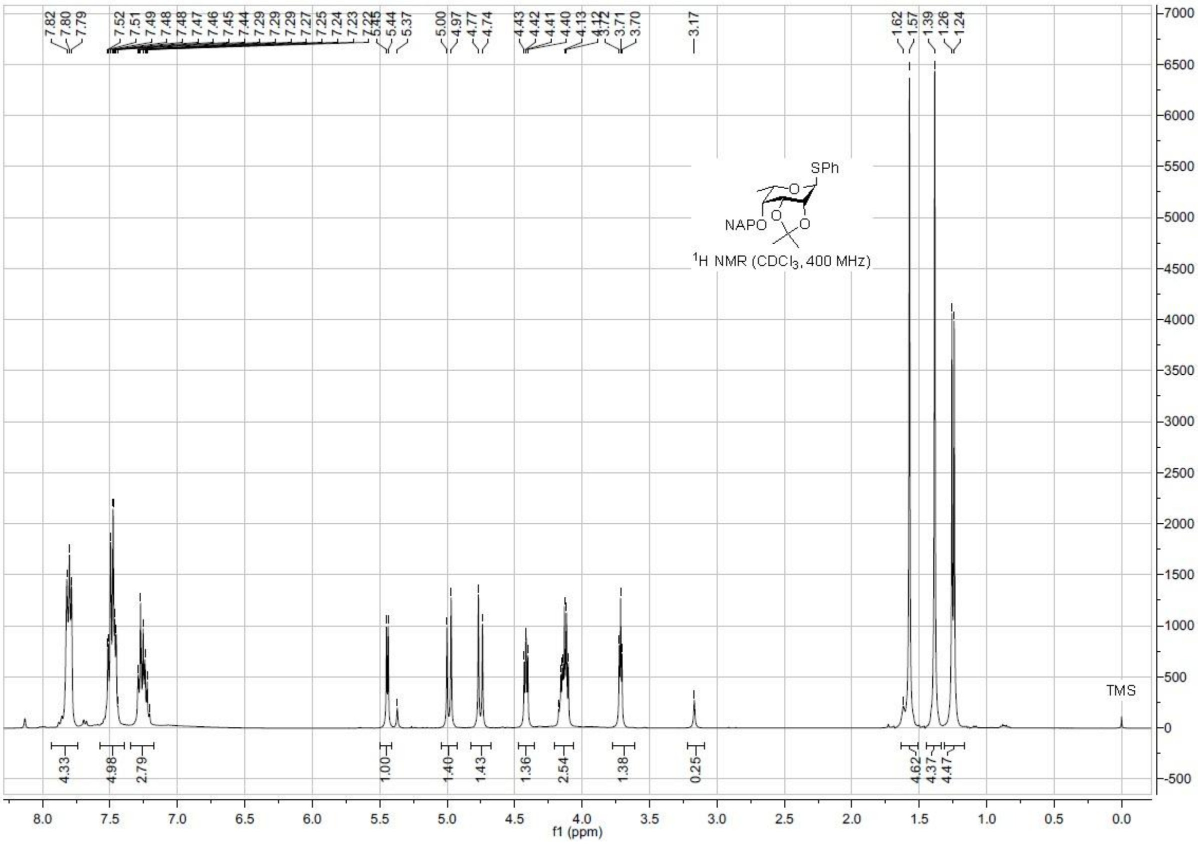

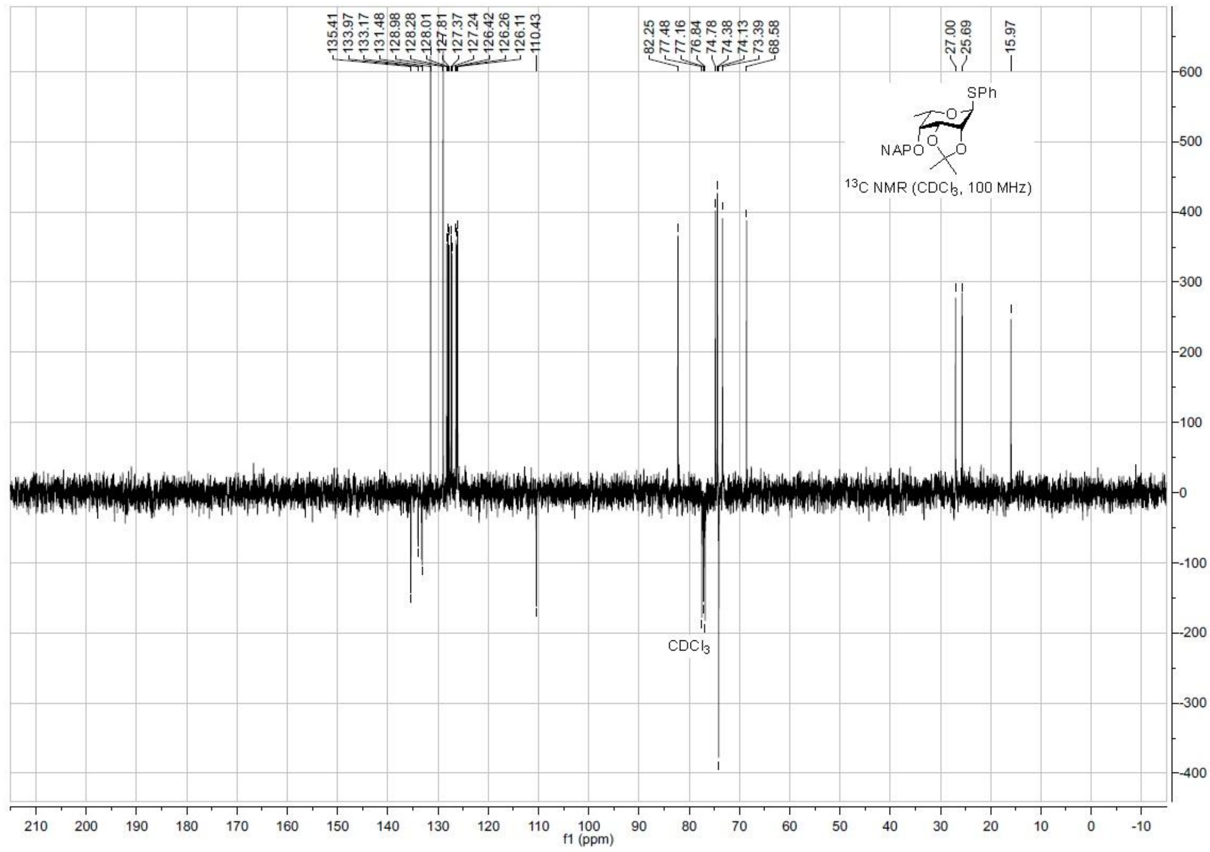
135.41
133.97
133.17
131.48
128.98
128.28
128.01
127.81
127.37
127.24
126.42
126.26
126.11
110.43
82.25
77.48
77.16
76.84
74.78
74.38
74.13
73.39
68.58
27.00
25.69
15.97
SPh
NAPO
O
13C NMR (CDCl3, 100 MHz)
CDCl3
f1 (ppm)

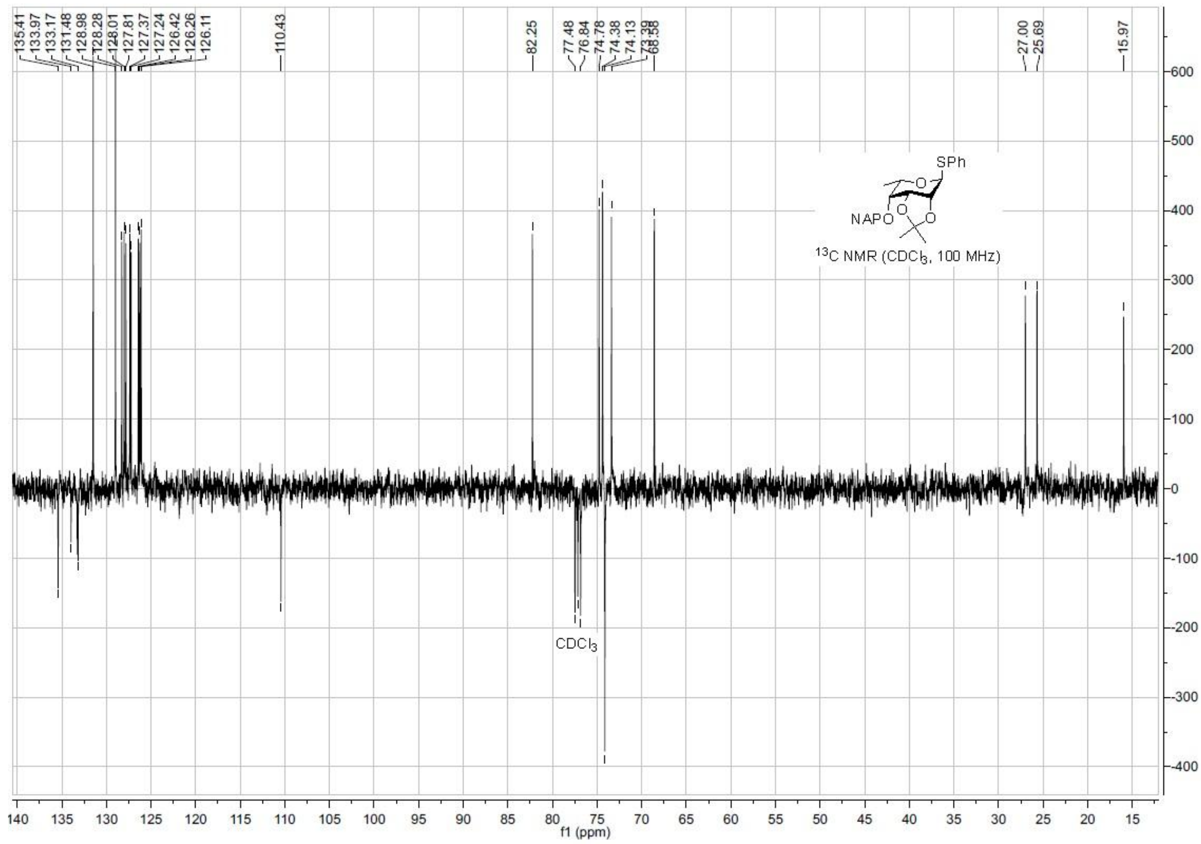
135.41
133.97
133.17
131.48
128.98
128.28
128.01
127.81
127.37
127.24
126.42
126.26
126.11
110.43
82.25
77.48
76.84
74.78
74.38
74.13
27.00
25.69
15.97
SPh
NAPO
O
13C NMR (CDCl3, 100 MHz)
CDCl3
f1 (ppm)

**$^{1}$H and $^{13}$C NMR spectra of compound 8:**

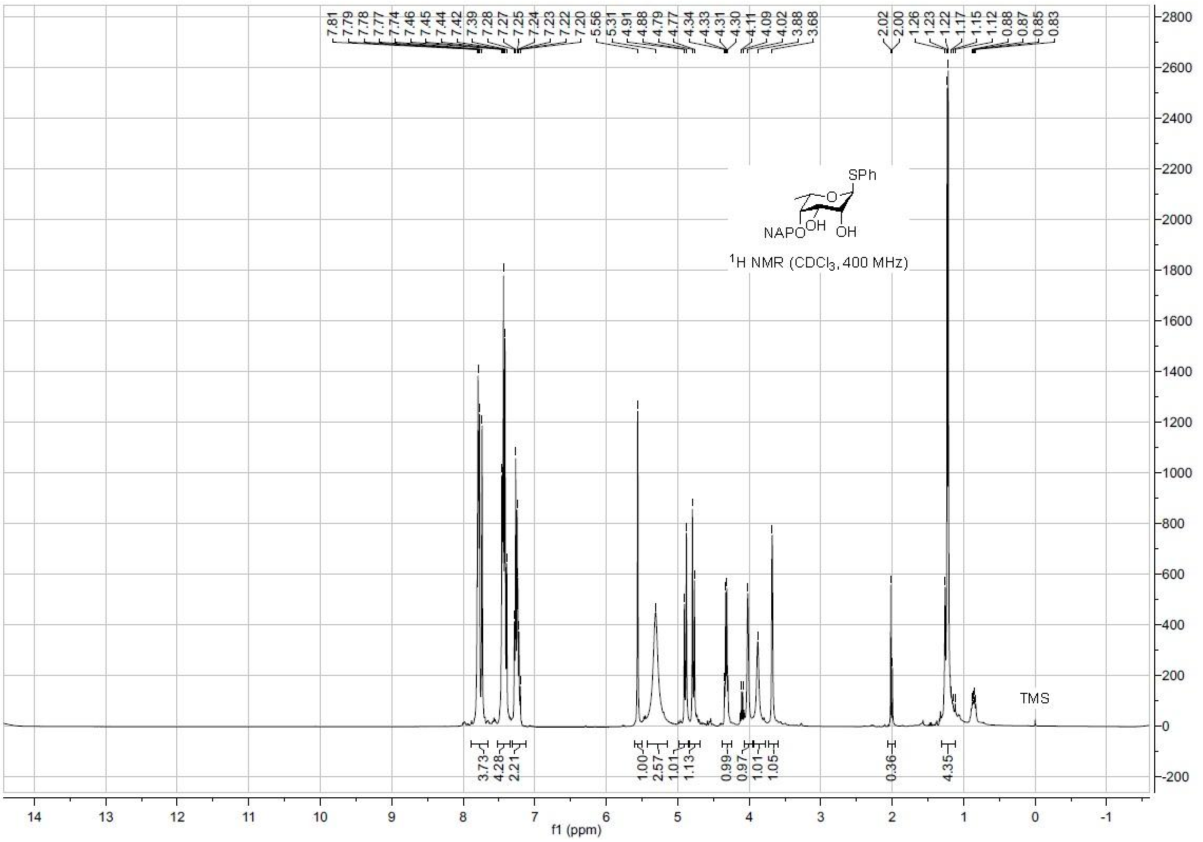


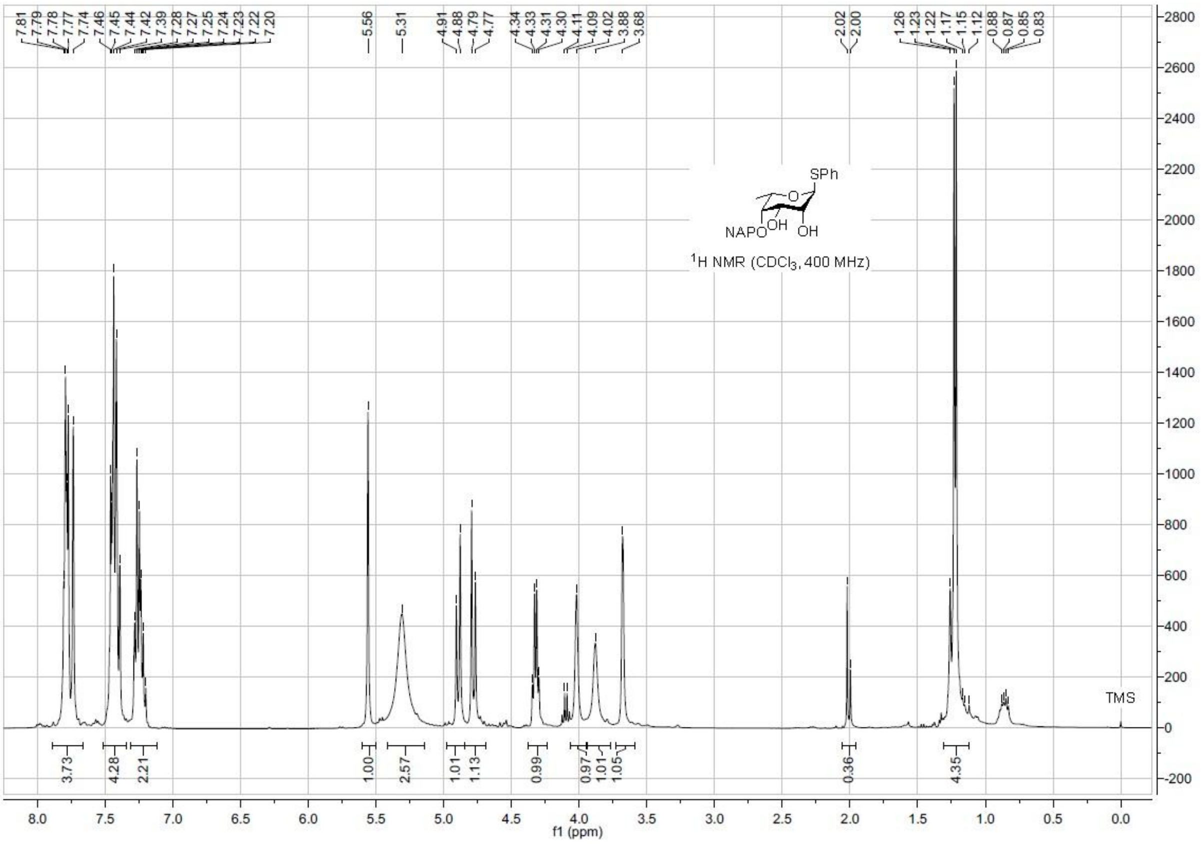

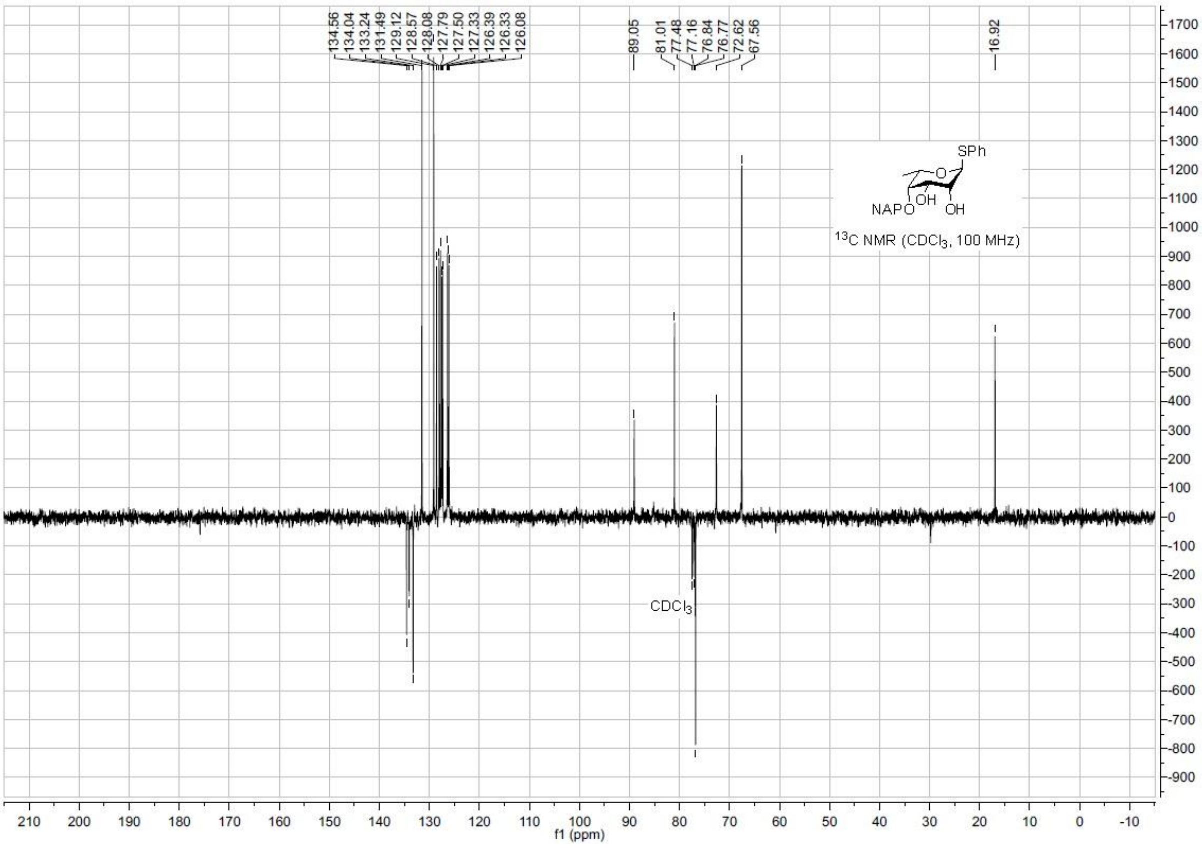
134.56
134.04
133.24
131.49
129.12
128.57
128.08
127.79
127.50
127.33
126.39
126.33
126.08
89.05
81.01
77.48
77.16
76.84
76.77
72.62
67.56
16.92
SPh
NAPO
OH
OH
$^{13}$C NMR (CDCl$_3$, 100 MHz)
CDCl$_3$
f1 (ppm)

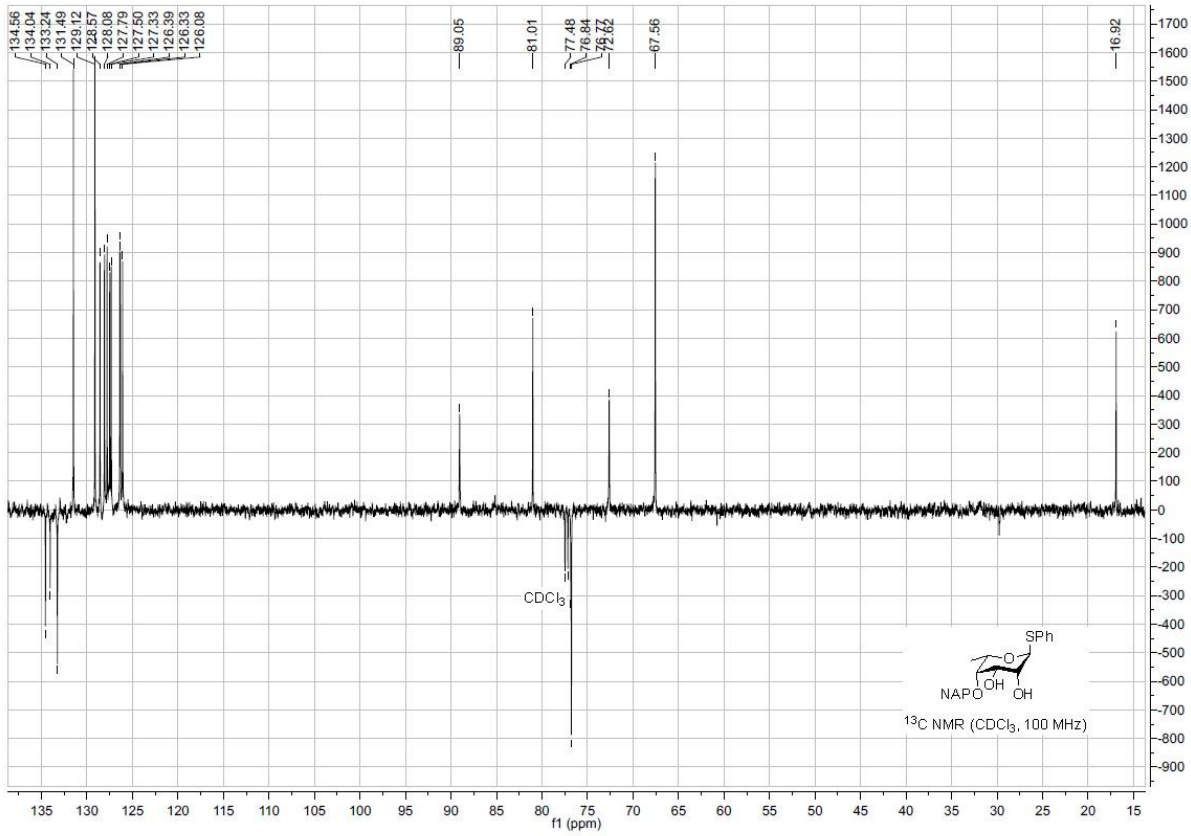
134.56
134.04
133.24
131.49
129.12
128.57
128.08
127.79
127.50
127.33
126.39
126.33
126.08
89.05
81.01
77.48
76.84
76.77
72.62
67.56
16.92
CDCl$_3$
SPh
NAPO
OH
OH
$^{13}$C NMR (CDCl$_3$, 100 MHz)
f1 (ppm)

**$^{1}$H and $^{13}$C NMR spectra of compound 9:**

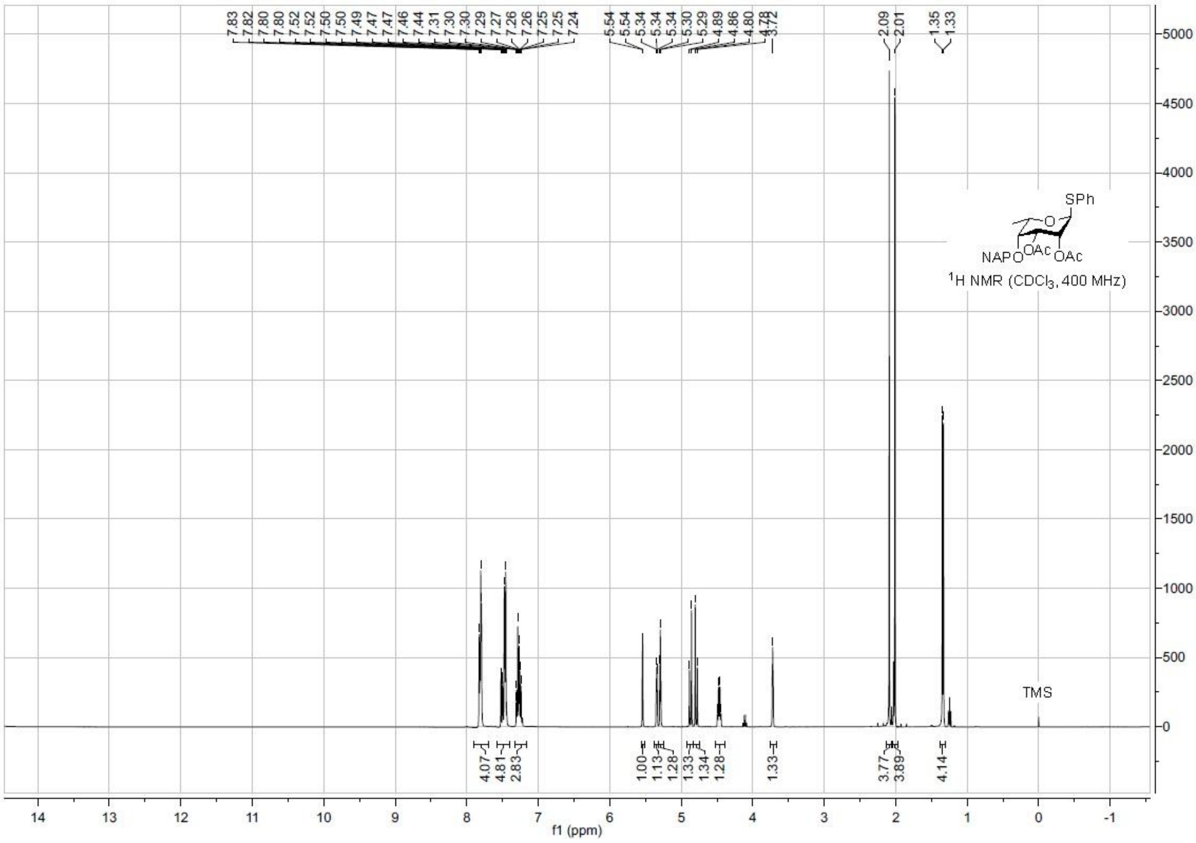


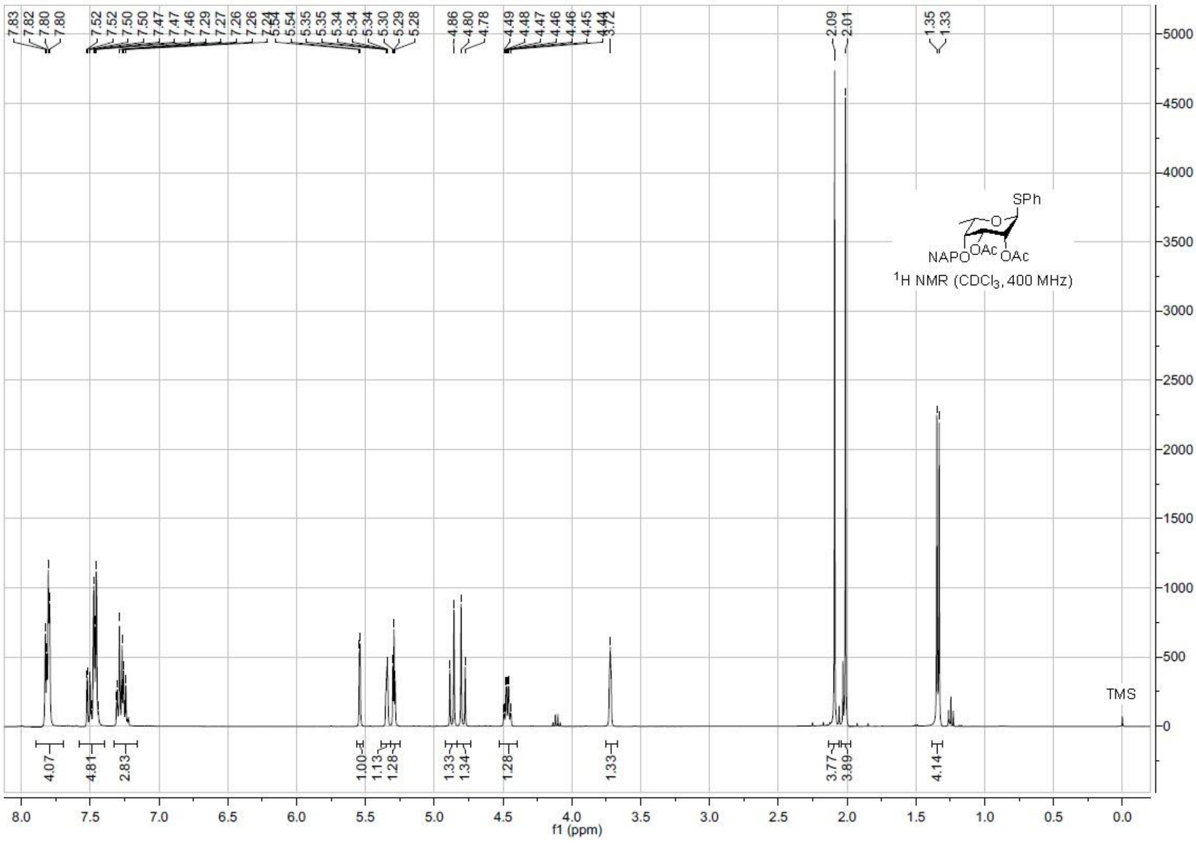

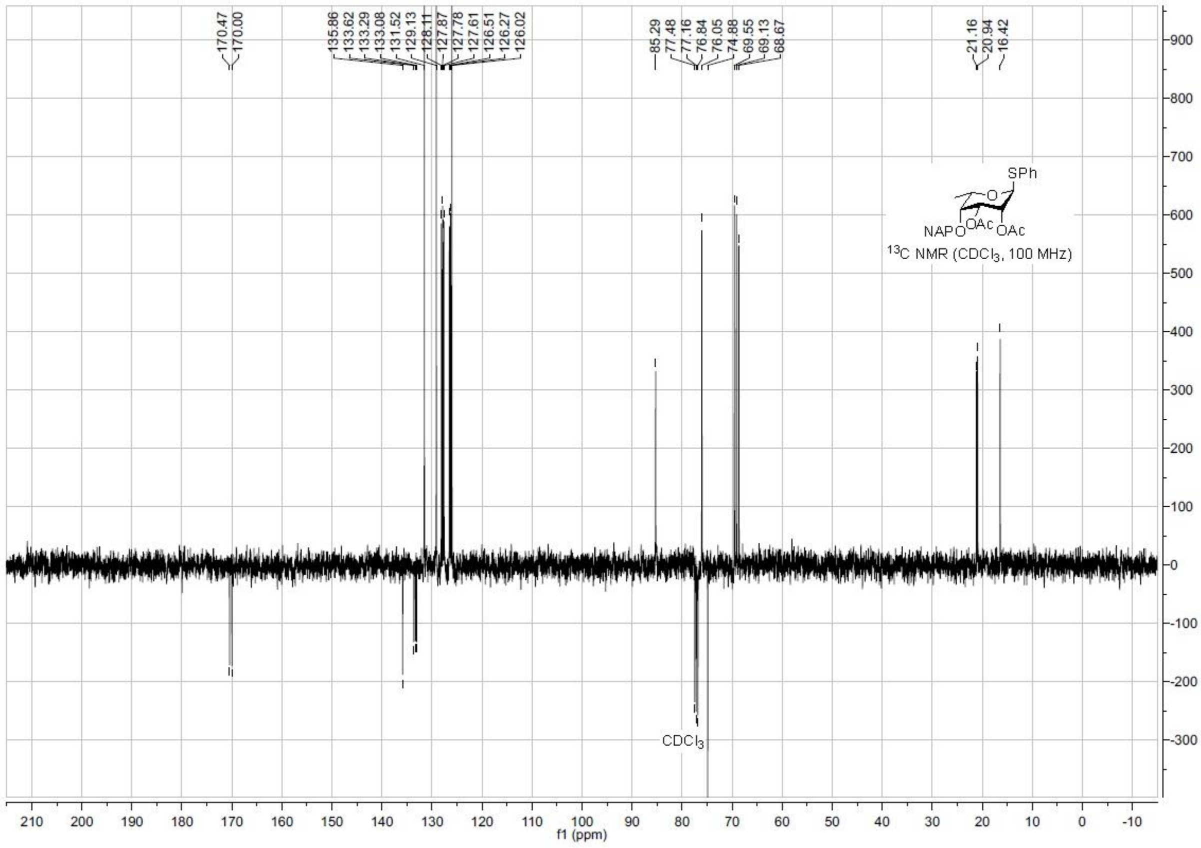
170.47
170.00
135.86
133.62
133.29
133.08
131.52
129.13
128.11
127.87
127.78
127.61
126.51
126.27
126.02
85.29
77.48
77.16
76.84
76.05
74.88
69.55
69.13
68.67
21.16
20.94
16.42
SPh
NAPO
OAc
OAc
$^{13}$C NMR (CDCl$_3$, 100 MHz)
CDCl$_3$
f1 (ppm)

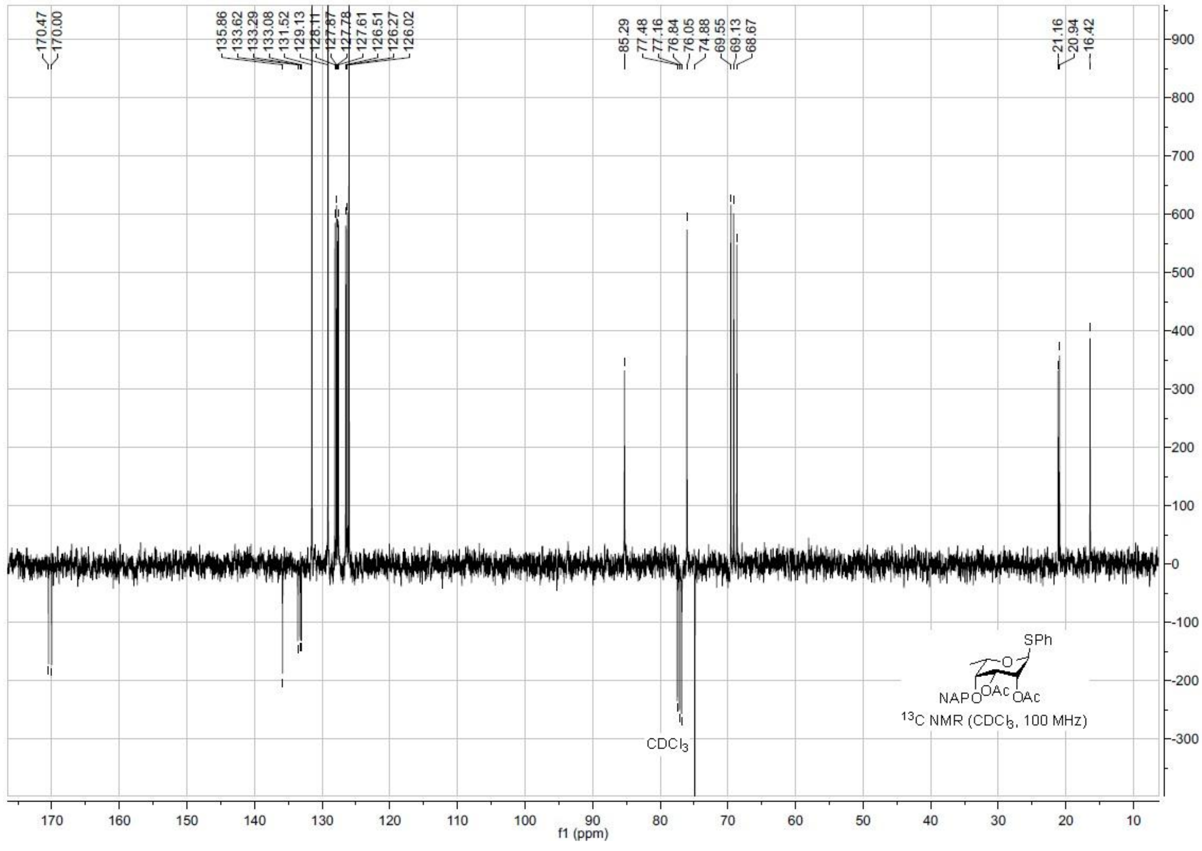
170.47
170.00
135.86
133.62
133.29
133.08
131.52
129.13
128.11
127.87
127.78
127.61
126.51
126.27
126.02
85.29
77.48
77.16
76.84
76.05
74.88
69.55
69.13
68.67
21.16
20.94
16.42
SPh
NAPO
OAc
OAc
$^{13}$C NMR (CDCl$_3$, 100 MHz)
CDCl$_3$
f1 (ppm)

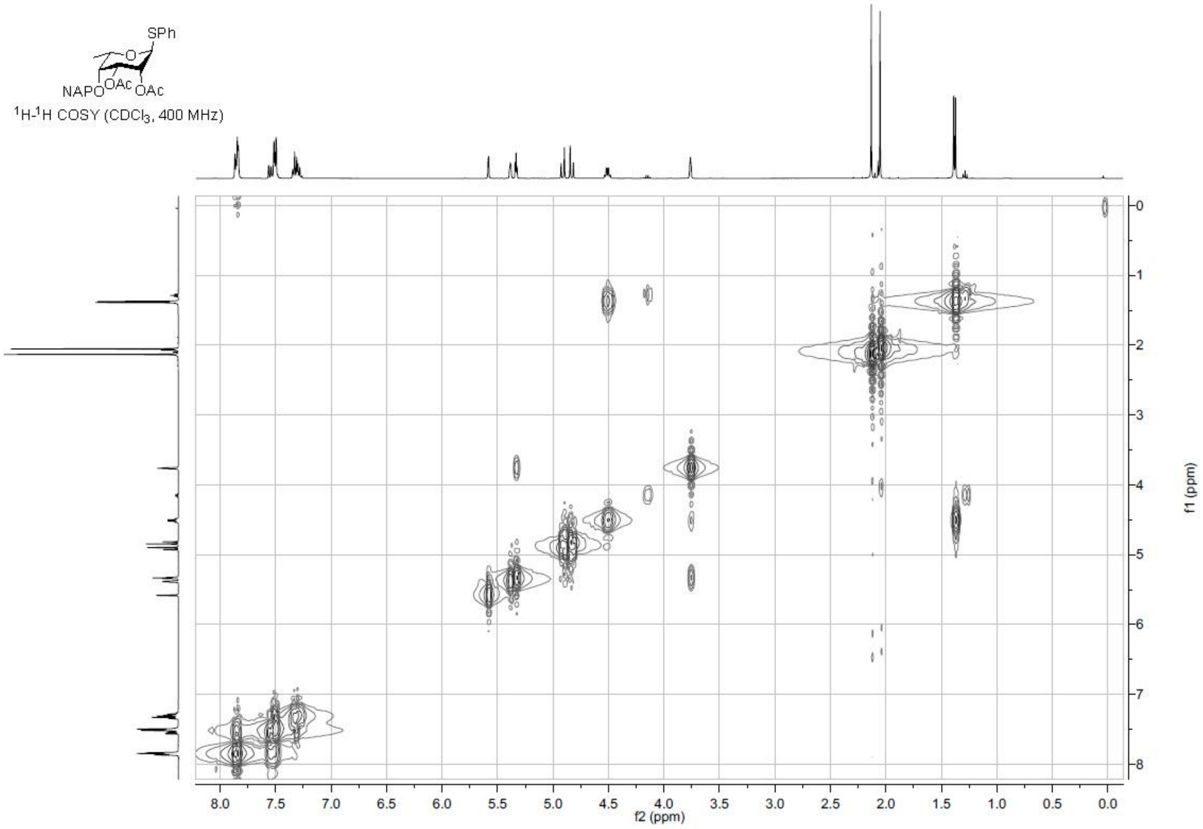
SPh
NAPO
OAc
OAc
$^1$H-$^1$H COSY ($CDCl_3$, 400 MHz)
f2 (ppm)
f1 (ppm)

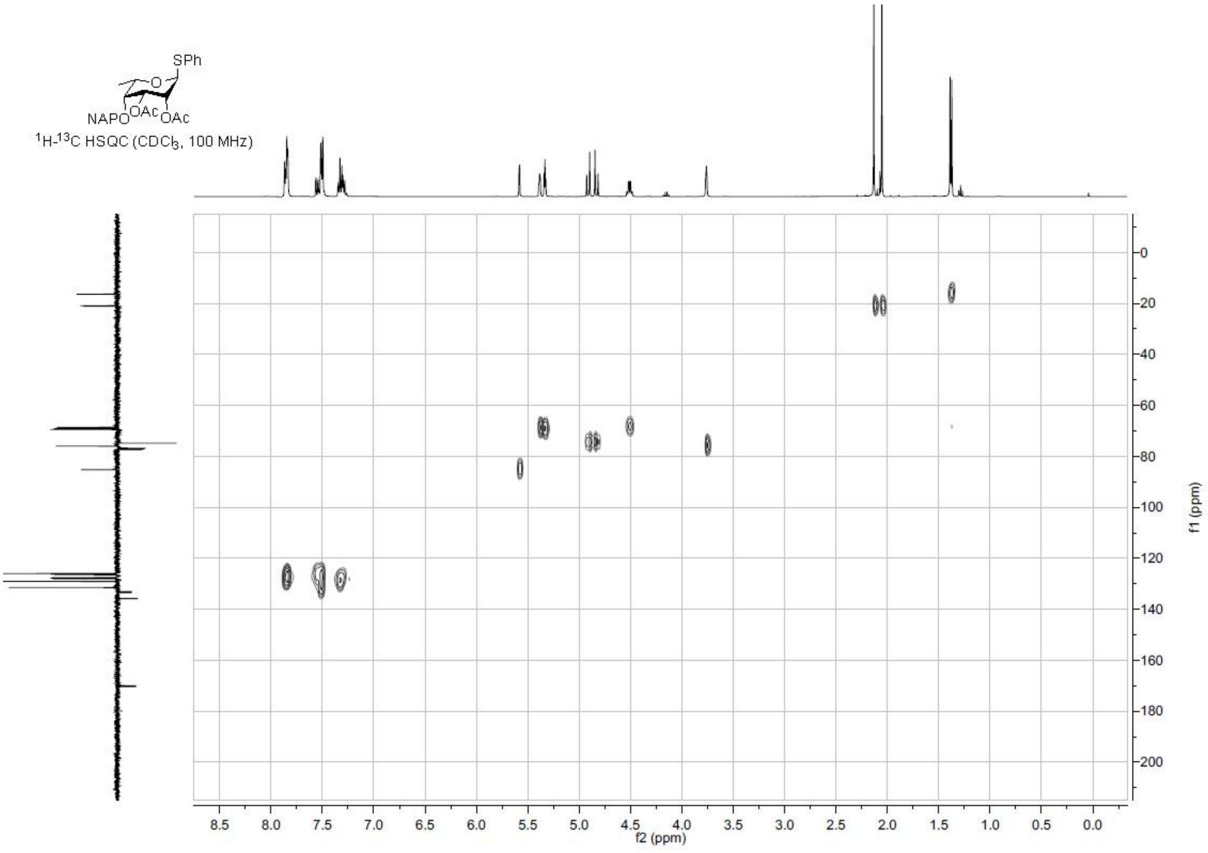
SPh
NAPO
OAc
OAc
$^1$H-$^{13}$C HSQC ($CDCl_3$, 100 MHz)
f2 (ppm)
f1 (ppm)

**$^{1}$H and $^{13}$C NMR spectra of compound 11:**

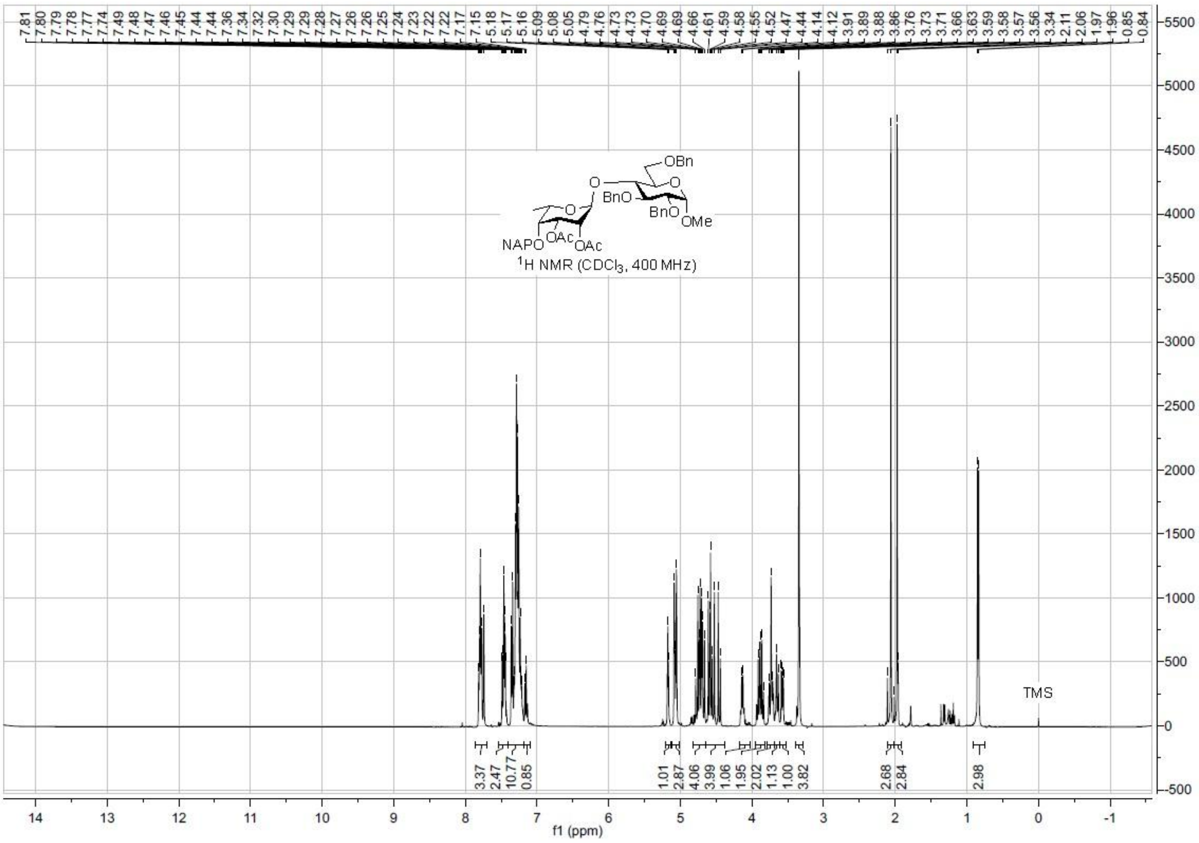


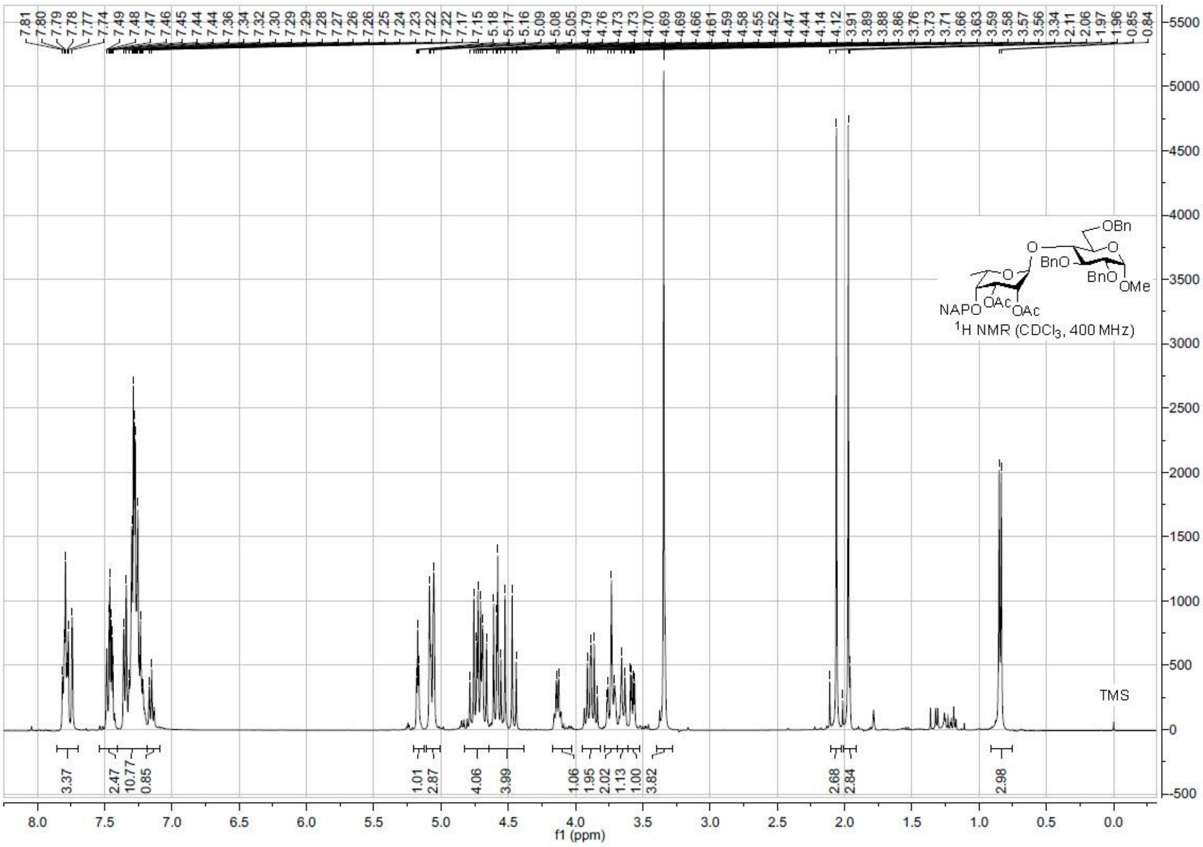

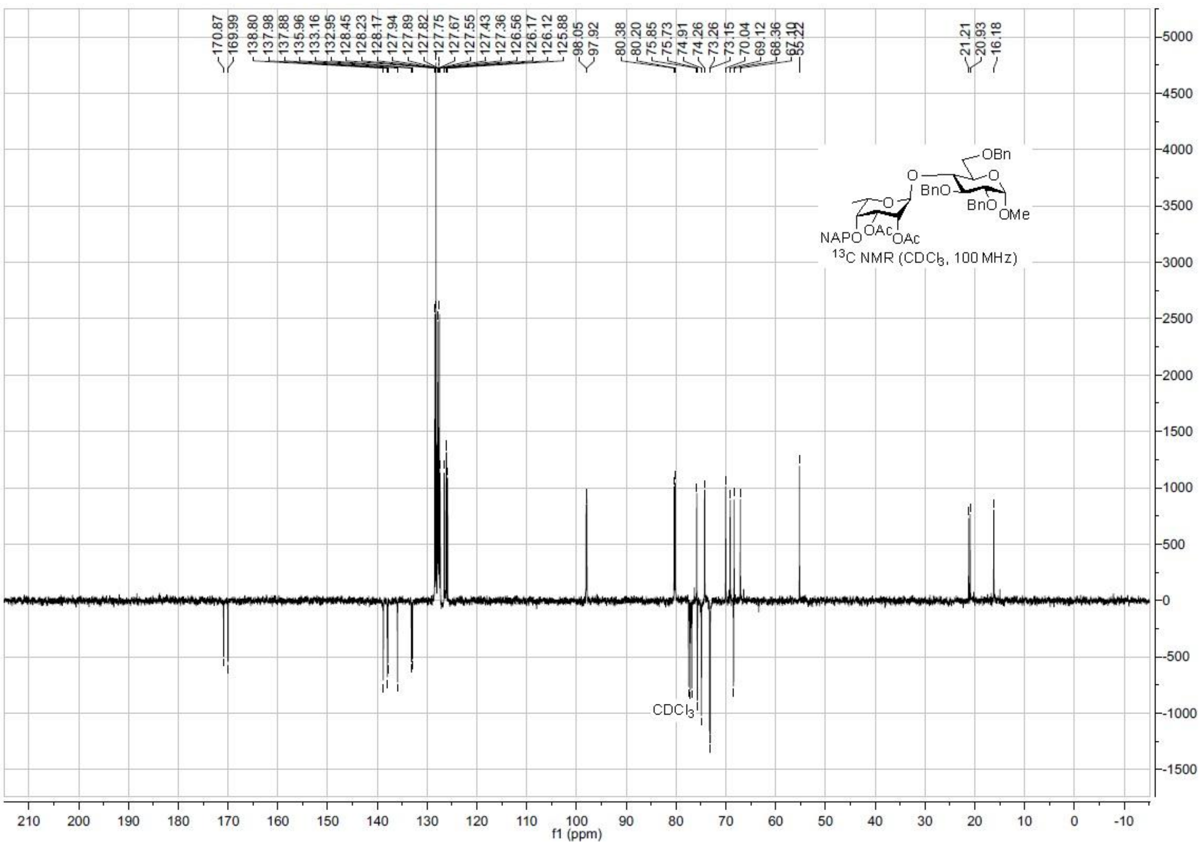

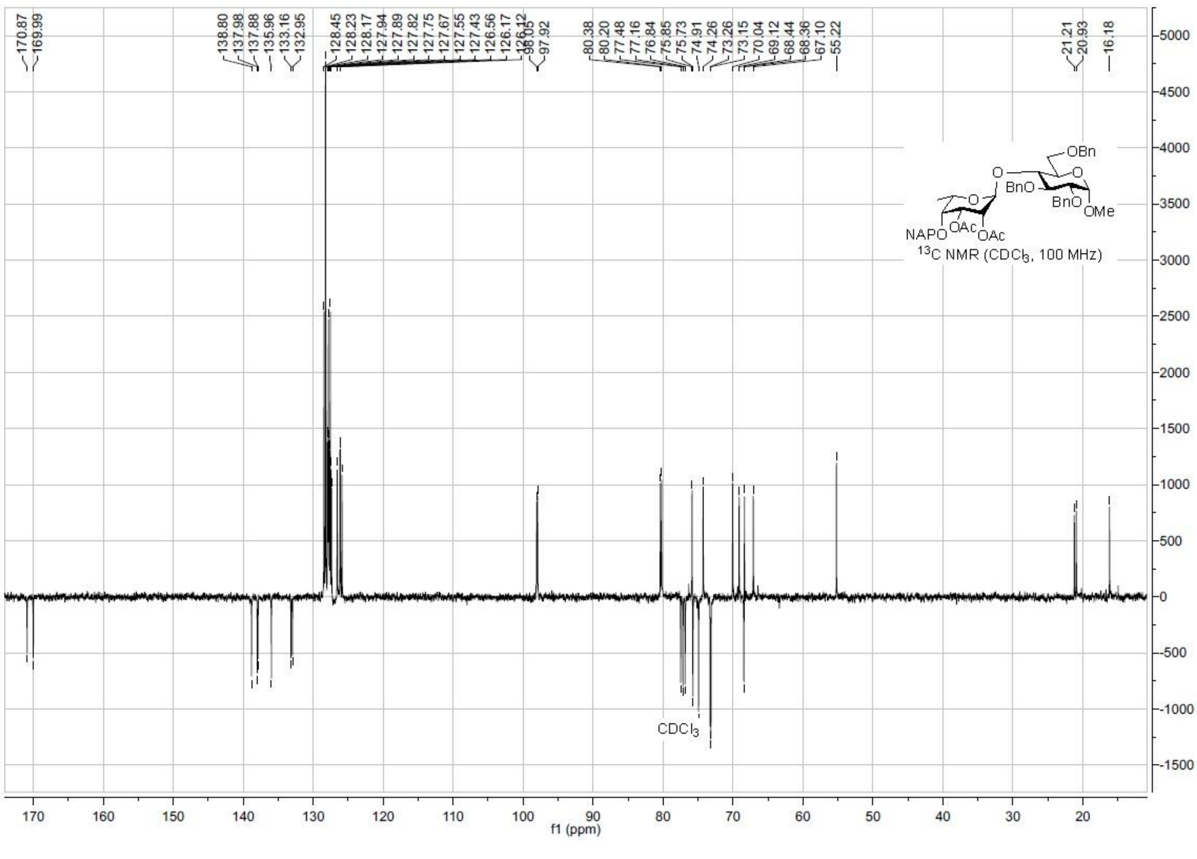

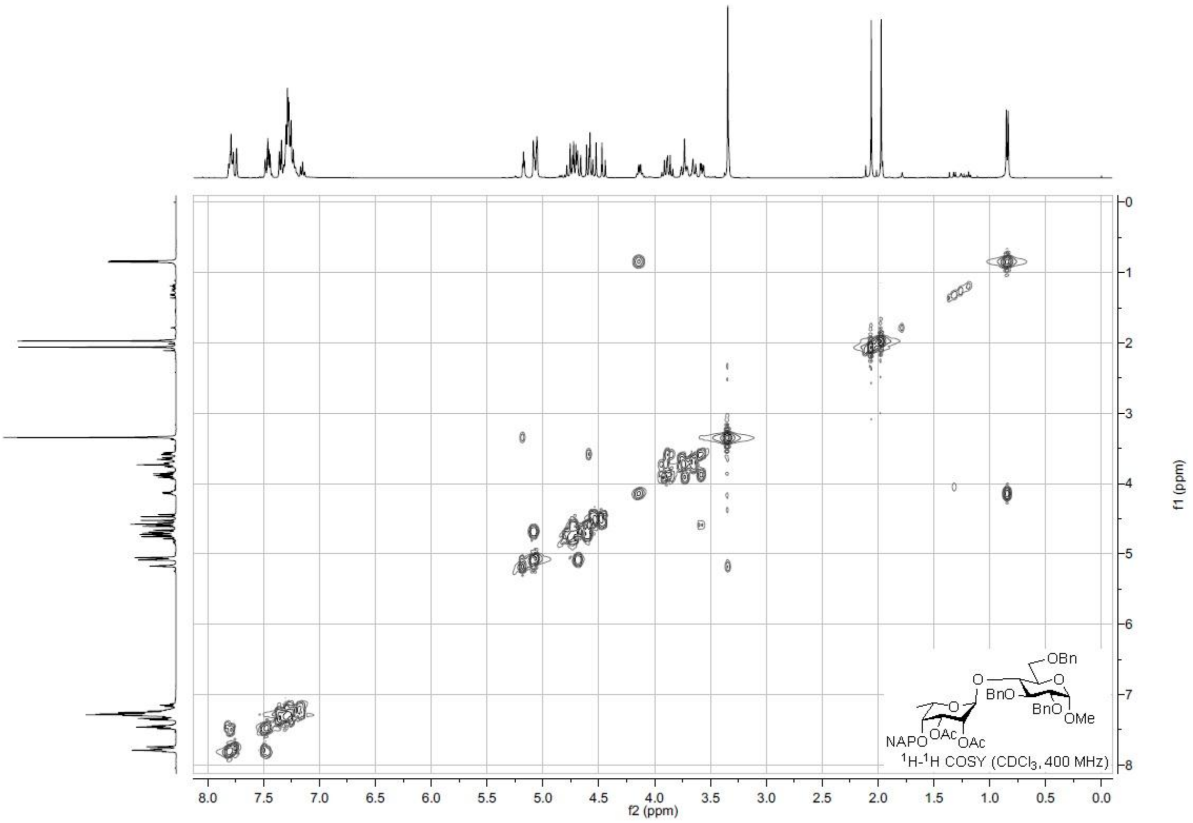
OBn
O
BnO
O
BnO
OMe
O
NAPO
OAc
OAc
$^1H$-$^1H$ COSY (CDCl$_3$, 400 MHz)
f2 (ppm)
f1 (ppm)

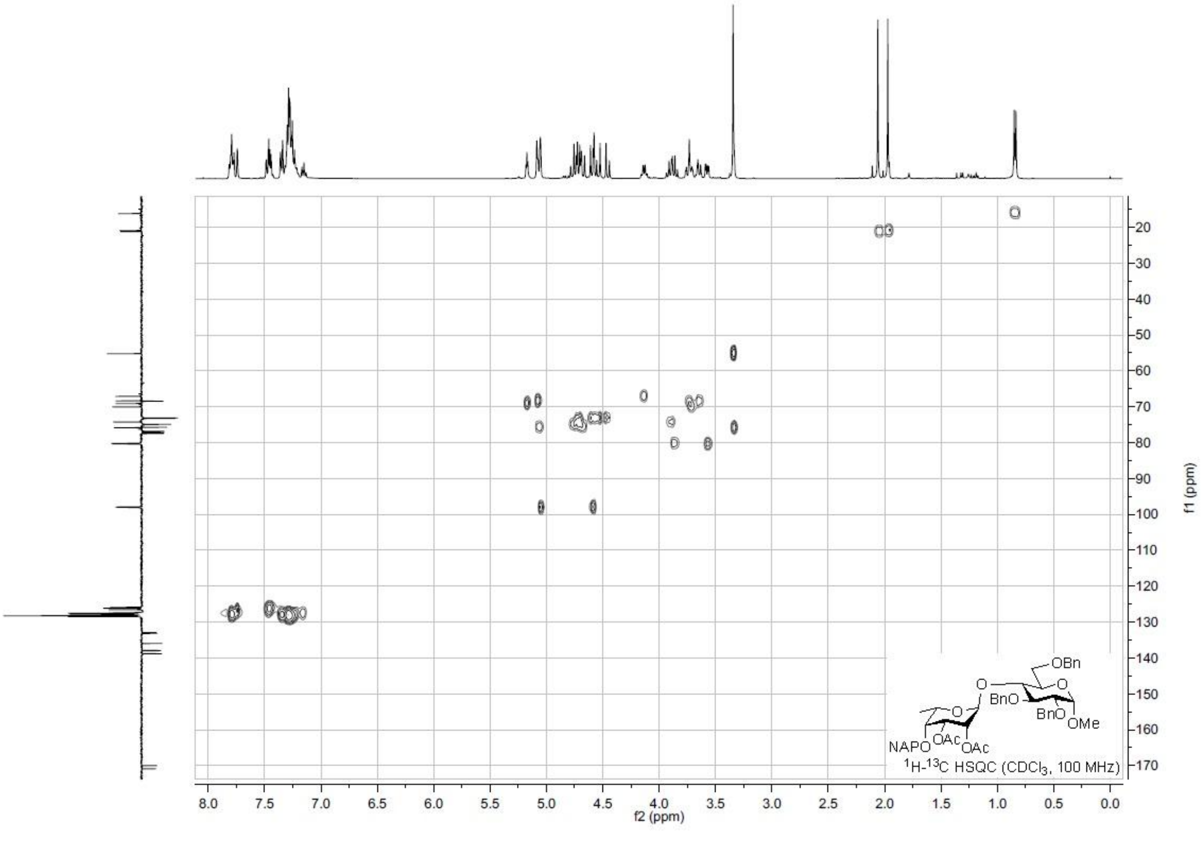
OBn
O
BnO
O
BnO
OMe
O
NAPO
OAc
OAc
$^1H$-$^{13}C$ HSQC (CDCl$_3$, 100 MHz)
f2 (ppm)
f1 (ppm)

# $^{1}$H and $^{13}$C NMR spectra of compound 12:

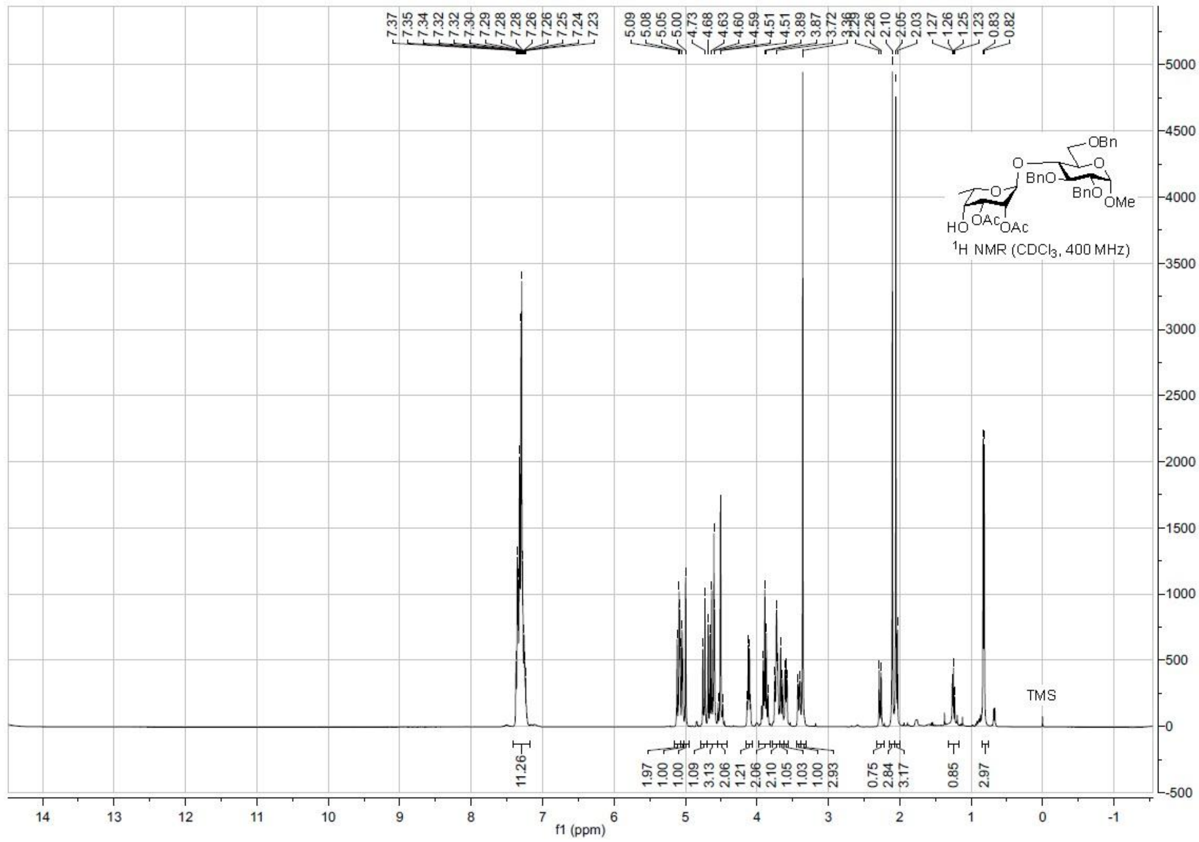


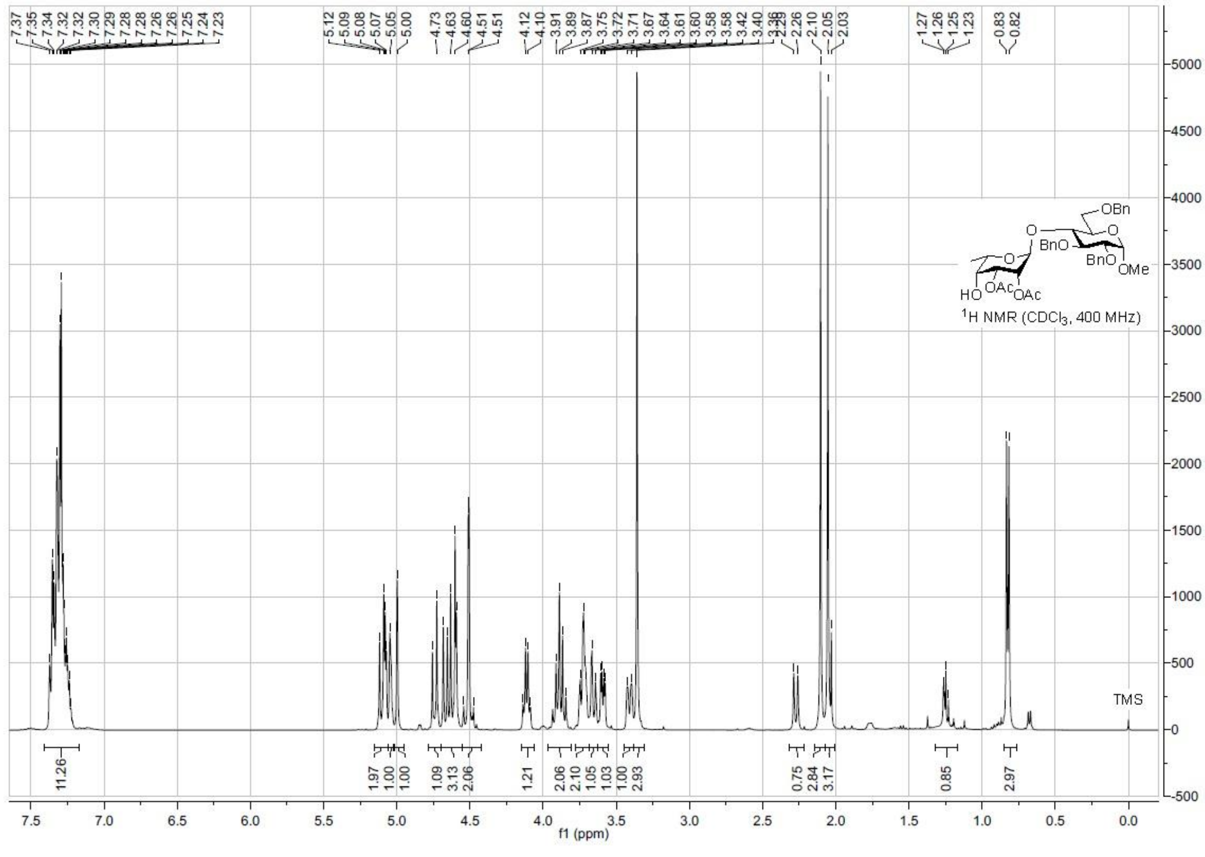

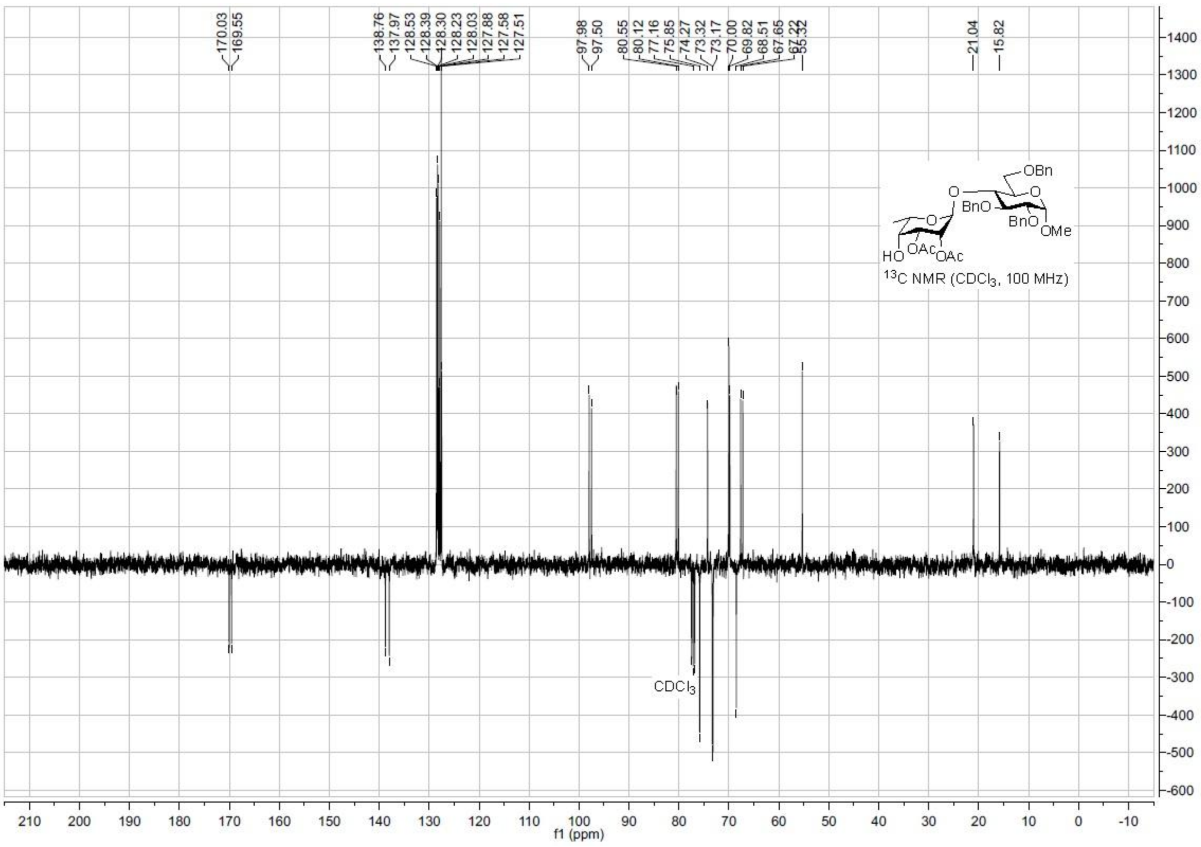
170.03
169.55
138.76
137.97
128.53
128.39
128.30
128.23
128.03
127.88
127.58
127.51
97.98
97.50
80.55
80.12
77.16
75.85
74.27
73.32
73.17
70.00
69.82
68.51
67.65
67.22
55.32
21.04
15.82
OBn
O
BnO
O
BnO
OMe
O
HO
OAc
OAc
$^{13}$C NMR (CDCl$_3$, 100 MHz)
CDCl$_3$
f1 (ppm)

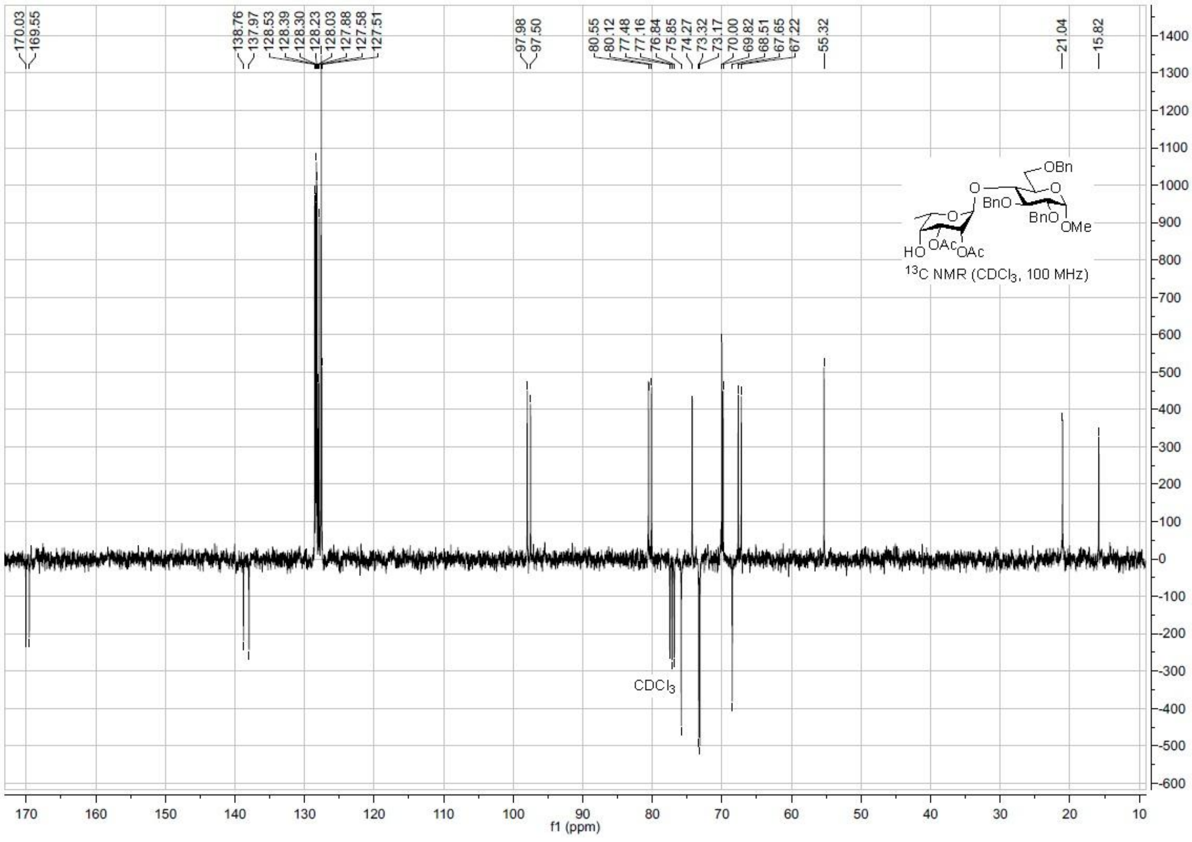
170.03
169.55
138.76
137.97
128.53
128.39
128.30
128.23
128.03
127.88
127.58
127.51
97.98
97.50
80.55
80.12
77.48
77.16
76.84
75.85
74.27
73.32
73.17
70.00
69.82
68.51
67.65
67.22
55.32
21.04
15.82
OBn
O
BnO
O
BnO
OMe
O
HO
OAc
OAc
$^{13}$C NMR (CDCl$_3$, 100 MHz)
CDCl$_3$
f1 (ppm)

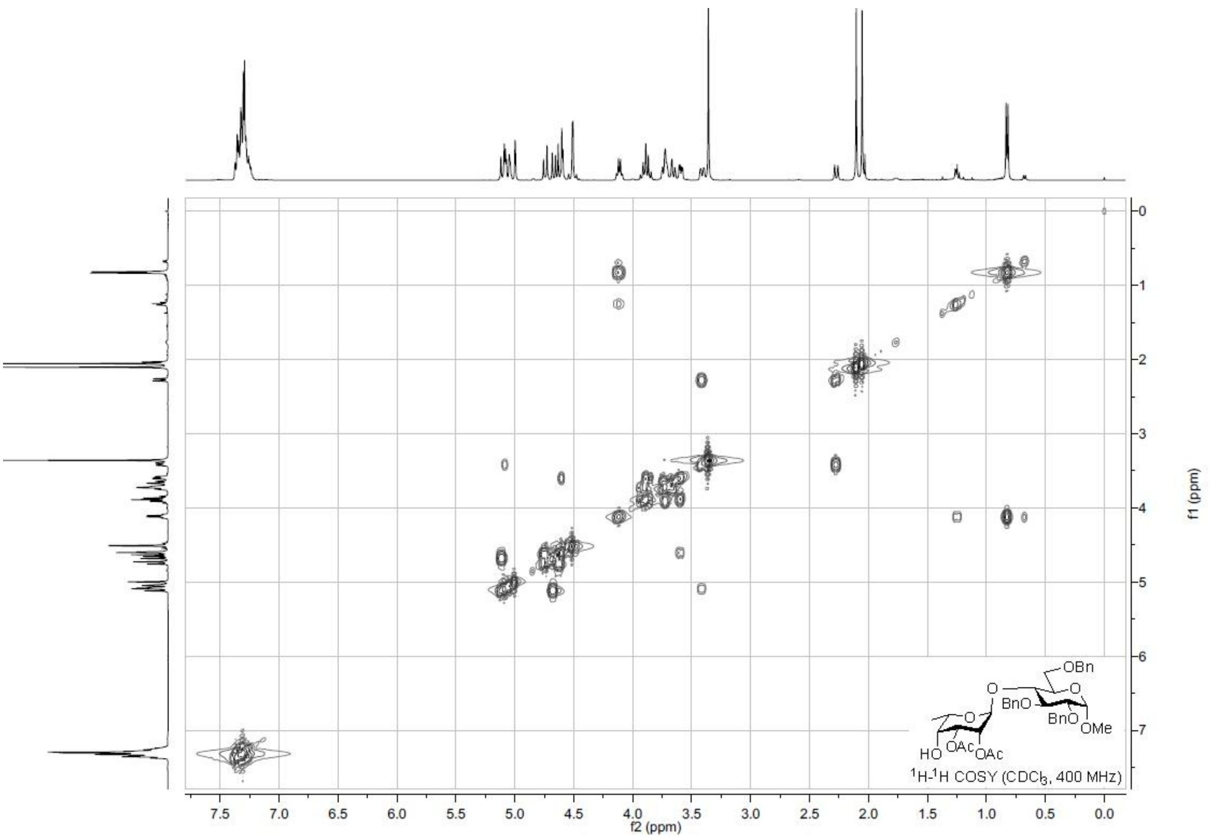

OBn
O
O
BnO
BnO
OMe
O
HO
OAc
OAc
$^1$H-$^1$H COSY (CDCl$_3$, 400 MHz)
f2 (ppm)
f1 (ppm)


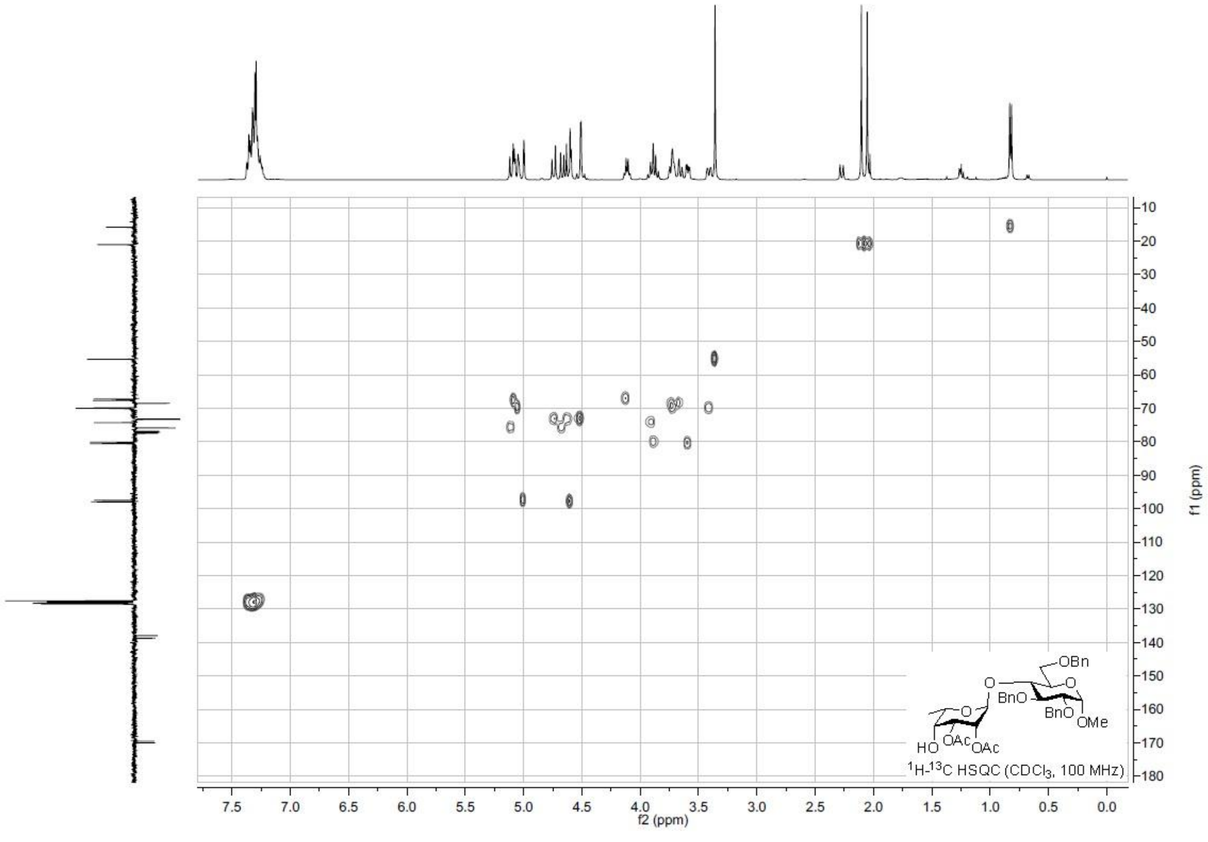

OBn
O
O
BnO
BnO
OMe
O
HO
OAc
OAc
$^1$H-$^{13}$C HSQC (CDCl$_3$, 100 MHz)
f2 (ppm)
f1 (ppm)

## $^{1}H$ and $^{13}C$ NMR spectra of compound 14α:

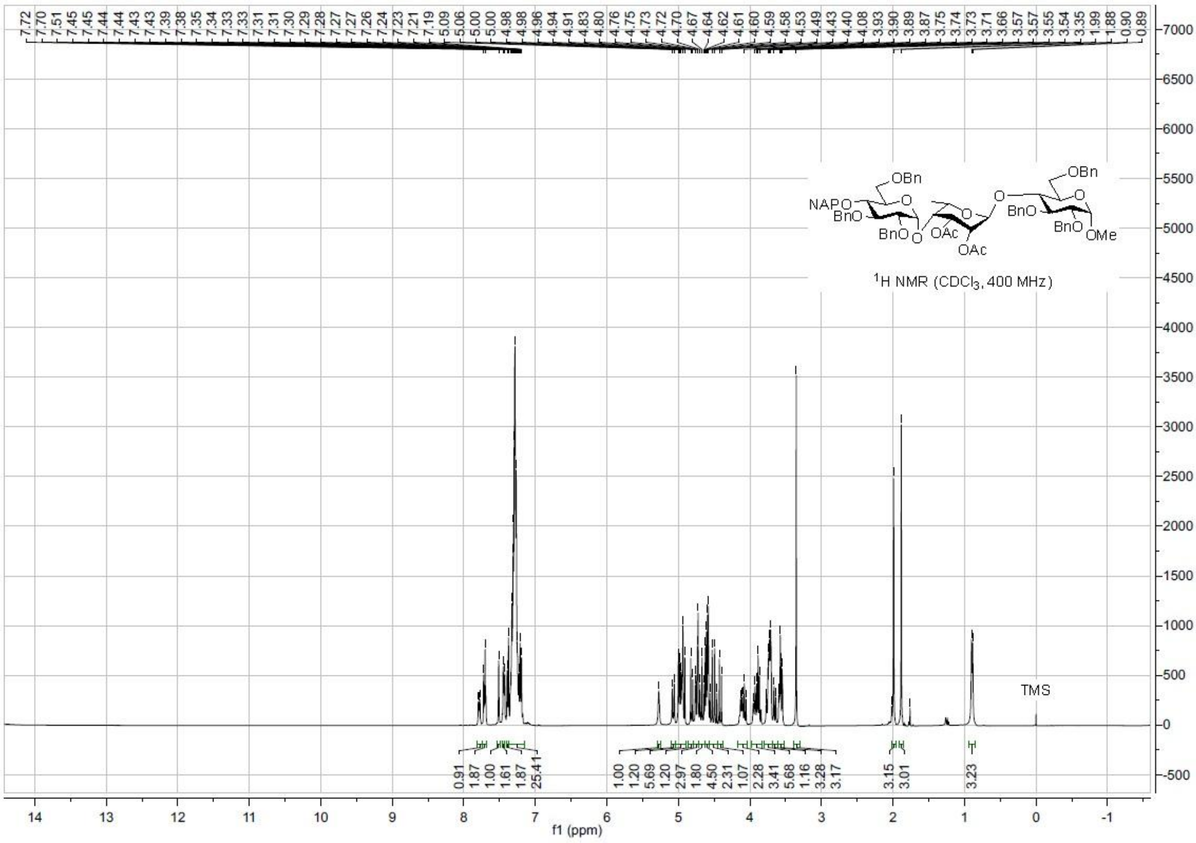


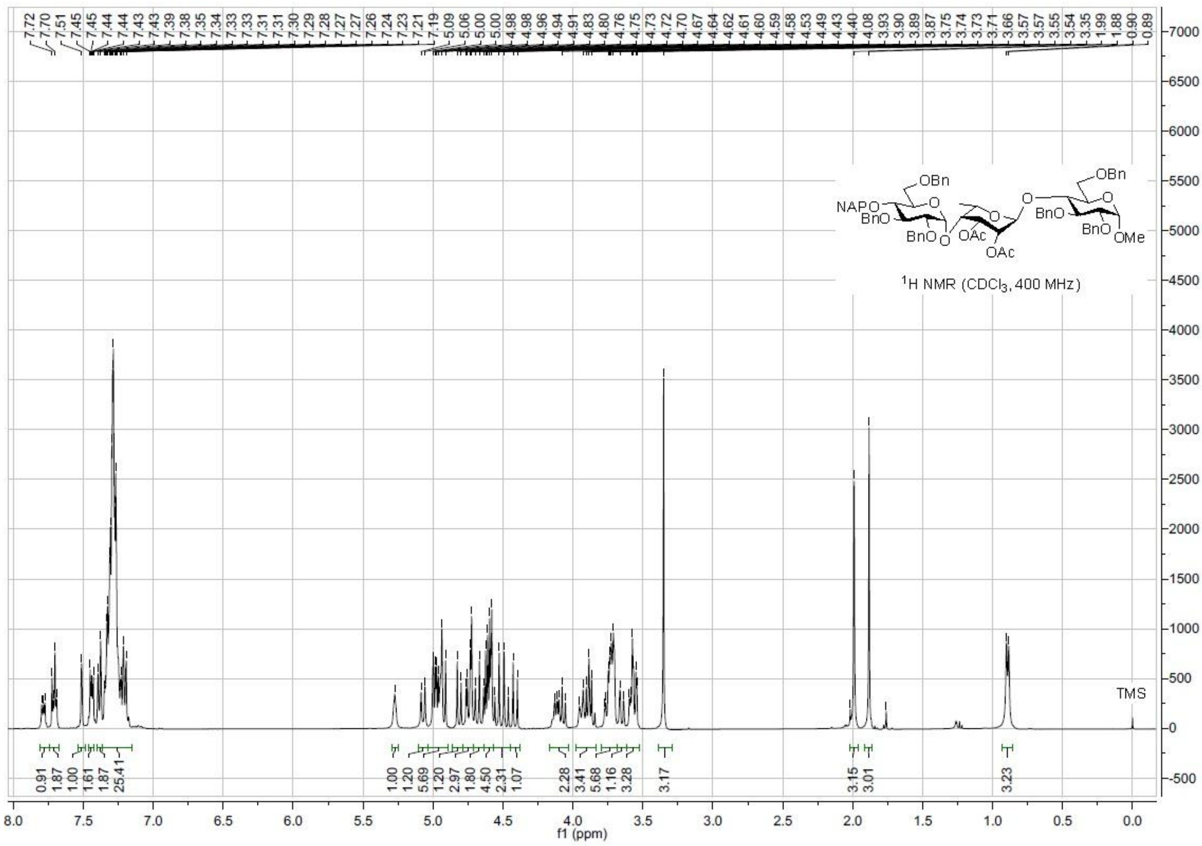

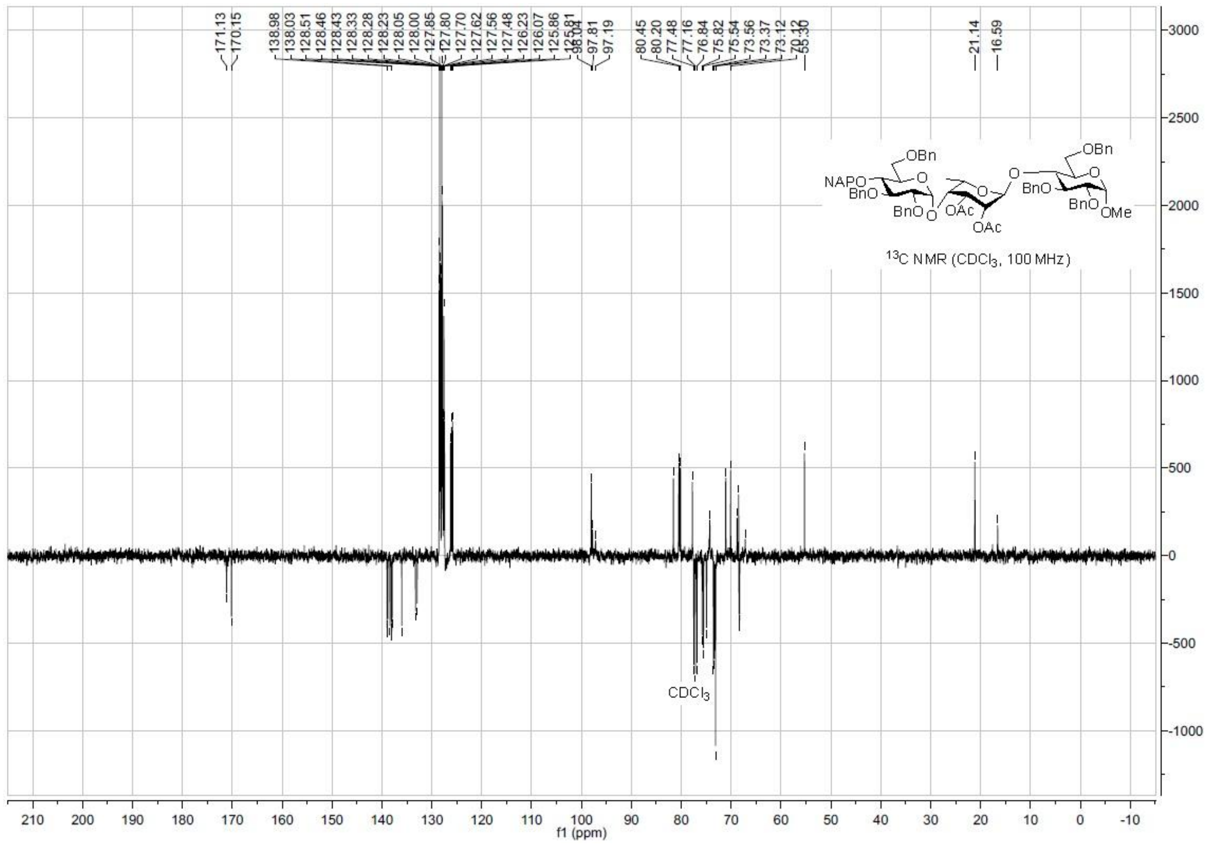
NAPO
BnO
BnO
OBn
OAc
OAc
BnO
BnO
OMe
OBn
$^{13}$C NMR (CDCl$_3$, 100 MHz)
CDCl$_3$
f1 (ppm)

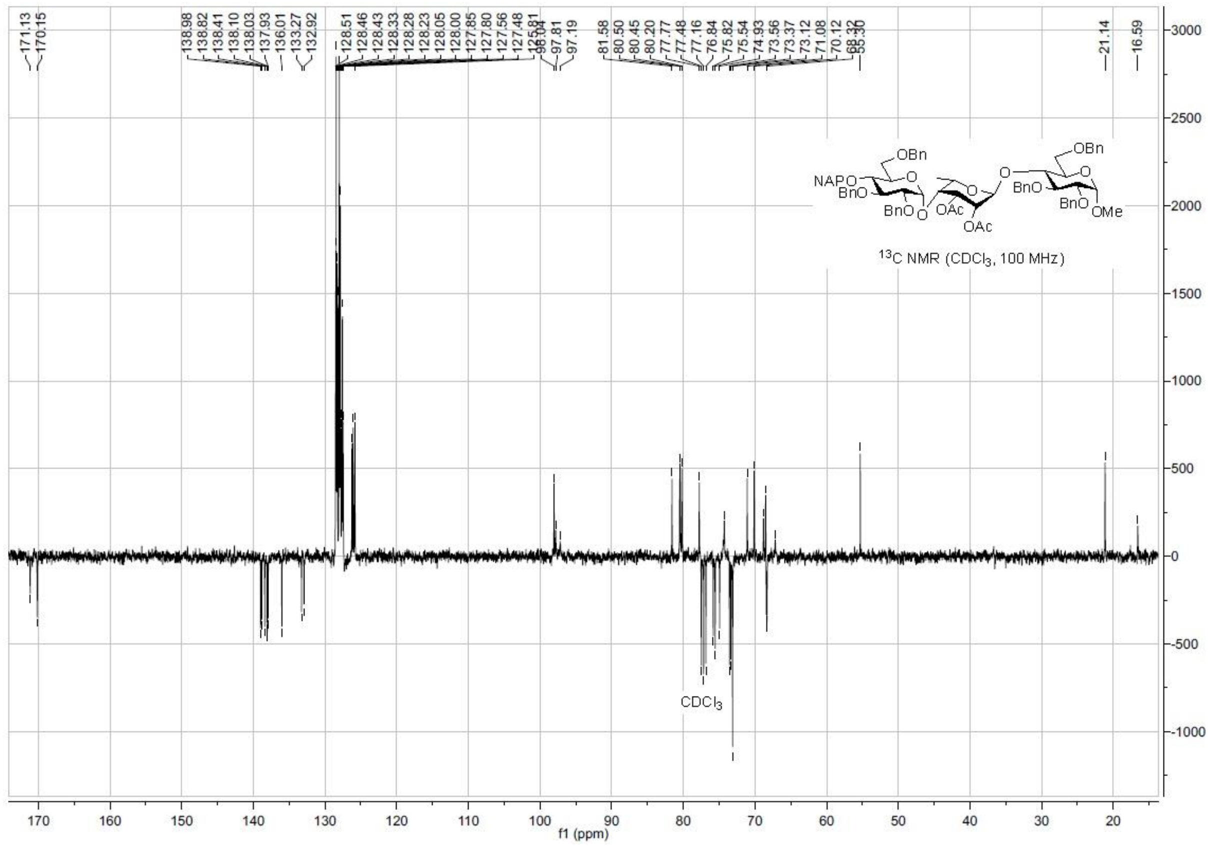
NAPO
BnO
BnO
OBn
OAc
OAc
BnO
BnO
OMe
OBn
$^{13}$C NMR (CDCl$_3$, 100 MHz)
CDCl$_3$
f1 (ppm)

## $^{1}H$ and $^{13}C$ NMR spectra of compound 15:

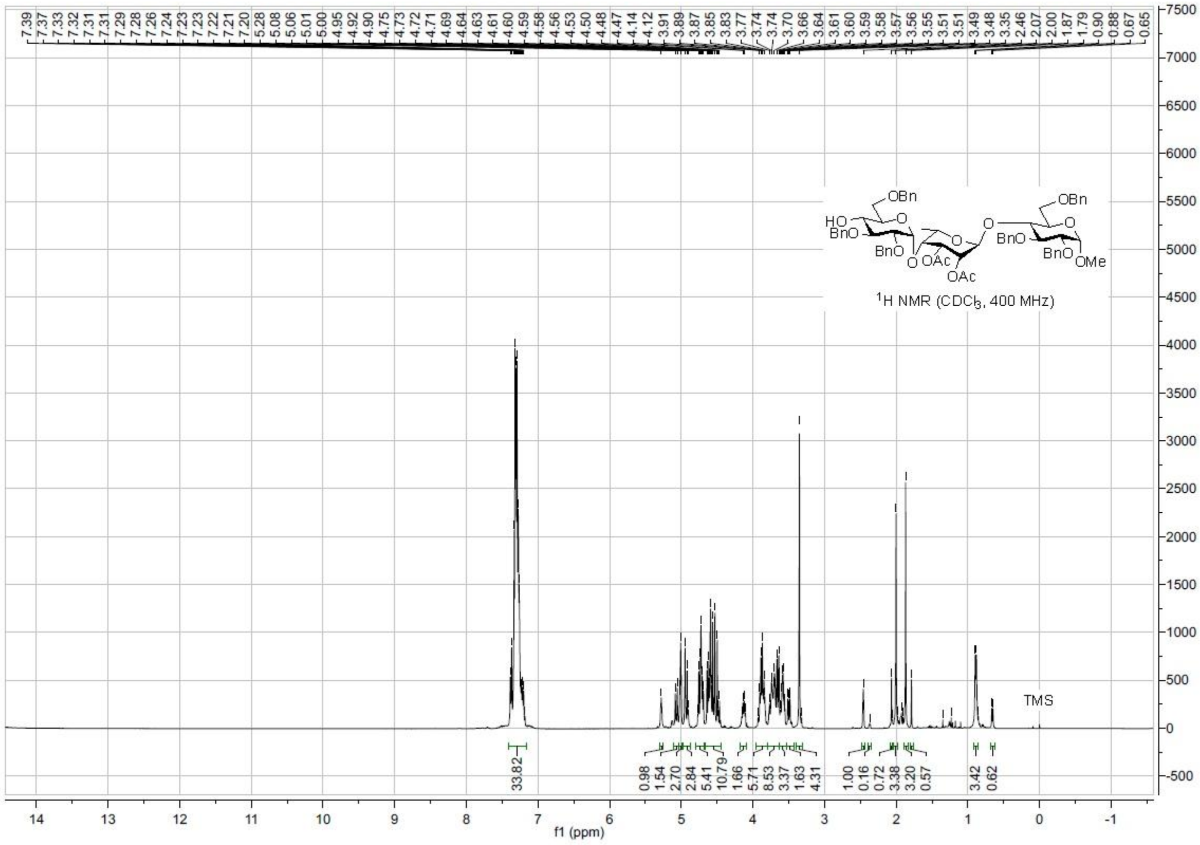


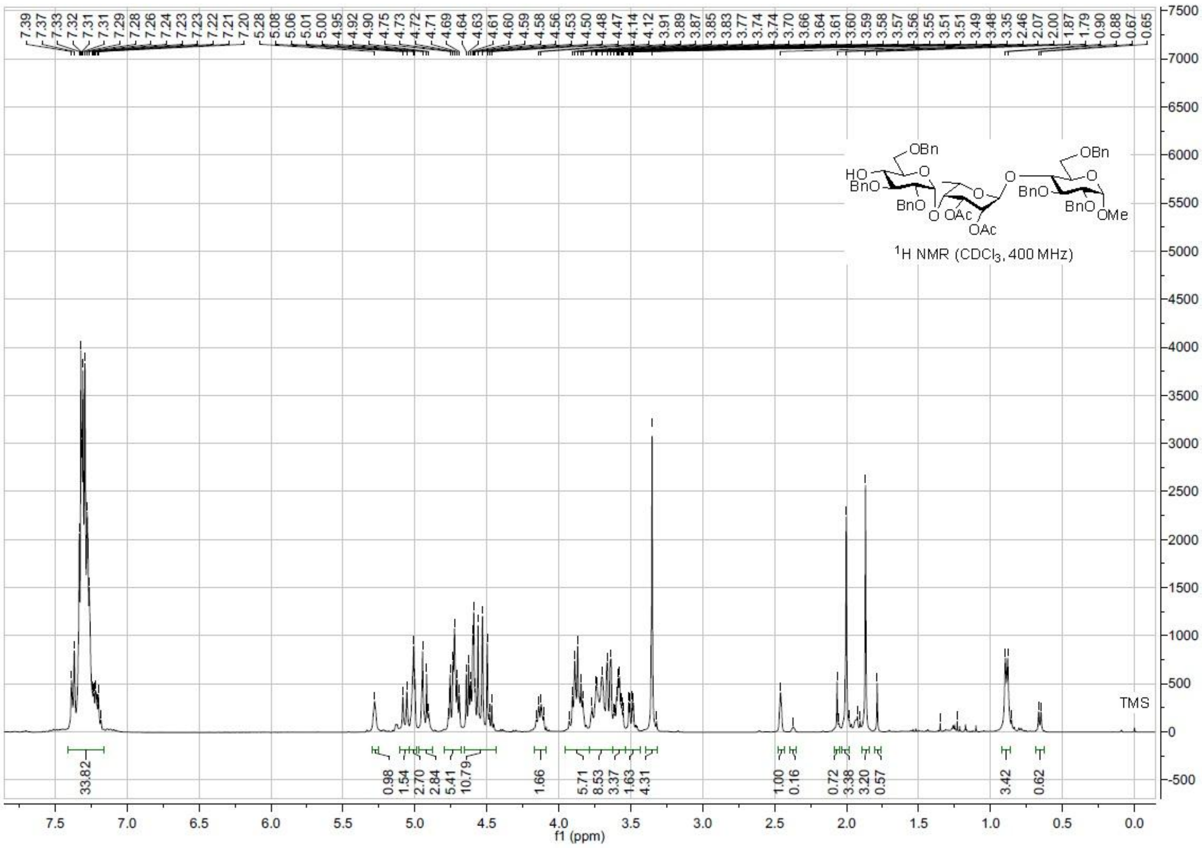

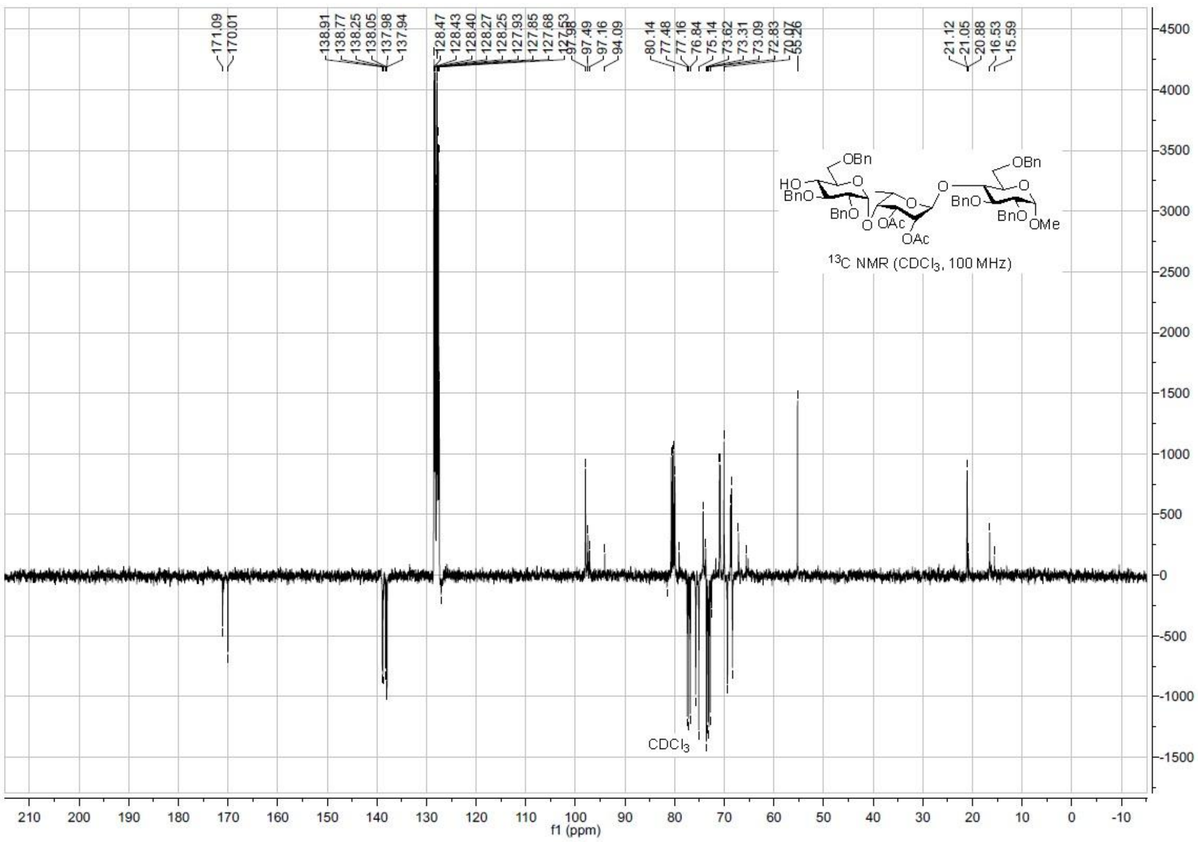

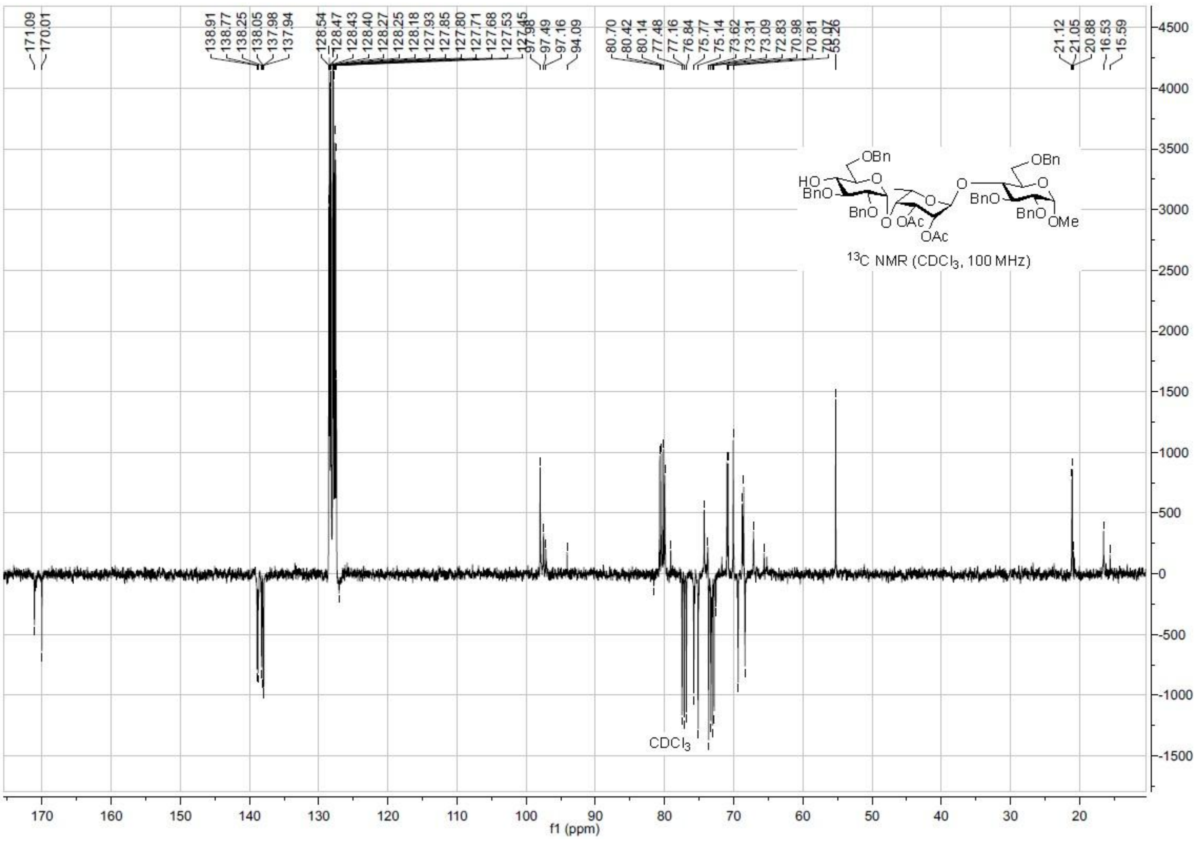

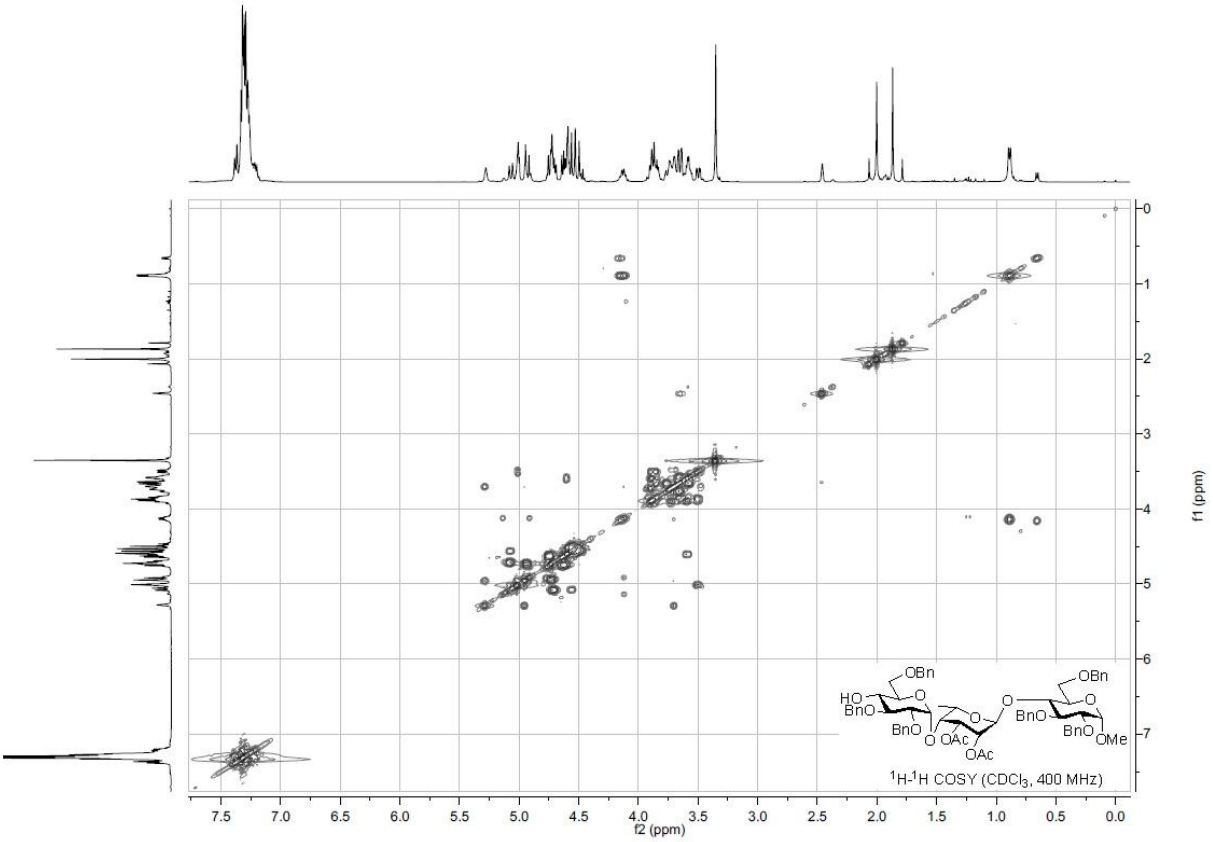

OBn
HO
BnO
BnO
O
OAc
OAc
O
BnO
OBn
BnO
OMe
$^1$H-$^1$H COSY (CDCl$_3$, 400 MHz)
f2 (ppm)
f1 (ppm)


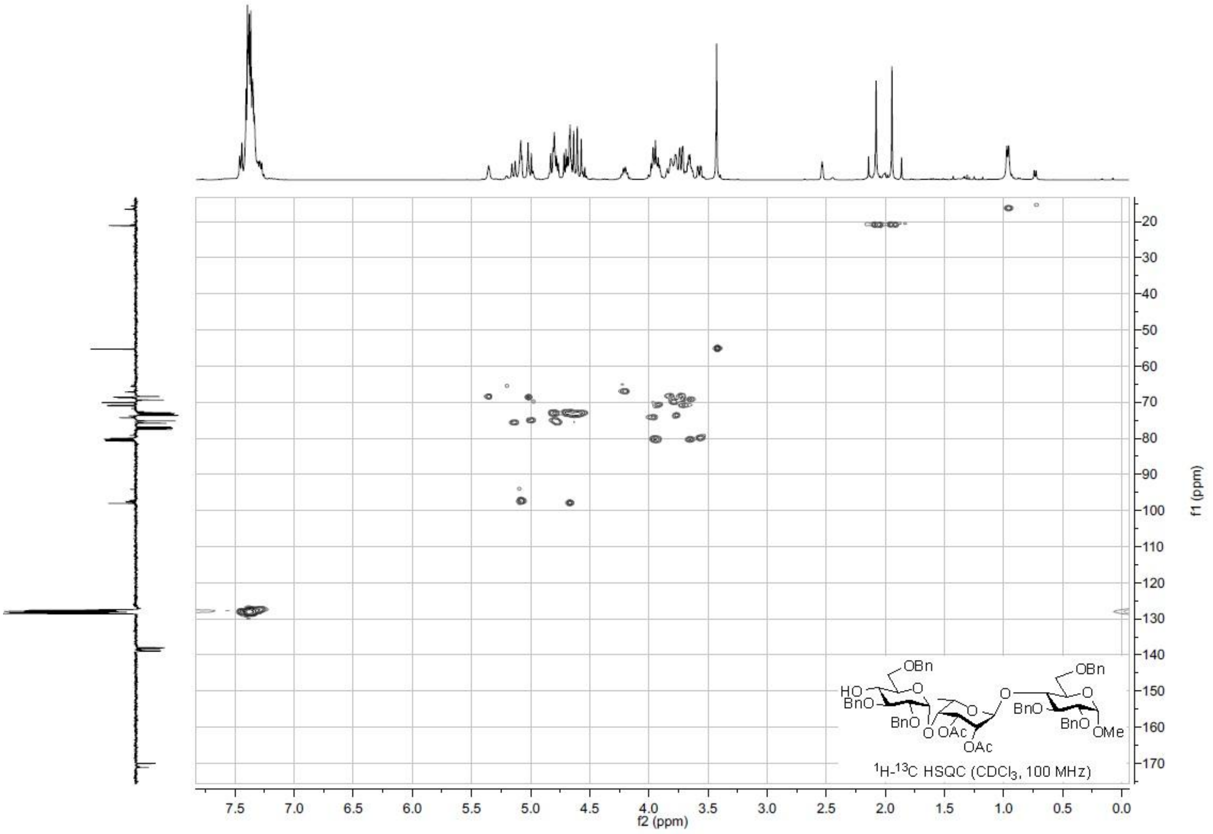

OBn
HO
BnO
BnO
O
OAc
OAc
O
BnO
OBn
BnO
OMe
$^1$H-$^{13}$C HSQC (CDCl$_3$, 100 MHz)
f2 (ppm)
f1 (ppm)

## $^{1}H$ and $^{13}C$ NMR spectra of compound 16:

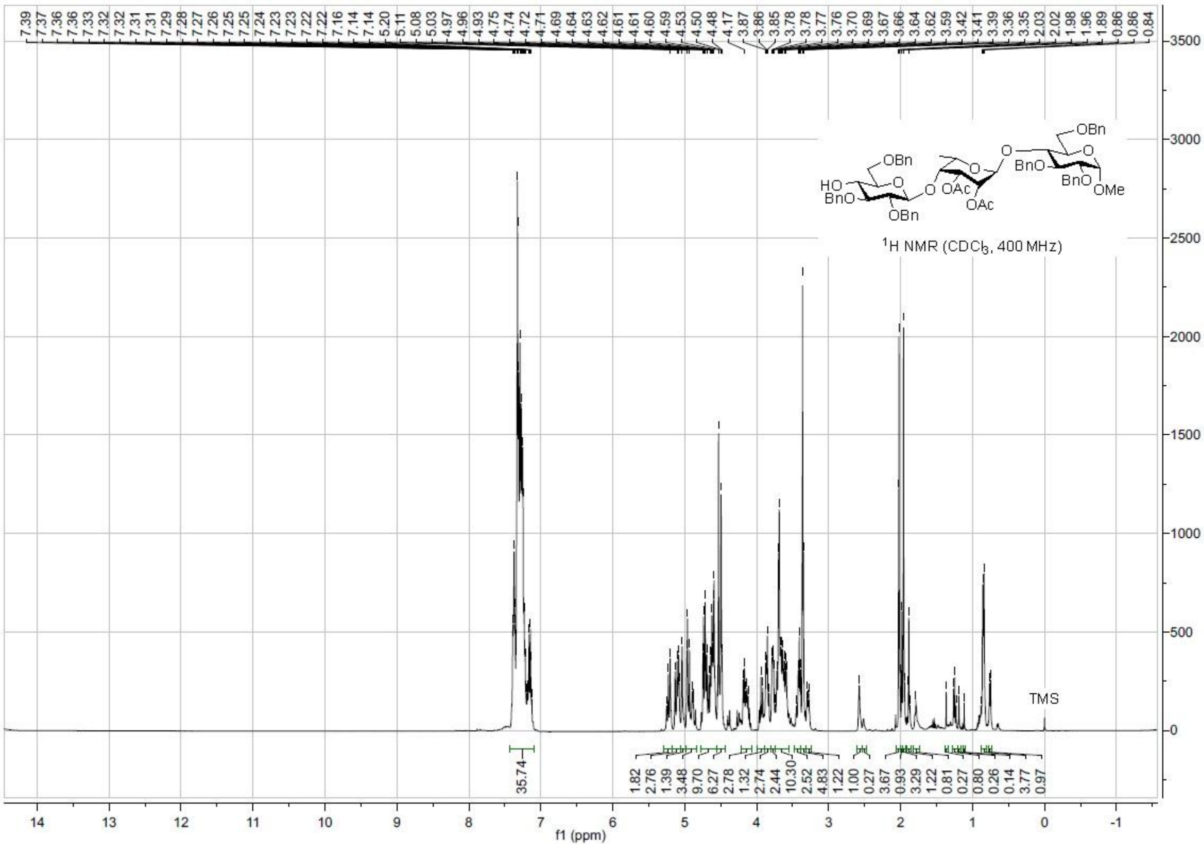


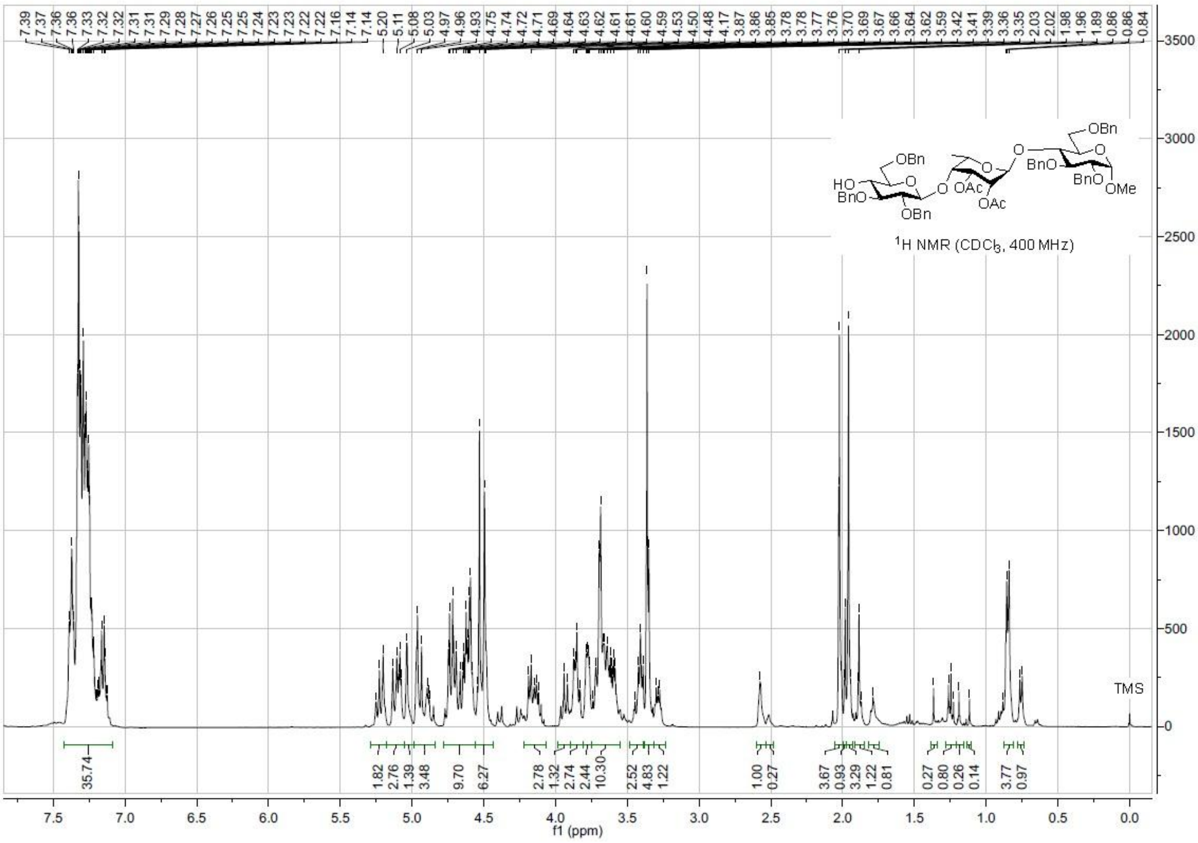

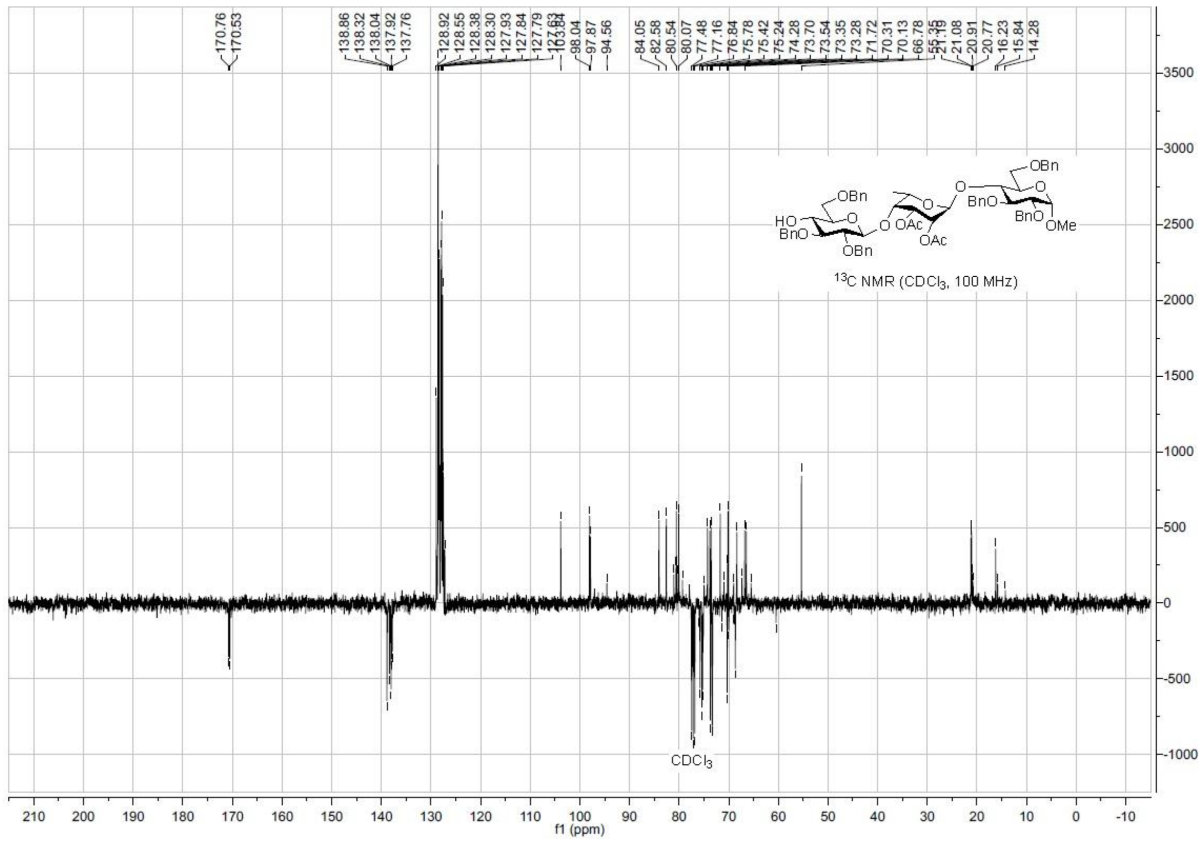

<sup></sup>
13C NMR (CDCl3, 100 MHz)
CDCl3
f1 (ppm)


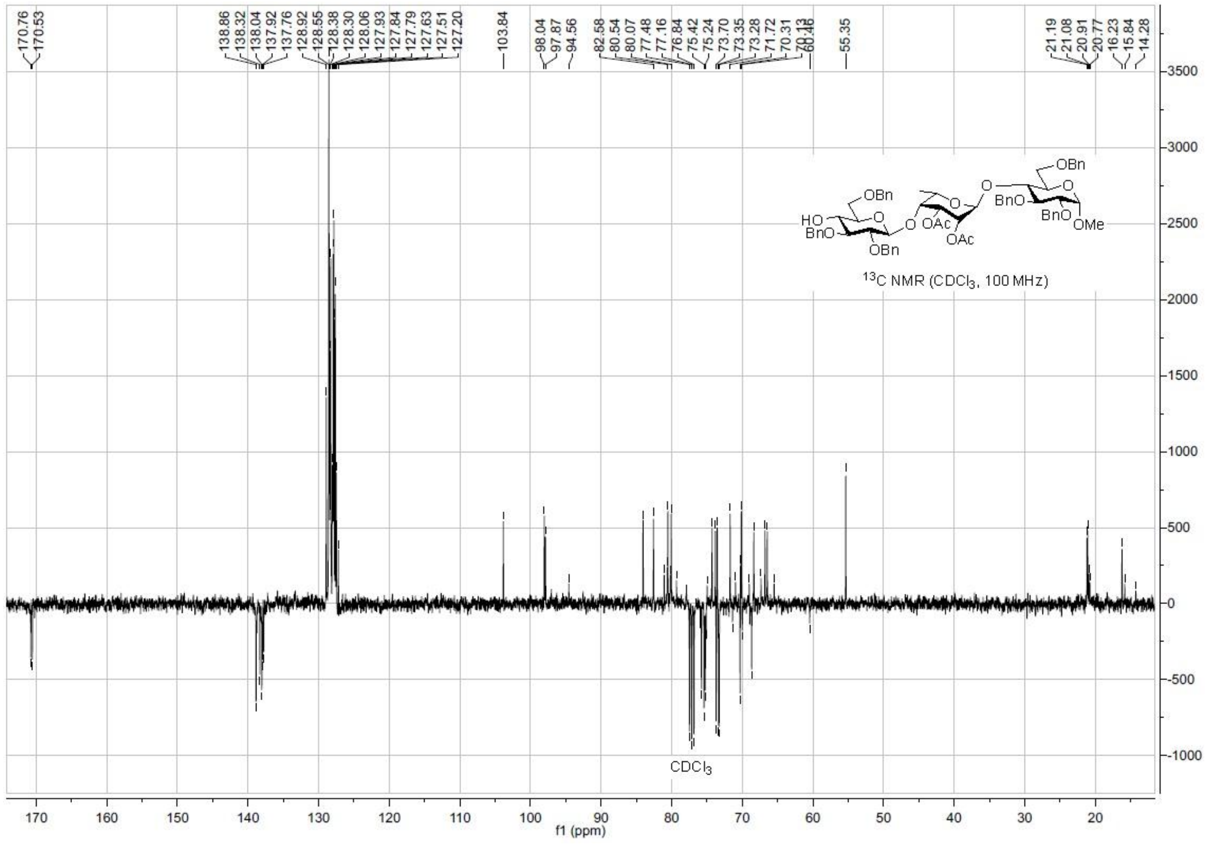

13C NMR (CDCl3, 100 MHz)
CDCl3
f1 (ppm)

**$^{1}$H and $^{13}$C NMR spectra of compound 17:**

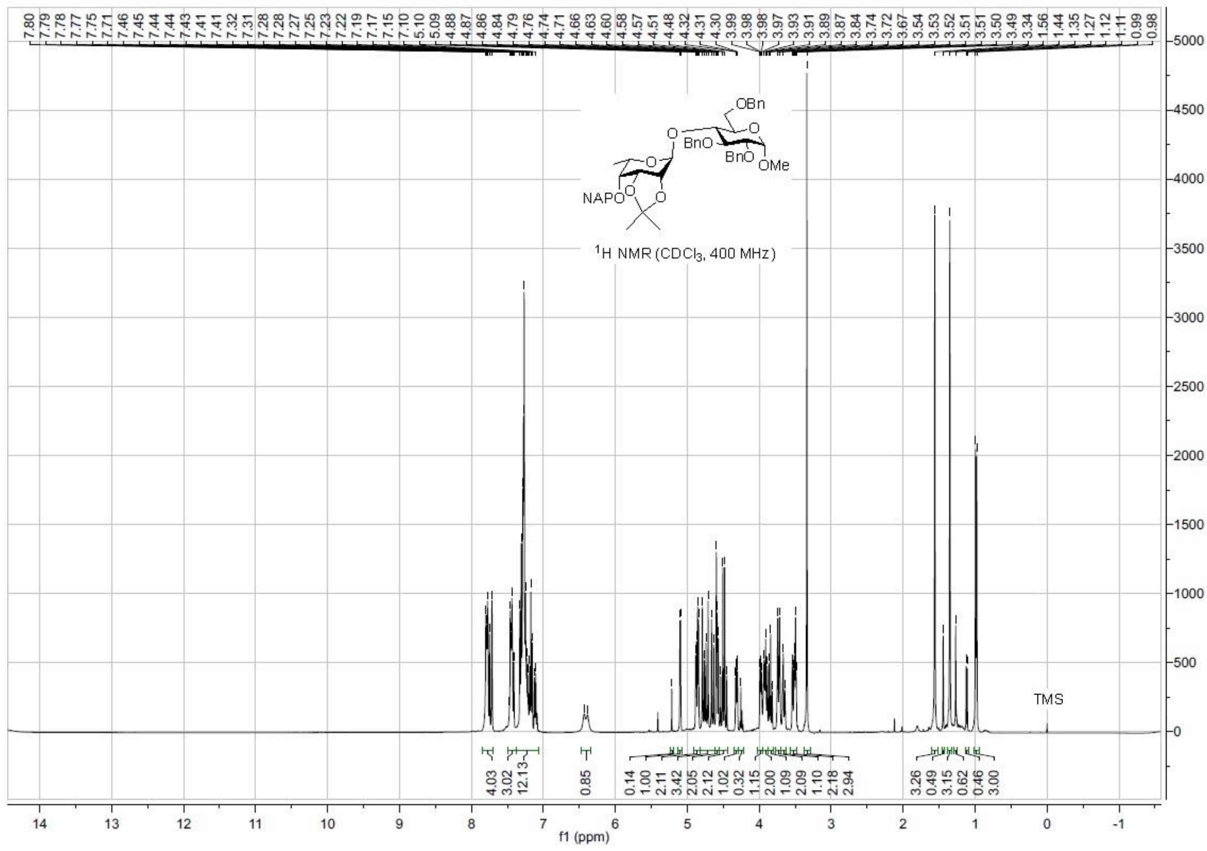

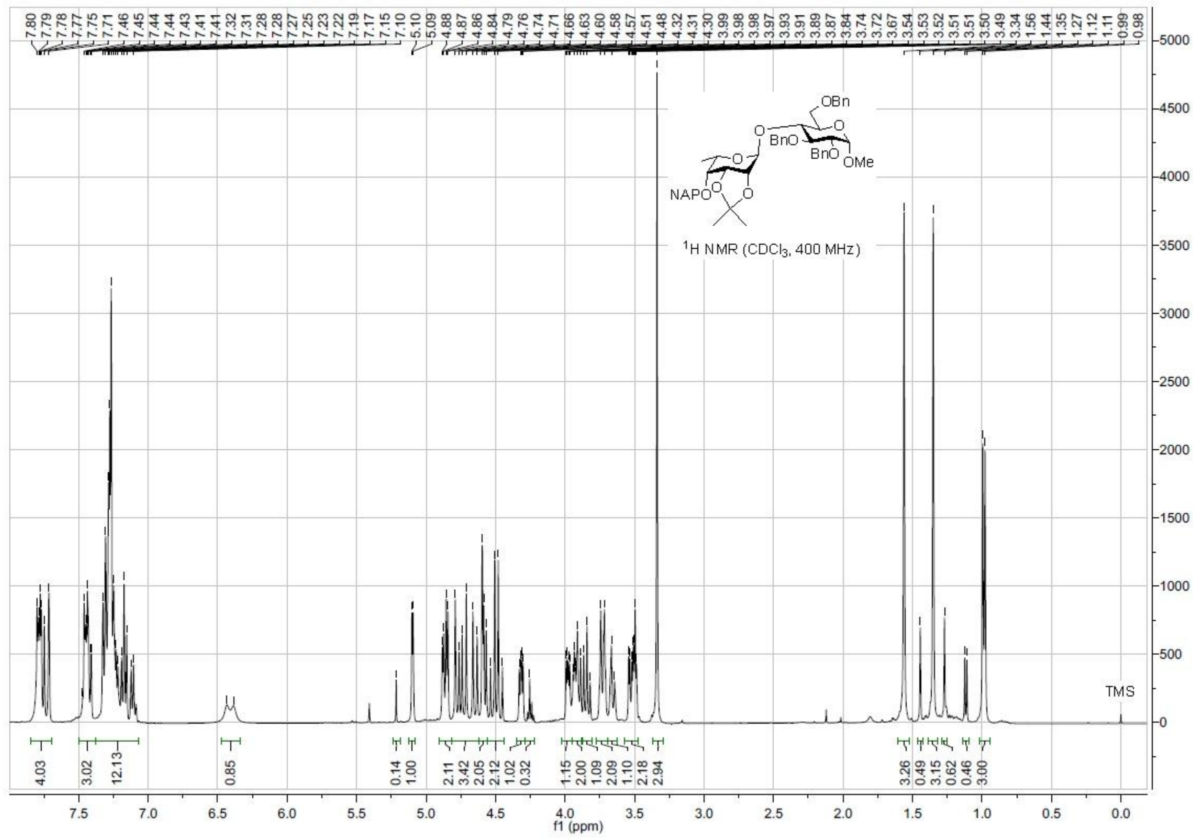

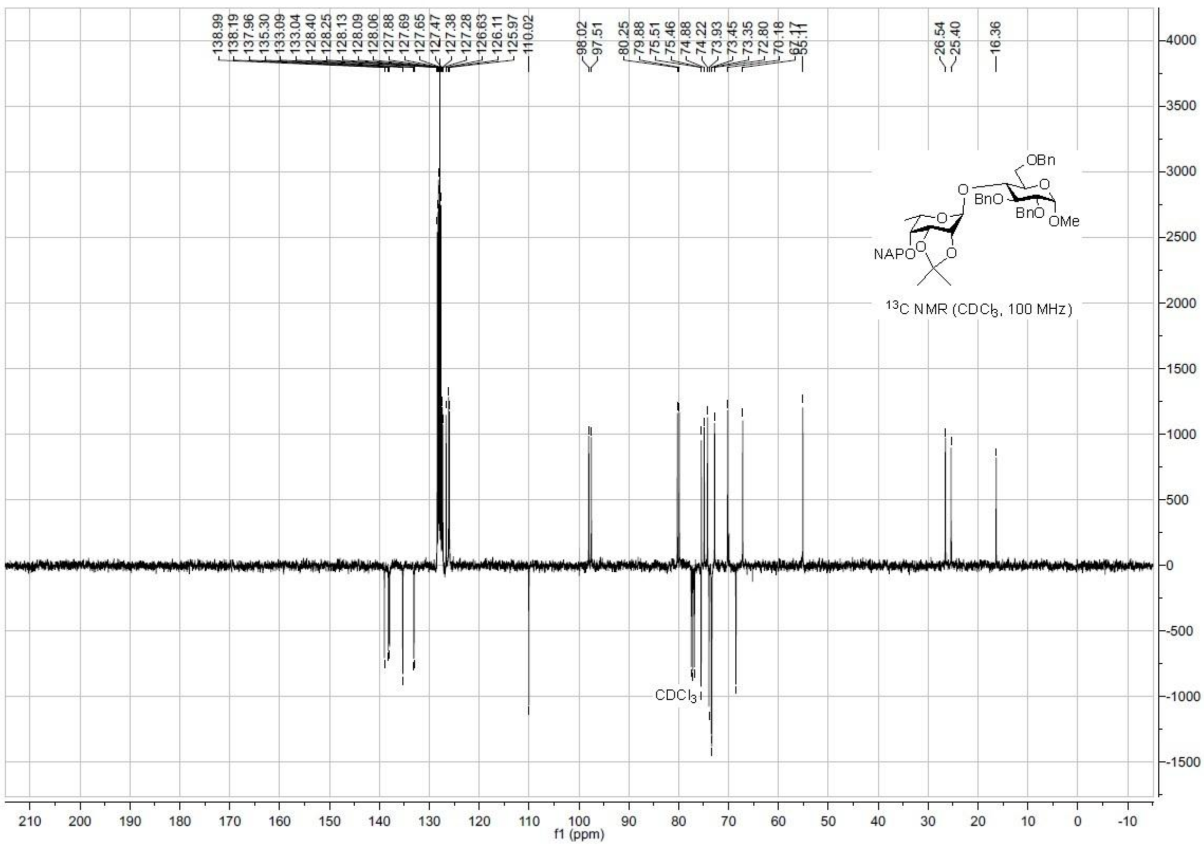

$^{13}C$ NMR (CDCl$_3$, 100 MHz)
CDCl$_3$
f1 (ppm)


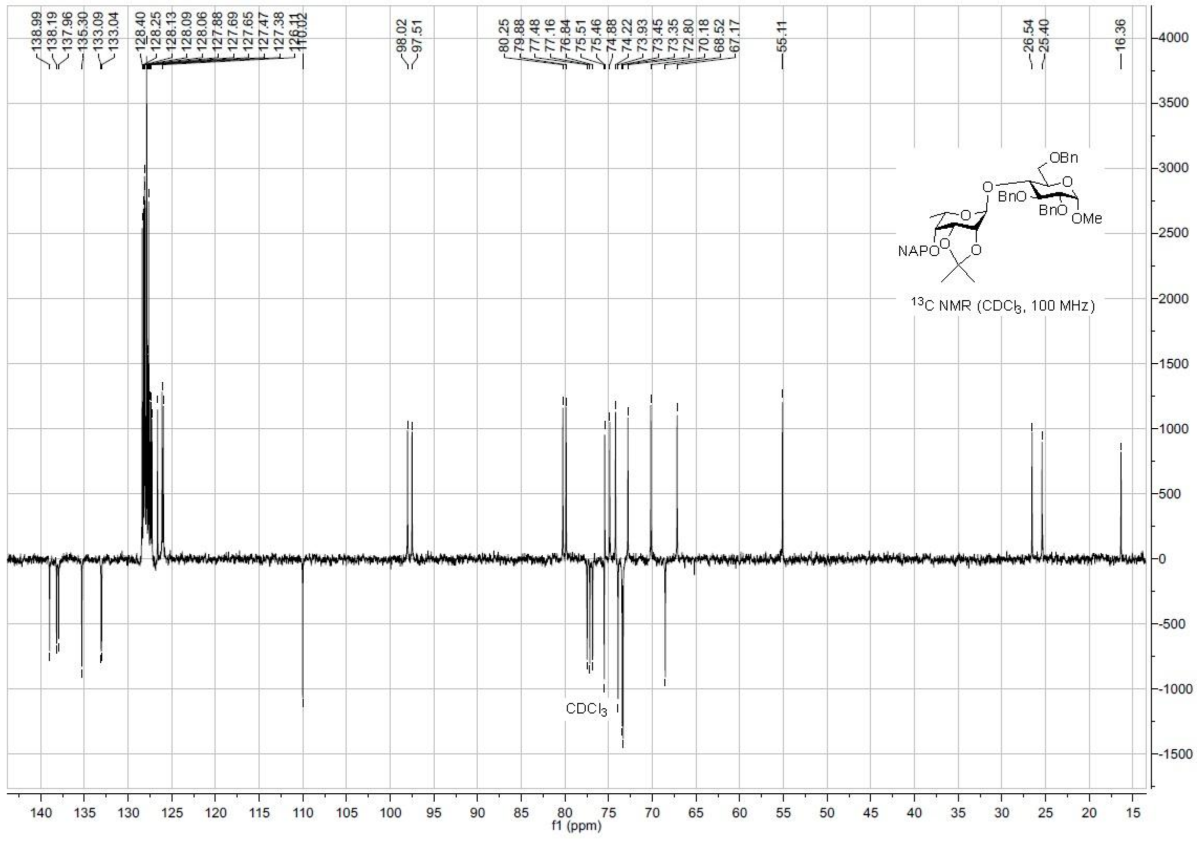

$^{13}C$ NMR (CDCl$_3$, 100 MHz)
CDCl$_3$
f1 (ppm)

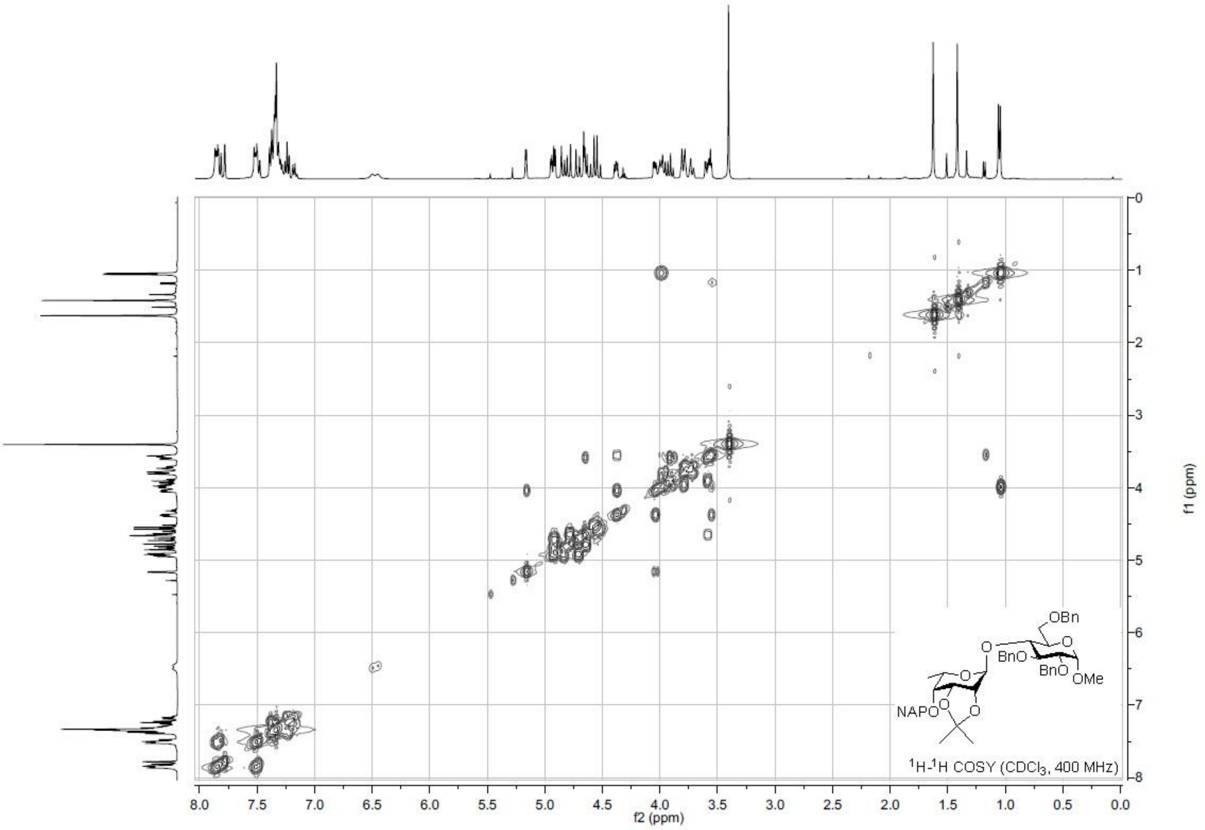
OBn
O
BnO
BnO
OMe
NAPO
O
O
O
O
¹H-¹H COSY (CDCl₃, 400 MHz)
f2 (ppm)
f1 (ppm)

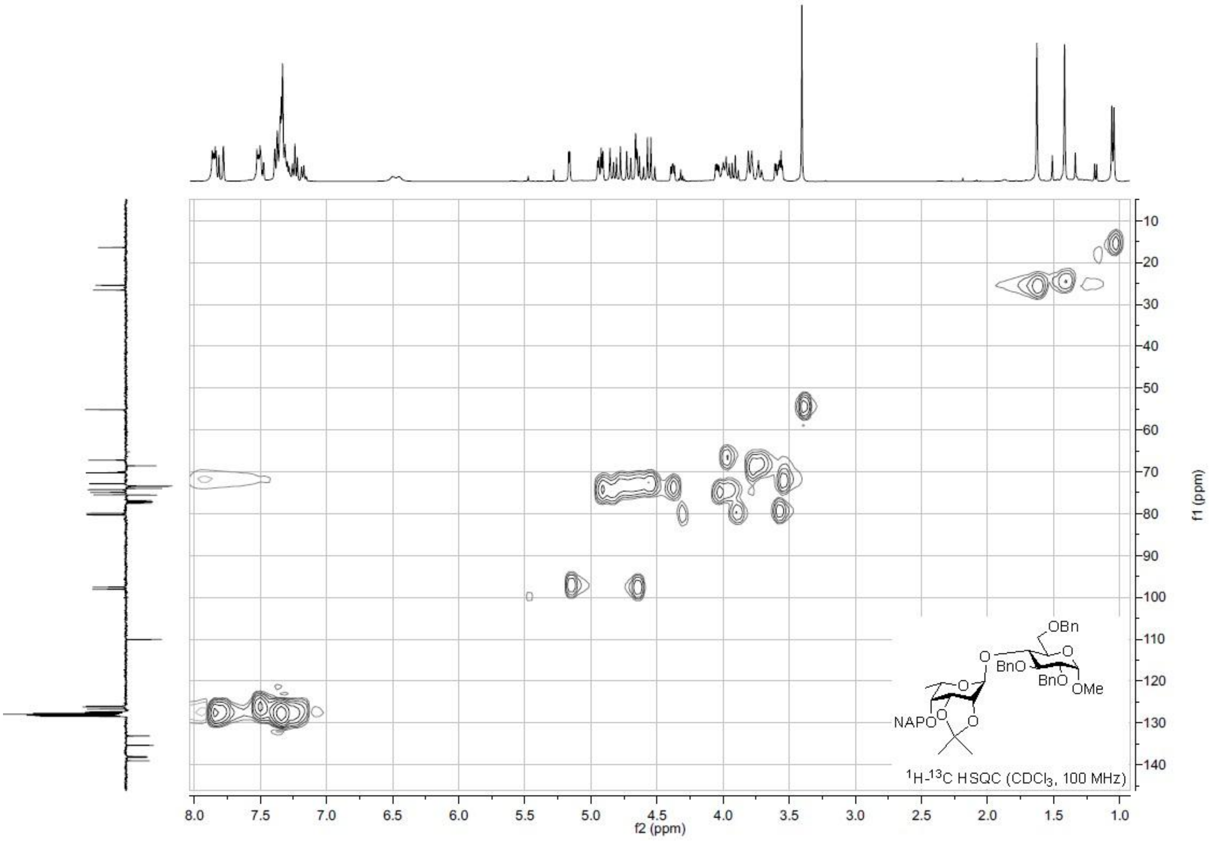
OBn
O
BnO
BnO
OMe
NAPO
O
O
O
O
¹H-¹³C HSQC (CDCl₃, 100 MHz)
f2 (ppm)
f1 (ppm)

**$^{1}H$ and $^{13}C$ NMR spectra of compound 18:**

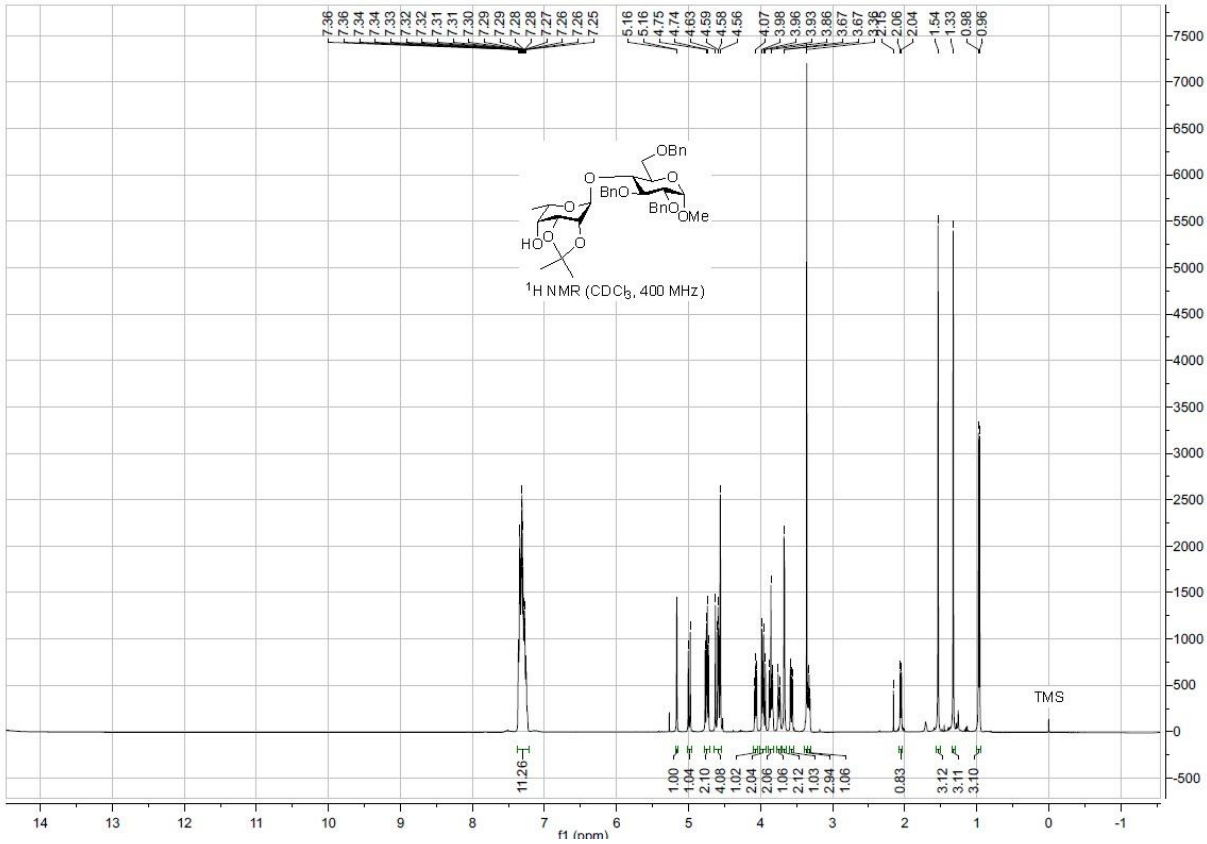


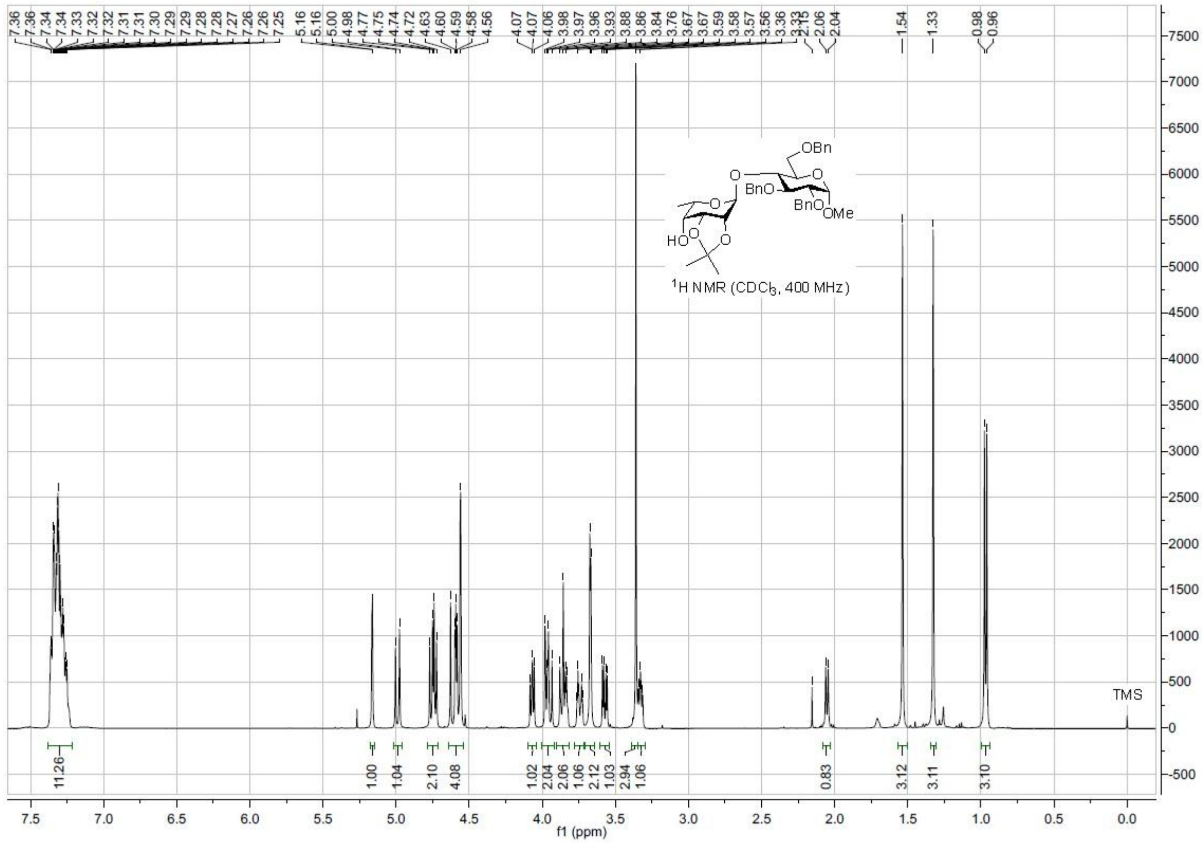

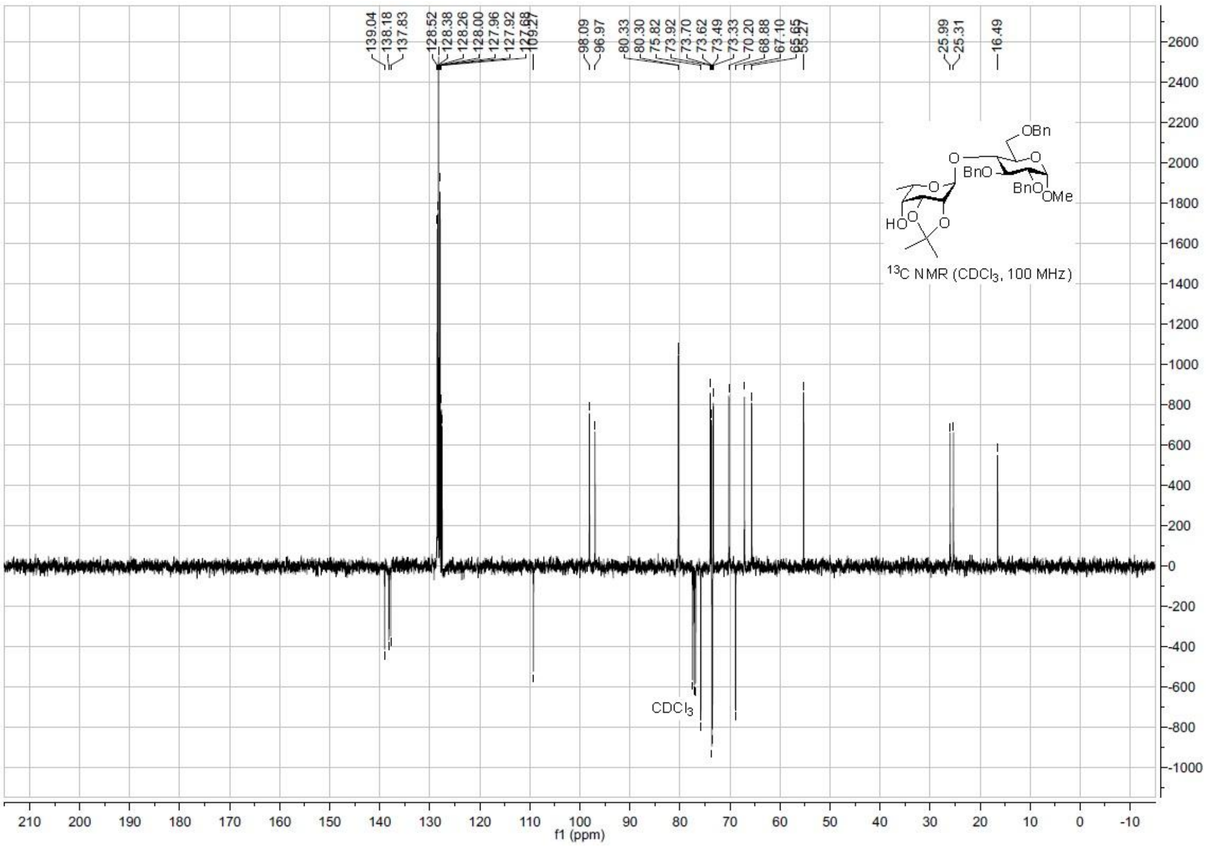
OBn
BnO
BnO
OMe
HO
$^{13}$C NMR (CDCl$_3$, 100 MHz)
CDCl$_3$
f1 (ppm)

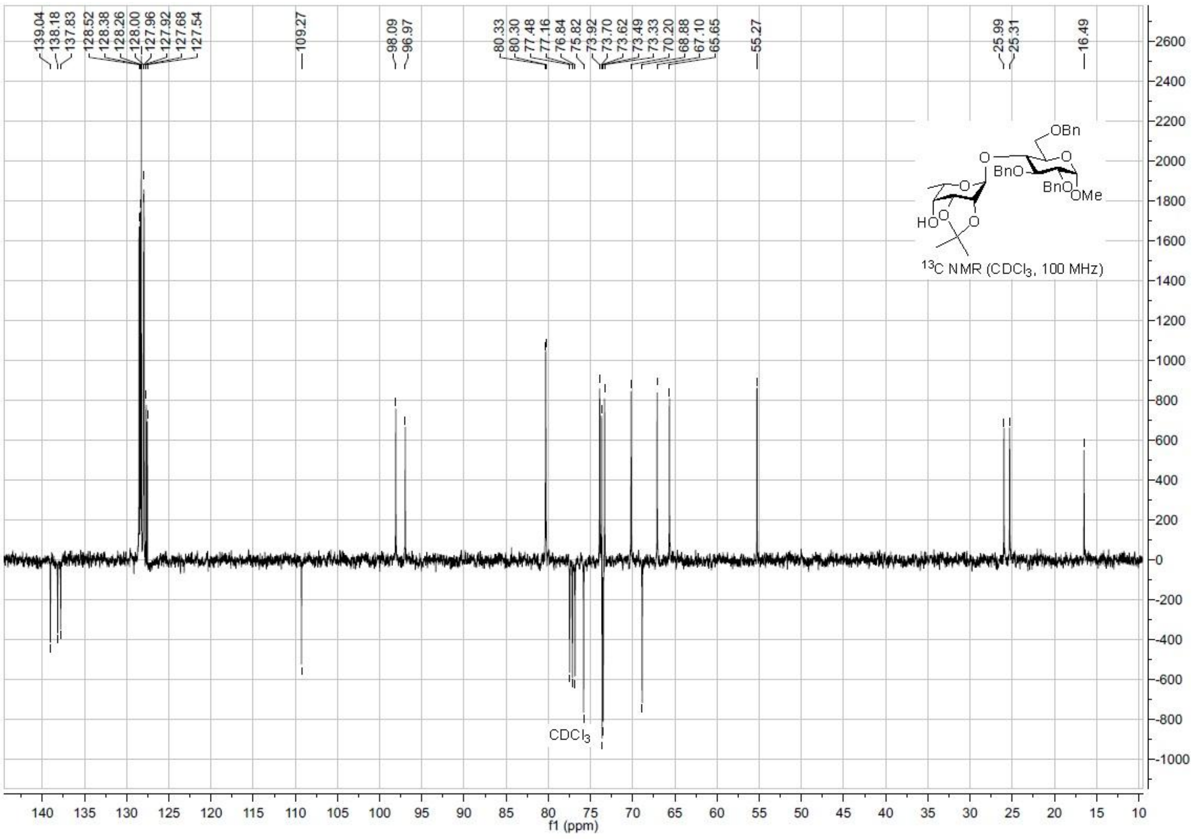
OBn
BnO
BnO
OMe
HO
$^{13}$C NMR (CDCl$_3$, 100 MHz)
CDCl$_3$
f1 (ppm)

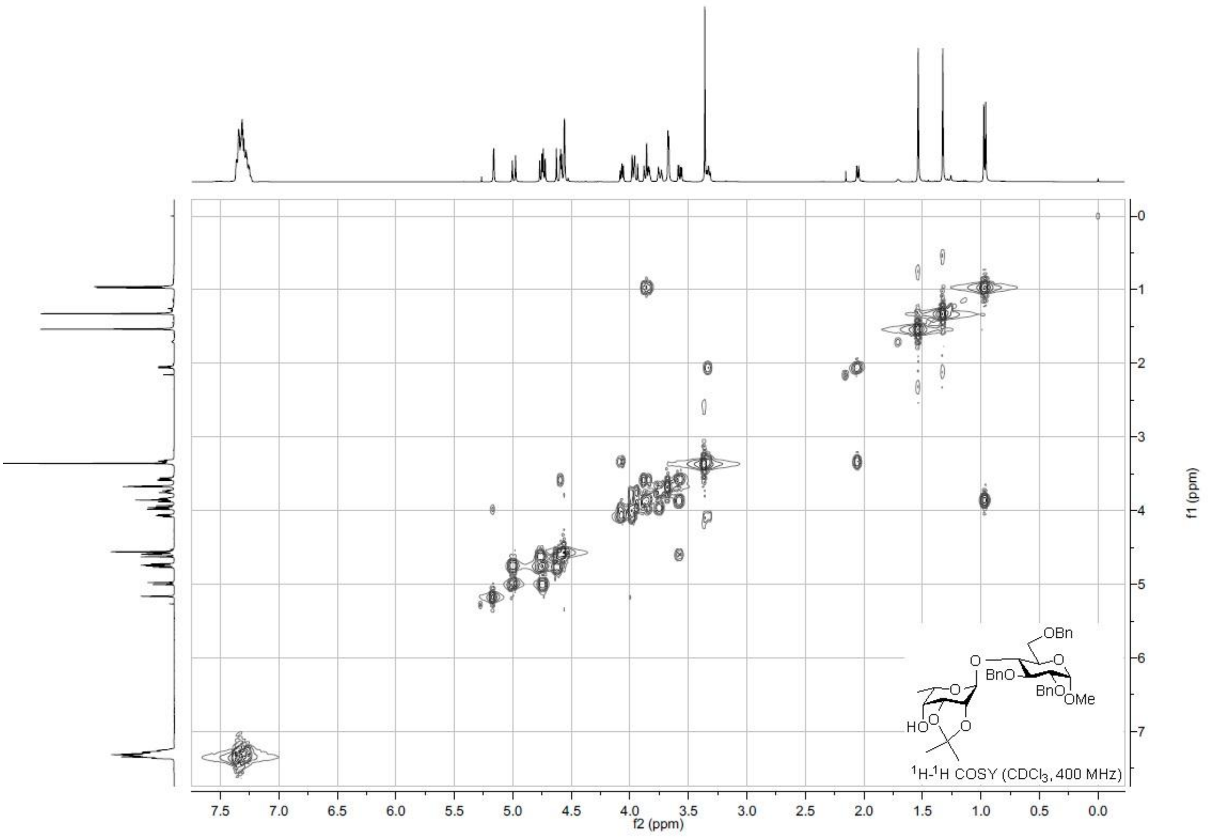
OBn
BnO
BnO
OMe
HO
1H-1H COSY (CDCl3, 400 MHz)
f2 (ppm)
f1 (ppm)

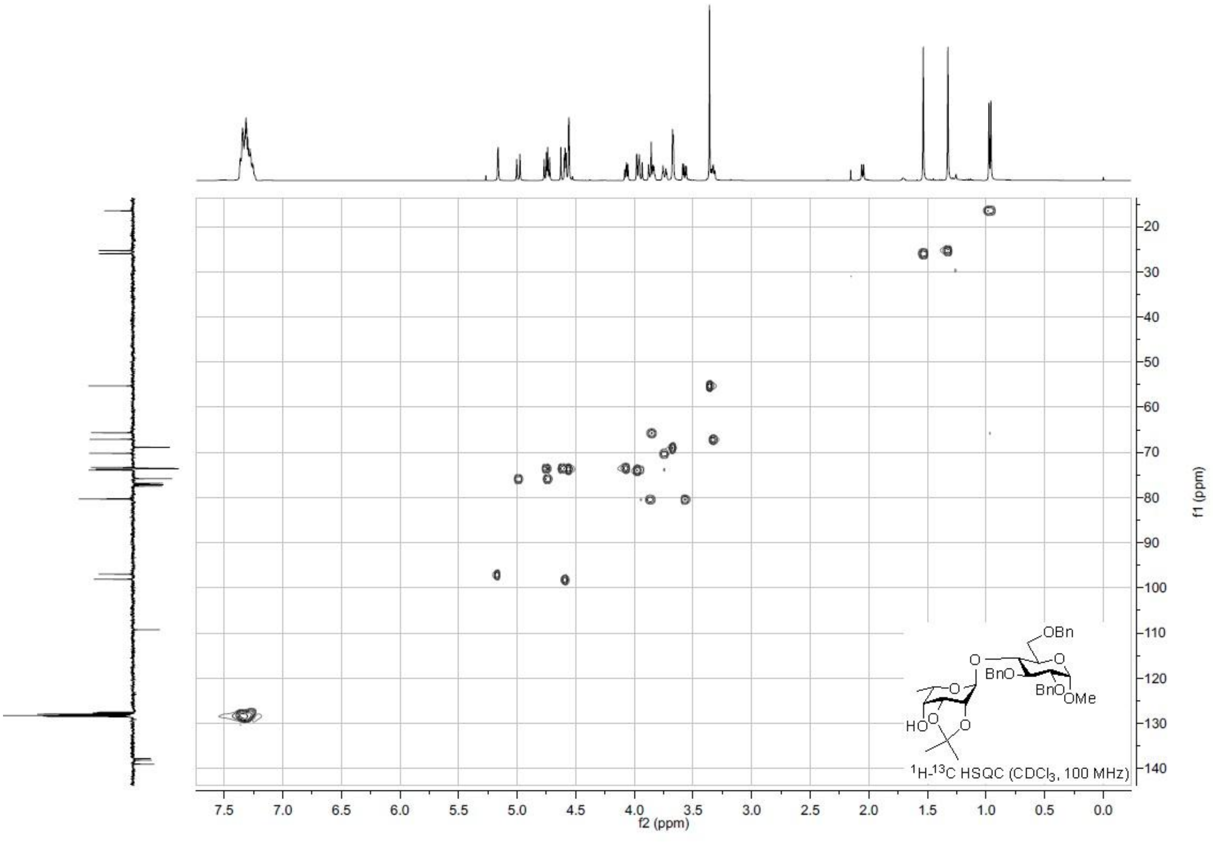
OBn
BnO
BnO
OMe
HO
1H-13C HSQC (CDCl3, 100 MHz)
f2 (ppm)
f1 (ppm)

**$^{1}$H and $^{13}$C NMR spectra of compound 19:**

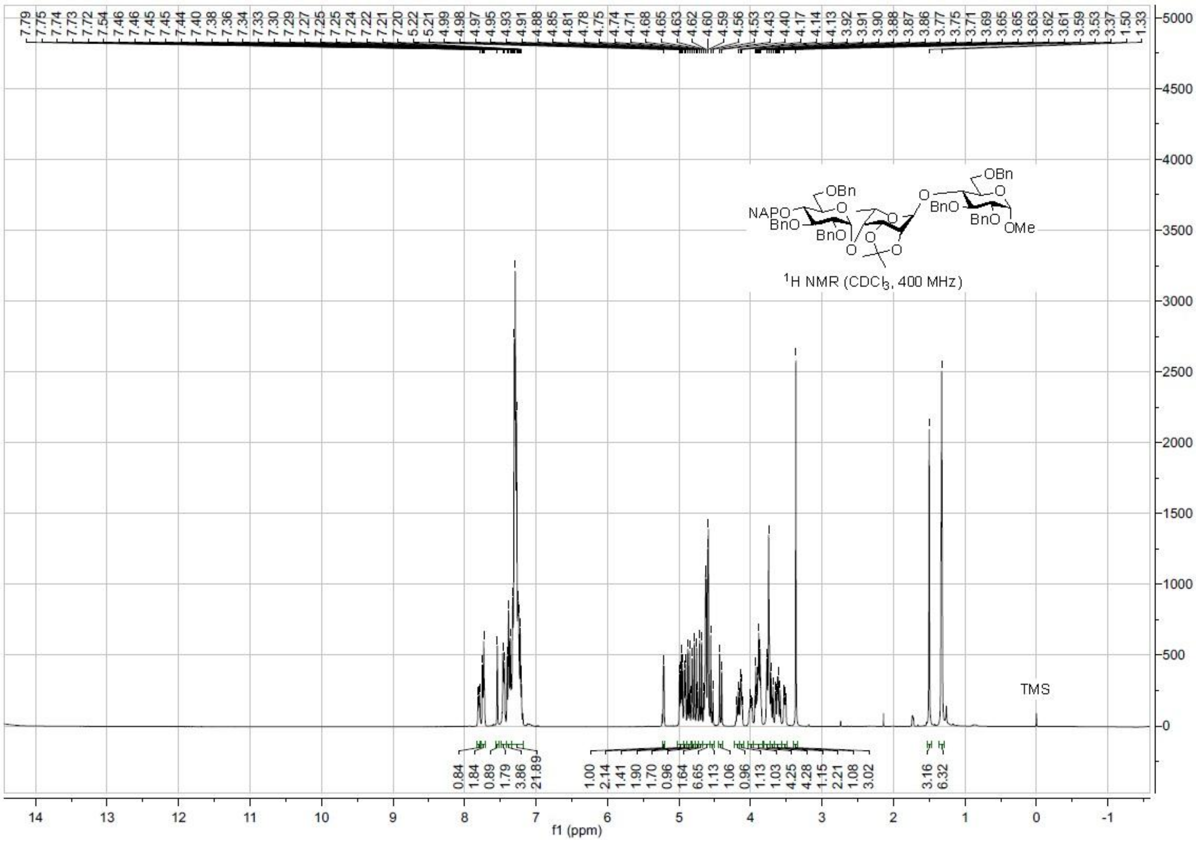


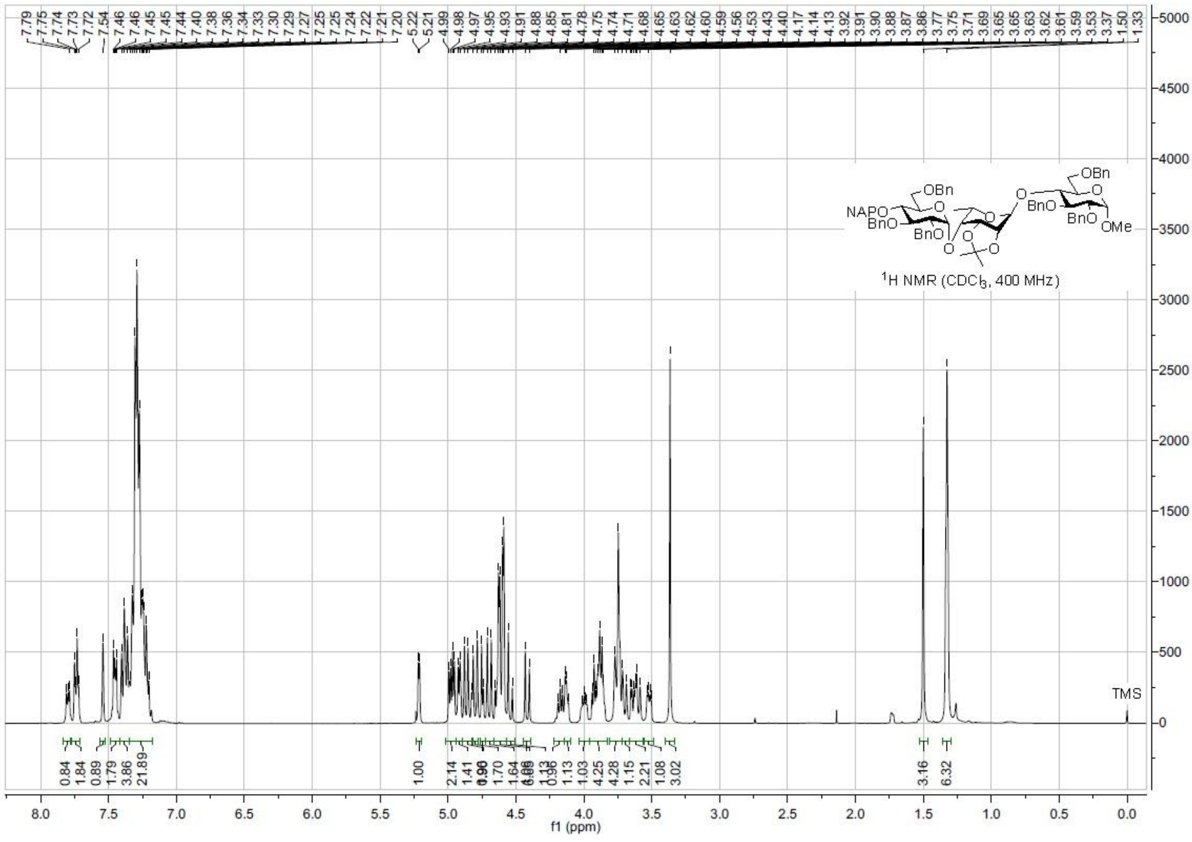

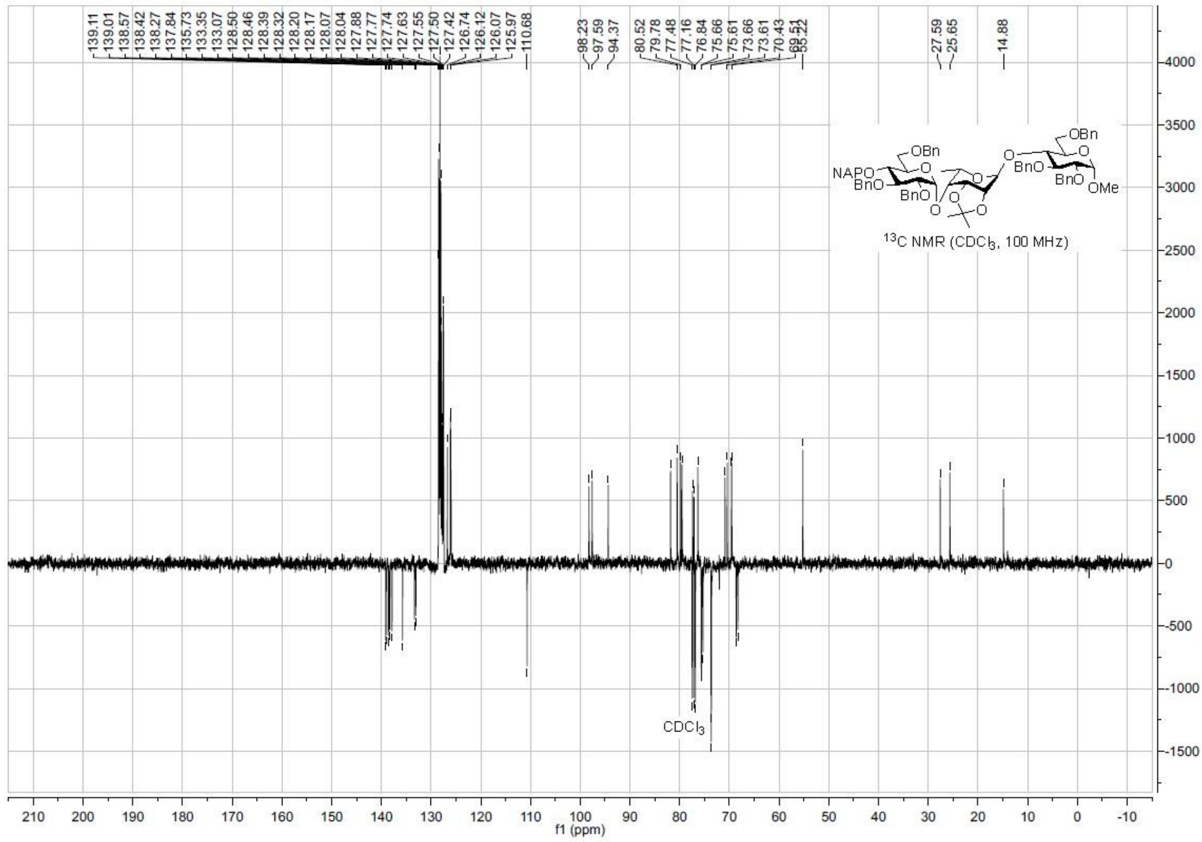

$^{13}$C NMR (CDCl$_3$, 100 MHz)
CDCl$_3$
f1 (ppm)


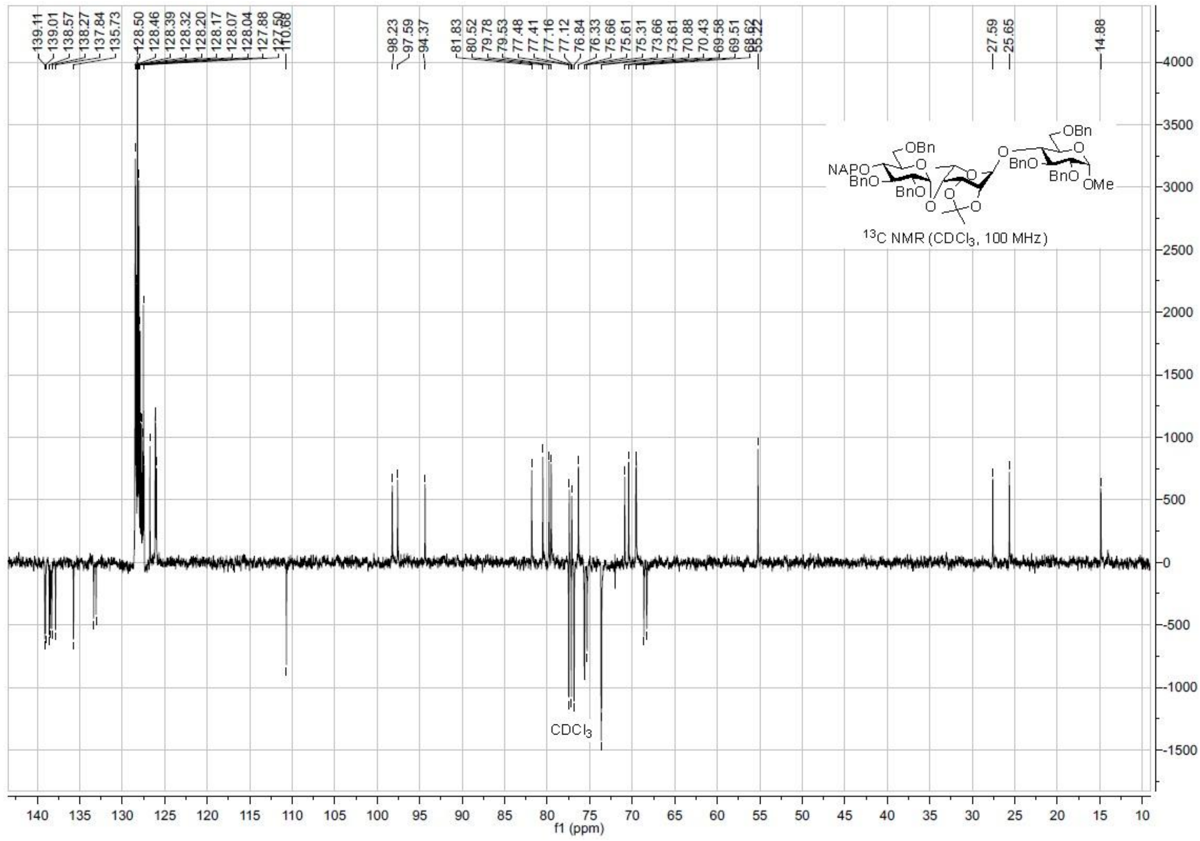

$^{13}$C NMR (CDCl$_3$, 100 MHz)
CDCl$_3$
f1 (ppm)

# $^{1}$H and $^{13}$C NMR spectra of compound 20:

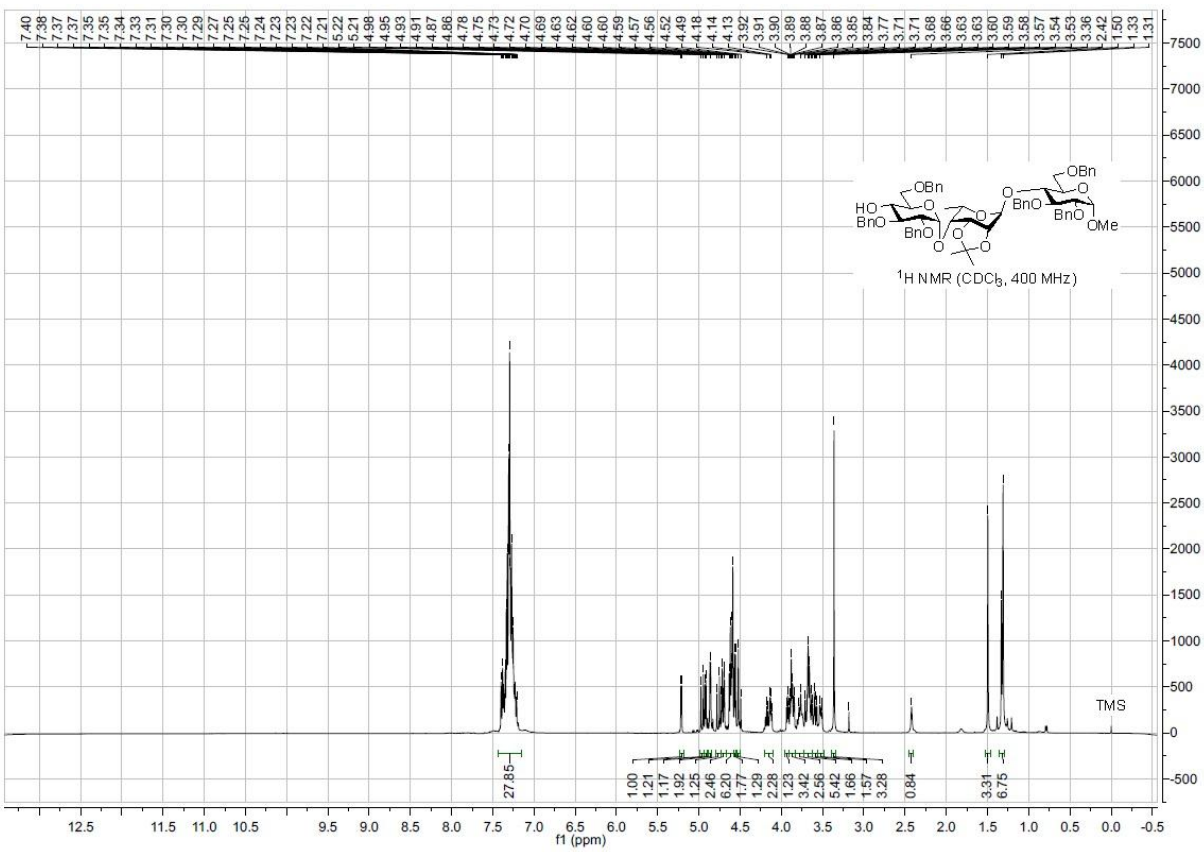

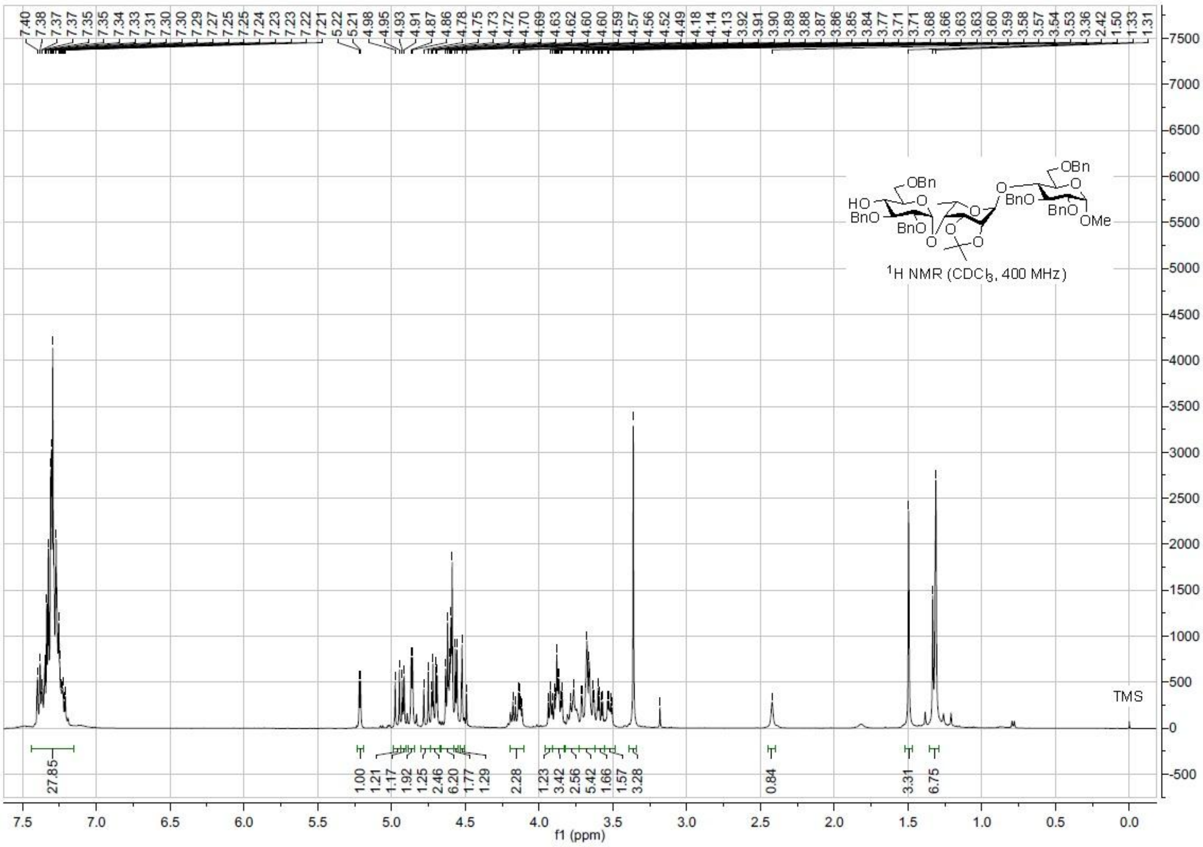

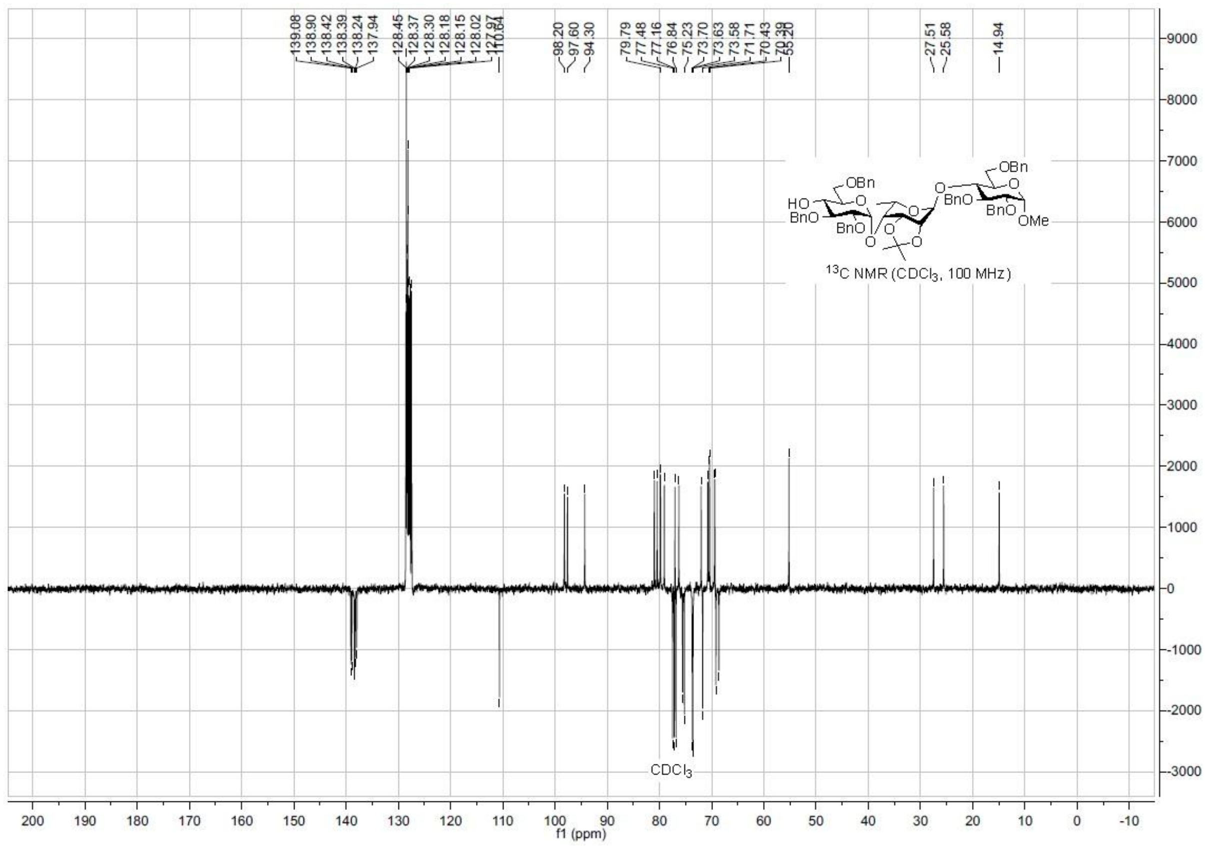

13C NMR (CDCl3, 100 MHz)
CDCl3
f1 (ppm)


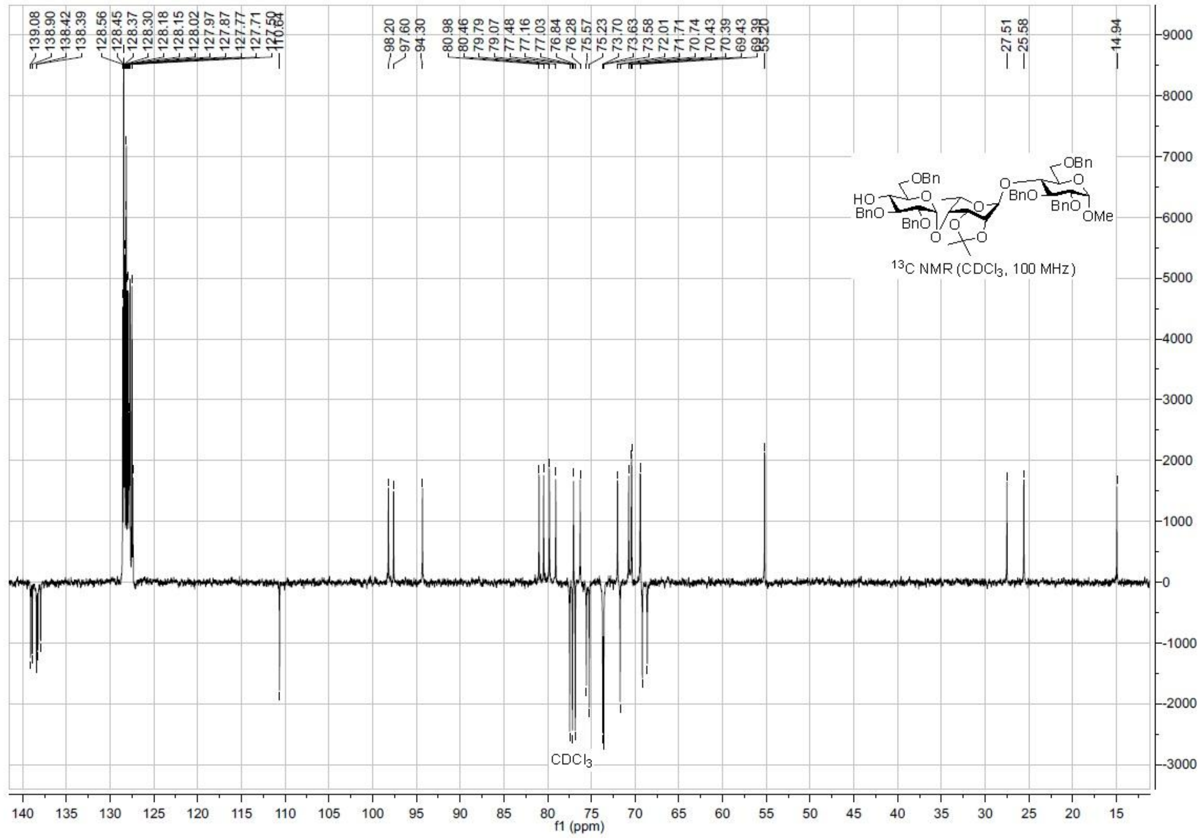

13C NMR (CDCl3, 100 MHz)
CDCl3
f1 (ppm)

**$^{1}$H and $^{13}$C NMR spectra of compound 21:**

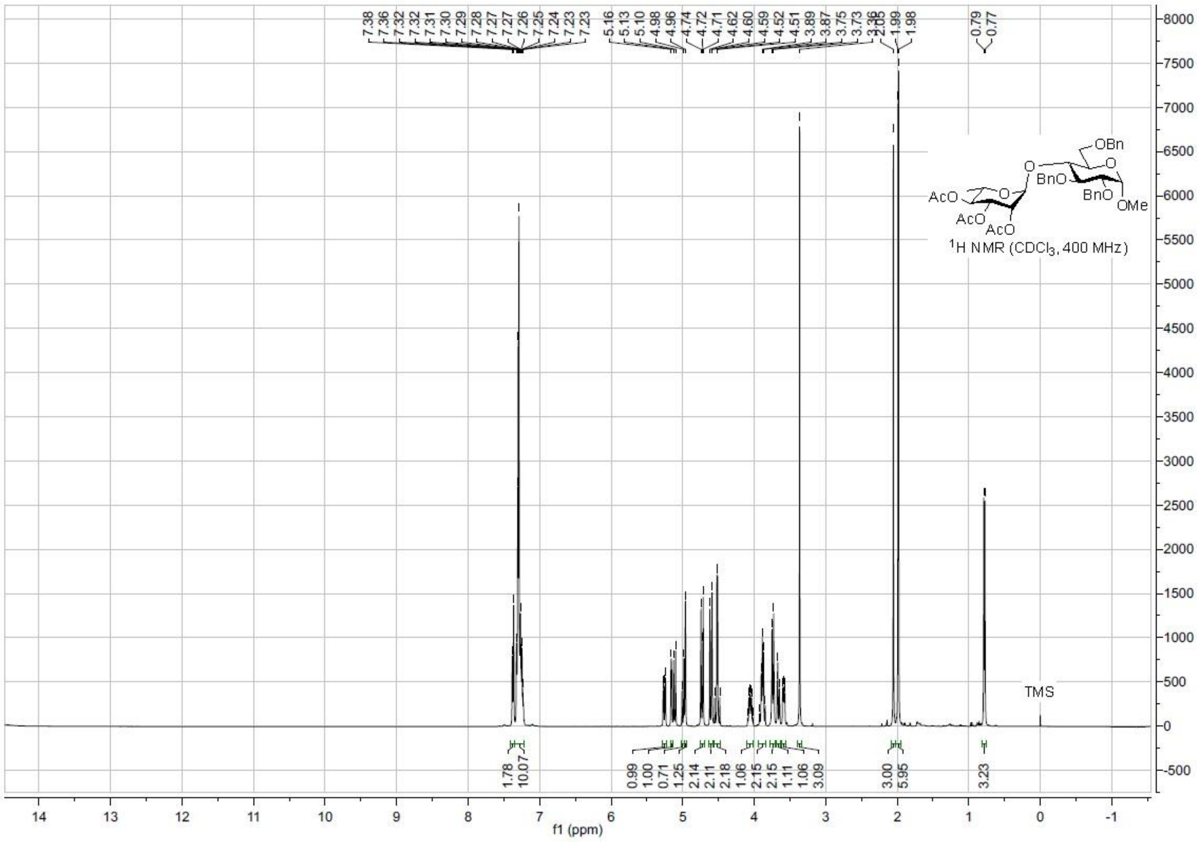


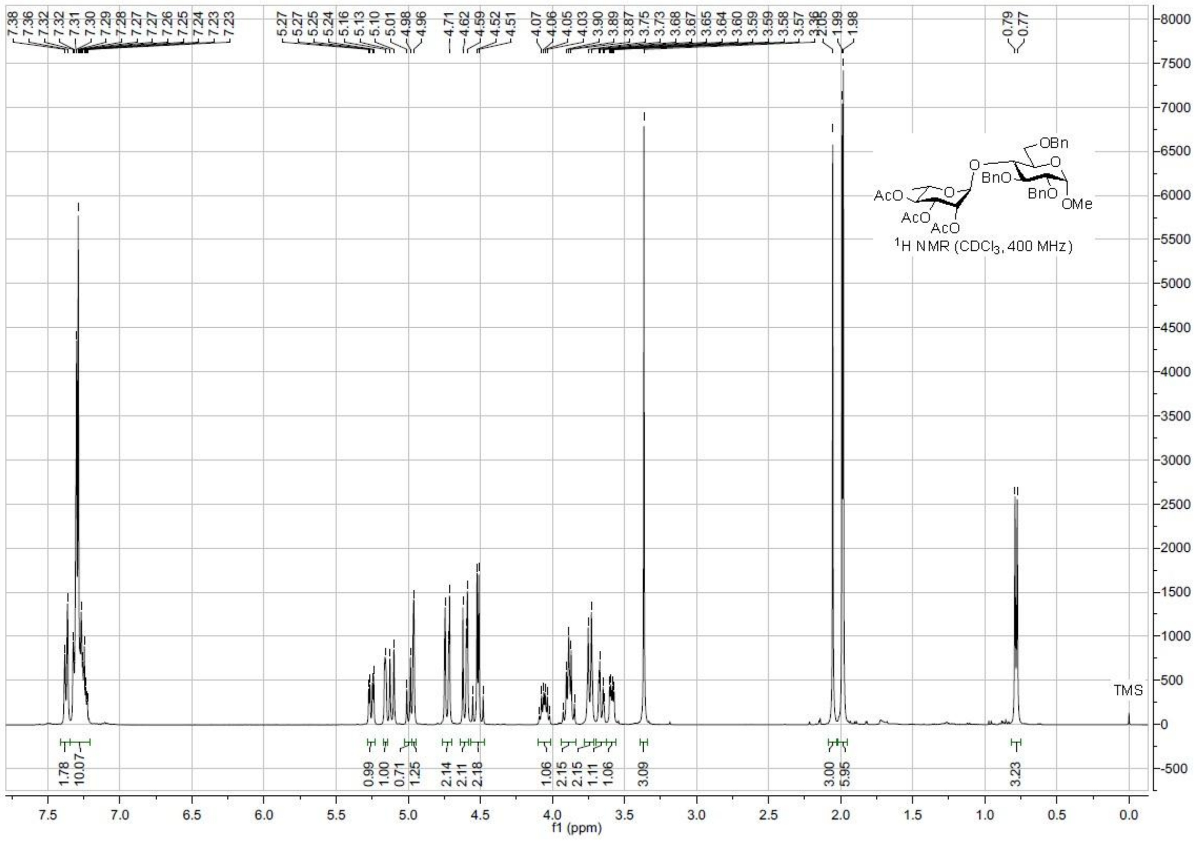

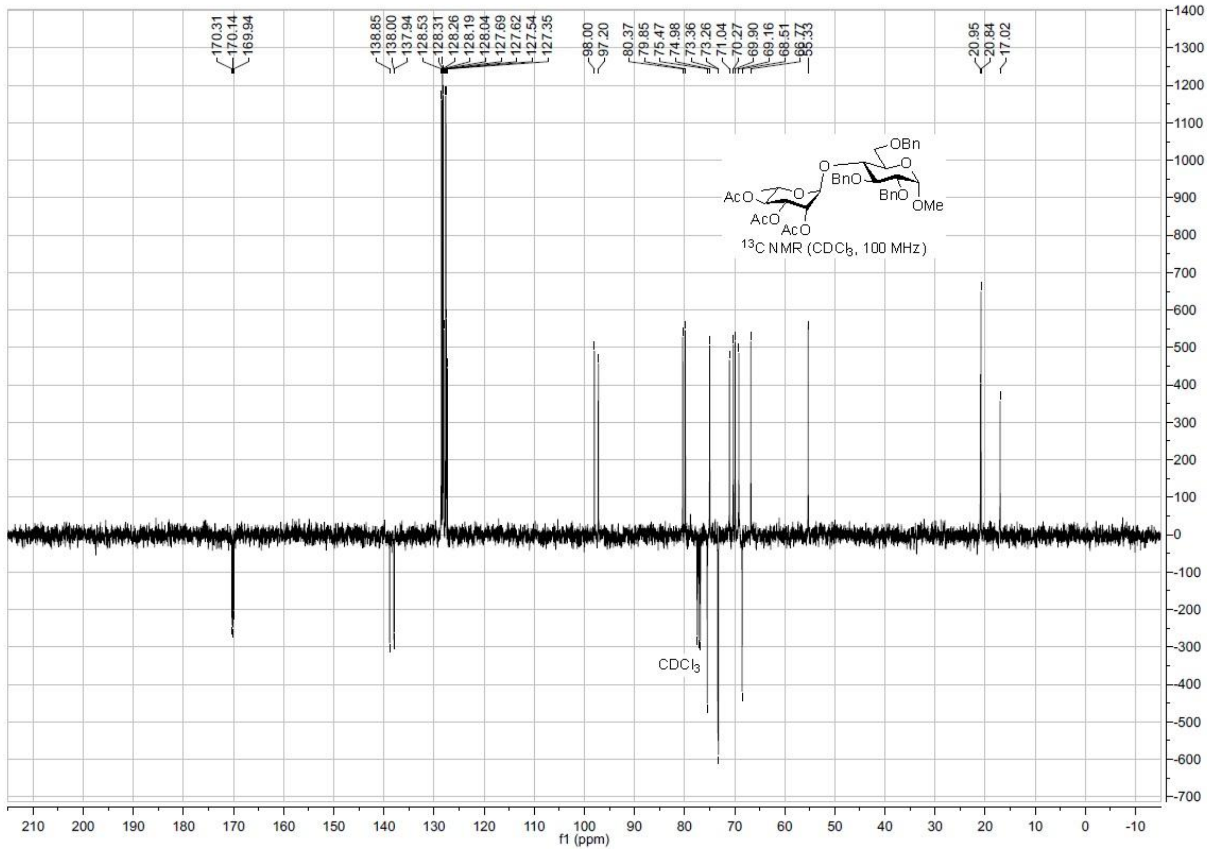
170.31
170.14
169.94
138.85
138.00
137.94
128.53
128.31
128.26
128.19
128.04
127.69
127.62
127.54
127.35
98.00
97.20
80.37
79.85
75.47
74.98
73.36
73.26
71.04
70.27
69.90
69.16
68.51
66.77
55.33
20.95
20.84
17.02
OBn
O
BnO
O
AcO
O
AcO
AcO
BnO
OMe
13C NMR (CDCl3, 100 MHz)
CDCl3
f1 (ppm)

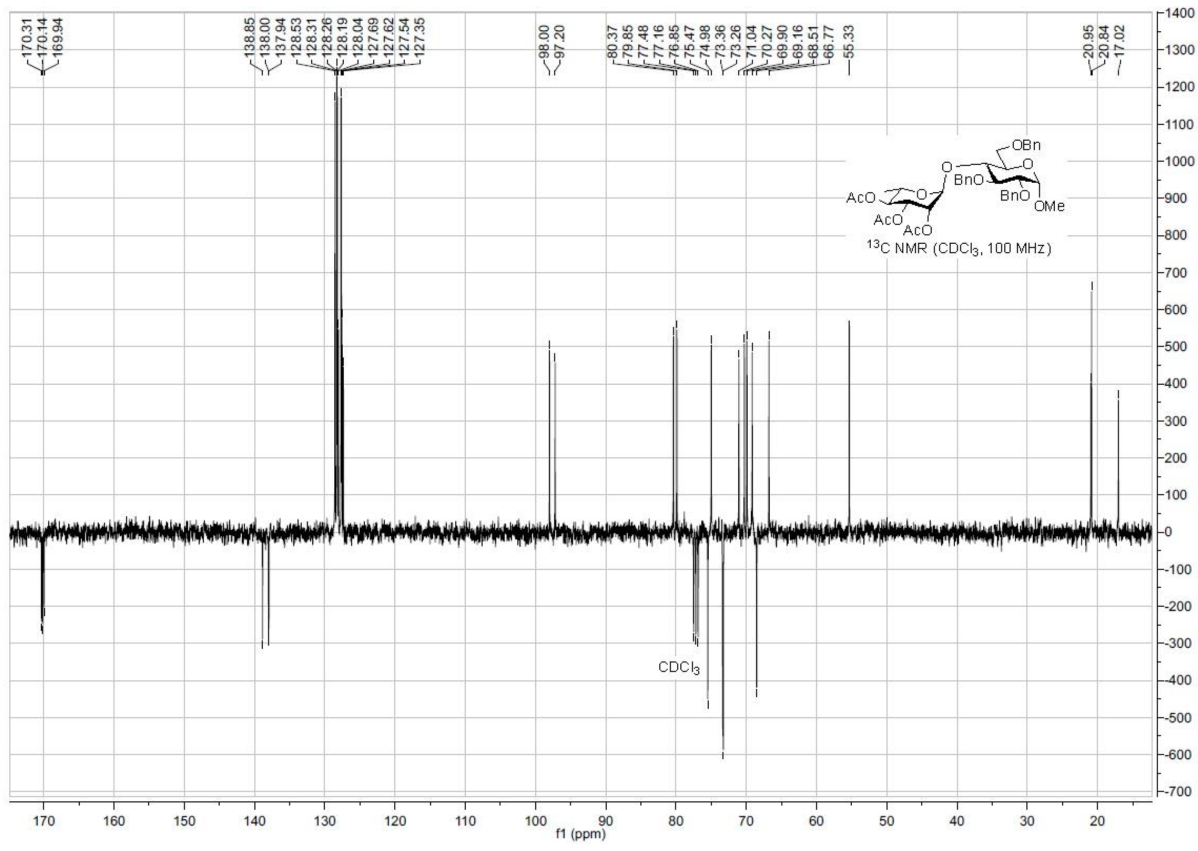
170.31
170.14
169.94
138.85
138.00
137.94
128.53
128.31
128.26
128.19
128.04
127.69
127.62
127.54
127.35
98.00
97.20
80.37
79.85
77.48
77.16
76.85
75.47
74.98
73.36
73.26
71.04
70.27
69.90
69.16
68.51
66.77
55.33
20.95
20.84
17.02
OBn
O
BnO
O
AcO
O
AcO
AcO
BnO
OMe
13C NMR (CDCl3, 100 MHz)
CDCl3
f1 (ppm)

## $^{1}$H and $^{13}$C NMR spectra of compound 22:

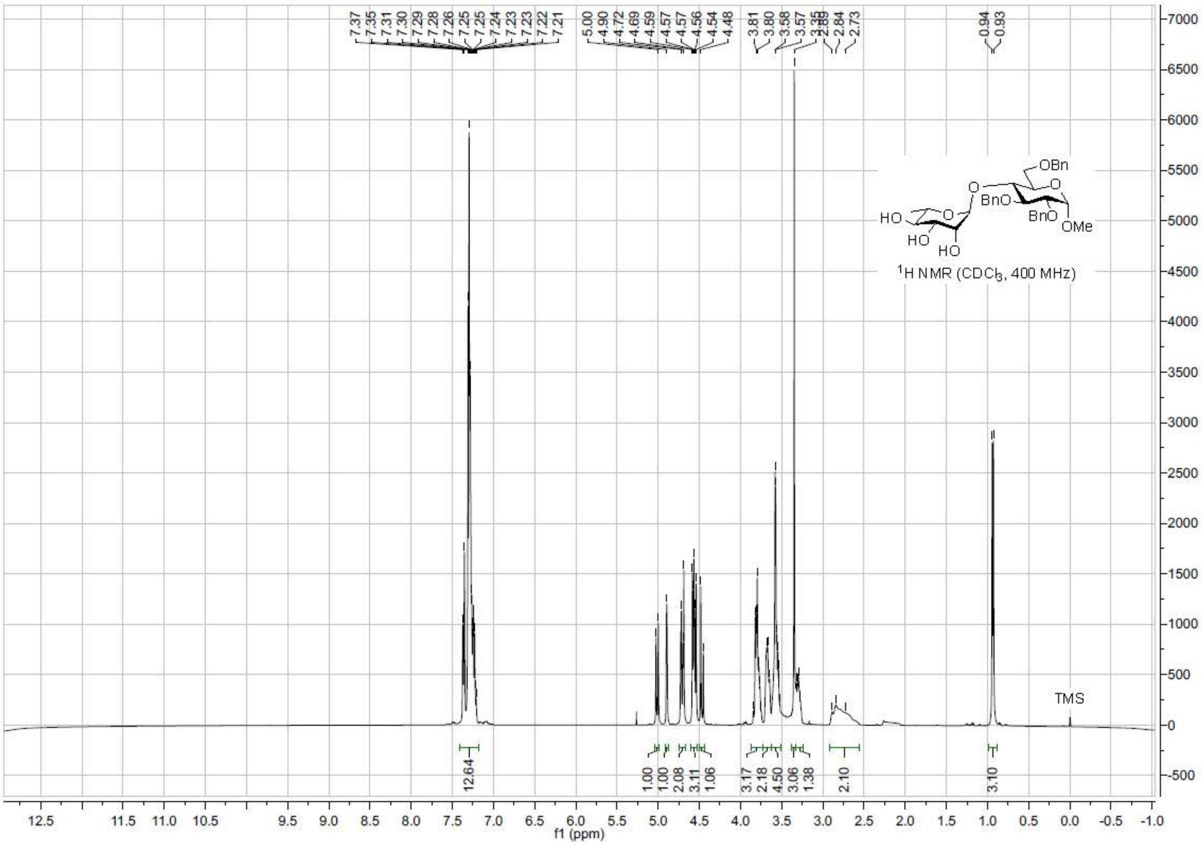


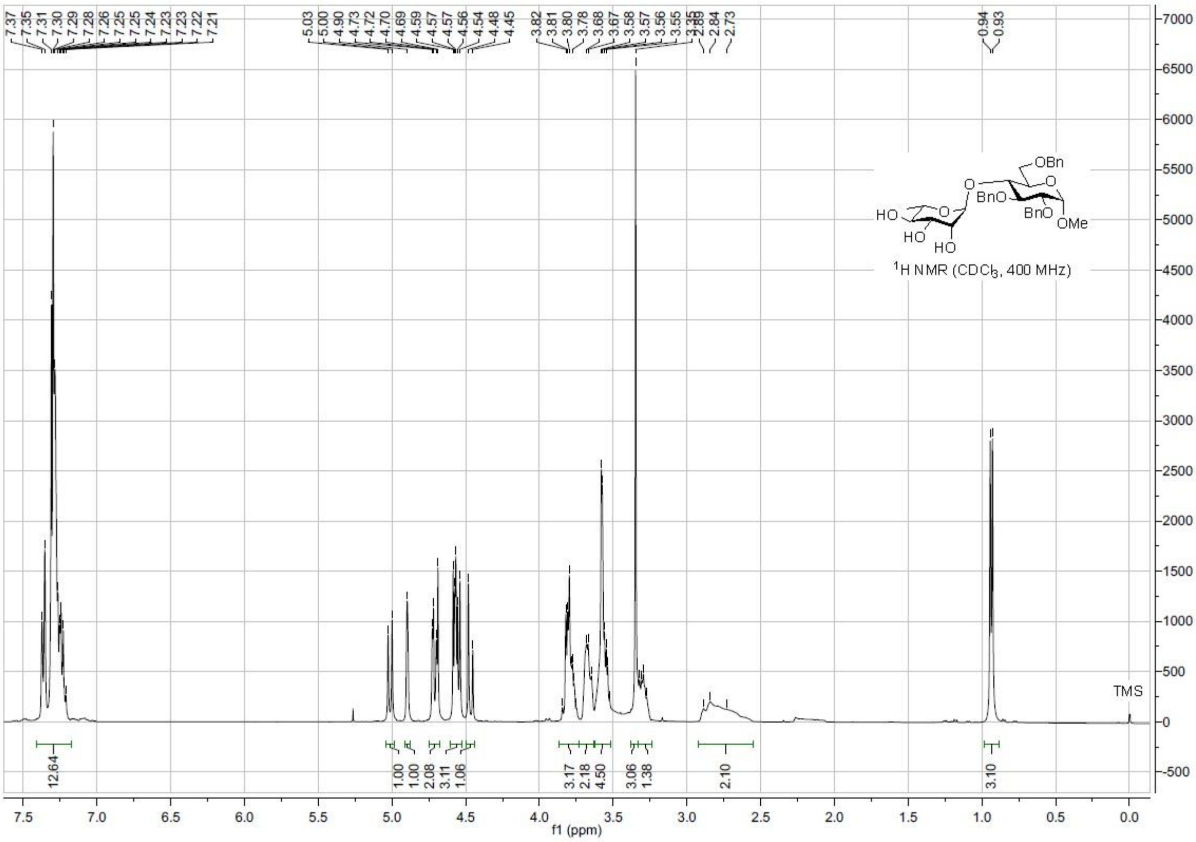

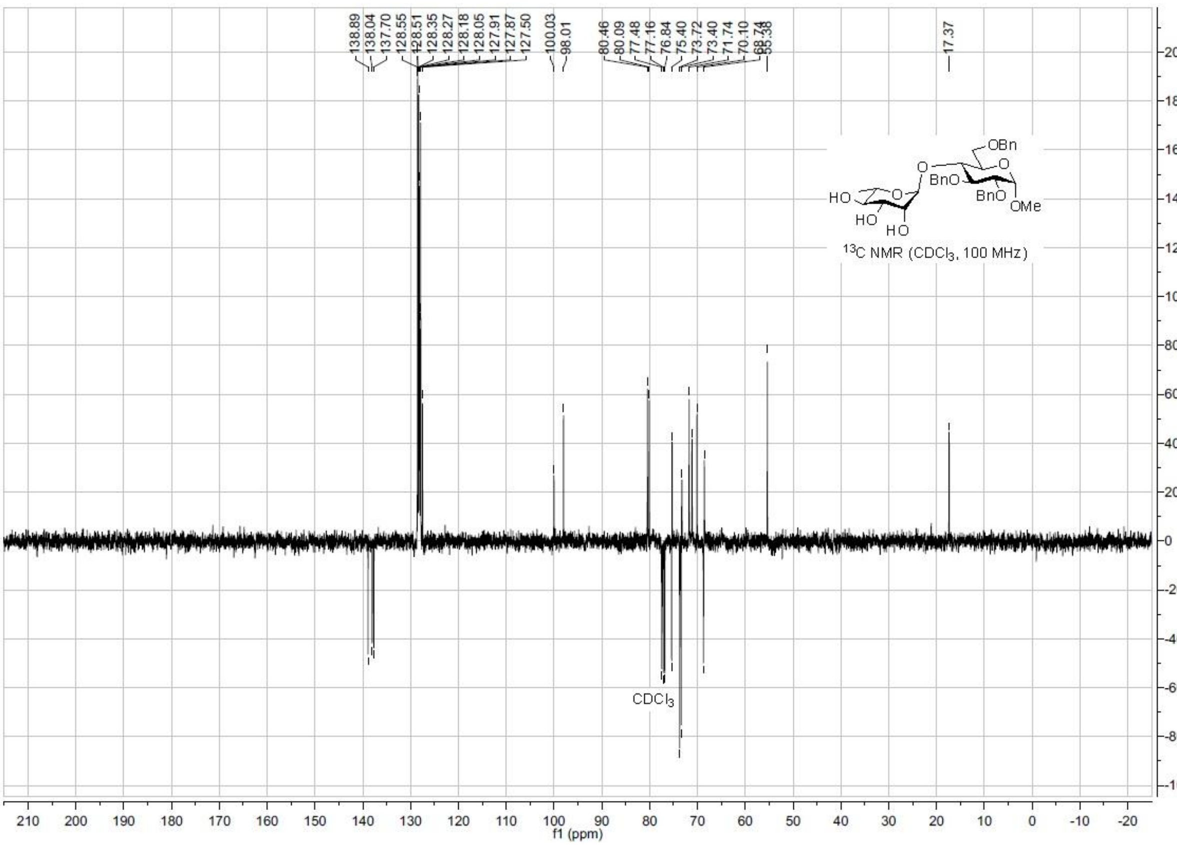
138.89
138.04
137.70
128.55
128.51
128.35
128.27
128.18
128.05
127.91
127.87
127.50
100.03
98.01
80.46
80.09
77.48
77.16
76.84
75.40
73.72
73.40
71.74
70.10
68.74
55.38
17.37
OBn
HO
HO
HO
BnO
BnO
OMe
$^{13}C$ NMR ($CDCl_3$, 100 MHz)
CDCl3
f1 (ppm)

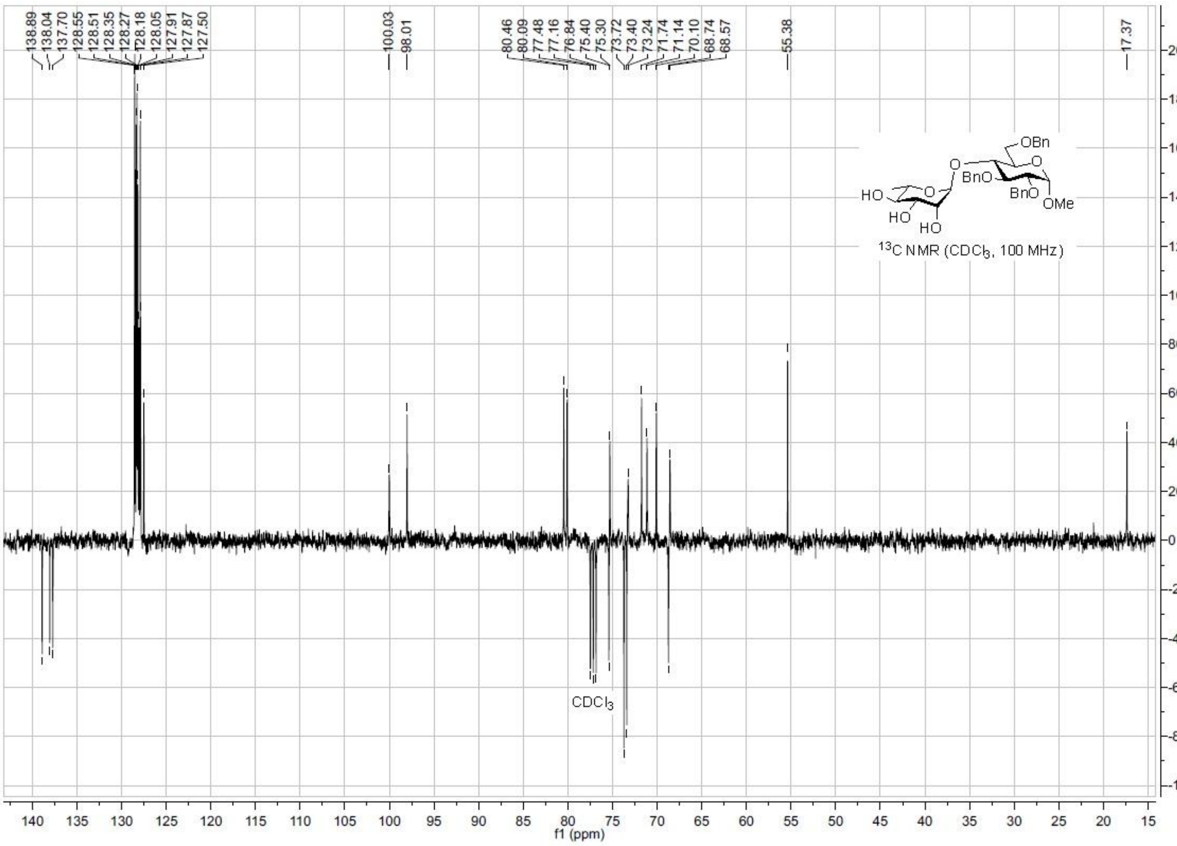
138.89
138.04
137.70
128.55
128.51
128.35
128.27
128.18
128.05
127.91
127.87
127.50
100.03
98.01
80.46
80.09
77.48
77.16
76.84
75.40
75.30
73.72
73.40
73.24
71.74
71.14
70.10
68.74
68.57
55.38
17.37
OBn
HO
HO
HO
BnO
BnO
OMe
$^{13}C$ NMR ($CDCl_3$, 100 MHz)
CDCl3
f1 (ppm)

## $^{1}$H and $^{13}$C NMR spectra of compound 23:

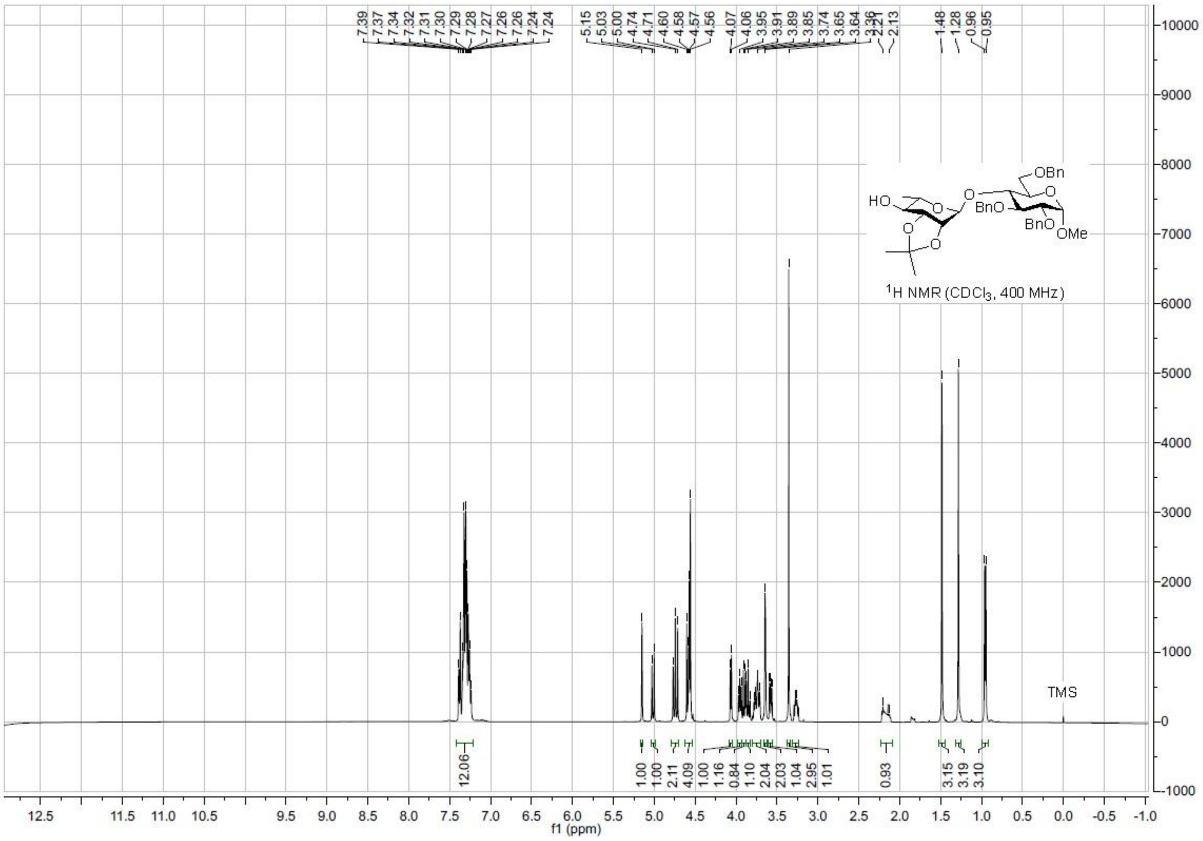


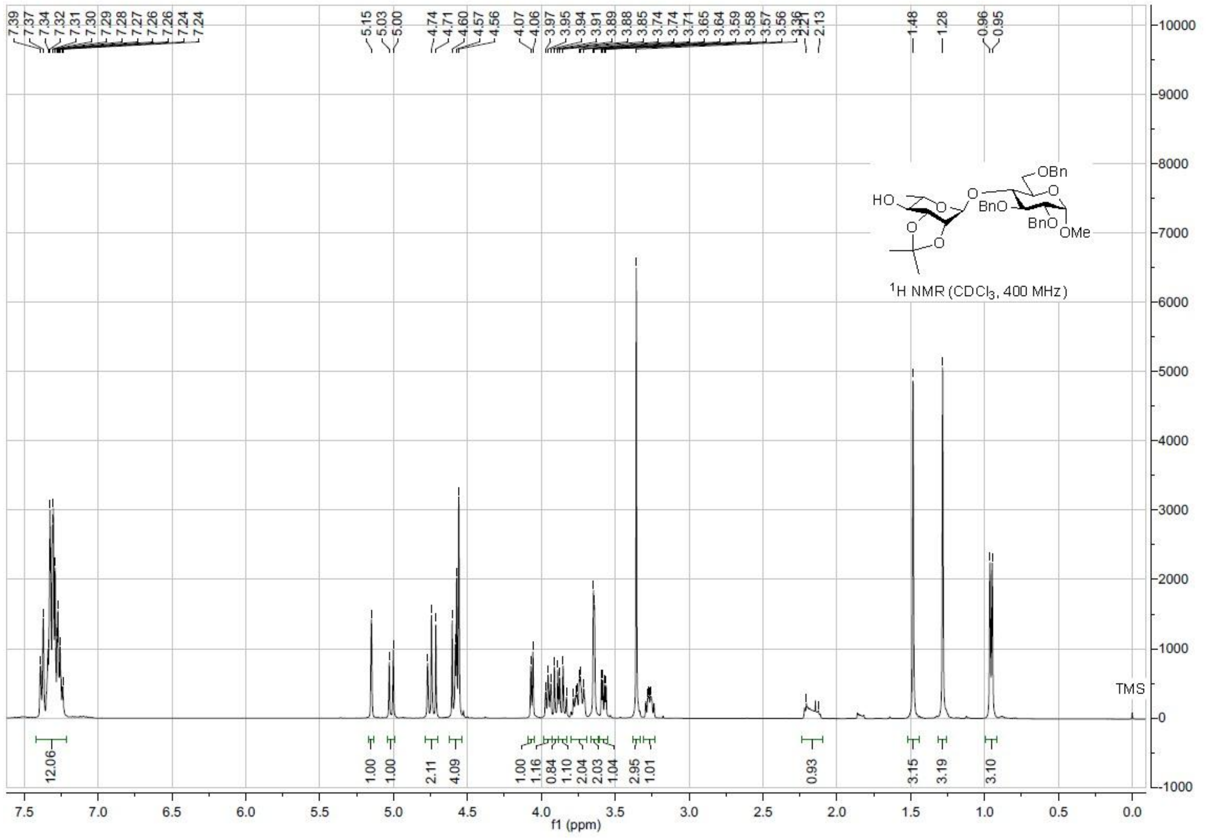

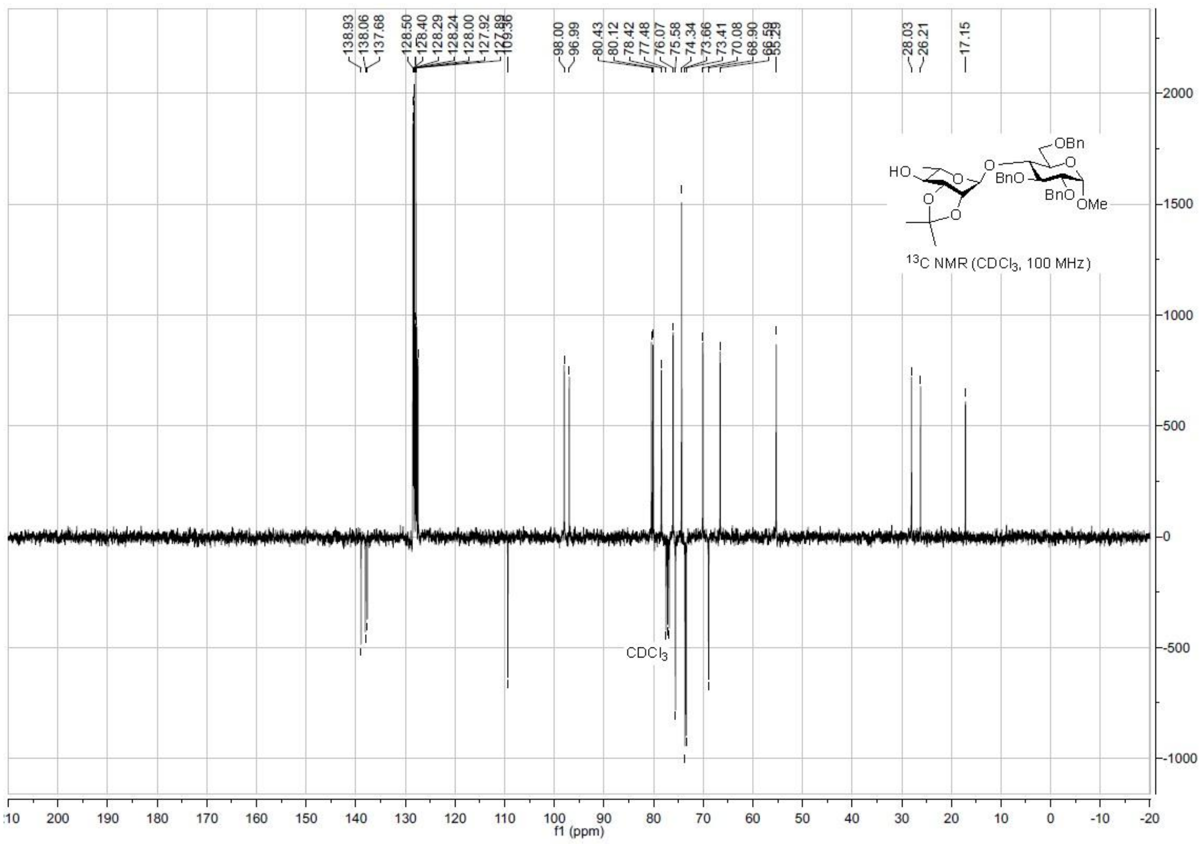

HO
O
O
O
O
OBn
O
BnO
BnO
OMe
13C NMR (CDCl3, 100 MHz)
CDCl3
f1 (ppm)


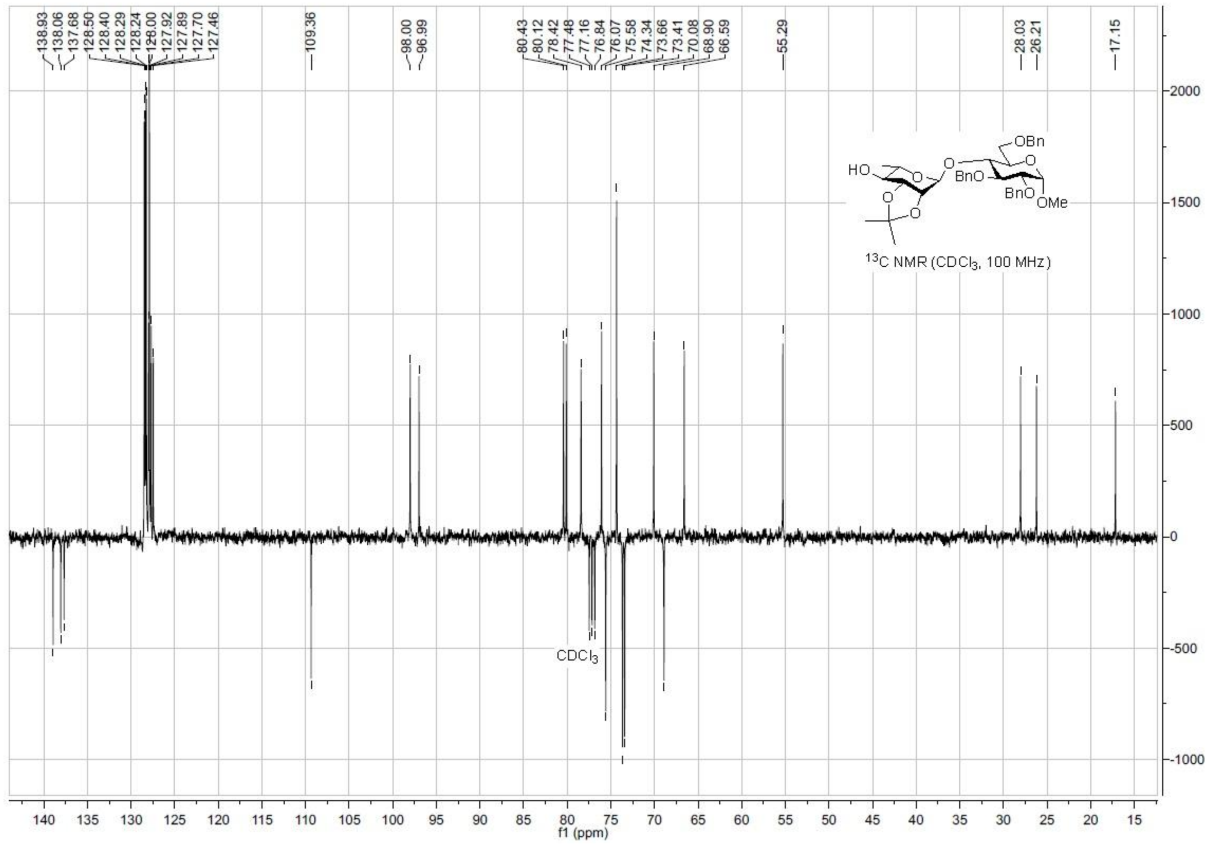

HO
O
O
O
O
OBn
O
BnO
BnO
OMe
13C NMR (CDCl3, 100 MHz)
CDCl3
f1 (ppm)

**$^{1}$H and $^{13}$C NMR spectra of 4'-ulose compound:**

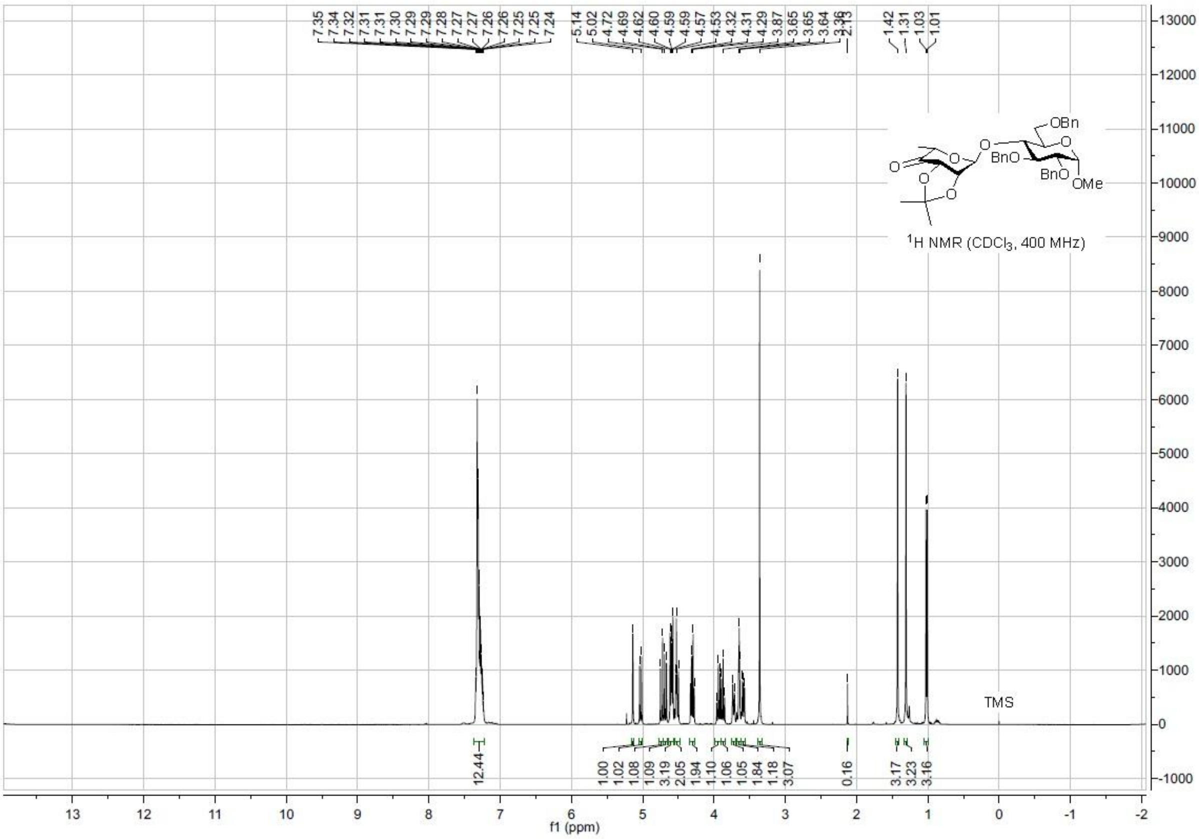

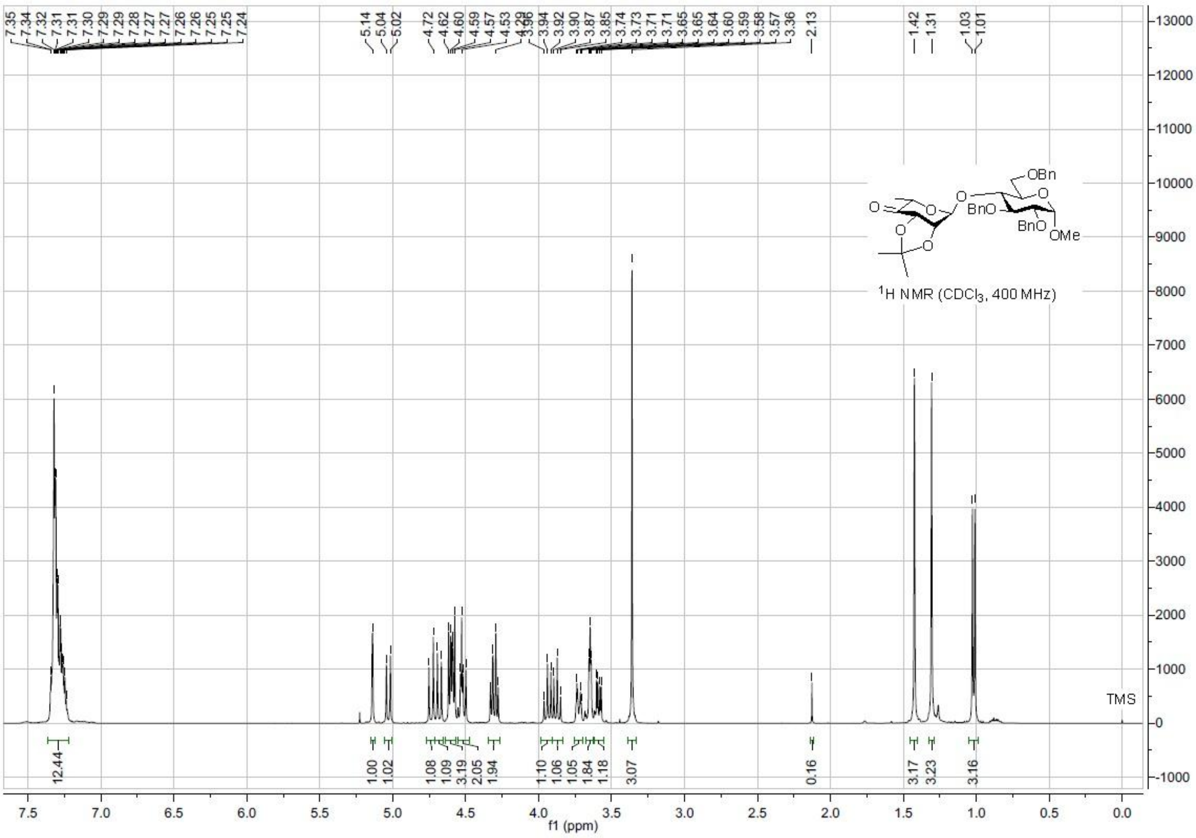

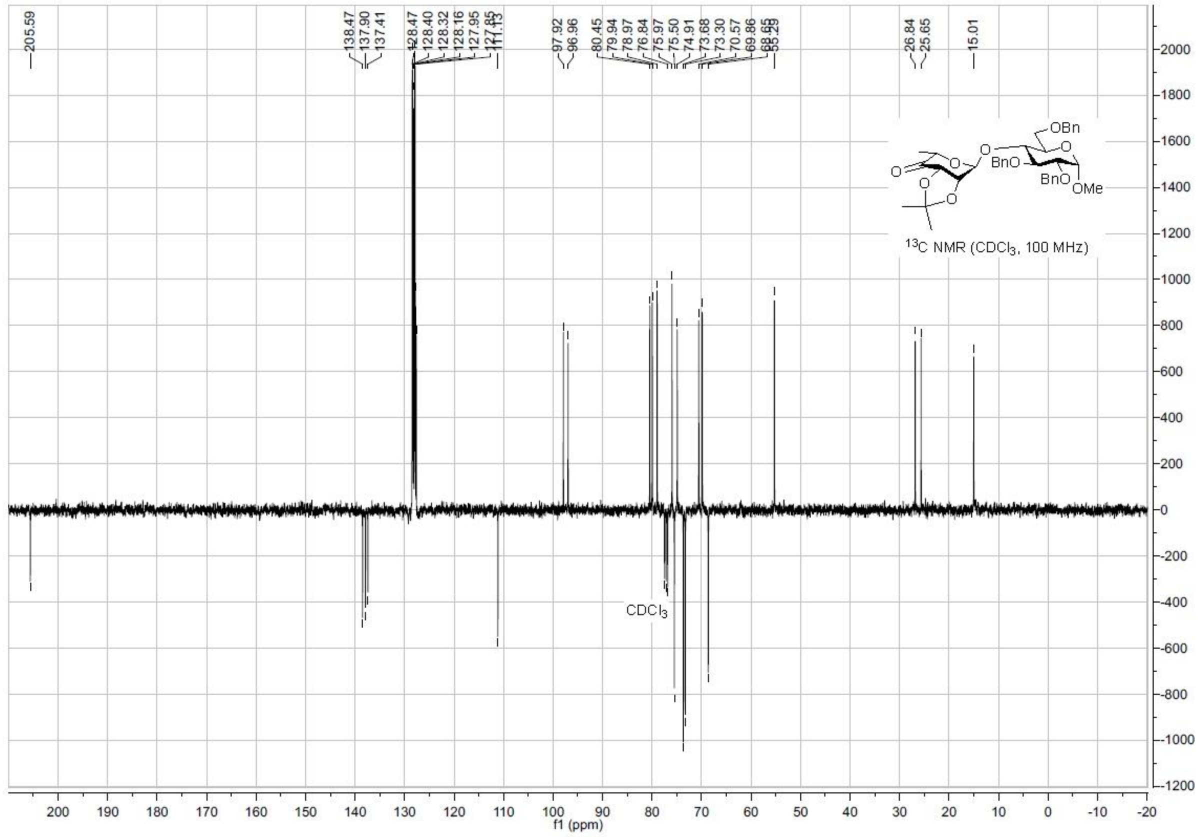

OBn
BnO
BnO
OMe
13C NMR (CDCl3, 100 MHz)
CDCl3
f1 (ppm)


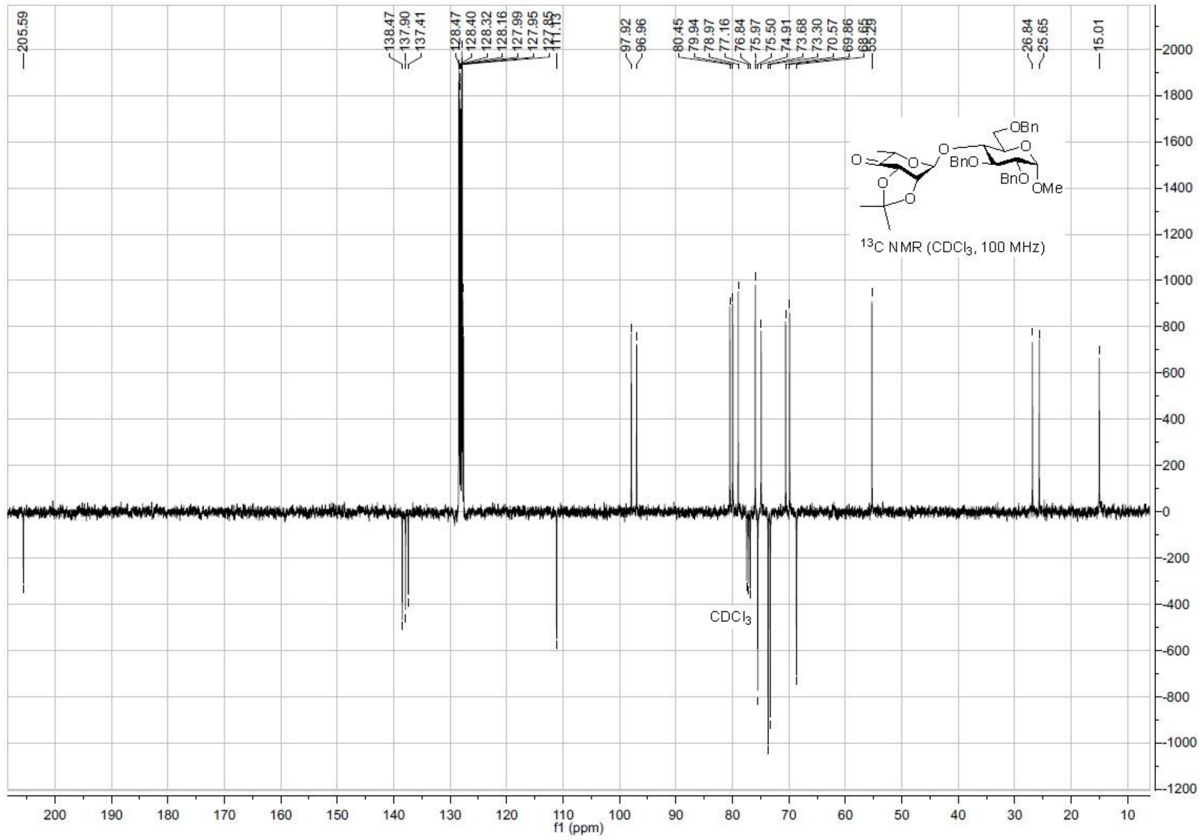

OBn
BnO
BnO
OMe
13C NMR (CDCl3, 100 MHz)
CDCl3
f1 (ppm)

**$^{1}$H and $^{13}$C NMR spectra of compound 24:**

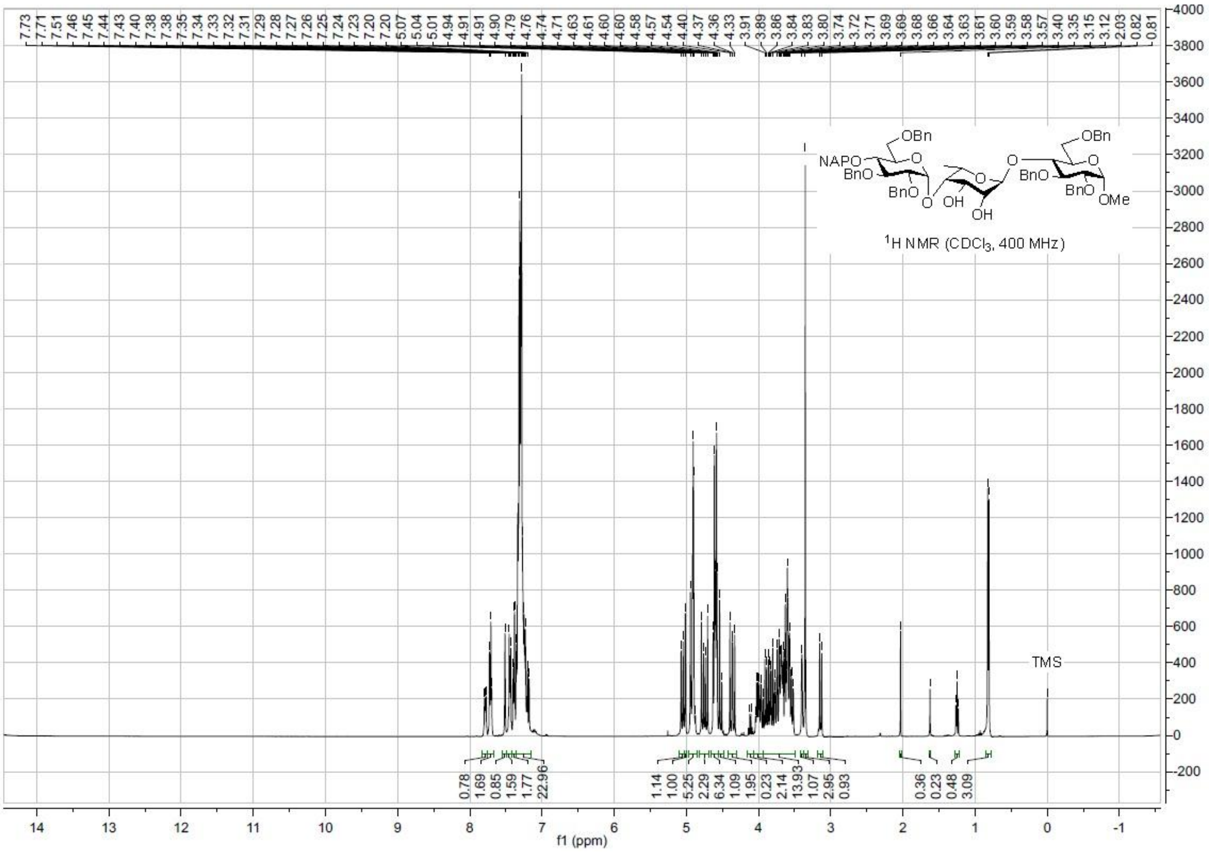


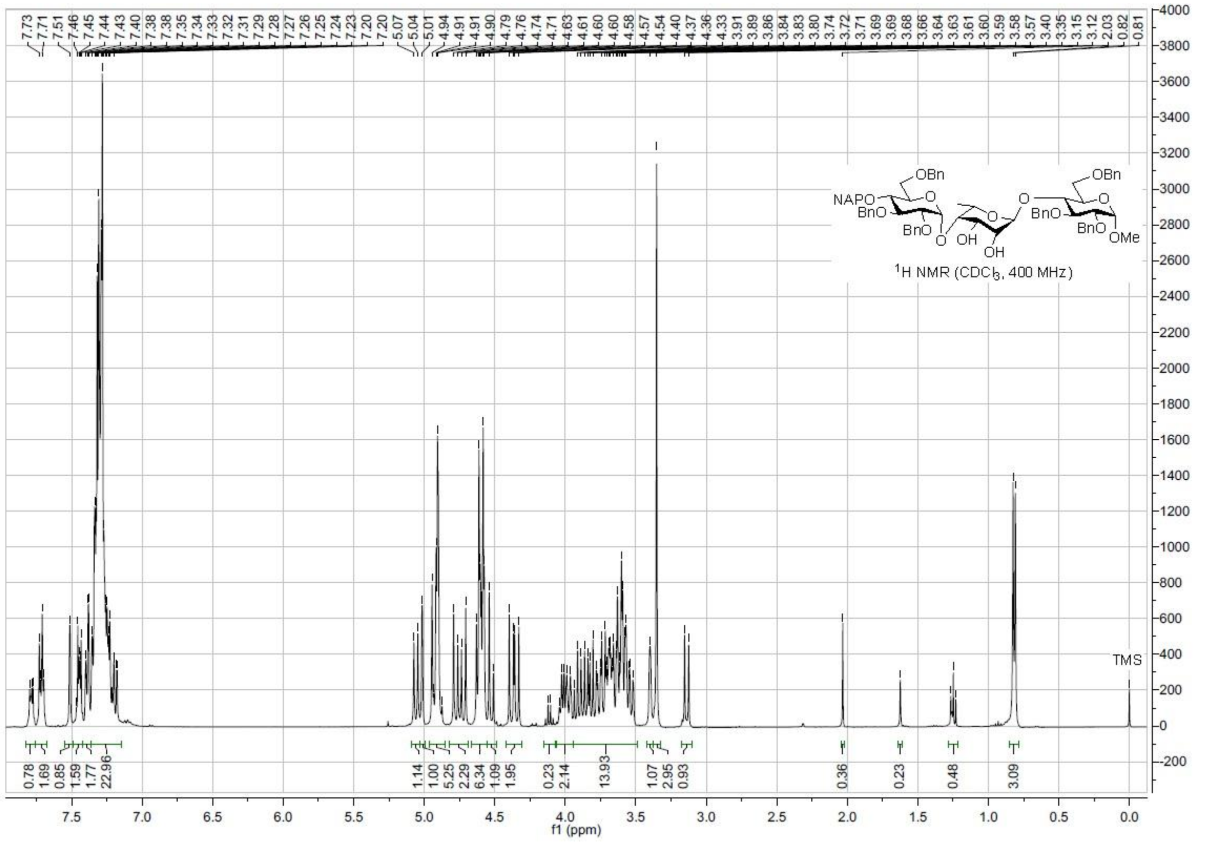

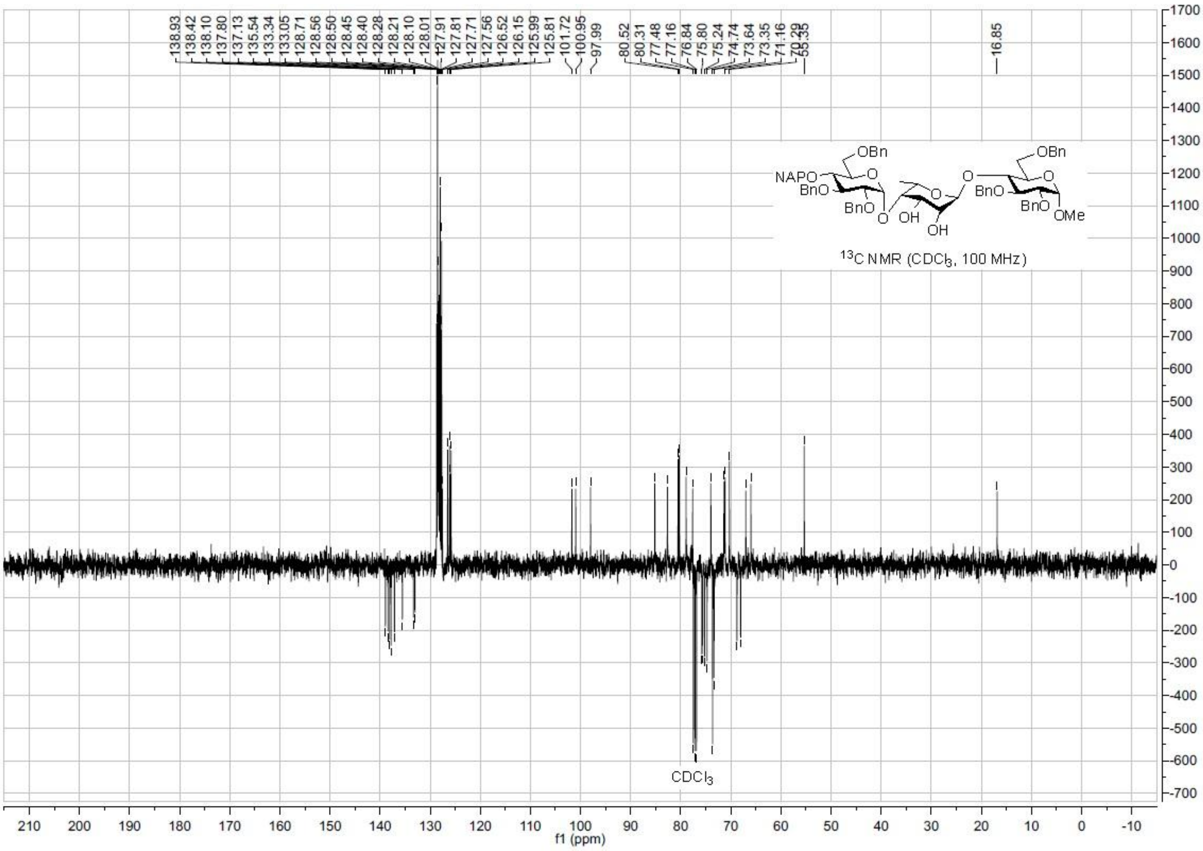
13C NMR (CDCl3, 100 MHz)
CDCl3
f1 (ppm)

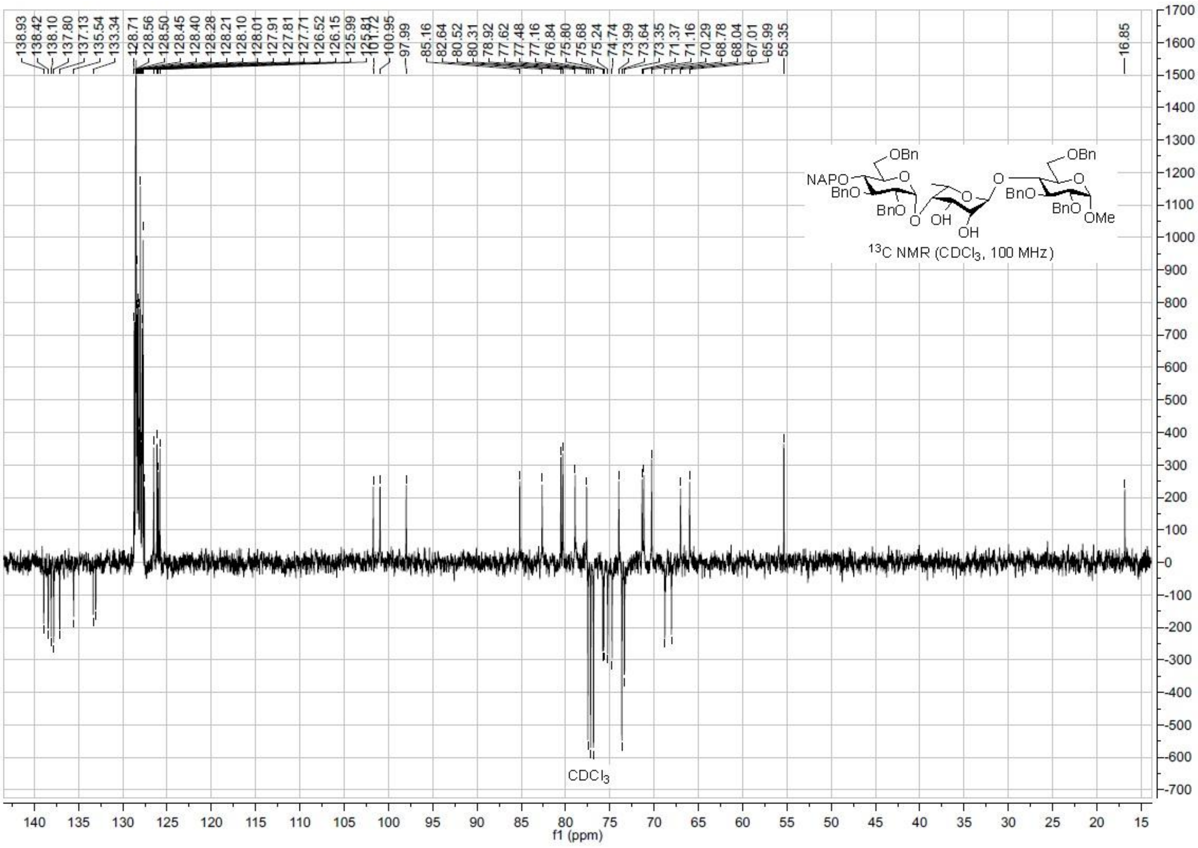
13C NMR (CDCl3, 100 MHz)
CDCl3
f1 (ppm)

# $^{1}H$ and $^{13}C$ NMR spectra of compound 26:

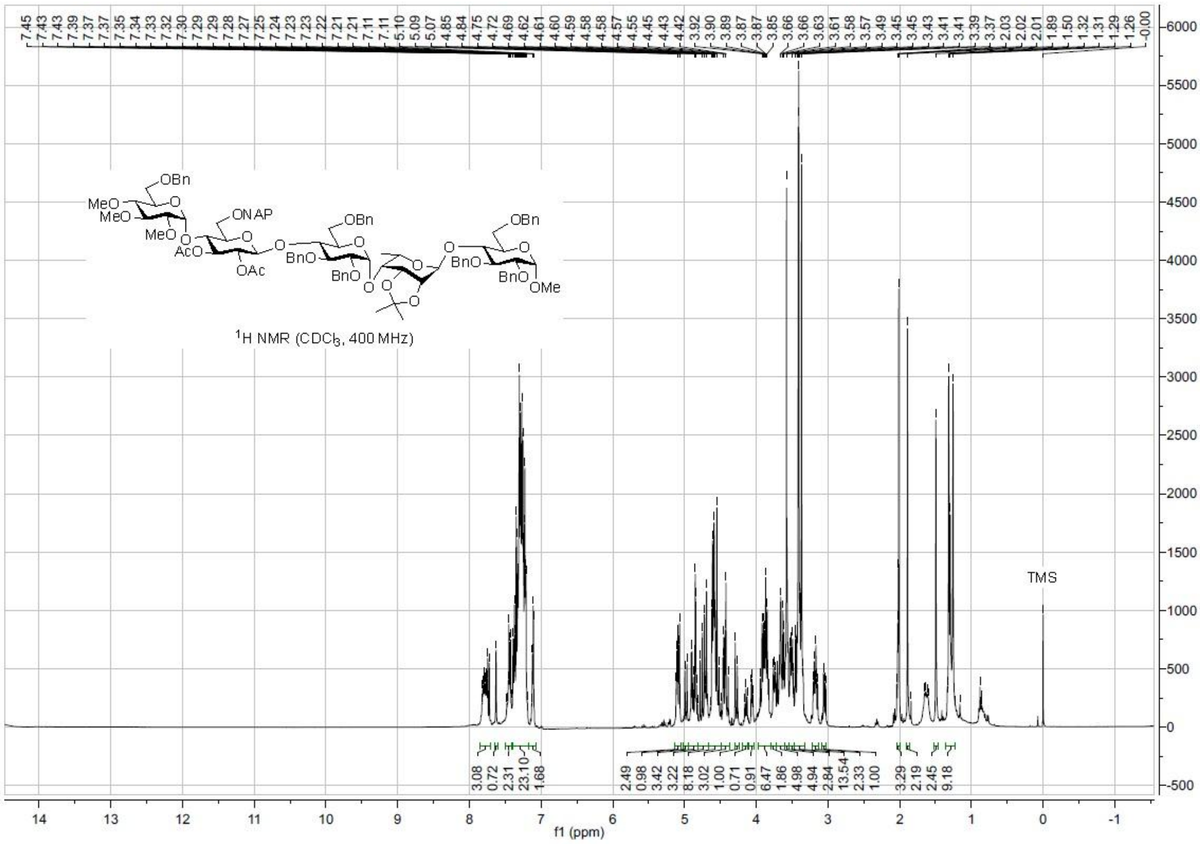


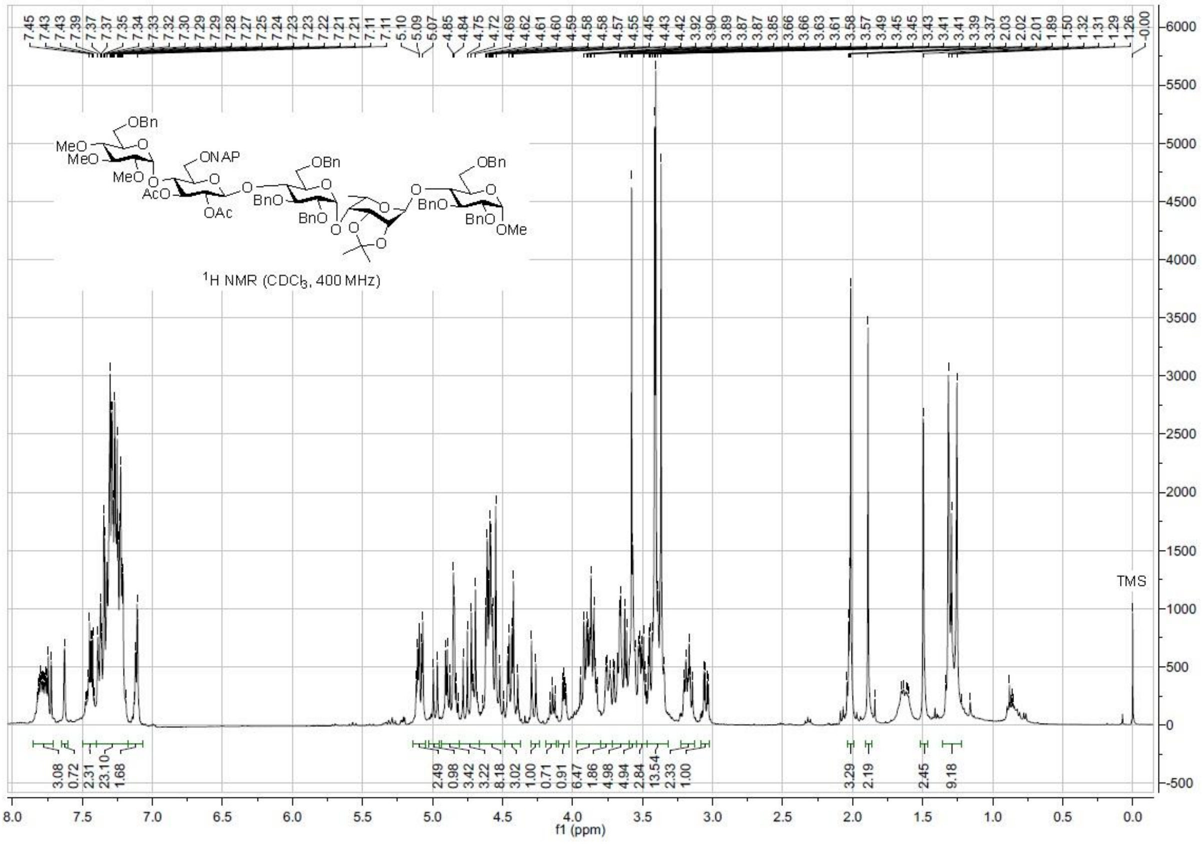

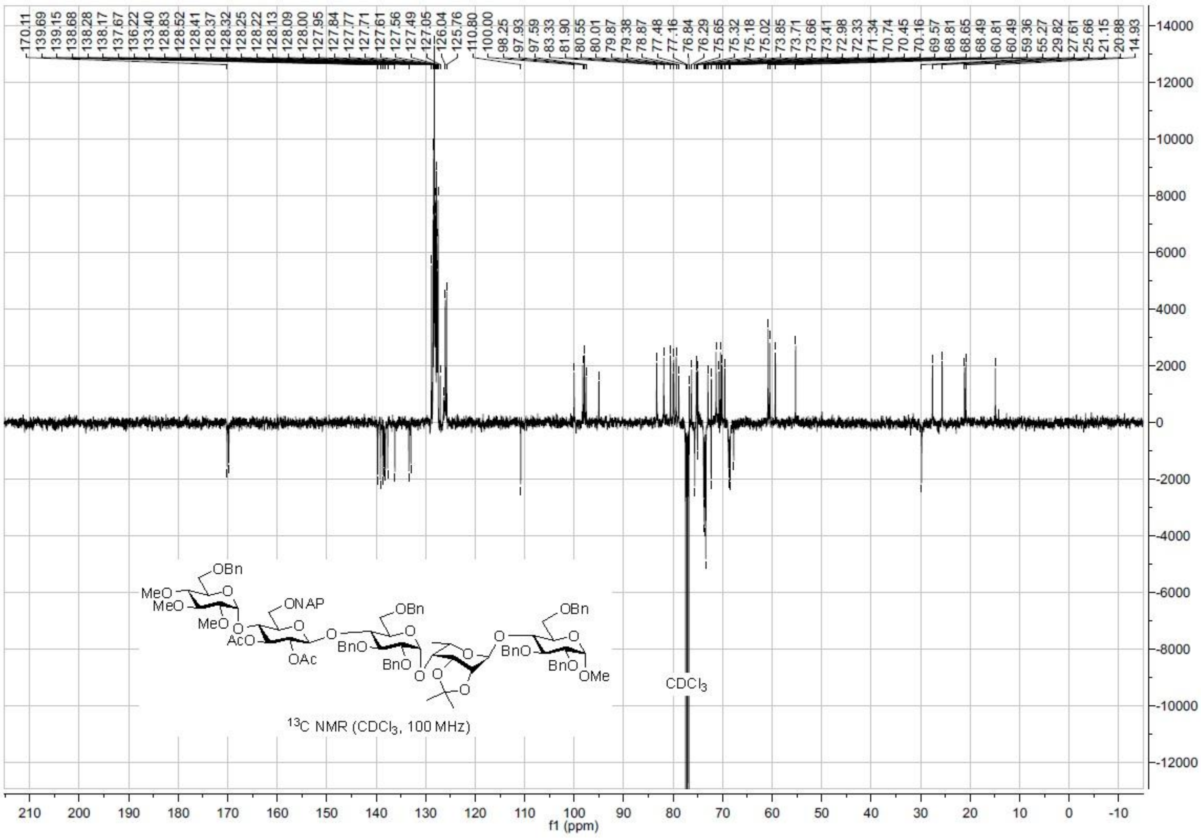
170.11 139.69 139.15 138.68 138.28 138.17 137.67 136.22 133.40 128.83 128.52 128.41 128.37 128.32 128.25 128.22 128.13 128.09 128.00 127.95 127.84 127.77 127.71 127.61 127.56 127.49 127.05 126.04 125.76 110.80 100.00 98.25 97.93 97.59 83.33 81.90 80.55 80.01 79.87 79.38 78.87 77.48 77.16 76.84 76.29 75.65 75.32 75.18 75.02 73.85 73.71 73.66 73.41 72.98 72.33 71.34 70.74 70.45 70.16 69.57 68.81 68.65 68.49 60.81 60.49 59.36 55.27 29.82 27.61 25.66 21.15 20.88 14.93
MeO
MeO
MeO
OBn
ONAP
AcO
OAc
OBn
BnO
BnO
O
OBn
BnO
BnO
OMe
CDCl3
13C NMR (CDCl3, 100 MHz)
f1 (ppm)

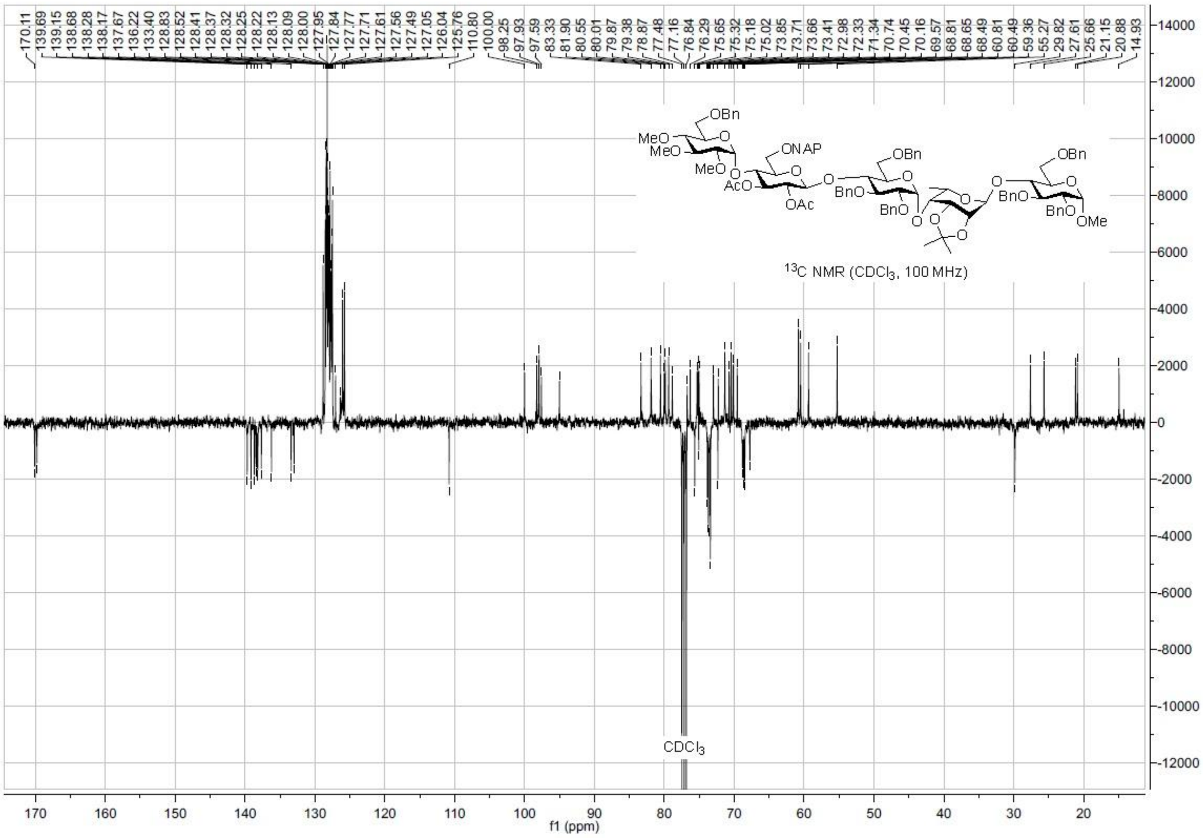
170.11 139.69 139.15 138.68 138.28 138.17 137.67 136.22 133.40 128.83 128.52 128.41 128.37 128.32 128.25 128.22 128.13 128.09 128.00 127.95 127.84 127.77 127.71 127.61 127.56 127.49 127.05 126.04 125.76 110.80 100.00 98.25 97.93 97.59 83.33 81.90 80.55 80.01 79.87 79.38 78.87 77.48 77.16 76.84 76.29 75.65 75.32 75.18 75.02 73.85 73.71 73.66 73.41 72.98 72.33 71.34 70.74 70.45 70.16 69.57 68.81 68.65 68.49 60.81 60.49 59.36 55.27 29.82 27.61 25.66 21.15 20.88 14.93
MeO
MeO
MeO
OBn
ONAP
AcO
OAc
OBn
BnO
BnO
O
OBn
BnO
BnO
OMe
13C NMR (CDCl3, 100 MHz)
CDCl3
f1 (ppm)

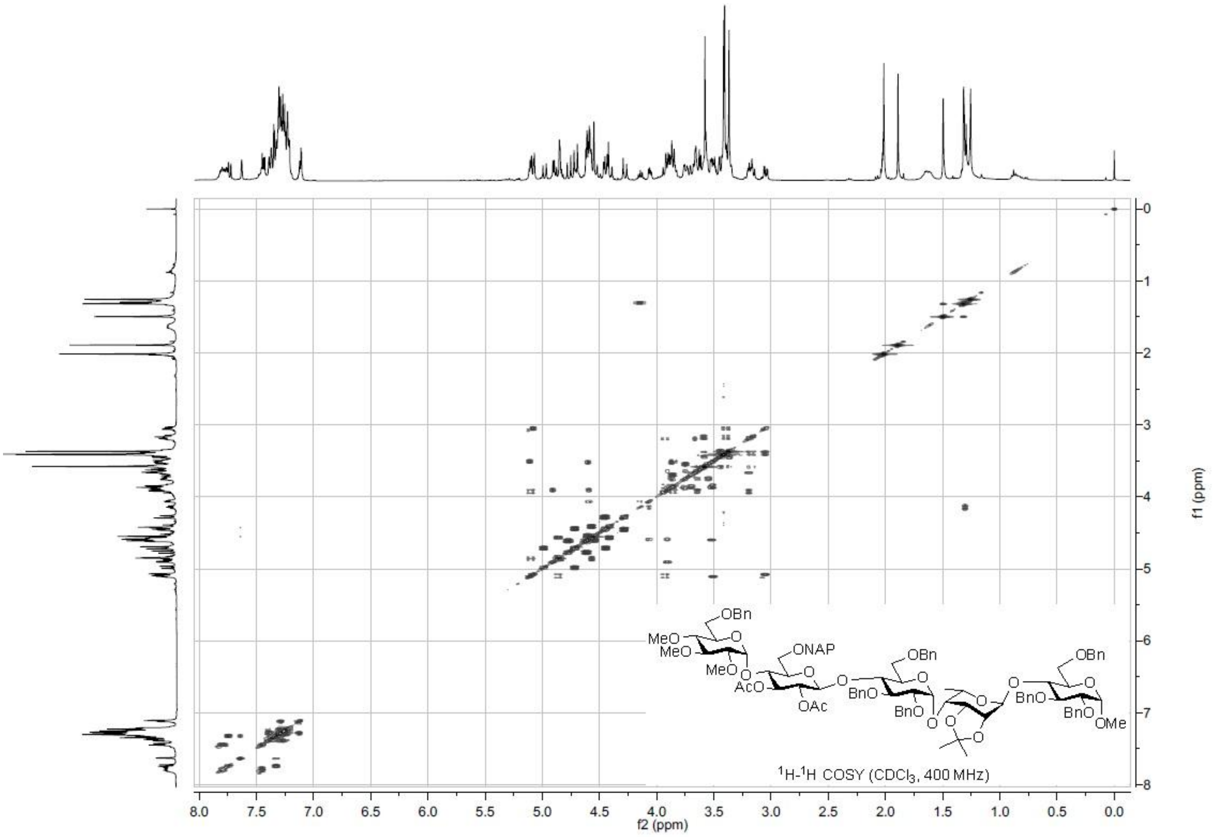
OBn
MeO
MeO
MeO
ONAP
AcO
OAc
OBn
BnO
BnO
OBn
BnO
BnO
OMe
$^1$H-$^1$H COSY (CDCl$_3$, 400 MHz)
f2 (ppm)
f1 (ppm)

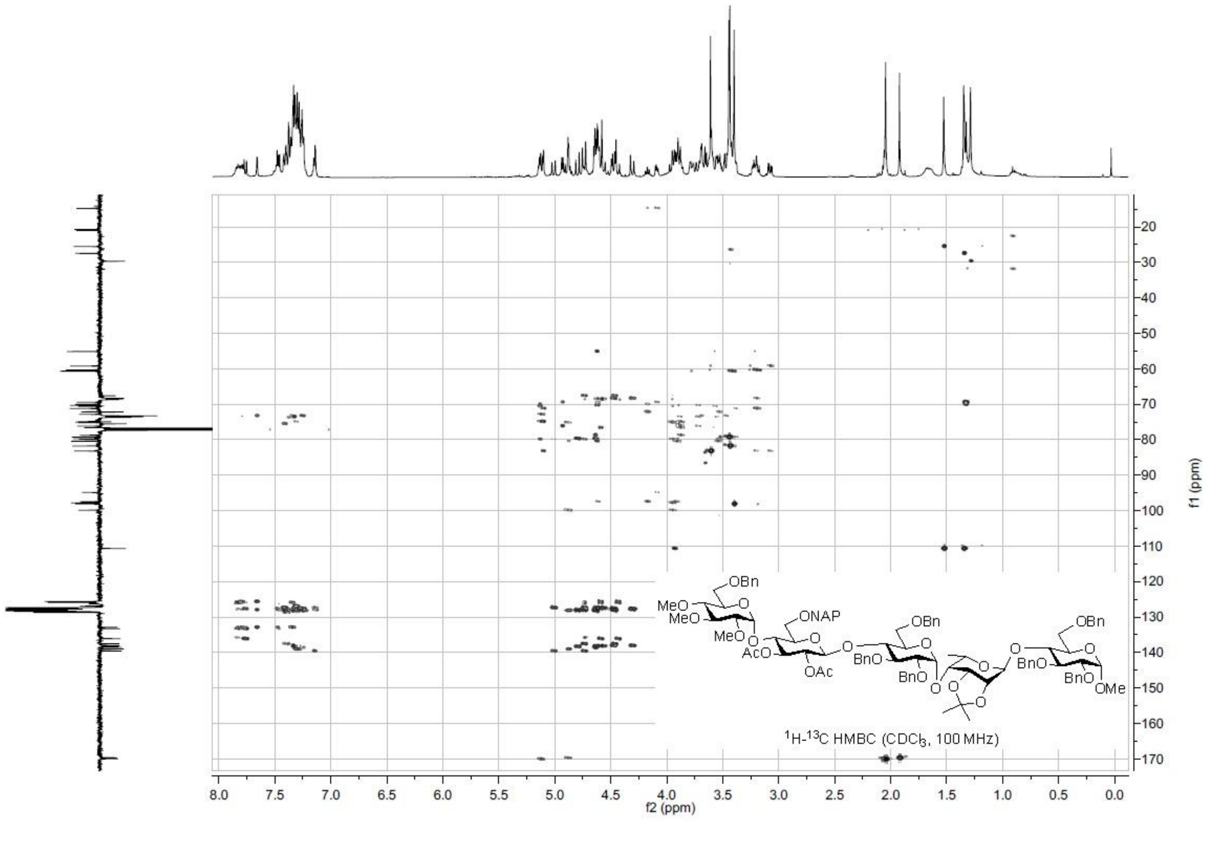
OBn
MeO
MeO
MeO
ONAP
AcO
OAc
OBn
BnO
BnO
OBn
BnO
BnO
OMe
$^1$H-$^{13}$C HMBC (CDCl$_3$, 100 MHz)
f2 (ppm)
f1 (ppm)

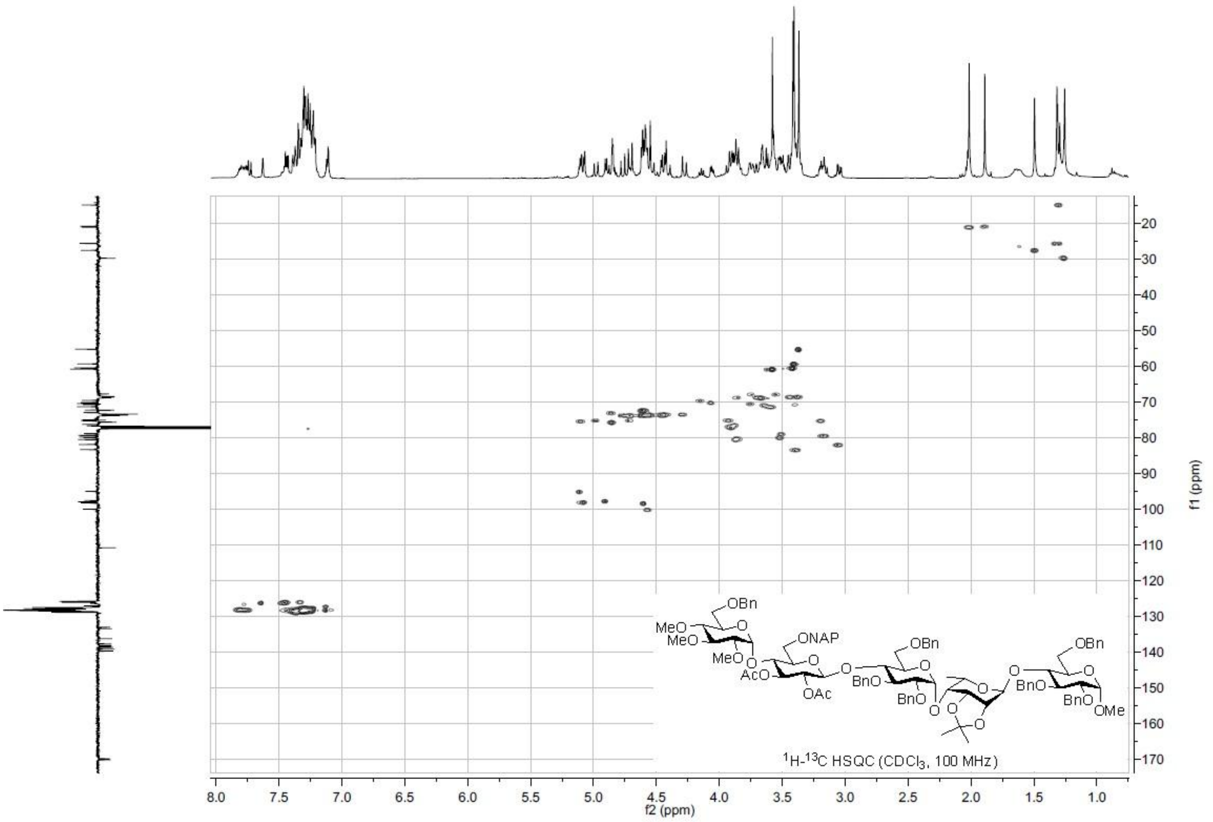


**$^{1}$H and $^{13}$C NMR spectra of compound 27:**

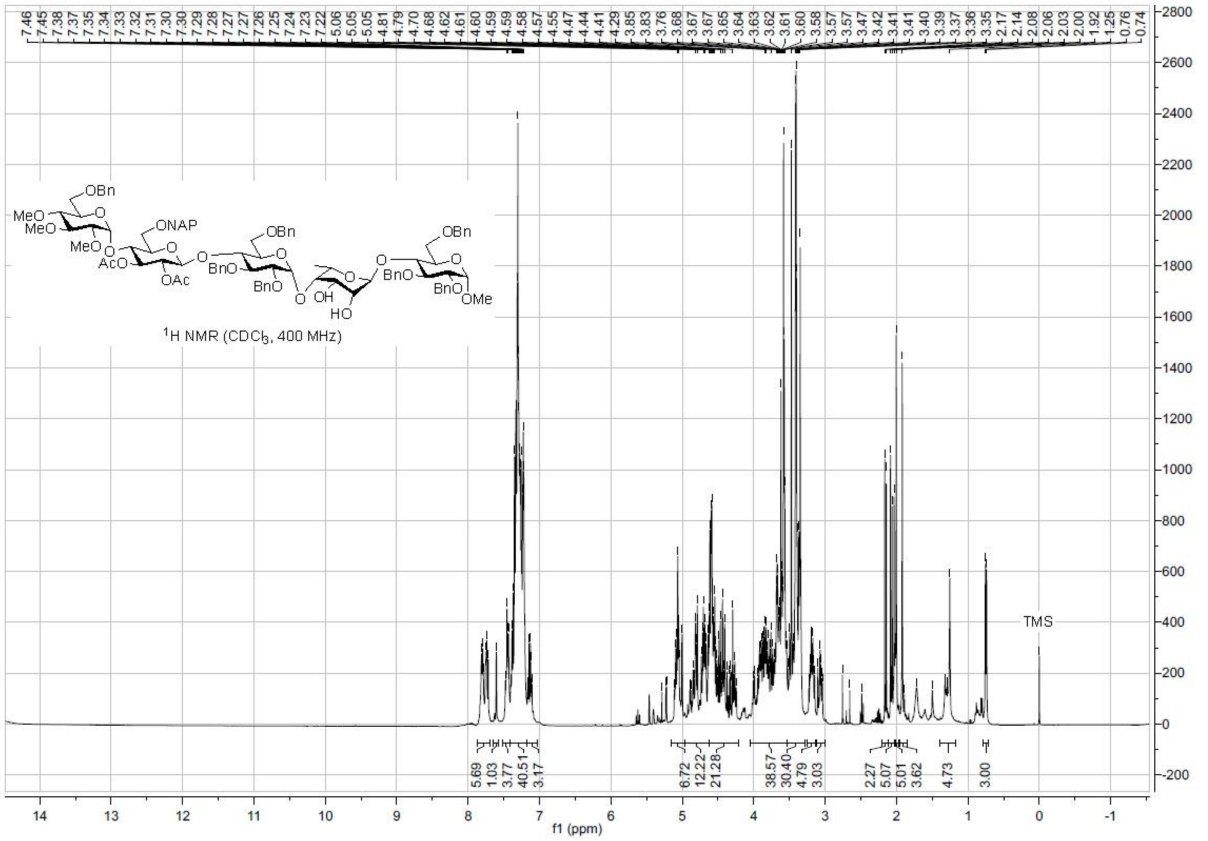

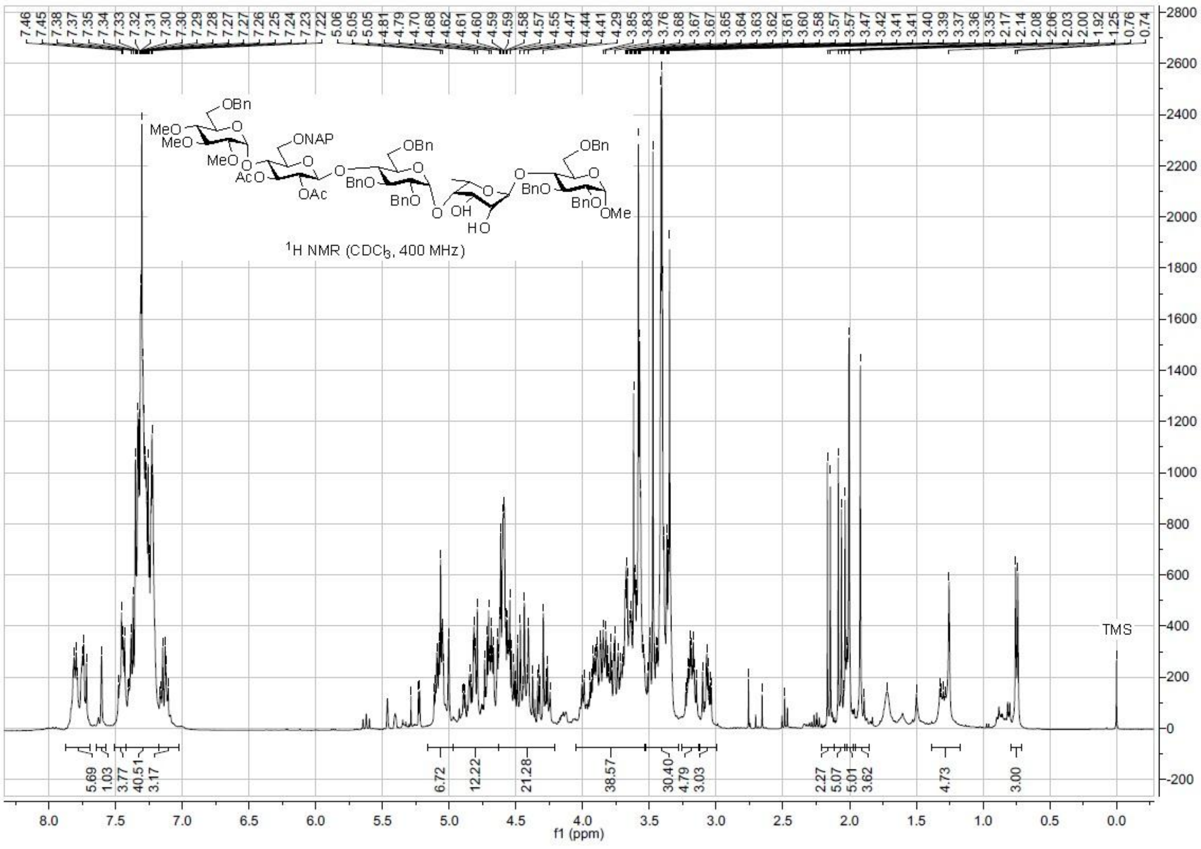
7.46 7.45 7.38 7.37 7.35 7.34 7.33 7.32 7.31 7.30 7.29 7.28 7.27 7.26 7.25 7.24 7.23 7.22 5.06 5.05 4.81 4.79 4.70 4.68 4.62 4.61 4.60 4.59 4.59 4.58 4.57 4.55 4.47 4.44 4.41 4.29 3.85 3.83 3.76 3.68 3.67 3.65 3.64 3.63 3.62 3.61 3.60 3.58 3.57 3.57 3.47 3.42 3.41 3.41 3.40 3.39 3.37 3.36 3.35 2.17 2.14 2.08 2.06 2.03 2.00 1.92 1.25 0.76 0.74
OBn
MeO
MeO
MeO
ONAP
AcO
OAc
OBn
BnO
BnO
O
OH
HO
OBn
BnO
BnO
OMe
1H NMR (CDCl3, 400 MHz)
TMS
5.69 1.03 3.77 40.51 3.17 6.72 12.22 21.28 38.57 30.40 4.79 3.03 2.27 5.07 5.01 3.62 4.73 3.00
f1 (ppm)

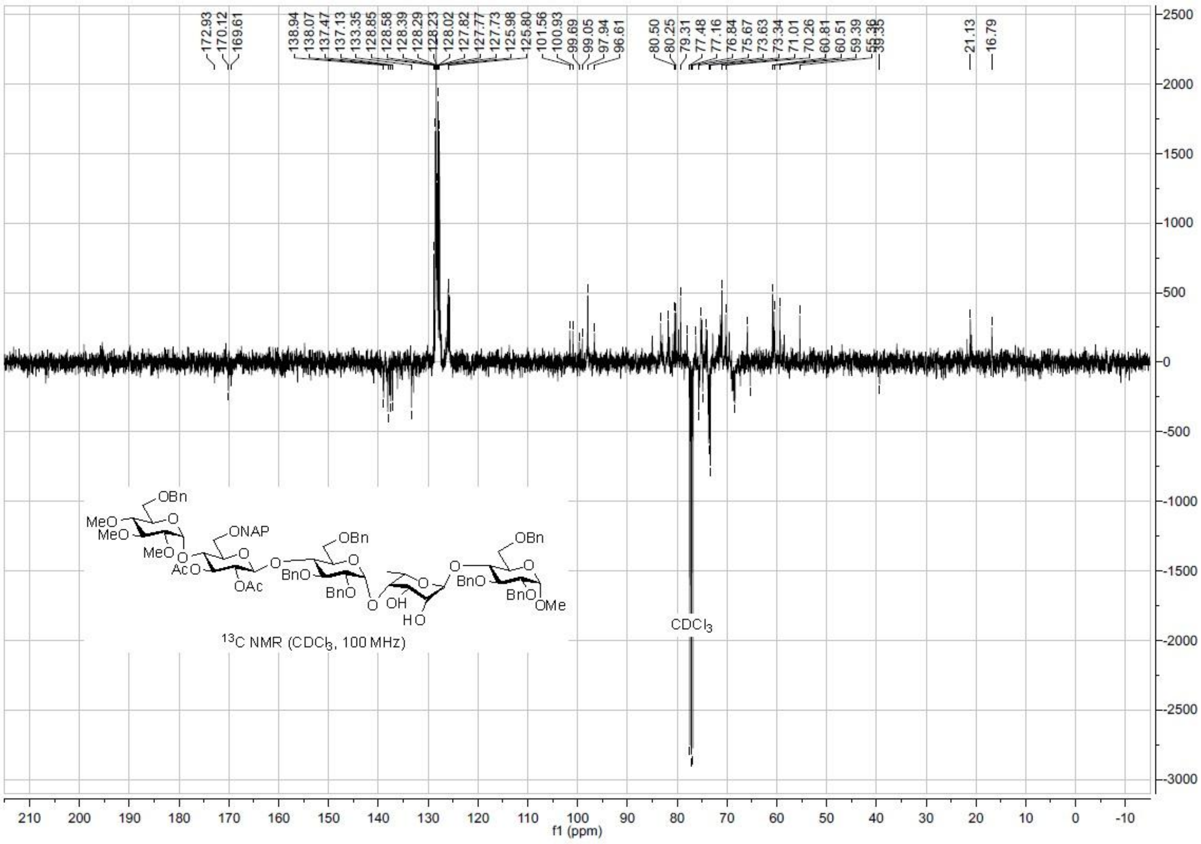
172.93 170.12 169.61 138.94 138.07 137.47 137.13 133.35 128.85 128.58 128.39 128.29 128.23 128.02 127.82 127.77 127.73 125.98 125.80 101.56 100.93 99.69 99.05 97.94 96.61 80.50 80.25 79.31 77.48 77.16 76.84 75.67 73.63 73.34 71.01 70.26 60.81 60.51 59.39 55.36 39.35 21.13 16.79
OBn
MeO
MeO
MeO
ONAP
AcO
OAc
OBn
BnO
BnO
O
OH
HO
OBn
BnO
BnO
OMe
13C NMR (CDCl3, 100 MHz)
CDCl3
f1 (ppm)

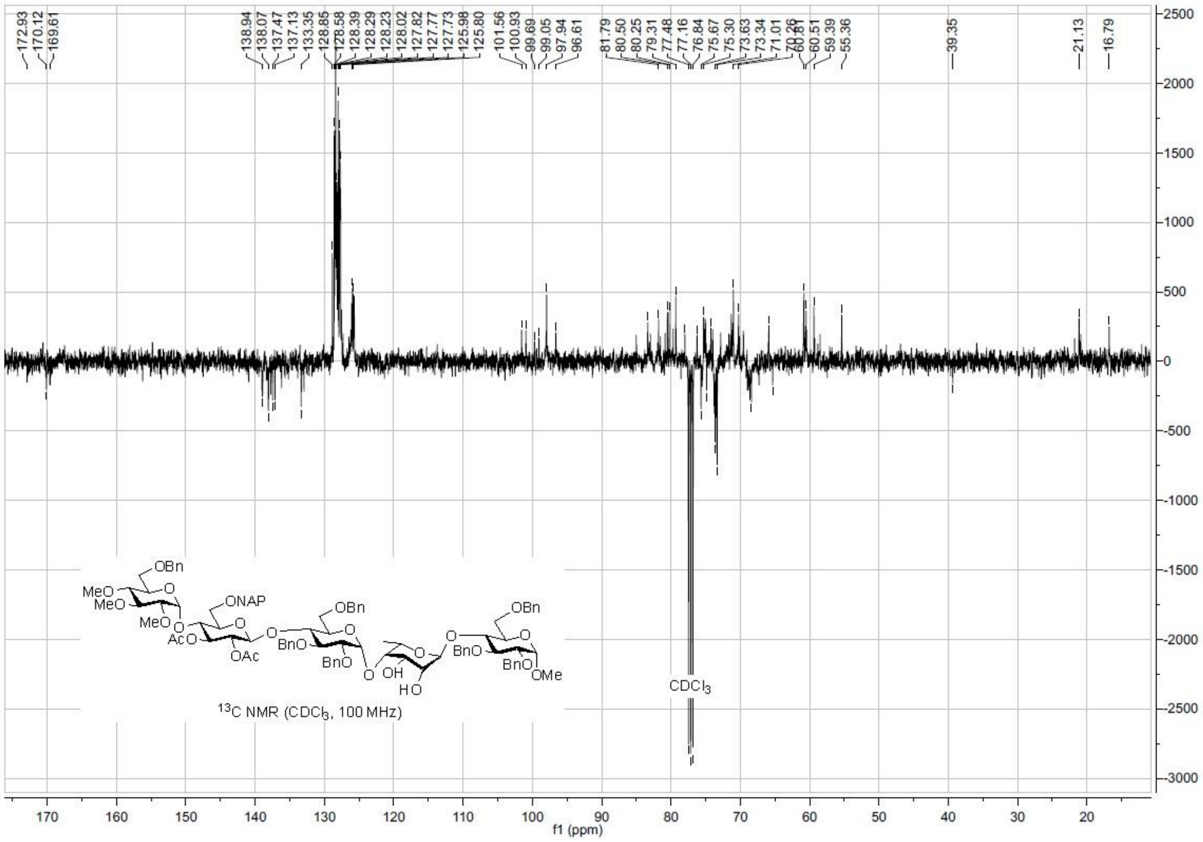

**$^{1}$H and $^{13}$C NMR spectra of compound 28:**

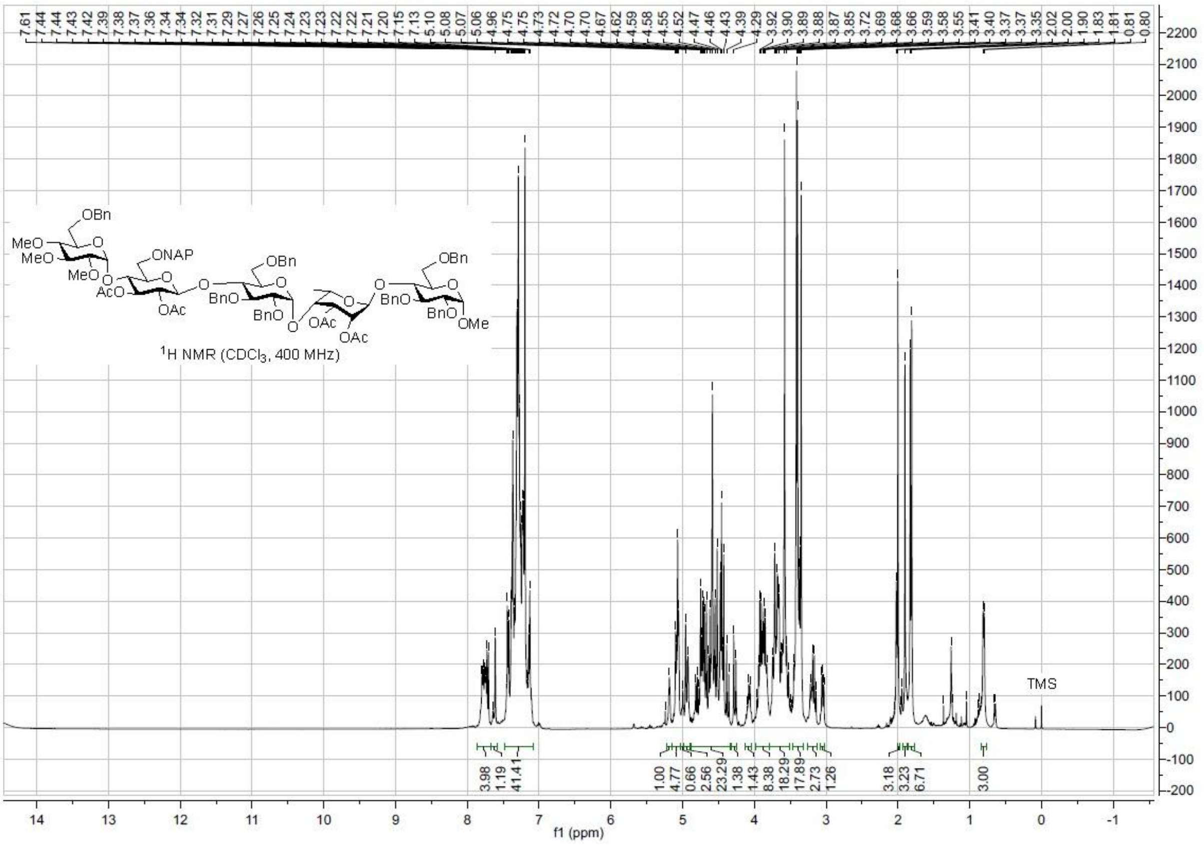

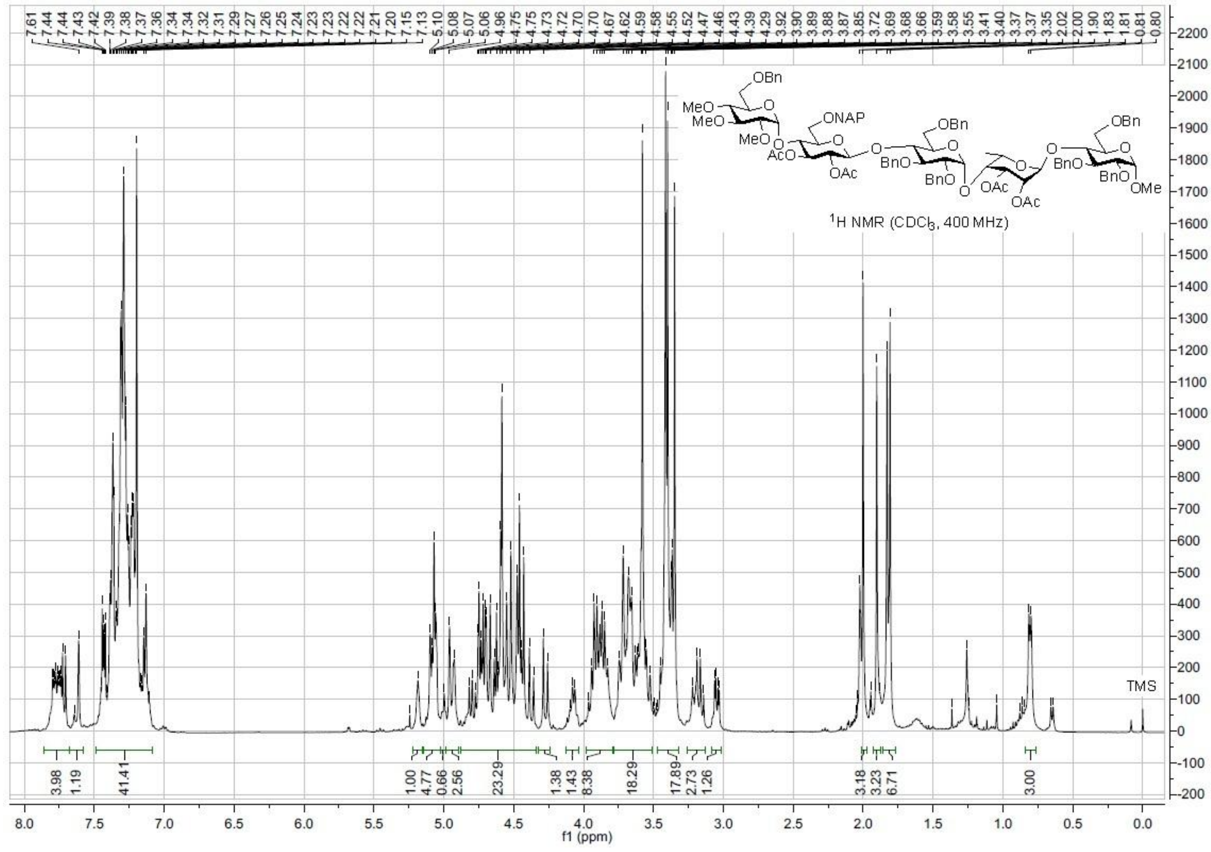

$^1$H NMR (CDCl$_3$, 400 MHz)
TMS
f1 (ppm)


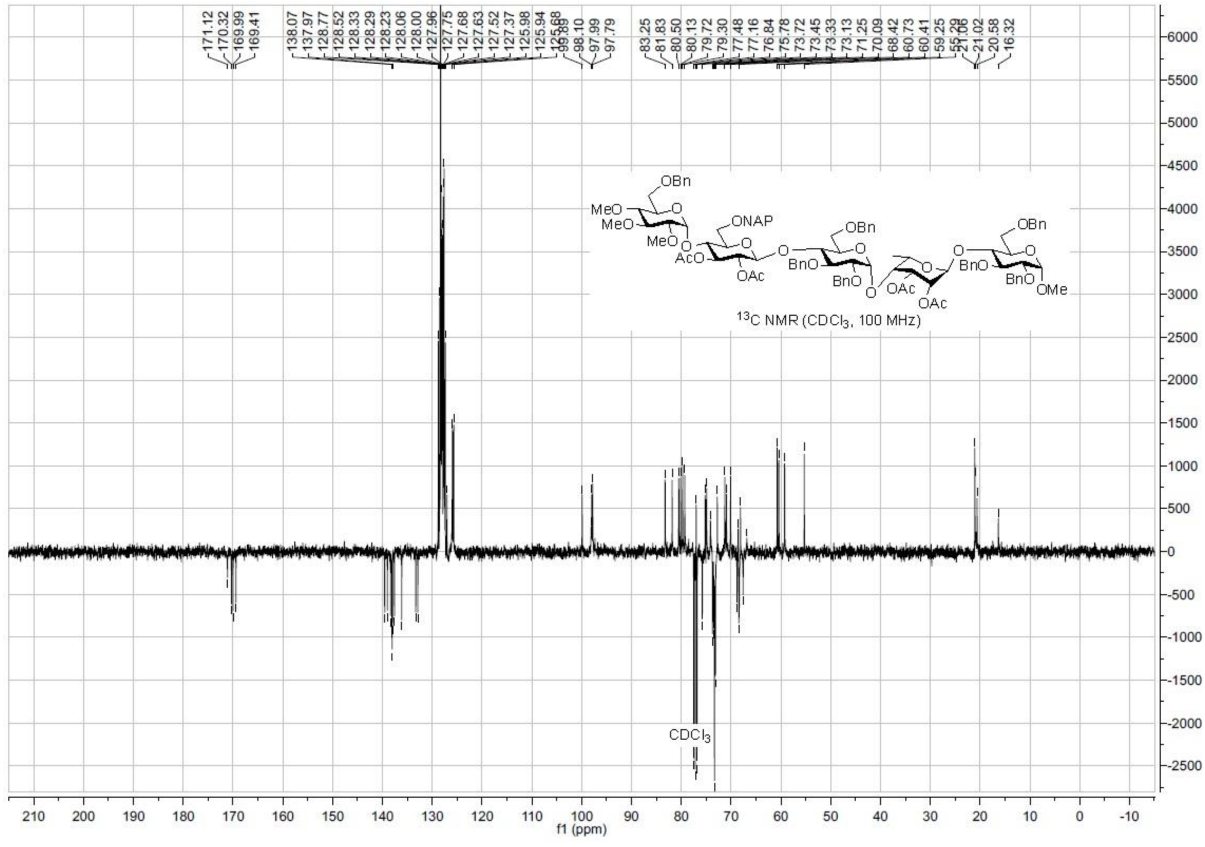

$^{13}$C NMR (CDCl$_3$, 100 MHz)
CDCl$_3$
f1 (ppm)

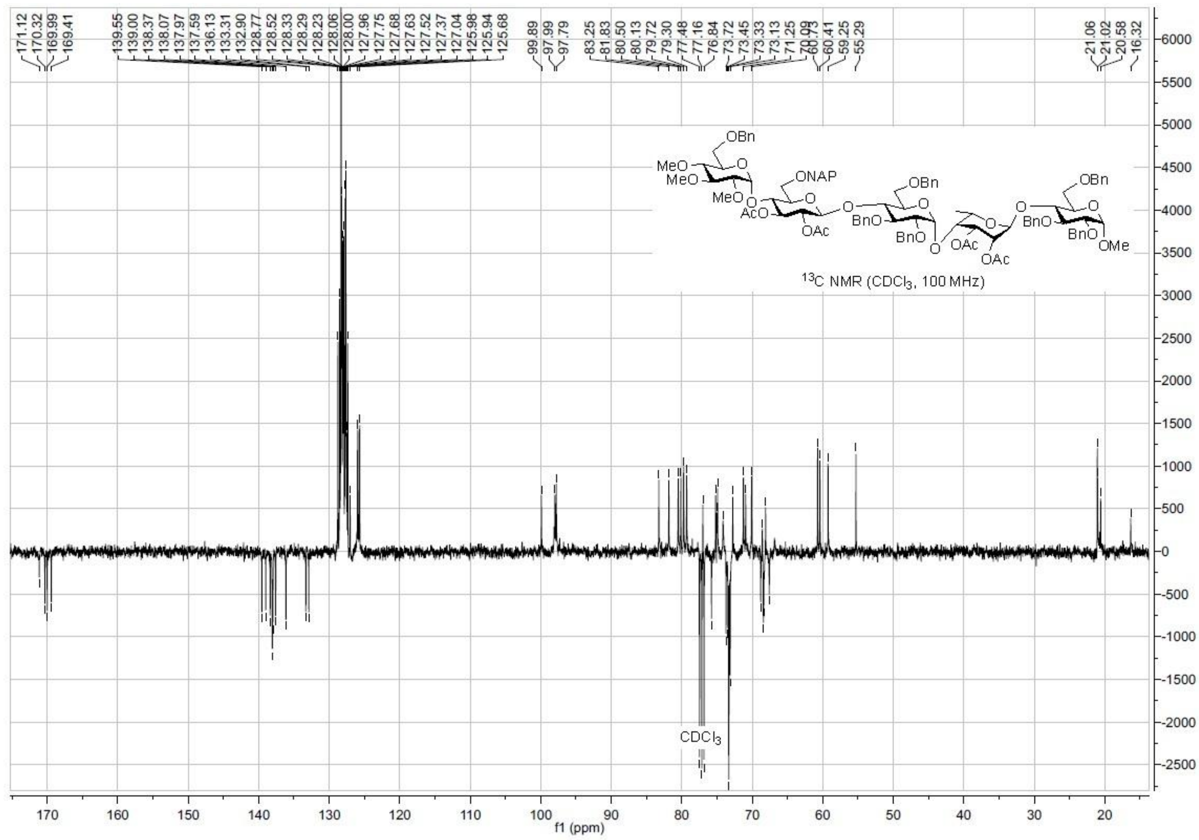
171.12
170.32
169.99
169.41
139.55
139.00
138.37
138.07
137.97
137.59
136.13
133.31
132.90
128.77
128.52
128.33
128.29
128.23
128.06
128.00
127.96
127.75
127.68
127.63
127.52
127.37
127.04
125.98
125.94
125.68
99.89
97.99
97.79
83.25
81.83
80.50
80.13
79.72
79.30
77.48
77.16
76.84
73.72
73.45
73.33
73.13
71.25
60.41
59.25
55.29
21.06
21.02
20.58
16.32
$^{13}$C NMR (CDCl$_3$, 100 MHz)
CDCl$_3$
f1 (ppm)

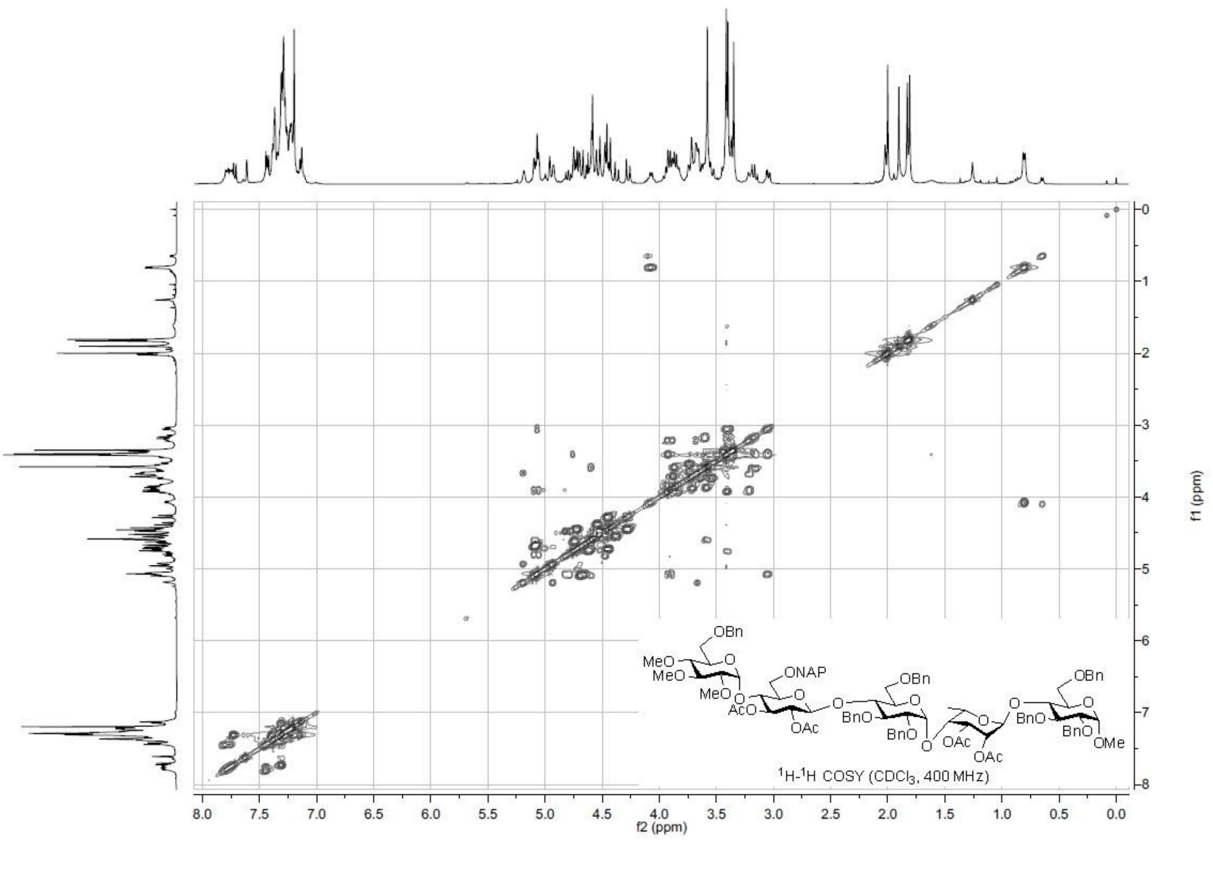
$^1$H-$^1$H COSY (CDCl$_3$, 400 MHz)
f2 (ppm)
f1 (ppm)

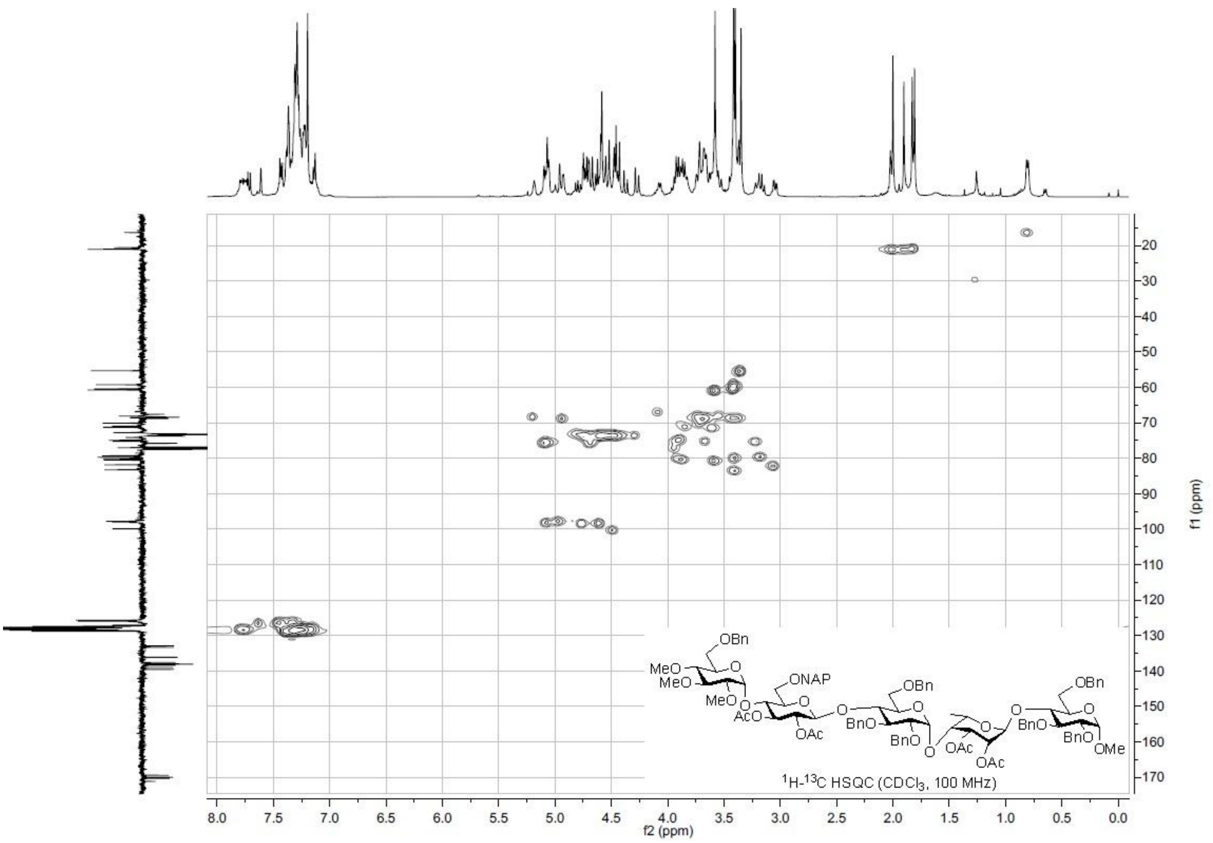


**$^{1}$H and $^{13}$C NMR spectra of compound 29:**

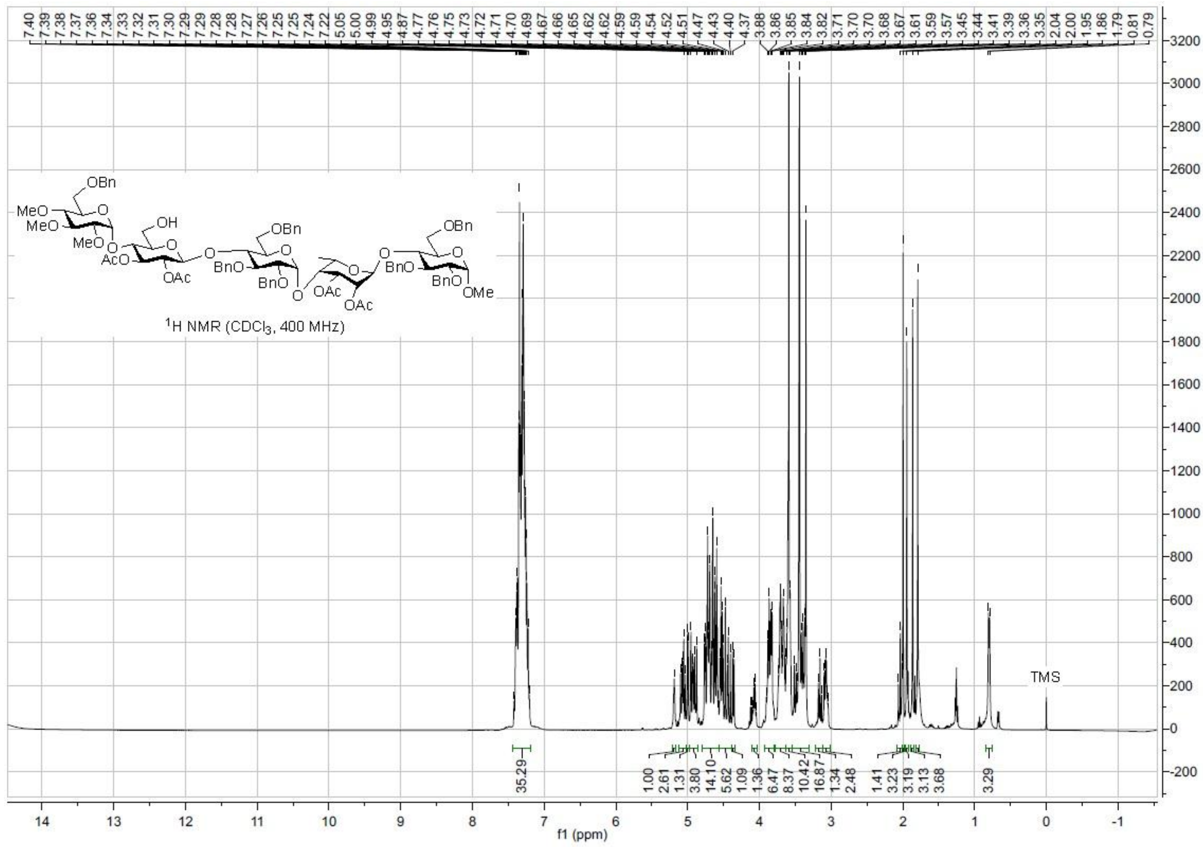

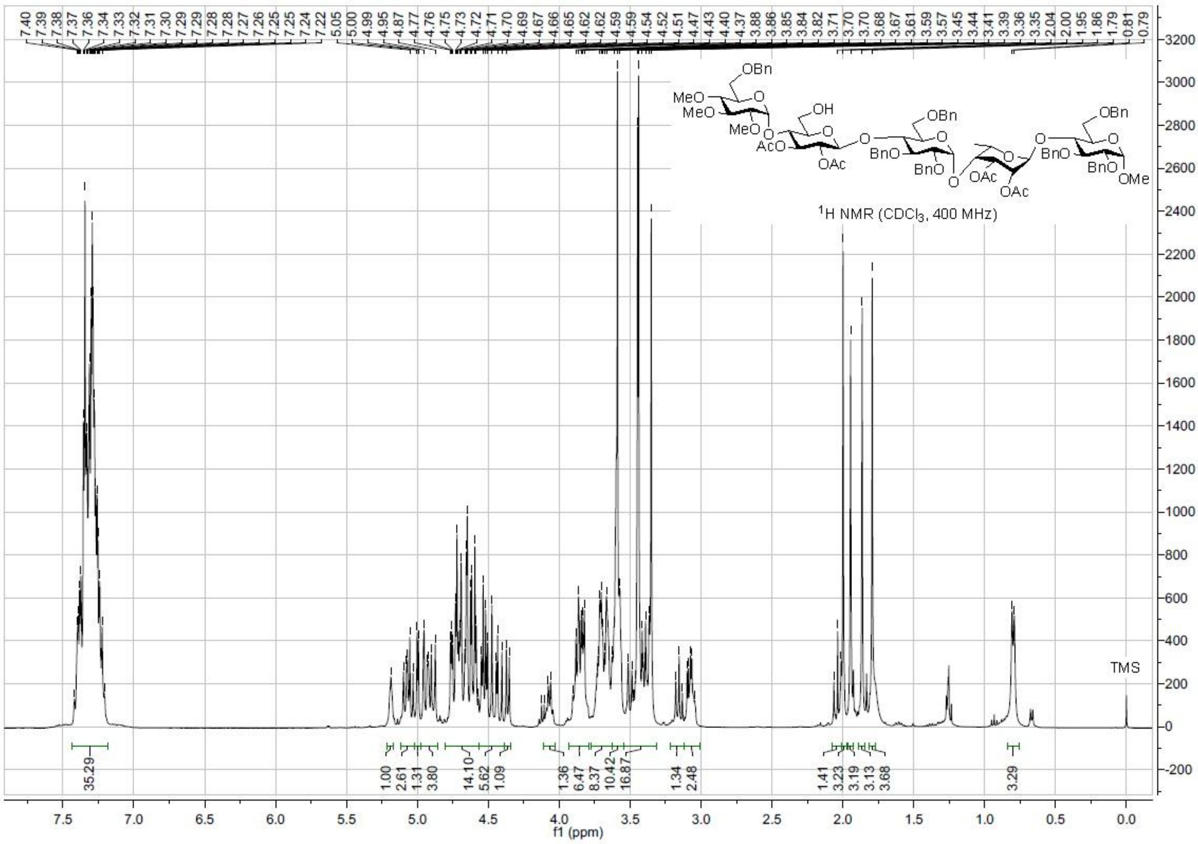
1H NMR (CDCl3, 400 MHz)
TMS
f1 (ppm)

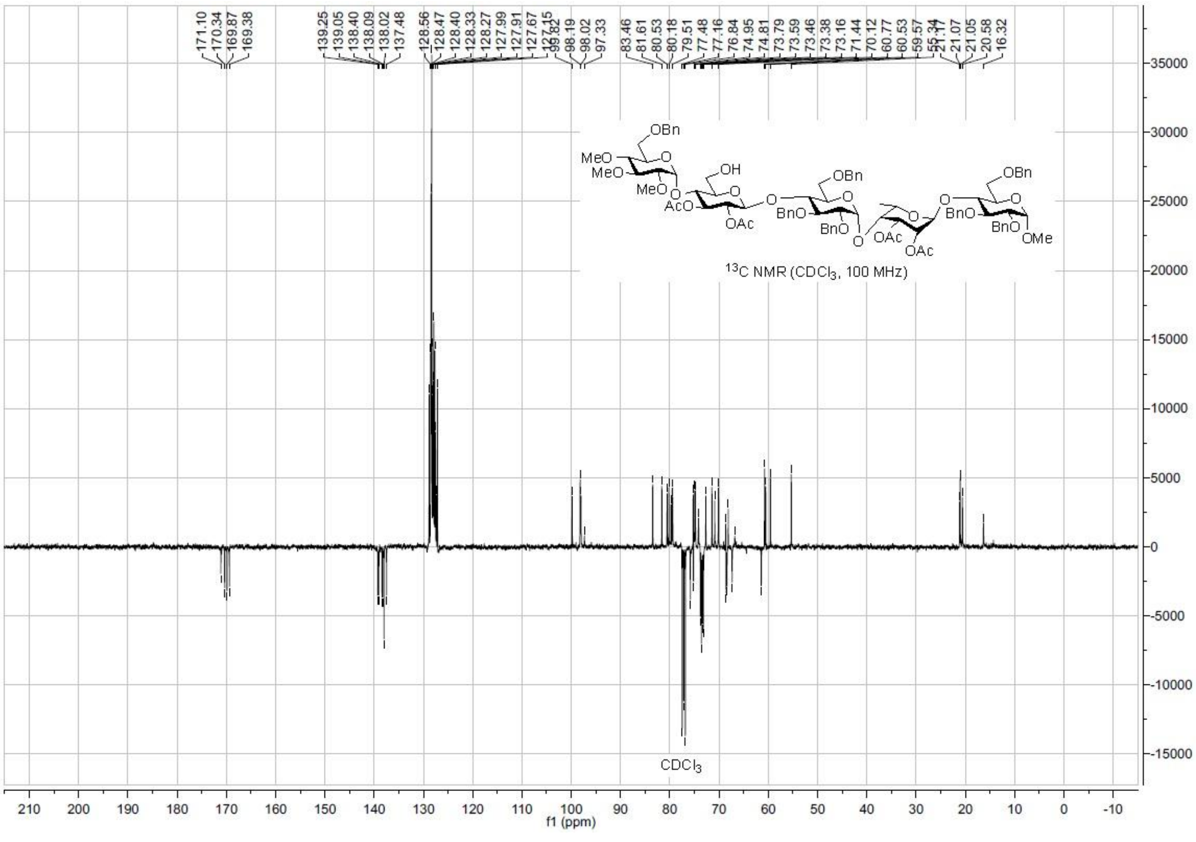
13C NMR (CDCl3, 100 MHz)
CDCl3
f1 (ppm)

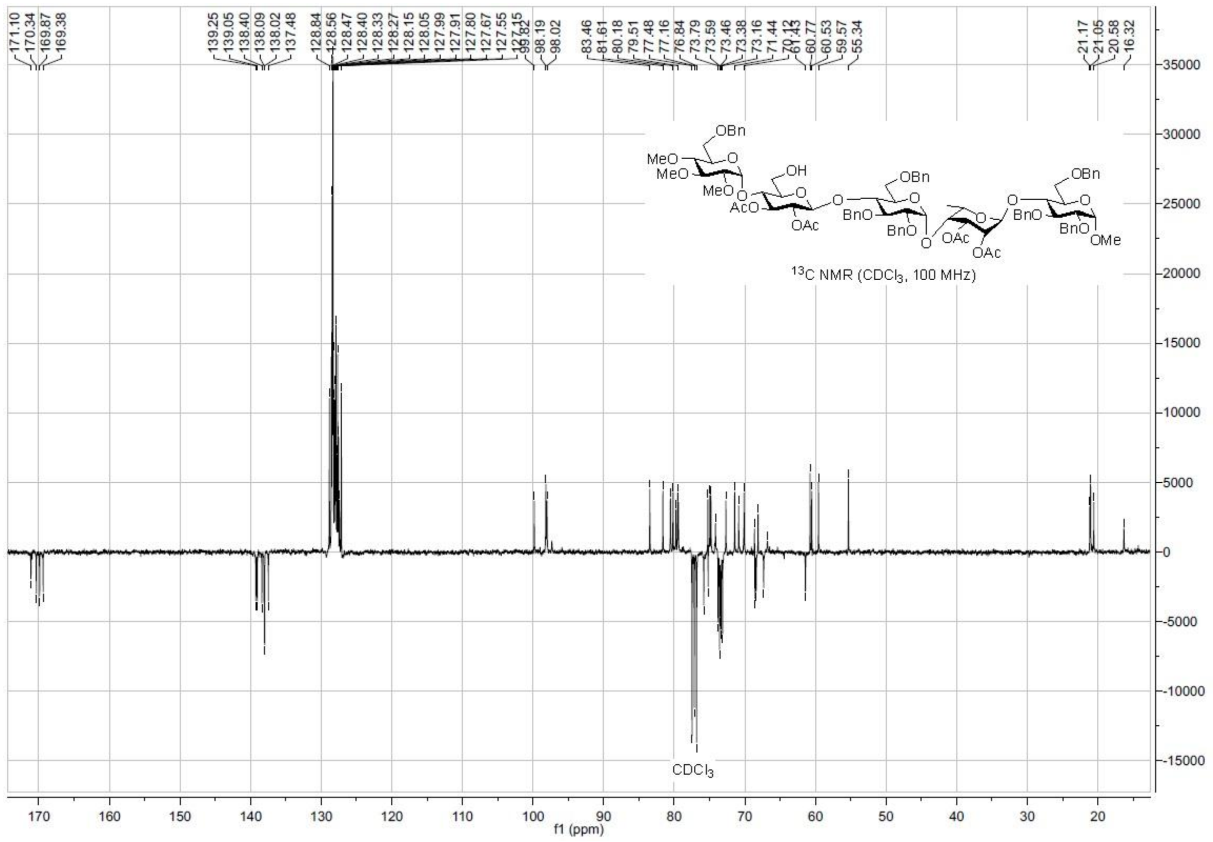

171.10
170.34
169.87
169.38
139.25
139.05
138.40
138.09
138.02
137.48
128.84
128.56
128.47
128.40
128.33
128.27
128.15
128.05
127.99
127.91
127.80
127.67
127.55
98.19
98.02
83.46
81.61
80.18
79.51
77.48
77.16
76.84
73.79
73.59
73.46
73.38
73.16
71.44
60.77
60.53
59.57
55.34
21.17
21.05
20.58
16.32
$^{13}$C NMR (CDCl$_3$, 100 MHz)
CDCl$_3$
f1 (ppm)


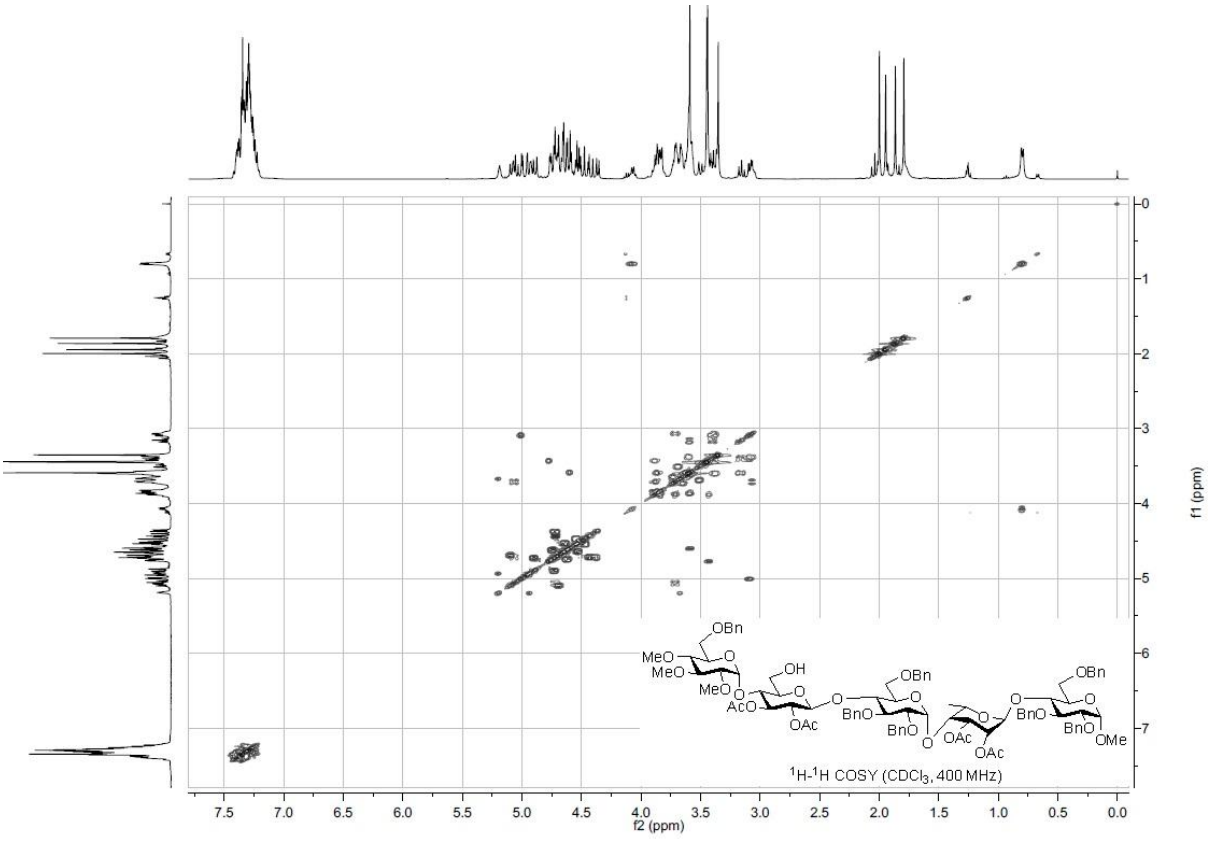

$^1$H-$^1$H COSY (CDCl$_3$, 400 MHz)
f2 (ppm)
f1 (ppm)

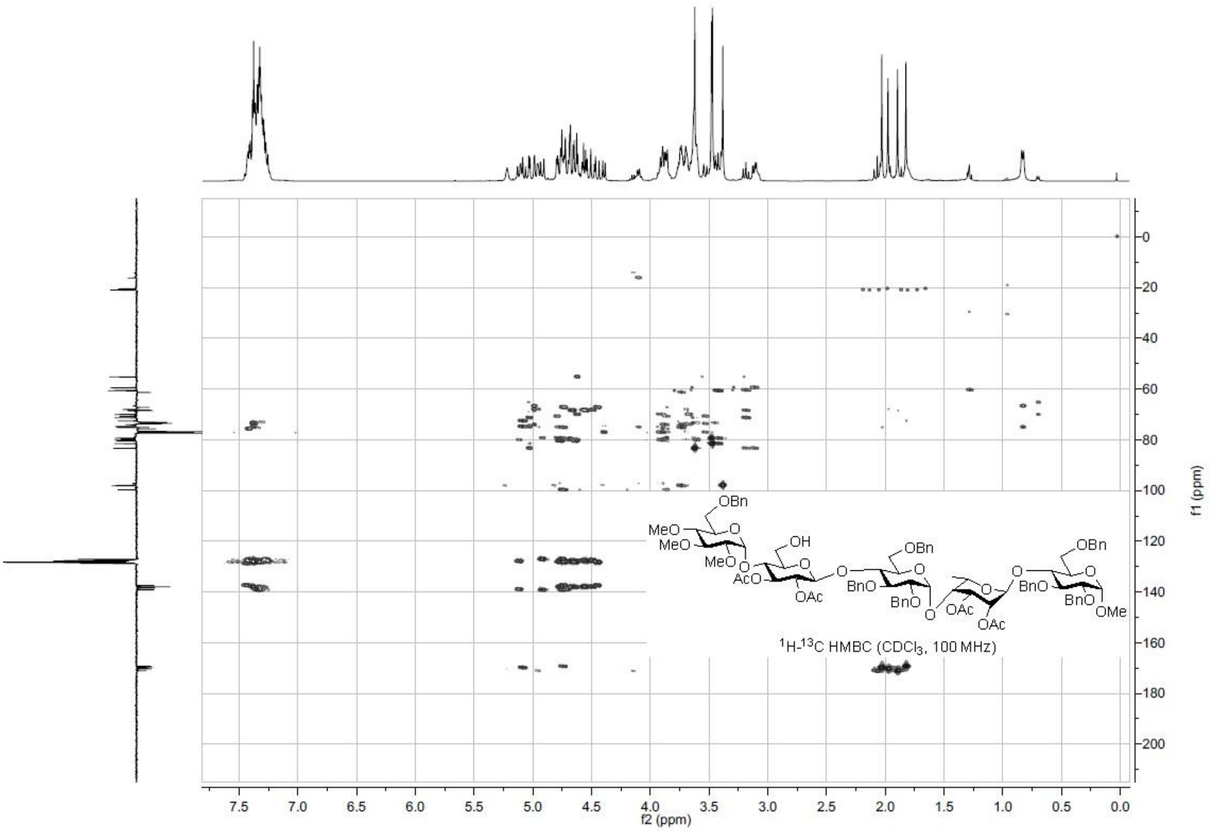

1H-13C HMBC (CDCl3, 100 MHz)
f1 (ppm)
f2 (ppm)


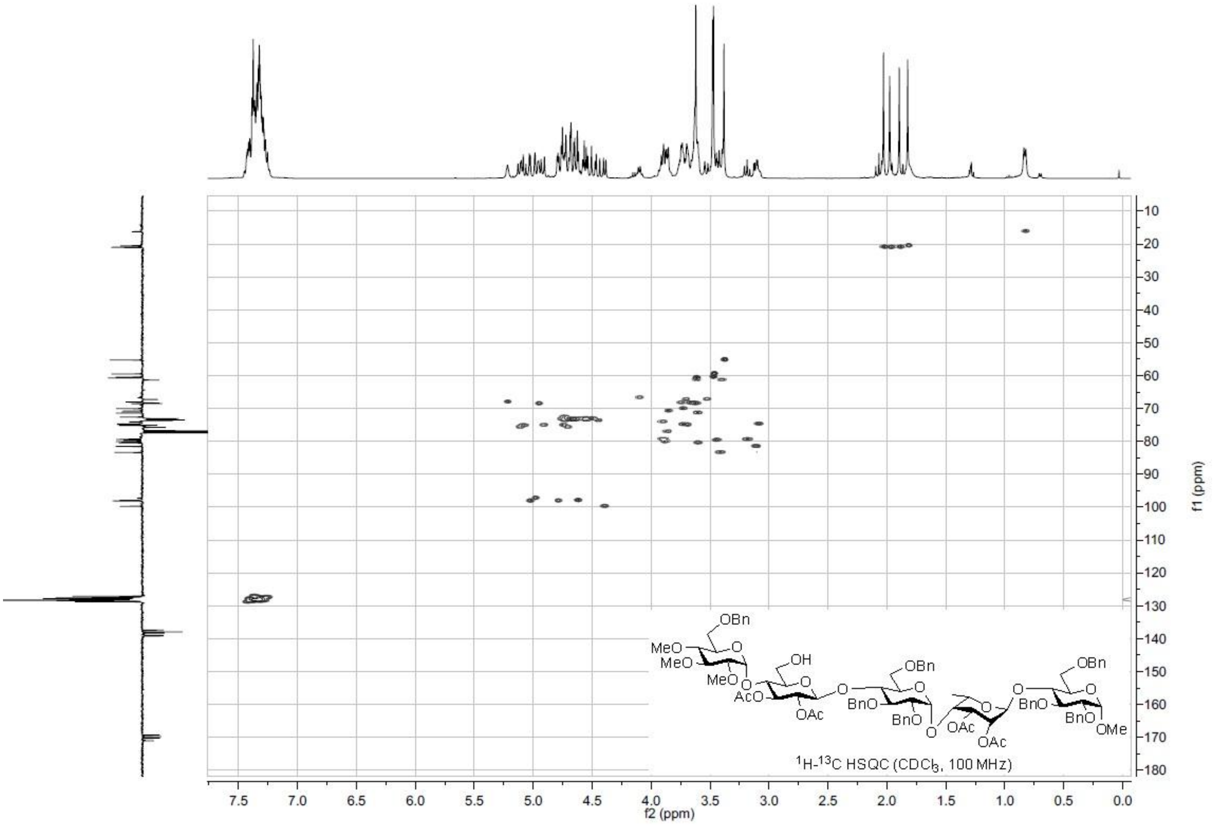

1H-13C HSQC (CDCl3, 100 MHz)
f1 (ppm)
f2 (ppm)

# $^{1}$H and $^{13}$C NMR spectra of compound 30:

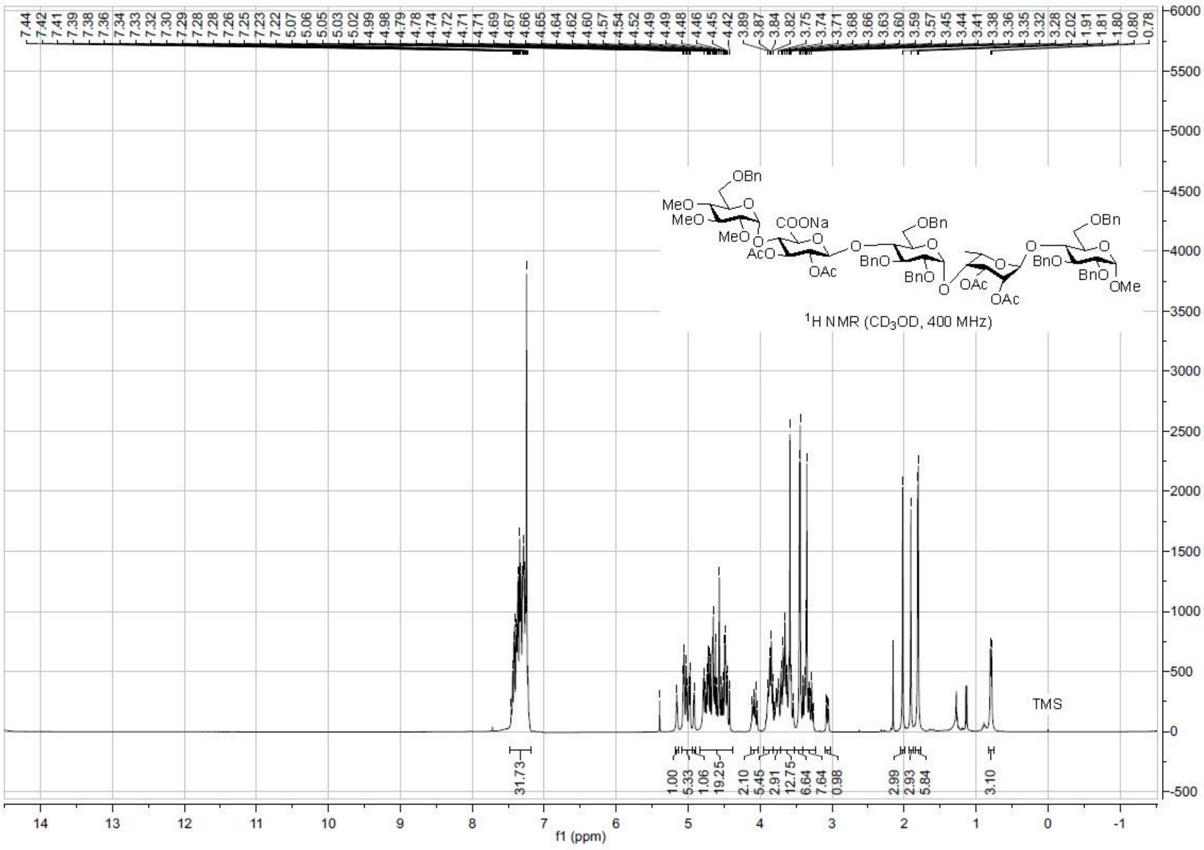


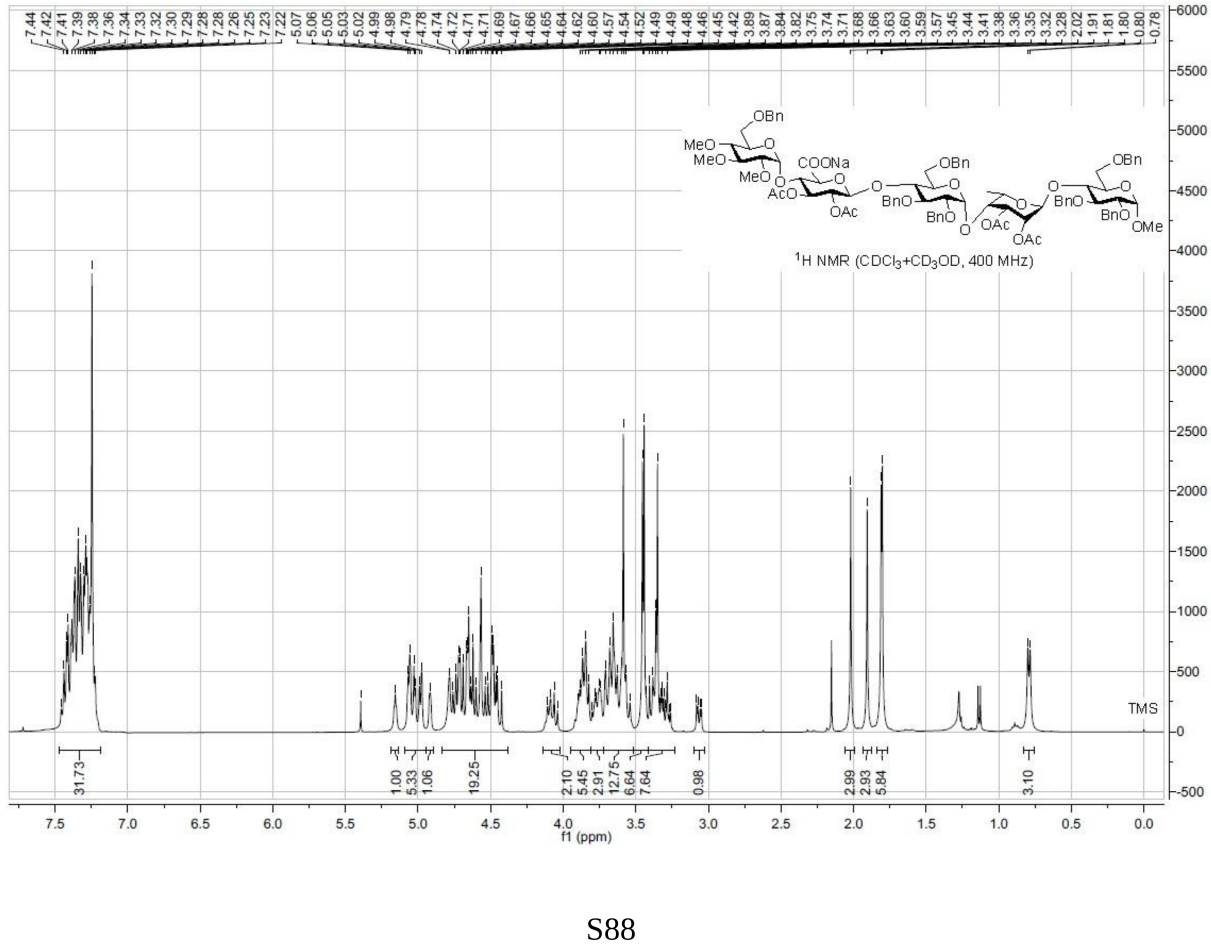

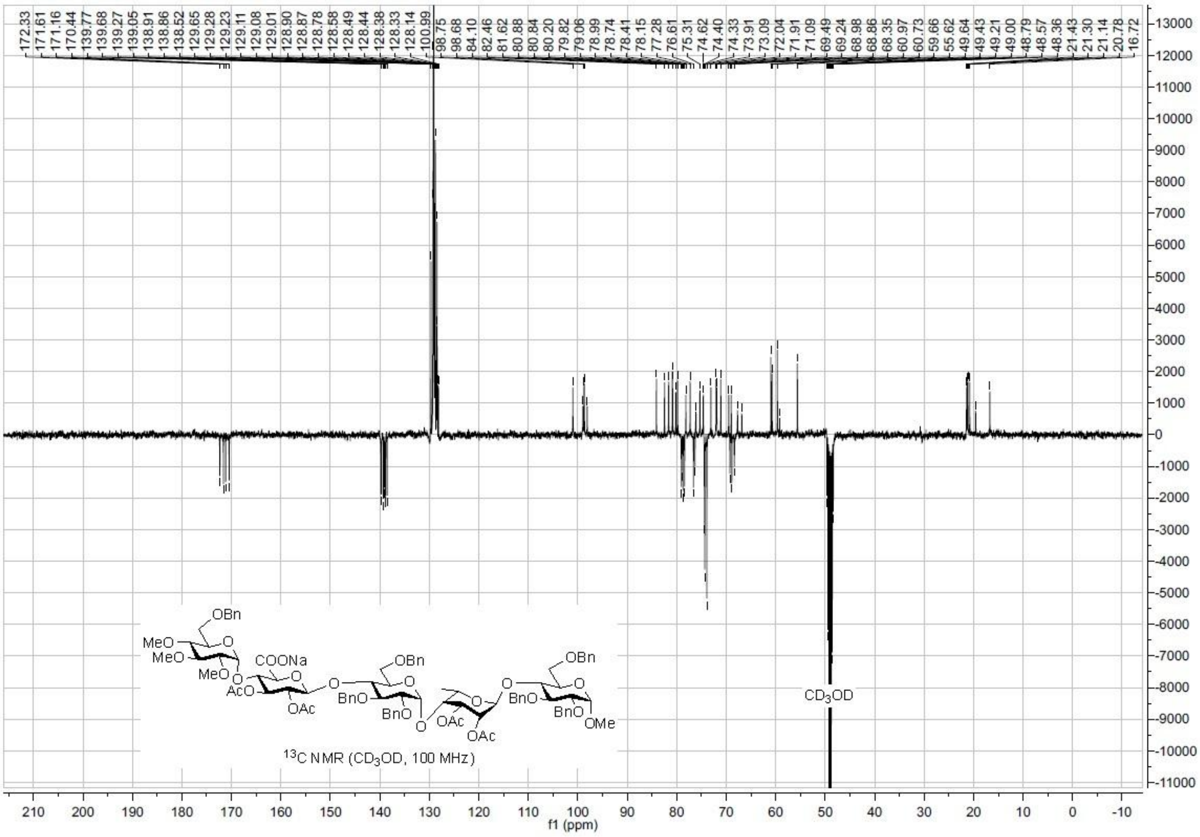
13C NMR (CD3OD, 100 MHz)
CD3OD
f1 (ppm)

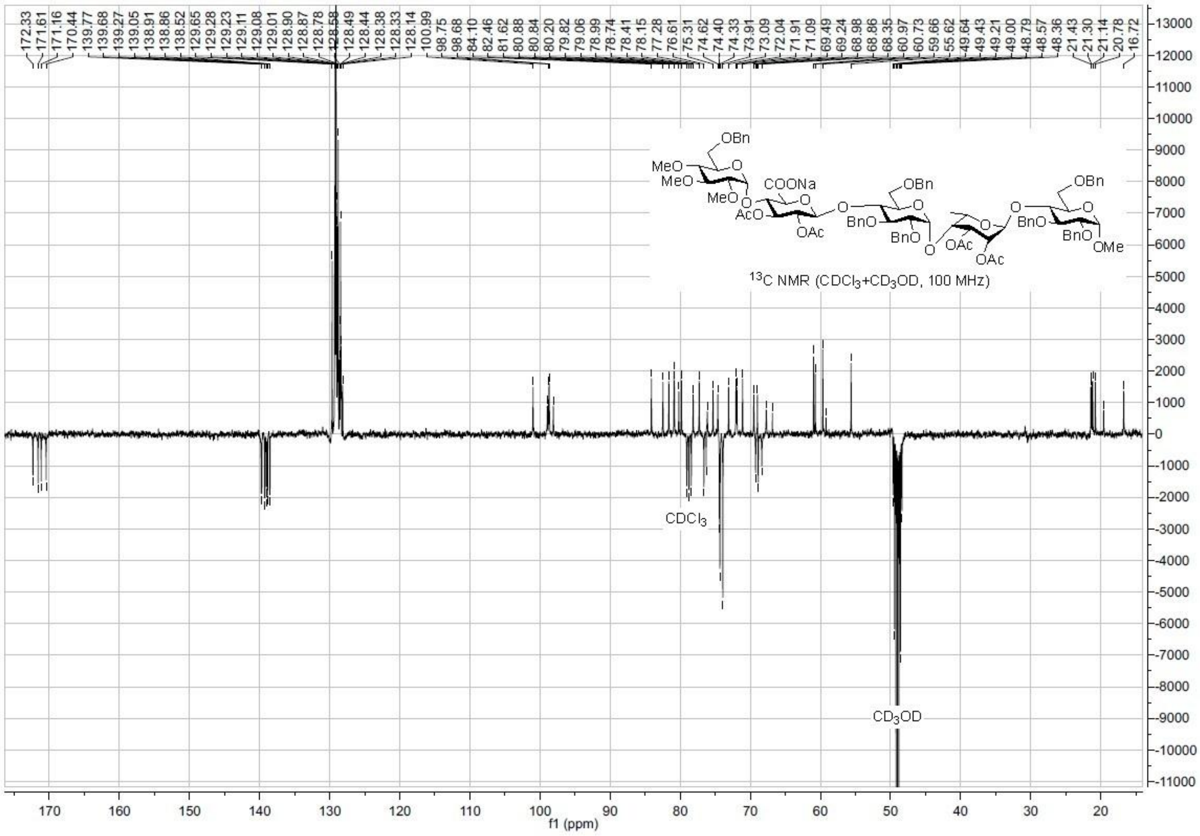
13C NMR (CDCl3+CD3OD, 100 MHz)
CDCl3
CD3OD
f1 (ppm)

**$^{1}$H and $^{13}$C NMR spectra of compound 31:**

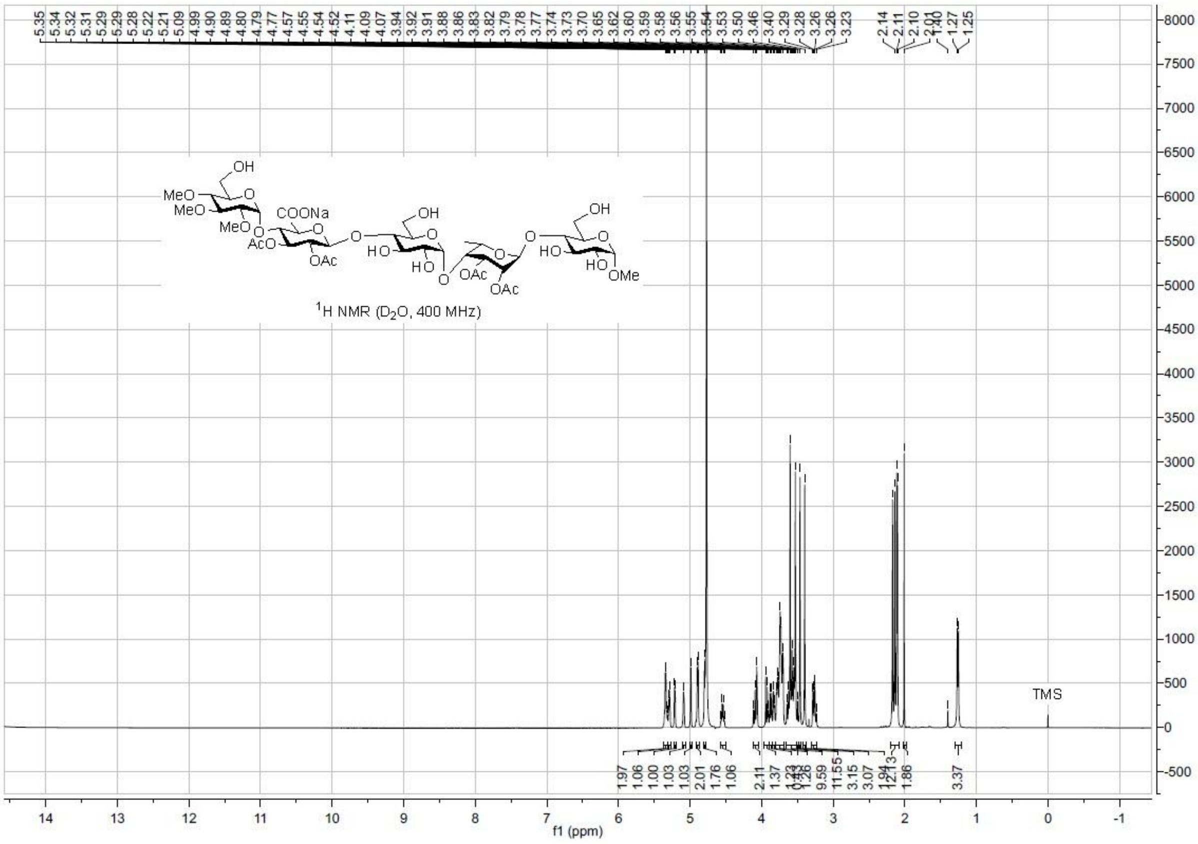


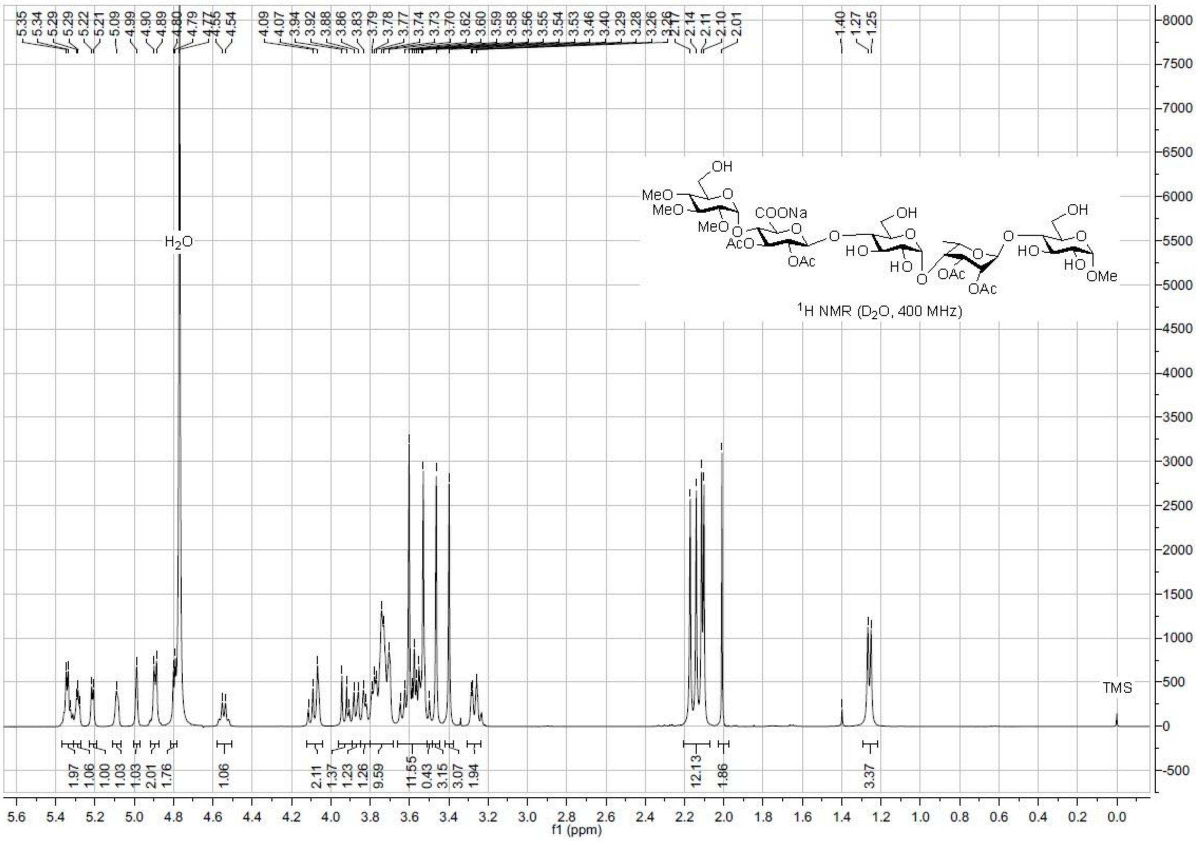

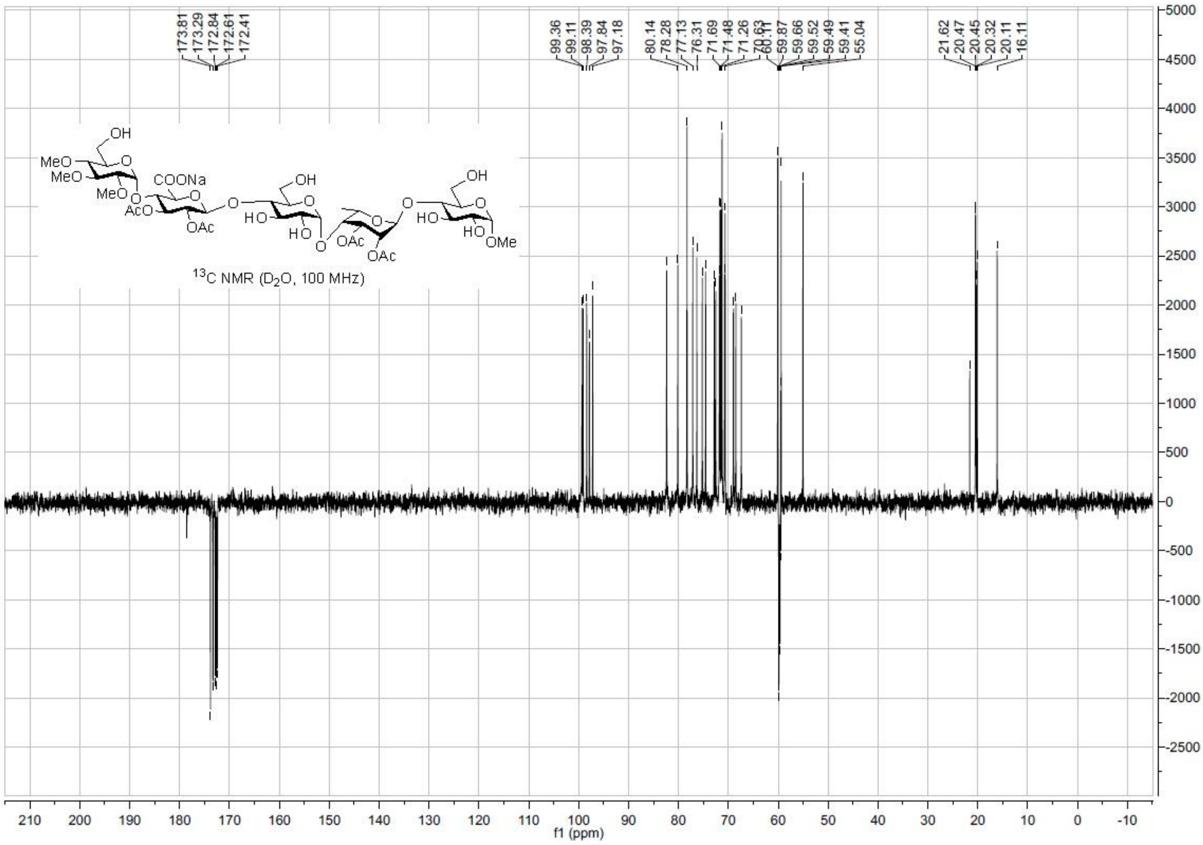

13C NMR (D2O, 100 MHz)
f1 (ppm)


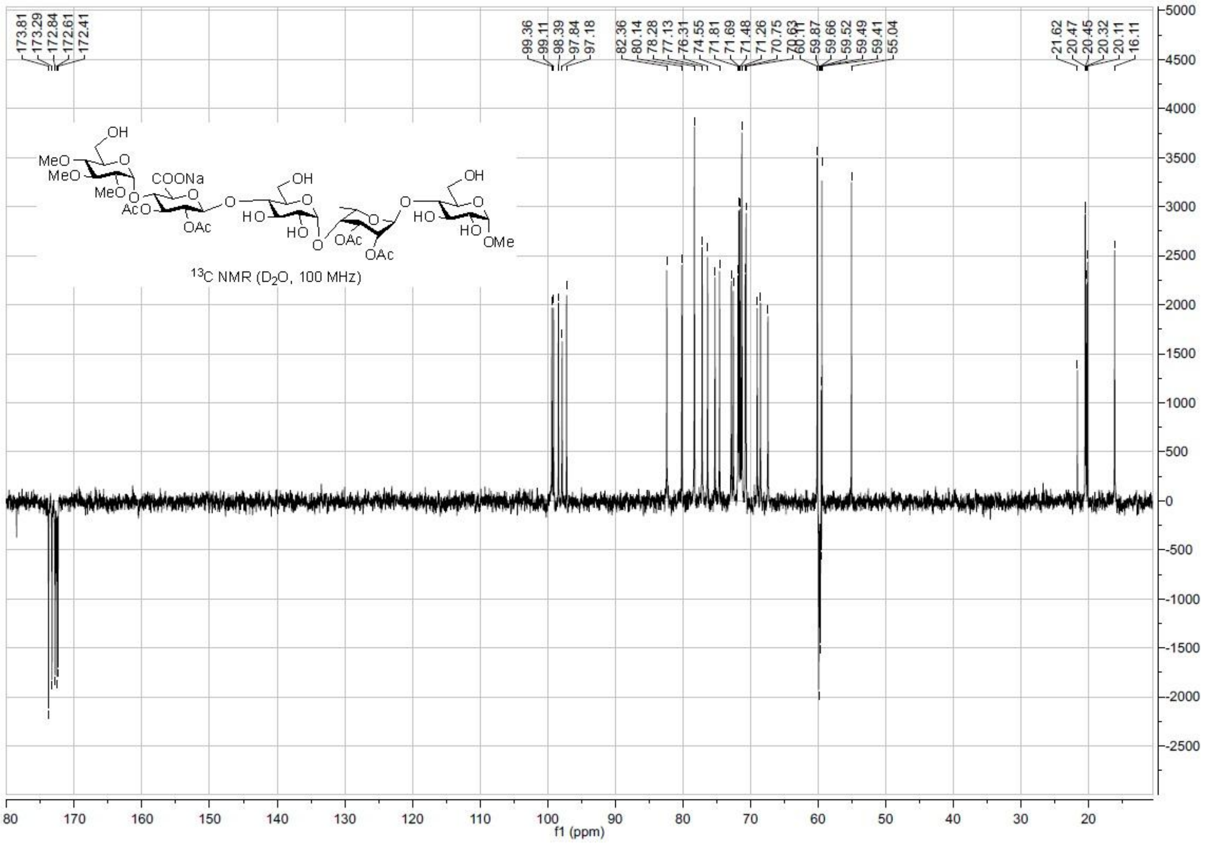

13C NMR (D2O, 100 MHz)
f1 (ppm)

**$^{1}$H and $^{13}$C NMR spectra of compound 32:**

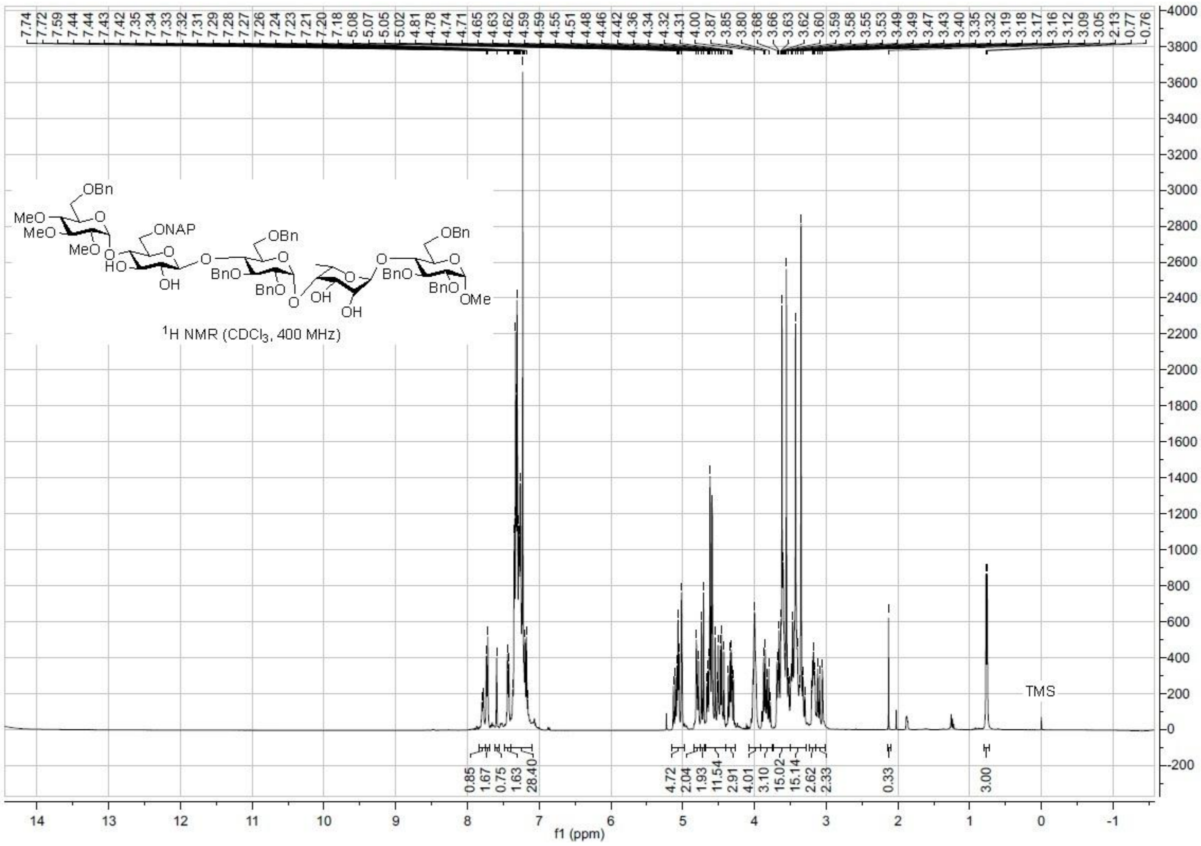


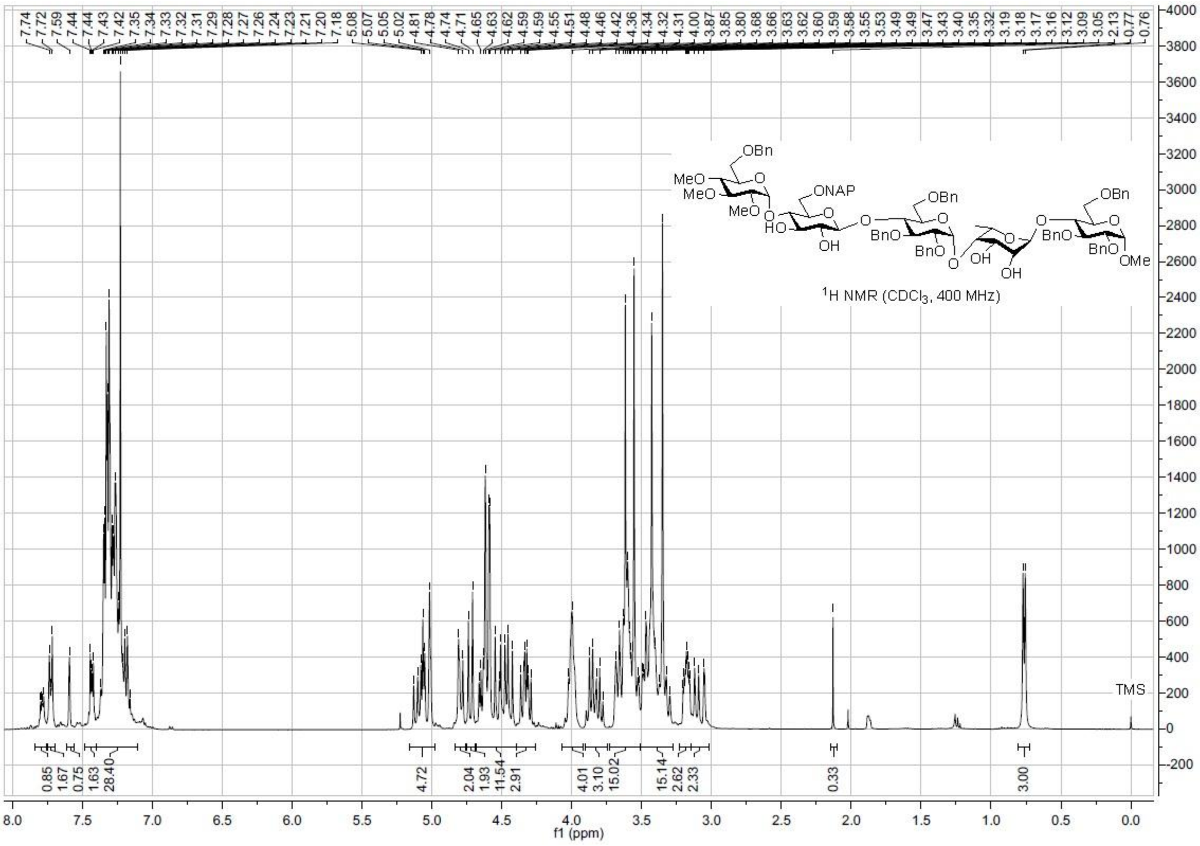

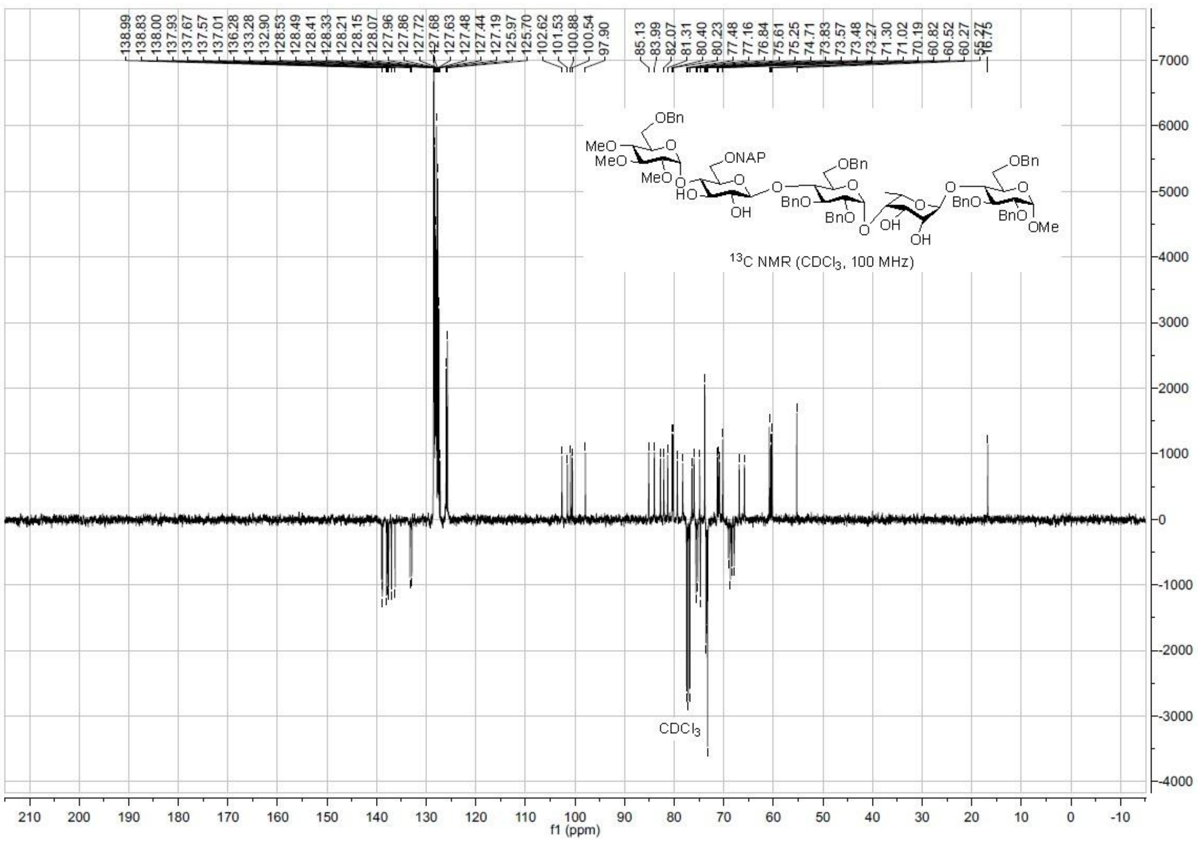
138.99
138.83
138.00
137.93
137.67
137.57
137.01
136.28
133.28
132.90
128.53
128.49
128.41
128.33
128.21
128.15
128.07
127.96
127.86
127.72
127.68
127.63
127.48
127.44
127.19
125.97
125.70
102.62
101.53
100.88
100.54
97.90
85.13
83.99
82.07
81.31
80.40
80.23
77.48
77.16
76.84
75.61
75.25
74.71
73.83
73.57
73.48
73.27
71.30
71.02
70.19
60.82
60.52
60.27
55.27
16.75
$^{13}$C NMR (CDCl$_3$, 100 MHz)
CDCl$_3$
f1 (ppm)

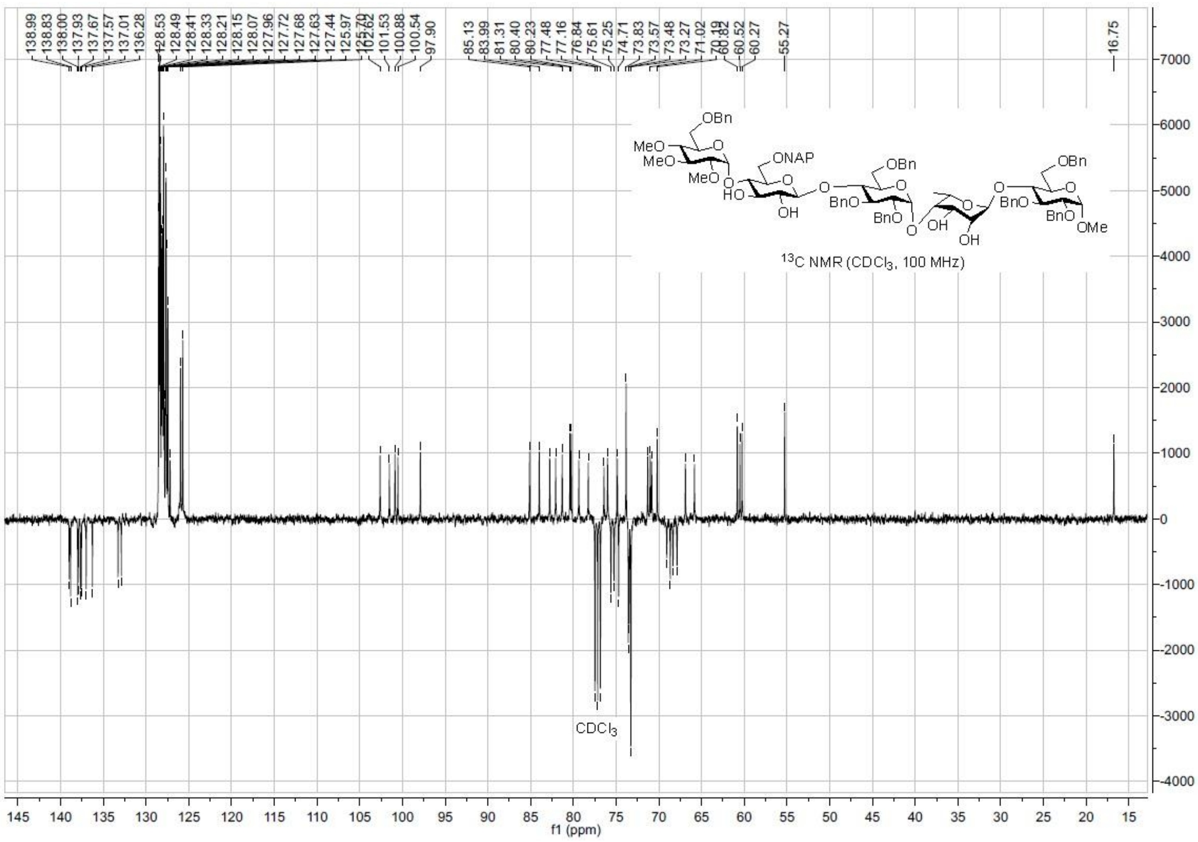
$^{13}$C NMR (CDCl$_3$, 100 MHz)
CDCl$_3$
f1 (ppm)

**$^{1}$H and $^{13}$C NMR spectra of compound 33:**

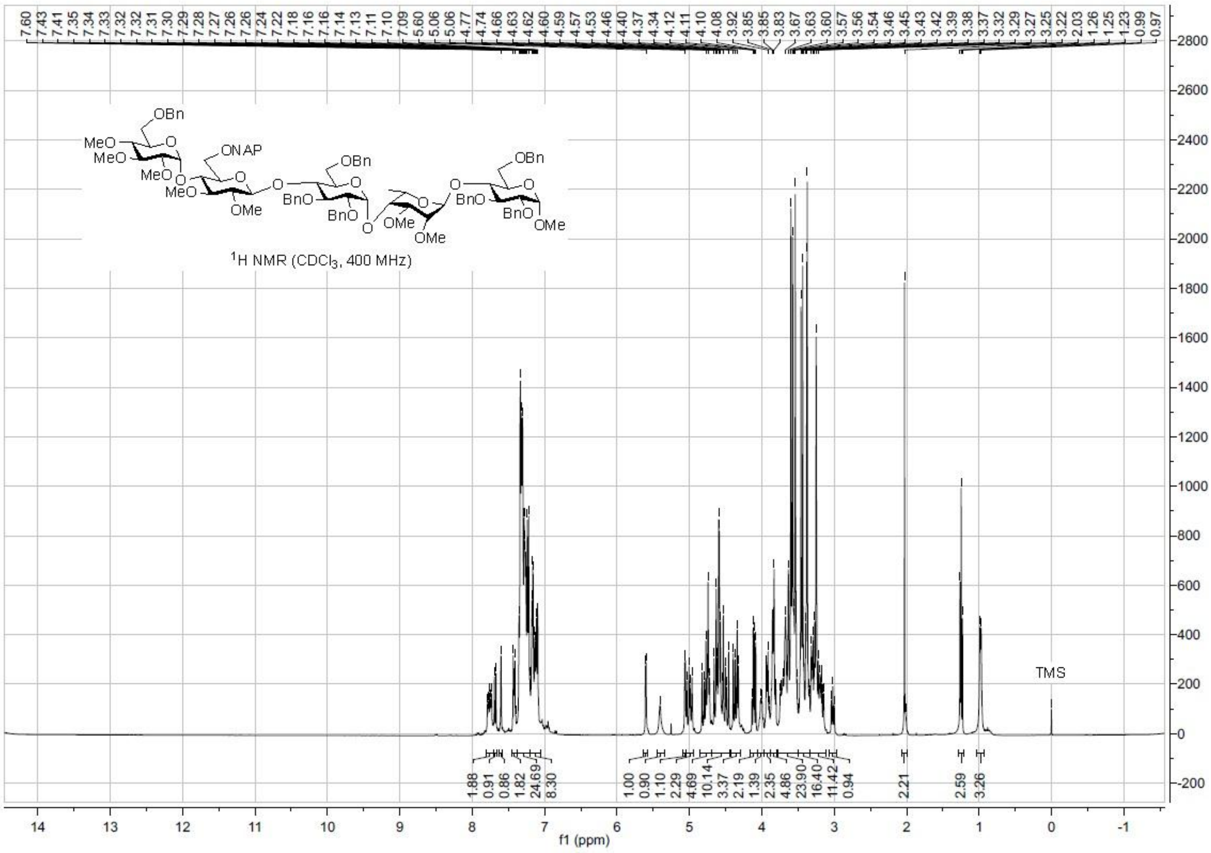


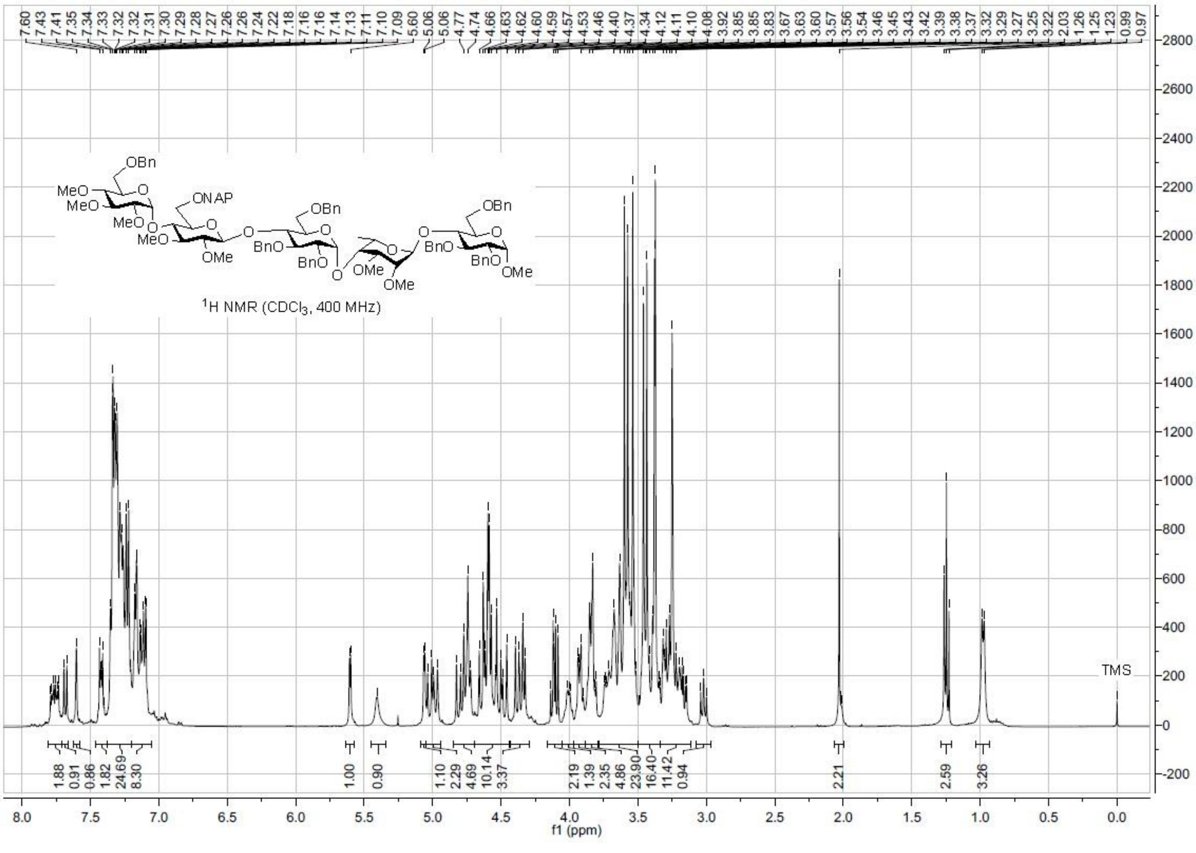

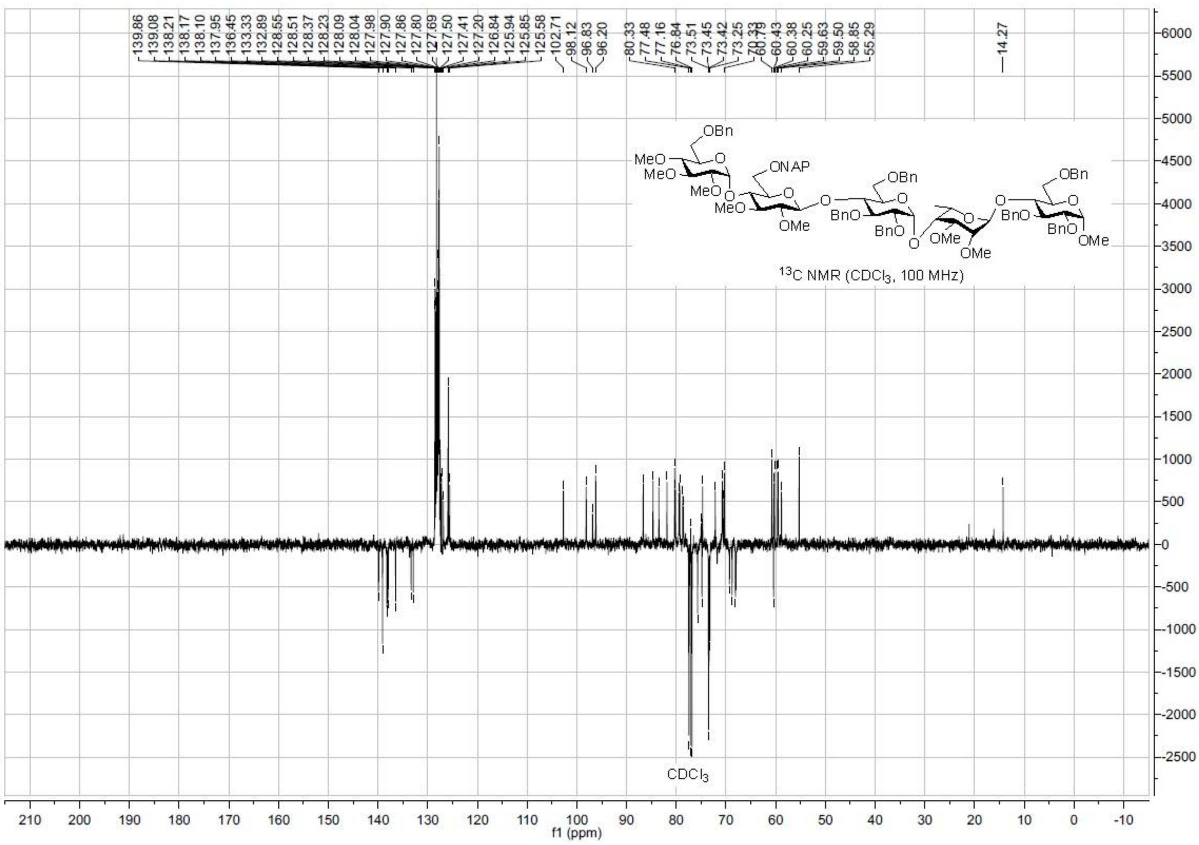
13C NMR (CDCl3, 100 MHz)
CDCl3
f1 (ppm)

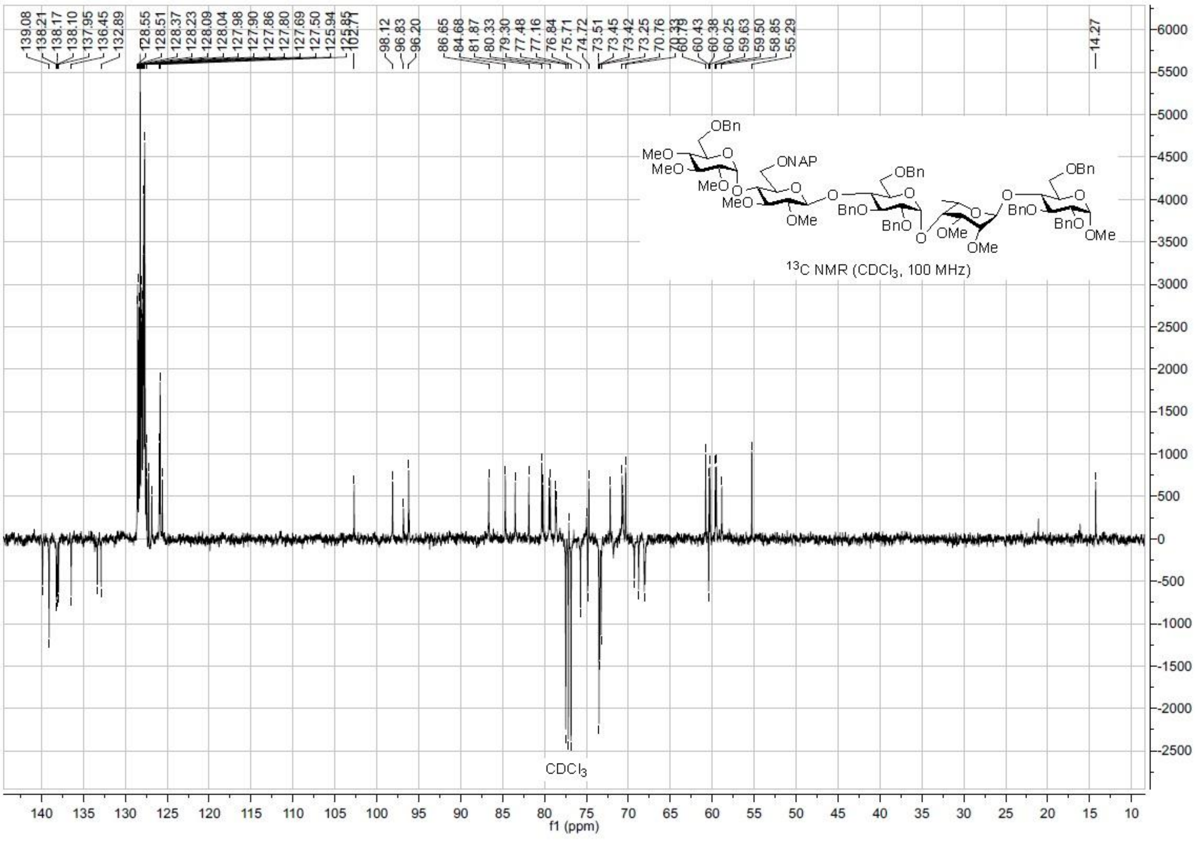
13C NMR (CDCl3, 100 MHz)
CDCl3
f1 (ppm)

**$^{1}$H and $^{13}$C NMR spectra of compound 34:**

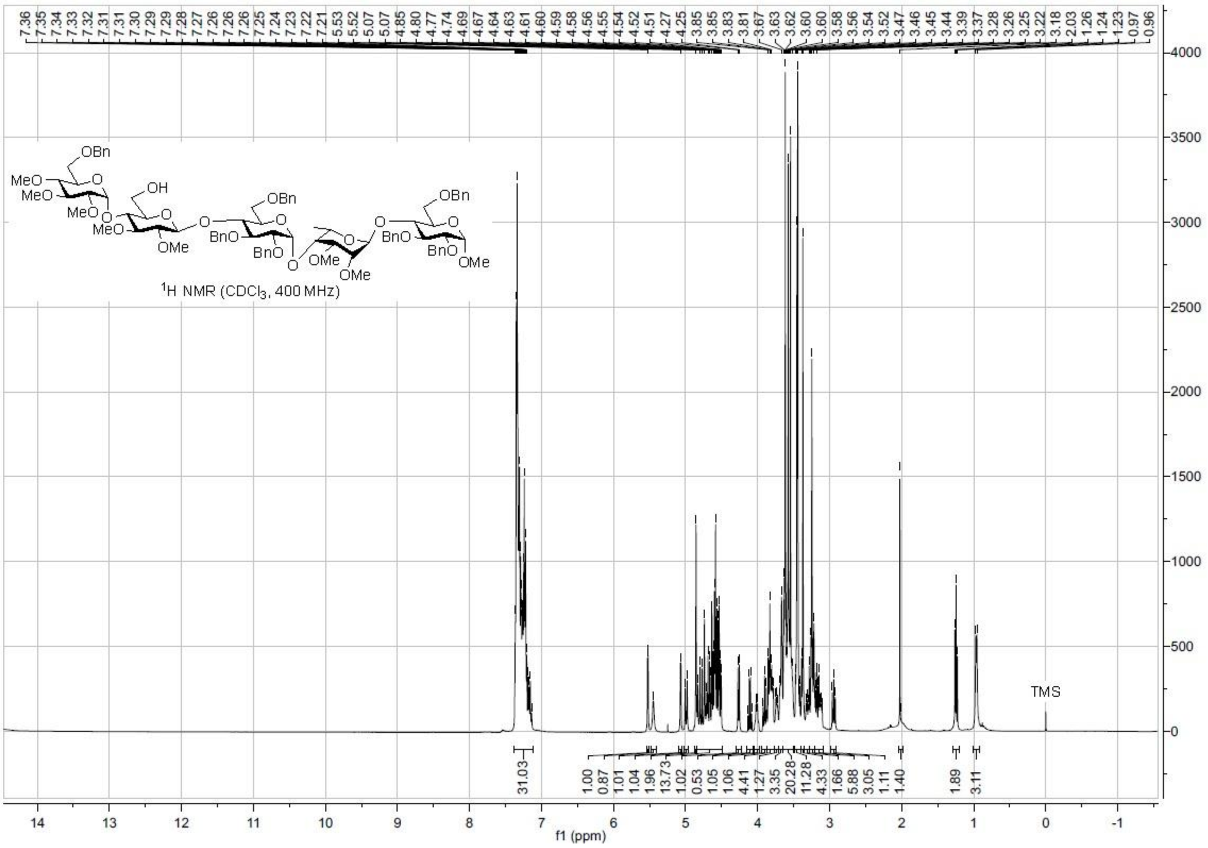


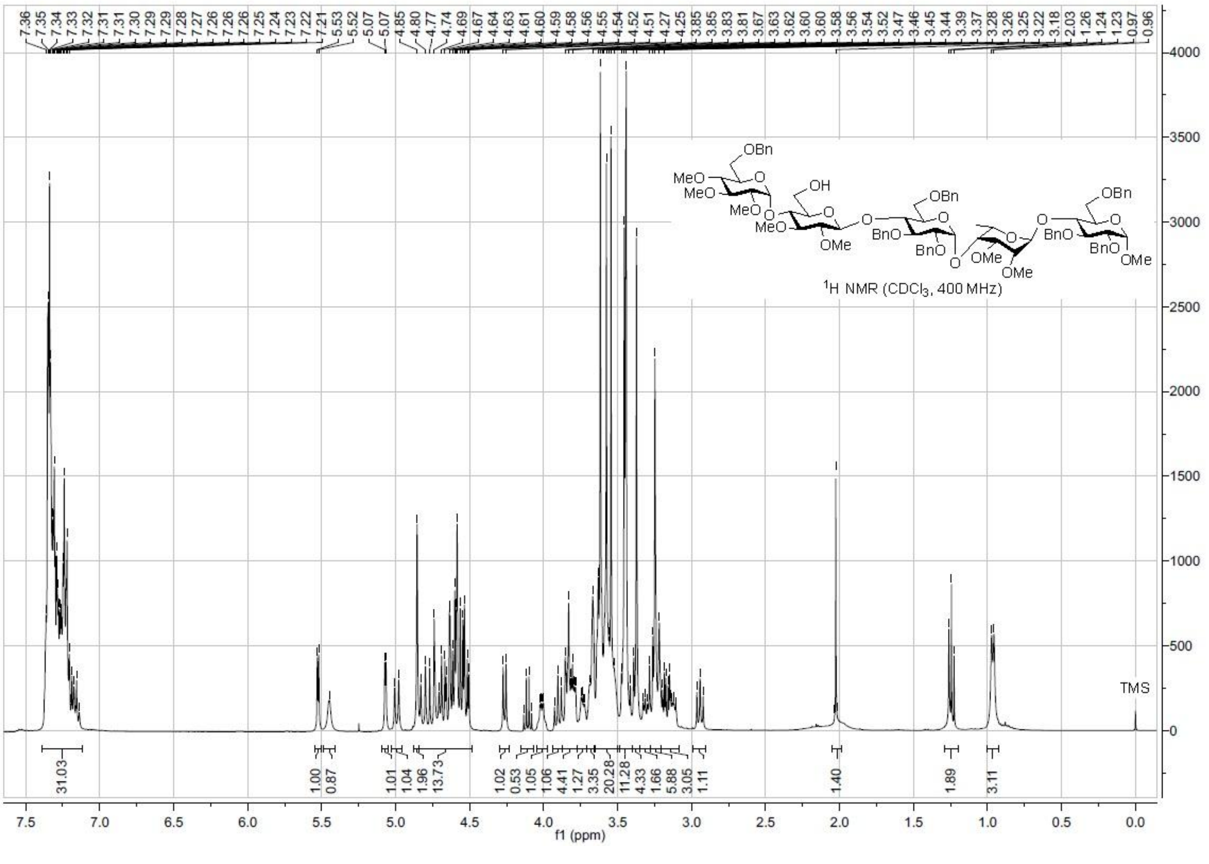

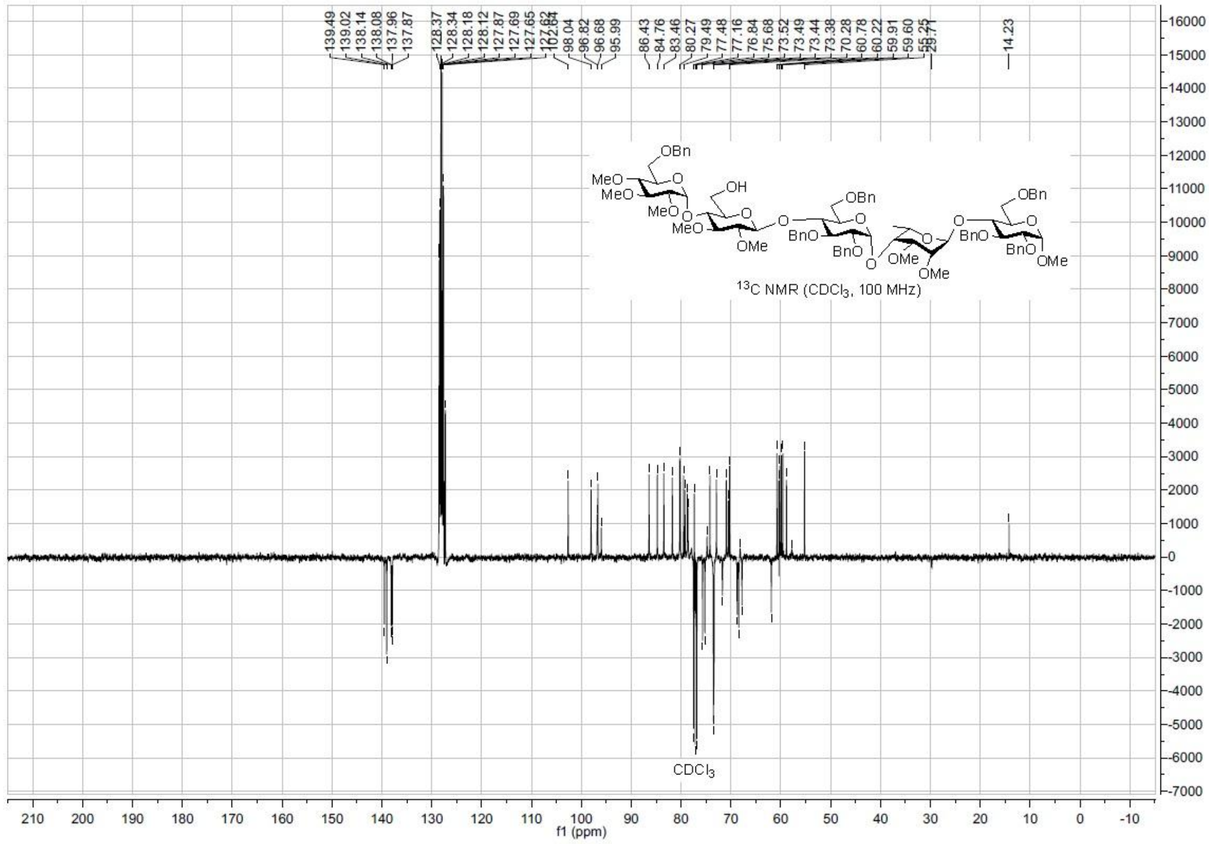

$^{13}C$ NMR ($CDCl_3$, 100 MHz)
CDCl3
f1 (ppm)


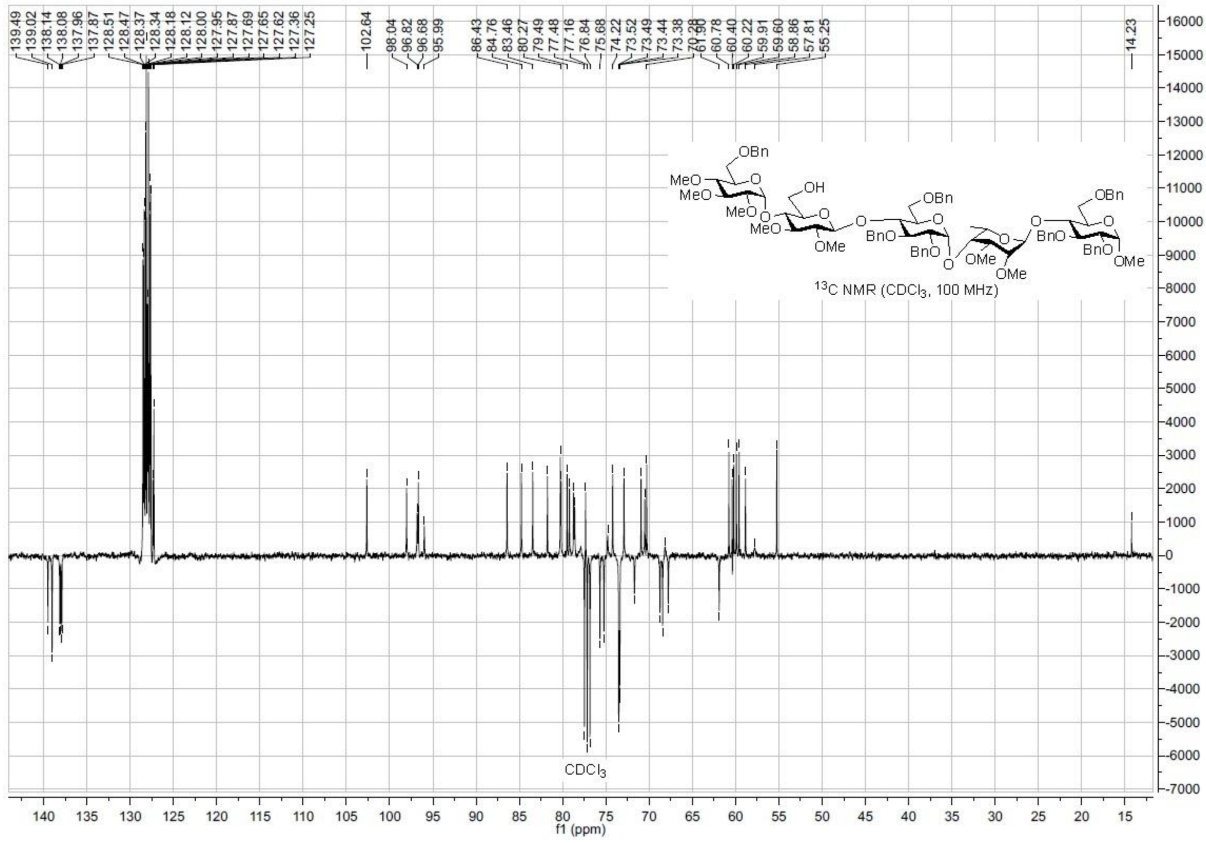

$^{13}C$ NMR ($CDCl_3$, 100 MHz)
CDCl3
f1 (ppm)

**$^{1}$H and $^{13}$C NMR spectra of compound 35:**

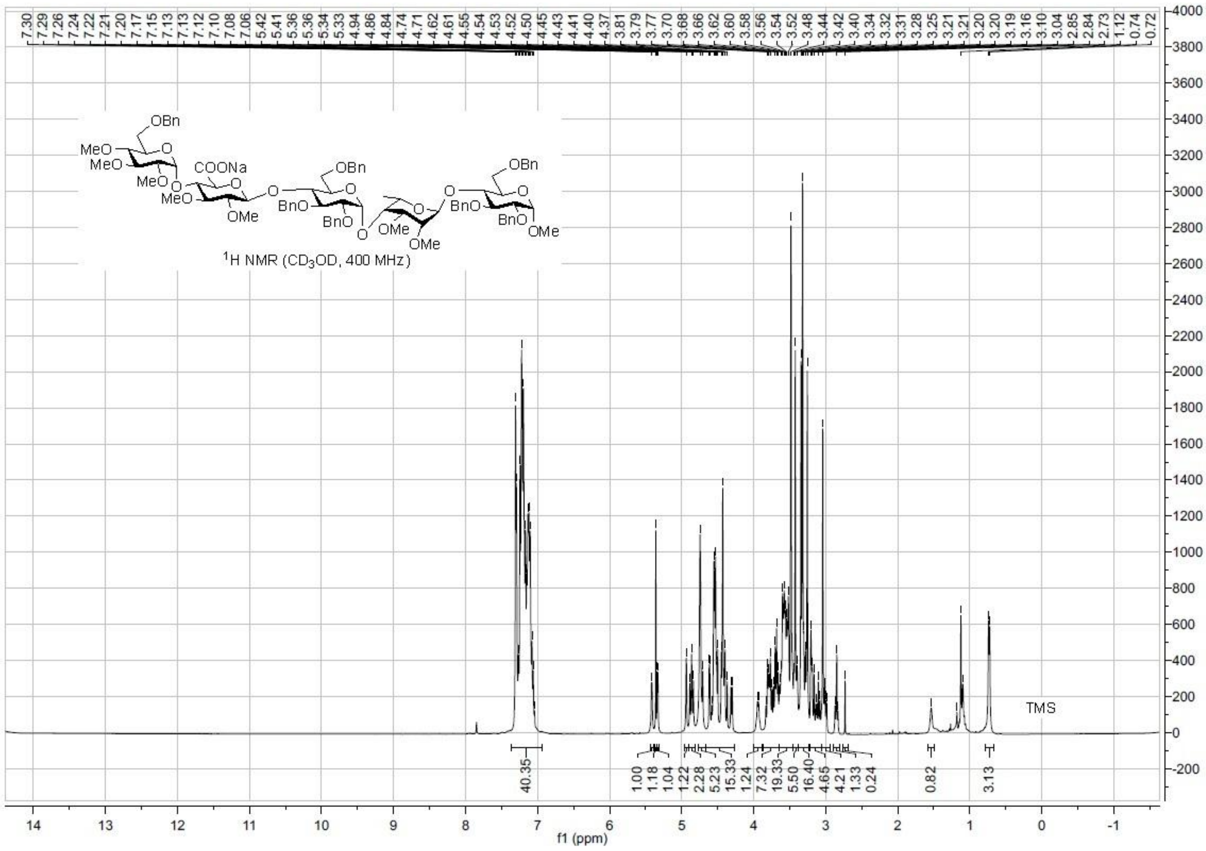


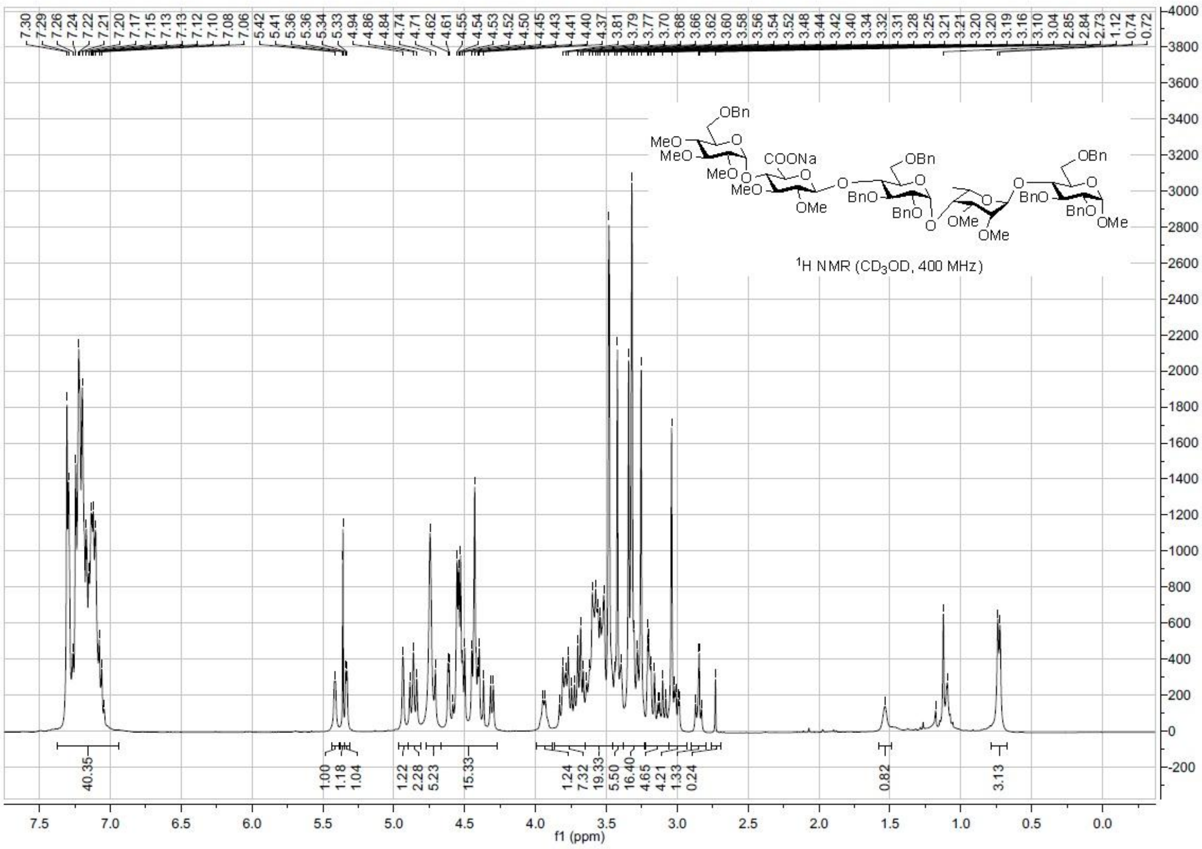

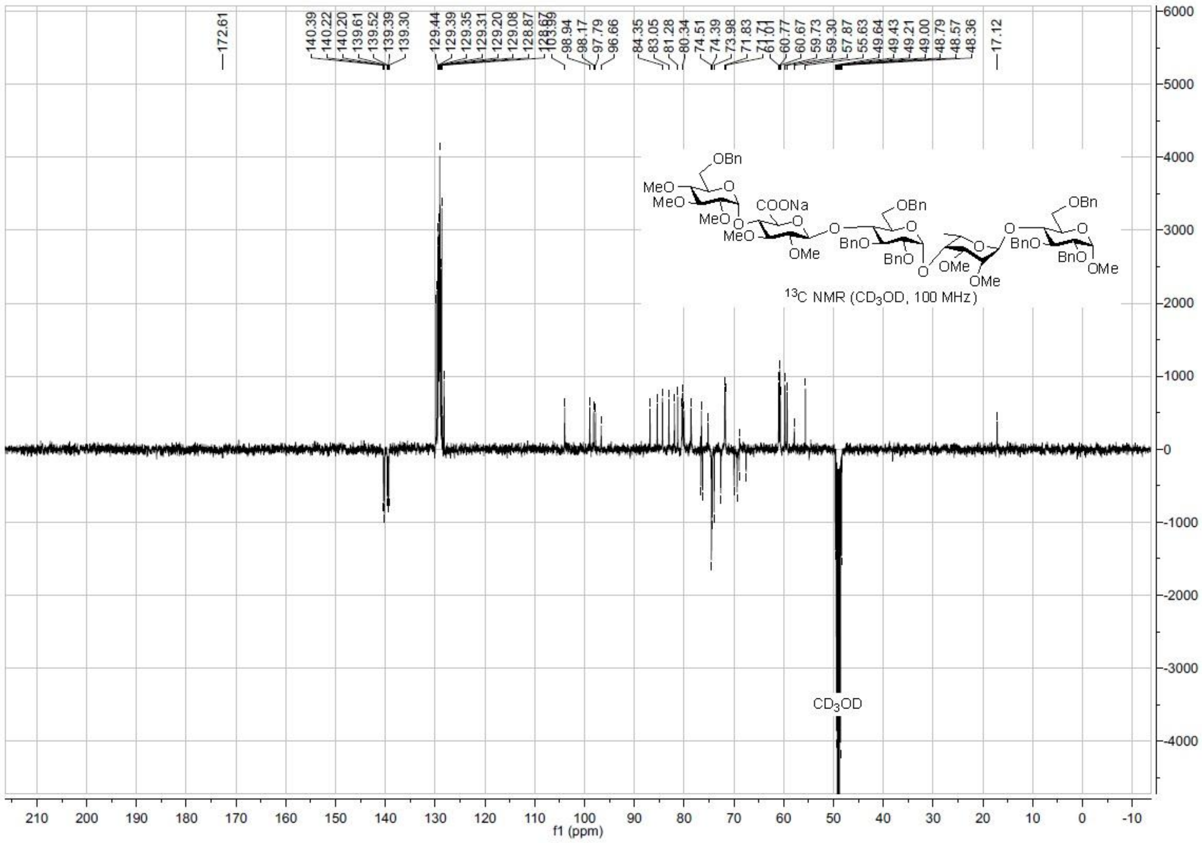
13C NMR (CD3OD, 100 MHz)
CD3OD
f1 (ppm)

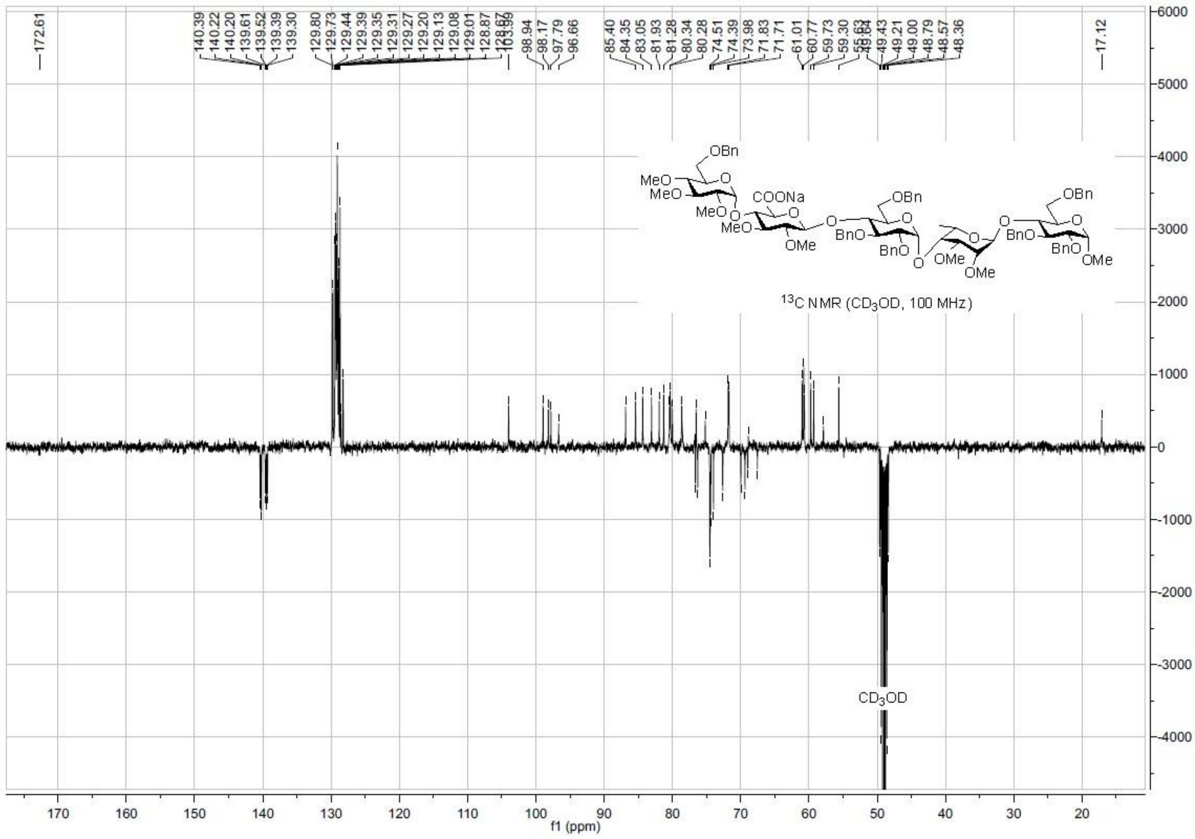
13C NMR (CD3OD, 100 MHz)
CD3OD
f1 (ppm)

**$^{1}$H and $^{13}$C NMR spectra of compound 36:**

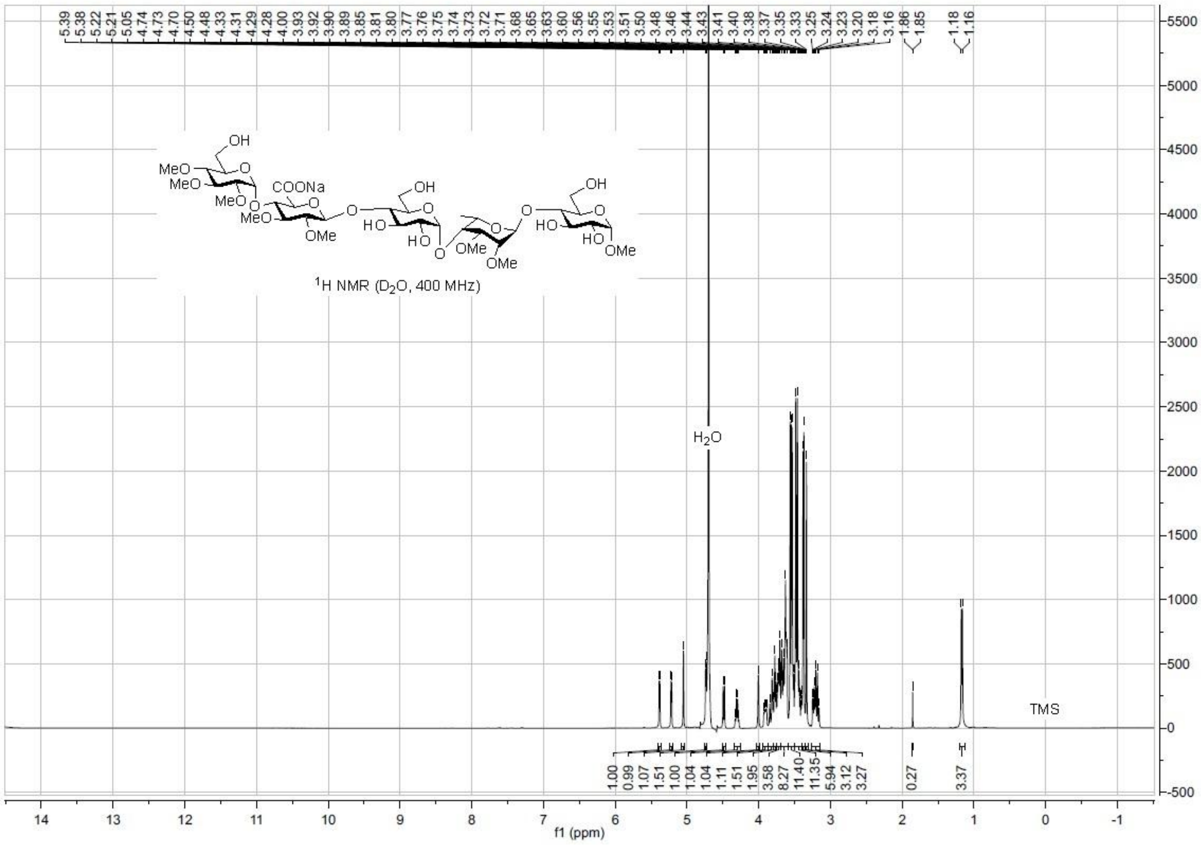


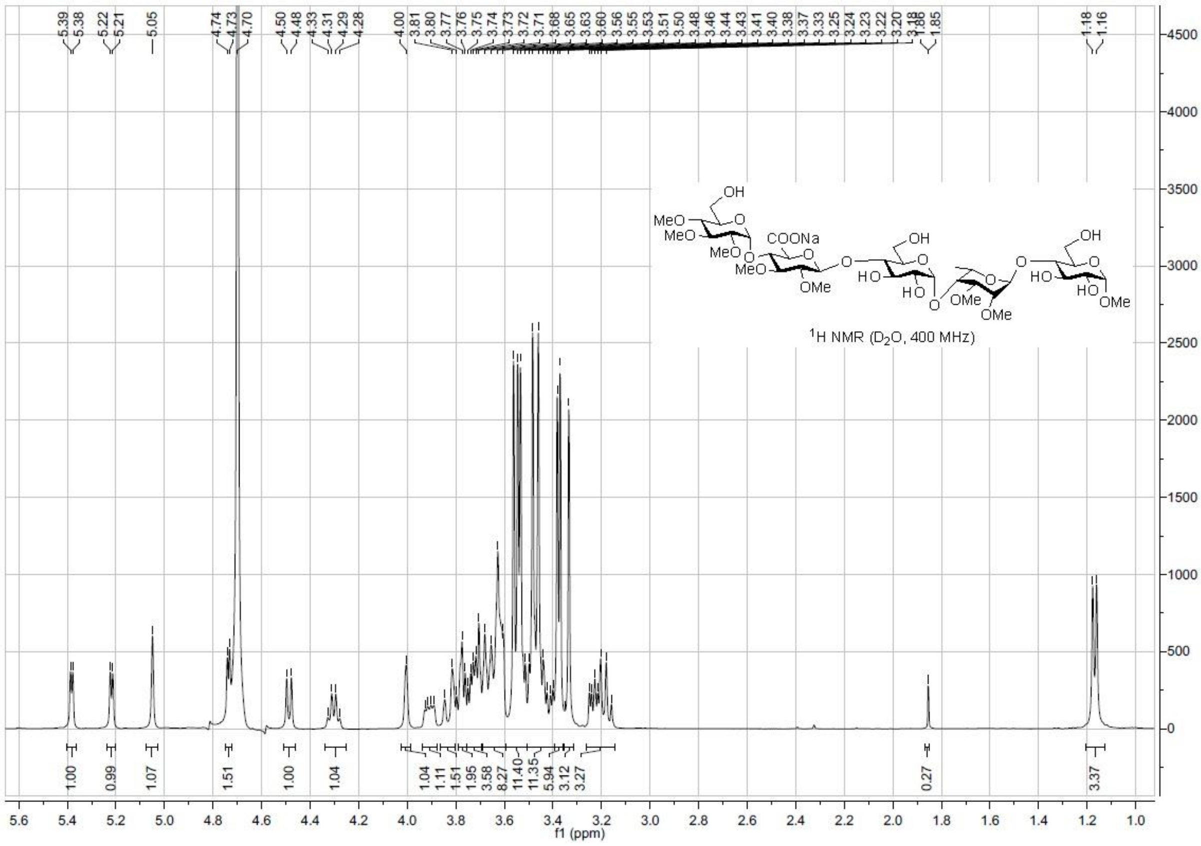

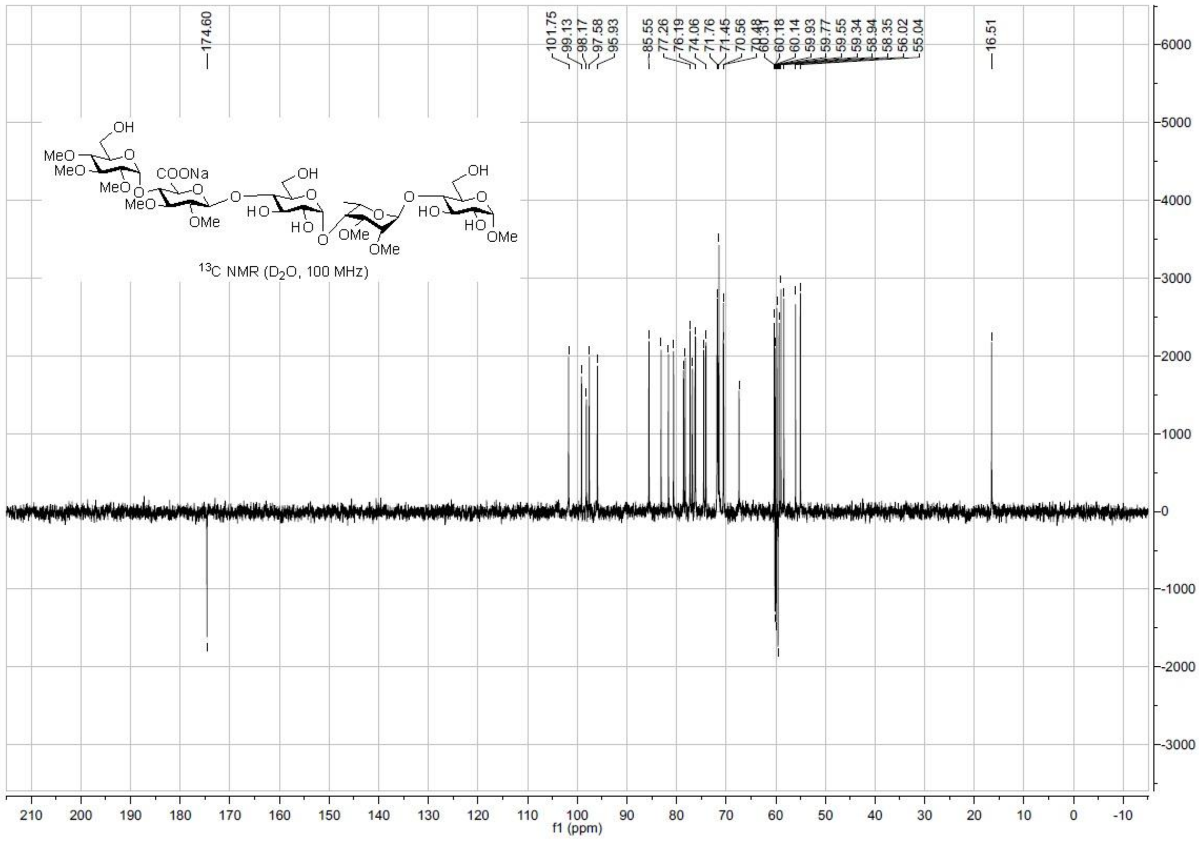
13C NMR (D2O, 100 MHz)
f1 (ppm)

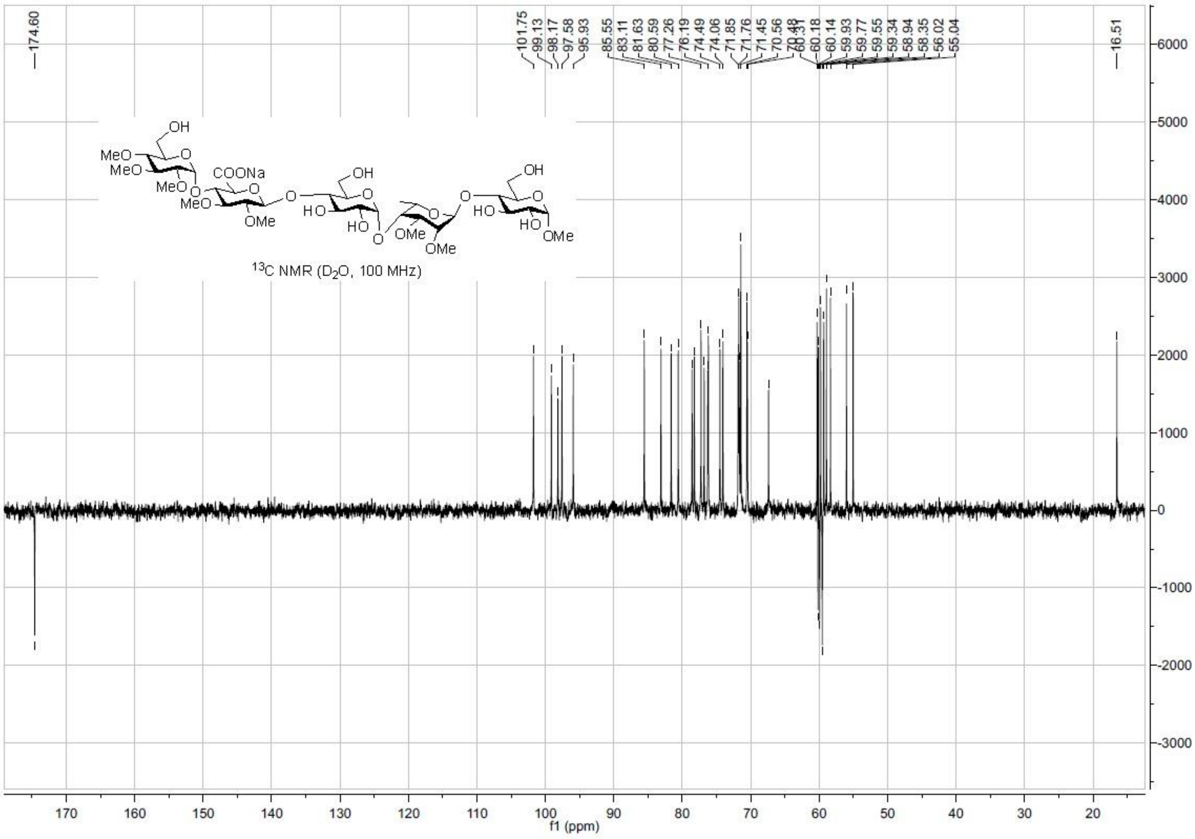
13C NMR (D2O, 100 MHz)
f1 (ppm)

## $^{1}H$-$^{1}H$ ROESY spectra of the target compounds

All two-dimensional (2D) $^{1}H$-$^{1}H$ EASY-ROESY[6] spectra were recorded on Bruker Neo 700 ($^{1}H$: 700.25 MHz) spectrometer at 25 °C using spin-lock mixing time of 300 ms.

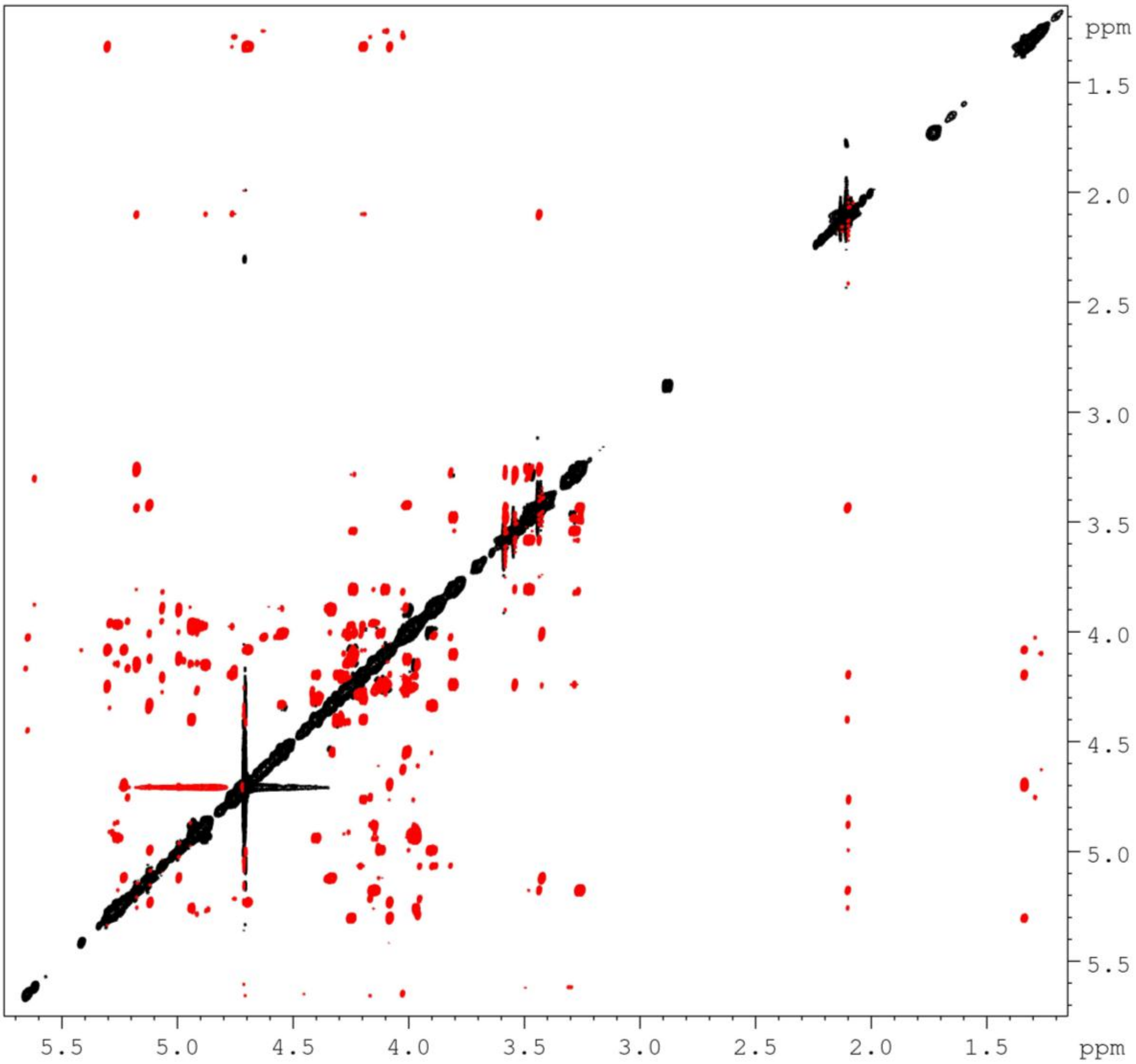

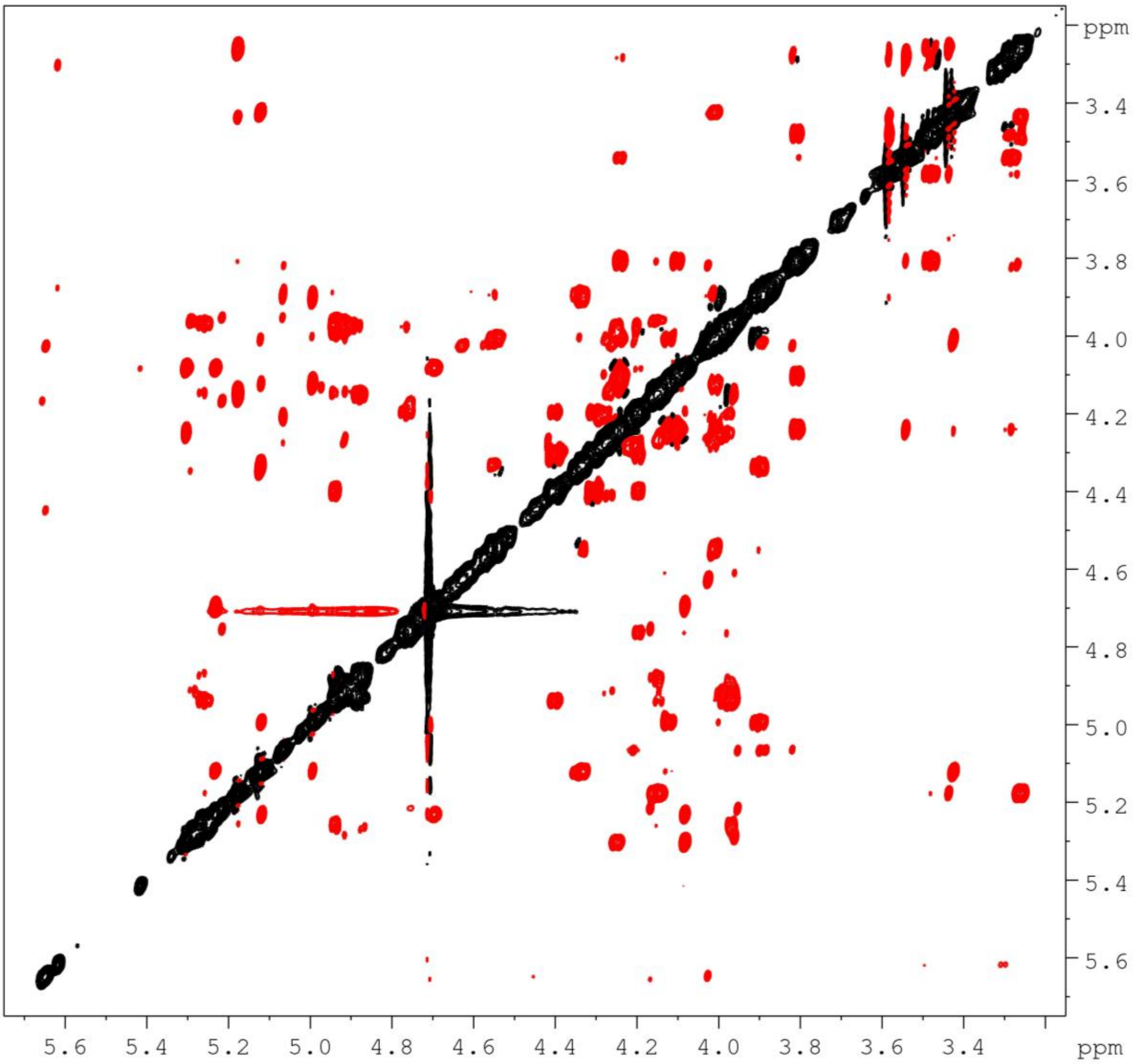


$^{1}H$-$^{1}H$ EASY-ROESY spectra of compound **2**

(top - full spectrum, bottom - excerpt)

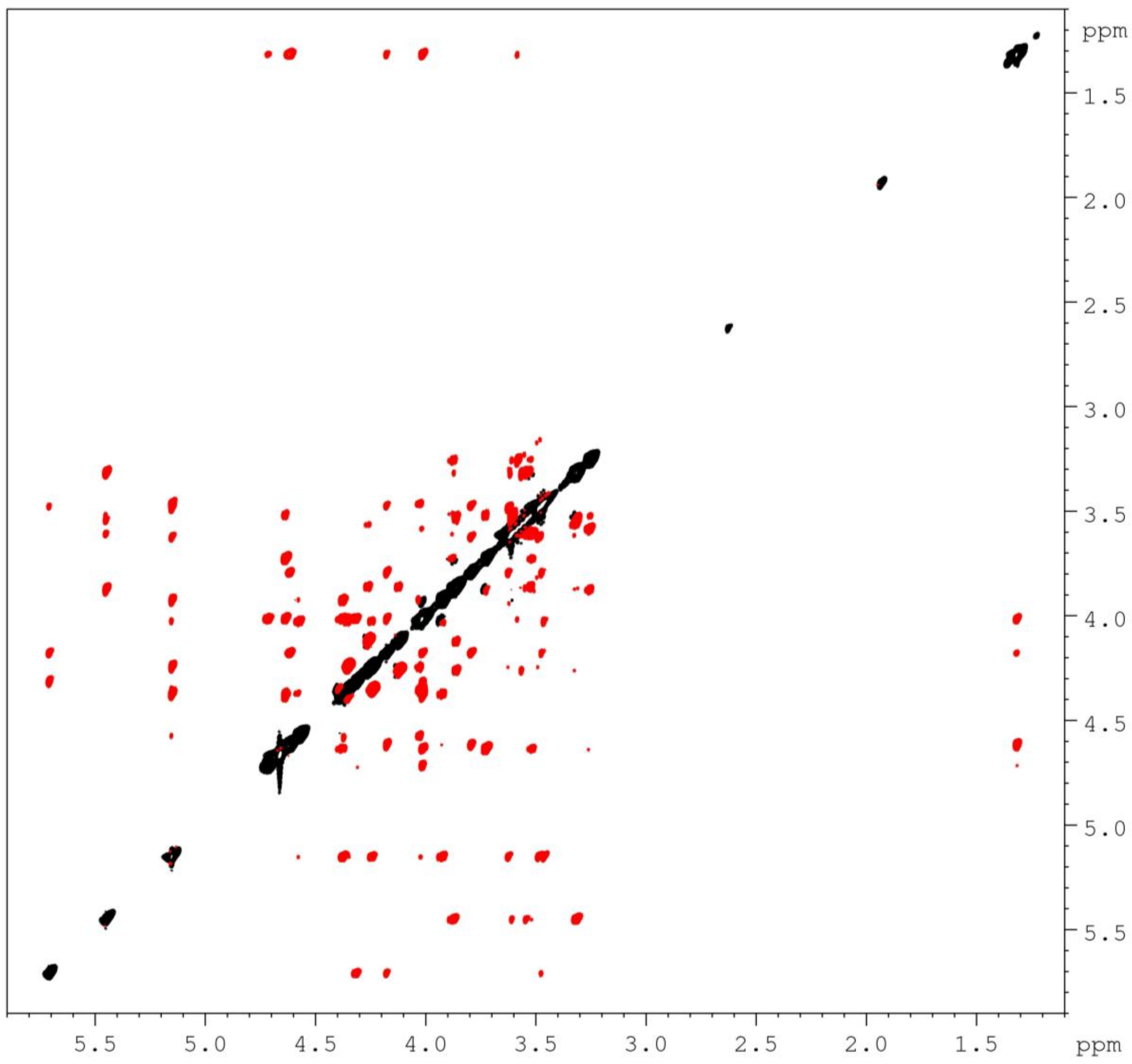
ppm
1.5
2.0
2.5
3.0
3.5
4.0
4.5
5.0
5.5
5.5
5.0
4.5
4.0
3.5
3.0
2.5
2.0
1.5
ppm

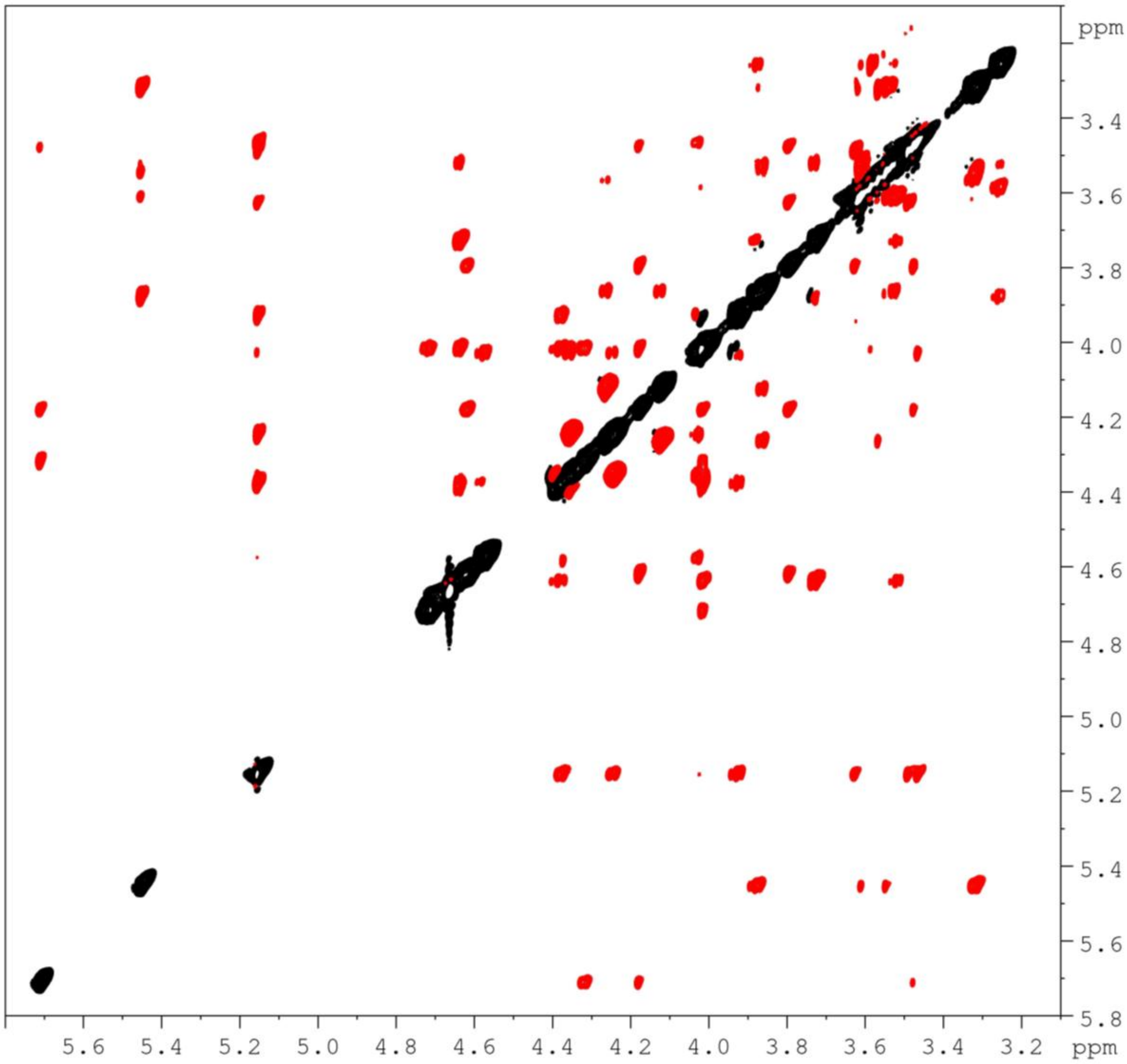


$^1H$-$^1H$ EASY-ROESY spectra of compound **3**

(top - full spectrum, bottom - excerpt)

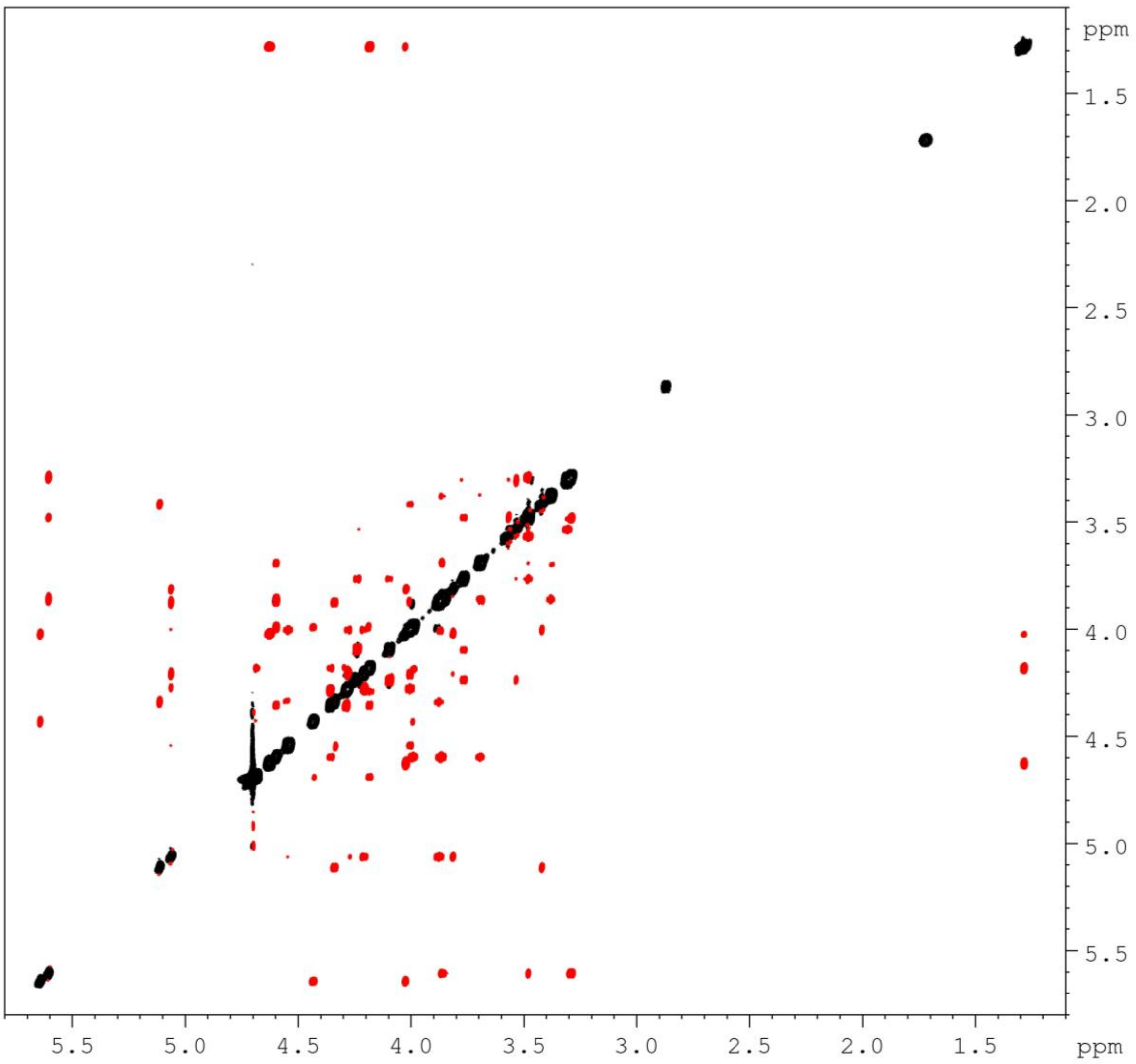

ppm
1.5
2.0
2.5
3.0
3.5
4.0
4.5
5.0
5.5
5.5
5.0
4.5
4.0
3.5
3.0
2.5
2.0
1.5
ppm

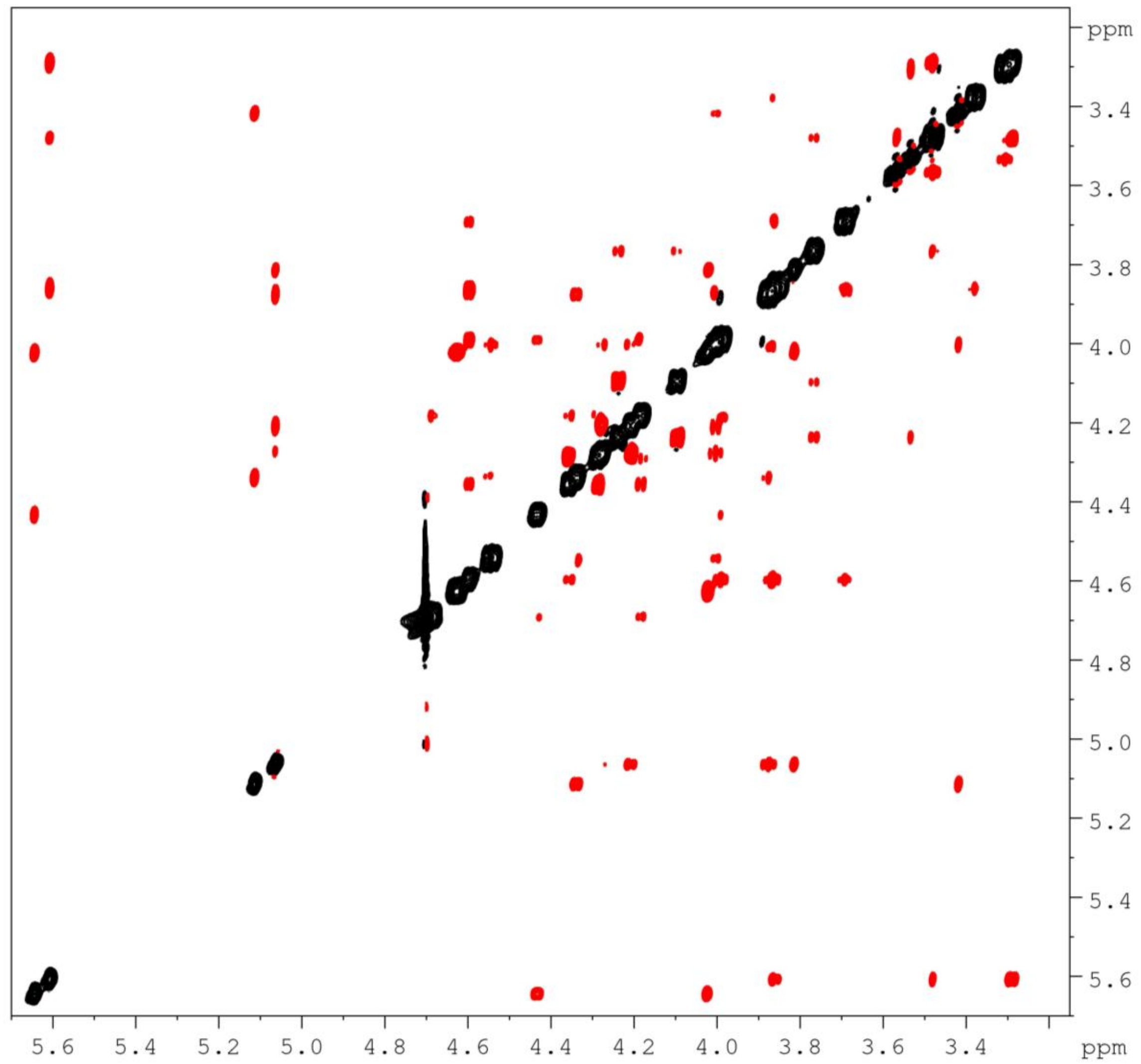


$^{1}H$-$^{1}H$ EASY-ROESY spectra of compound **4**

(top - full spectrum, bottom - excerpt)

## $^{1}H$-$^{1}H$ distances based on ROESY experiments (Table S2)

| H-H for unit G | ROESY derived $^{1}H$-$^{1}H$ distances (Å) | | |
|---|---|---|---|
| | **2** | **3** | **4** |
| H1-H2 | 2.6 | 2.6 | 2.4 |
| H2-H3 | 2.5 | 2.6 | 2.7 |
| H3-H4 | 2.4 | 2.4 | signal overlap |
| H4-H5 | 2.4 | 2.4 | signal overlap |
| H2-H5 | 3.5 | 3.6 | 3.2 |

**Table S2.** $^{1}H$-$^{1}H$ distances within G-unit estimated from the integrals of ROESY cross-peaks
